# Interacting Proteins, Polymorphisms and the Susceptibility of Animals to SARS-CoV-2

**DOI:** 10.3390/ani11030797

**Published:** 2021-03-12

**Authors:** John T. Hancock, Ros C. Rouse, Emma Stone, Alexander Greenhough

**Affiliations:** 1 Department of Applied Sciences, University of the West of England, Bristol BS16 1QY, UK; Emma4.Stone@uwe.ac.uk (E.S.); Alexander.Greenhough@uwe.ac.uk (A.G.); 2 Research, Business and Innovation, University of the West of England, Bristol BS16 1QY, UK; ros.rouse@uwe.ac.uk

**Keywords:** ACE2, COVID-19, furin, neuropilin-1, polymorphisms, SARS-CoV-2, TMPRSS2

## Abstract

**Simple Summary:**

COVID-19 is the disease caused by a coronavirus: SARS-CoV-2. The disease was declared by WHO in March 2020 as a pandemic and is still affecting countries around the world. Although considered a human disease, it is thought to have its origins in bats, and transmitted to humans potentially through an intermediate animal. What is not fully understood is the likelihood of the virus transmitting back to animal populations, either to companion or wild animals. For the virus to enter host cells, it needs to interact with particular proteins on the cell surface. Here we review the susceptibility of animals to the SARS-CoV-2 virus through the comparison of proteins between animals and humans. The most studied is the angiotensin-converting enzyme 2 receptor (ACE2), which has been used to rank the viral susceptibility of a range of vertebrates. Here, we also assess three other proteins. Of these, TMPRSS2 may be helpful in determining susceptibility, whereas the other two would appear to be of limited use. We propose that future work should examine changes seen in these proteins which alter the ease by which the virus can enter cells. This type of analysis may contribute limited evidence in predicting if animals are safe from such viruses and may help to guide future welfare concerns.

**Abstract:**

COVID-19, caused by SARS-CoV-2, is a world-wide problem for the human population. It is known that some animal species, such as mink, can become infected and transmit the virus. However, the susceptibility of most animals is not known. Here, we review the use of sequence analysis of the proteins which are known to interact with SARS-CoV-2 as a way to estimate an animal’s susceptibility. Although most such work concentrates on the angiotensin-converting enzyme 2 receptor (ACE2), here TMPRSS2 (Transmembrane Serine Protease 2), neuropilin-1 and furin are also considered. Polymorphisms, especially ones which are known to alter viral/host interactions are also discussed. Analysis of ACE2 and TMPRSS2 protein sequences across species suggests this approach may be of some utility in predicting susceptibility; however, this analysis fails to highlight some susceptible animals such as mink. However, combined with observational data which emerges over time about which animals actually become infected, this may, in the future, be a useful tool to assist the management of risks associated with human/animal contact and support conservation and animal welfare measures.

## 1. Introduction

COVID-19 is the disease caused by a new coronavirus called SARS-CoV-2, and the disease outbreak in 2020 was declared by the World Health Organization (WHO) as a pandemic [1]. COVID-19 is caused by an RNA containing enveloped virus, which interacts with its host cells by the use of spike proteins on the outside, giving the virus its characteristic image. The virus causing COVID-19 is principally transmitted through the population in respiratory droplets and on surfaces [2], leading primarily to respiratory problems, but also other pathologies, with complications such as a cytokine storm and eventual death not being uncommon [3,4]. SARS-CoV-2 is a zoonotic virus. The virus, when sequenced, appears to have similarities to a coronavirus found in bats. The SARS-Cov-2 virus genome has 96% percent identity to a virus from the bat *Rhinolophus affinis* [5]. The disease was first reported in Wuhan Province, China, where it was thought potentially to have passed to pangolins, although this has been questioned [6], and then humans. It spread rapidly through the human population and was declared by the WHO as a pandemic on 11th March 2020. At the time of writing (22nd February 2021), worldwide there are 111 M cases reported with 2.46 M deaths, and it appears that the pandemic will continue for some time, although vaccines have been developed [7,8] with the first ones approved for use in the UK and elsewhere.

However, as COVID-19 is caused by a zoonotic virus there is also the possibility that the virus can be transmitted to other animals (and in some cases this has already been the case). Here, the susceptibility of a number of animal species to SARS-CoV-2 is reviewed, with a focus on whether such susceptibility can be predicted and used to inform future practice and animal welfare and conservation. 

### 1.1. SARS-CoV-2 and Viral Entry into Host Cells

SARS-CoV-2 first has to enter a host’s cells to cause the disease. It is a spike protein containing enveloped virus, with the spike proteins being used to interact with the surface of the host cell. The process has been reviewed by others [9], so will only be covered briefly here.

The spike proteins consist of three regions: an extra-viral region; a transmembrane section; and a small C-terminal region. The proteins are heavily glycosylated, helping them evade the host immune system. However, the proteins have two domains which are crucial for viral host recognition and entry. S1 is a globular domain at the N-terminal end of the protein and is used for cell recognition. The second domain, S2, is used for cell fusion and viral entry into the cell [10].

The host protein responsible for recognition of the spike protein is the angiotensin-converting enzyme 2 receptor (ACE2) [11]. This is a metalloproteinase, normally involved in the regulation of vasoconstriction and therefore blood pressure. The entry of the virus involves the cleavage of the spike proteins. The S1/S2 cleavage site on SARS-CoV-2 is given as TQTNSPRRAR↓SVAS [12]. However, there are other proteases involved. Notable is the role of Transmembrane Protease Serine Type2 (TMPRSS2). It has been recognized that both ACE2 and TMPRSS2 are needed for SARS-CoV-2 entry into host cells [13]. 

More recently, other proteins have been identified as having roles in SARS-CoV-2 viral entry. Neuropilin-1 is a cell surface receptor reported to be important [14]. The fourth protein of note is furin [15], a polypeptide mooted as a possible therapeutic target [16]. This is a protease originally thought to be involved in the secretory pathway of cells, but has long been recognized as being important in disease, including viral disease such as Ebola [17]. However, there is doubt that the furin cleavage site in the SARS-CoV-2 protein is actually required for entry of the virus into cells in vivo [18]. 

In this paper, we focus on the infection of animals. If SARS-CoV-2 viral proteins are reliant on host proteins to both recognize their presence, and to process them, (in this case by proteolytic cleavage), then for the virus to be infectious in animals there must be a mechanism akin to that seen in humans. One possibility is that the same proteins are involved and that the regions and domains on those proteins will be the same, or least have a consensus amino acid sequence. Taking this approach, we aligned four major proteins involved in viral entry in 38 animal species to assess similarities and differences.

There are potentially thousands of cell surface proteins, and many of these have polymorphisms [19]. As discussed below, polymorphisms in proteins used by SARS-CoV-2 may increase or decrease virus host cell entry. Therefore, it might not be expected that important domains in animals are identical, but the presence of certain polymorphisms may be indicative of SARS-CoV-2 susceptibility, a hypothesis assessed here. It is interesting to note that such polymorphisms have been of interest in the treatment of COVID-19, such as the susceptibility to hydroxychloroquine or chloroquine [20]. In this case it was polymorphisms in TMPRSS2.

### 1.2. Animals Which Have Become Infected by SARS-CoV-2

One of the fears stemming from the fact that COVID-19 is a disease caused by a zoonotic virus is that the virus may be transmitted from humans to other animals, where it may transmit across populations of species and even back to humans, perhaps in a mutated form. This can happen [21], perhaps in a limited manner. Amongst the first animals to be reported to be SARS-CoV-2 positive were tigers in a New York zoo [22,23]: Malayan tigers (*Panthera tigris jacksoni*), Amur tigers (*Panthera tigris altaica*) and African lions (*Panthera leo krugeri*) all developed respiratory problems. Although it has been reported that cats and dogs are unlikely to be infected when in close proximity to humans [24], there is no doubt that domestic cats can become SARS-CoV-2 positive and transmit the virus across their populations [25,26]. A serological study in Wuhan [27] showed that cats could indeed become seropositive. Domestic dogs too are susceptible but seem to shed the virus less and are less likely to transmit it [28]. Abdel-Moneim and Abdelwhab provide a current overview of the status of SARS-CoV-2 infections in animals as of the 1st June 2020 [10]. Recently, four lions at Barcelona Zoo in Catalonia were reported as SARS-CoV-2 positive [29].

The major problem with SARS-CoV-2 infection which seems to have arisen in the animal populations are with mustelids [30]. It has been reported that not only do mink (*Neovison vison*) become infected with SARS-CoV-2, but the virus is transmitted within populations and back to humans. The SARS-CoV-2 virus has been found to have mutated in mink [21,31]. This has led to the euthanasia of thousands of animals and, along with mutations that have arisen in humans, raises concerns over the efficacy of any vaccine being developed [32].

There are several reports of animals being infected under laboratory conditions. Infection, accompanied by a cytokine storm, was seen in SARS-CoV-2 infected Caribbean vervets, for example [4]. In a study to determine if animals can be useful models of infection, ferrets, cats and dogs, along with several other animals, were used [33]. In ferrets there was poor inter-individual transmission, even though the virus infected the upper respiratory tract in these animals. Airborne transmission and deeper respiratory infection were seen in cats, whilst dogs poorly supported viral replication. Pigs, chicken and ducks seemed to have no infection. A summary of the animal infections recorded until October 2020 was given by Hobbs and Reid [34]. Ferrets, cats, Syrian golden hamsters and non-human primates all showed symptoms, with some evidence of infection in tree shrews (*Tupaia belangeris*) and fruit bats (*Rousettus aegyptiacus*). Non-human primate species studied include *Macaca mulatta*, *Macaca fascicularis, Callithrix jacchus* and *Chlorocebus aethiops*. As well as in the laboratory setting, it was also reported that 14 domestic animals (11 cats and 3 dogs) were positive for SARS-CoV-2, along with 8 captive wild animals (5 tigers and 3 lions). Nineteen mink farms also reported animals infected with the virus [34]. The USDA keep a database [35] of the animals found to be positive for the virus in the United States of America, and at the time of writing there were 11 tigers, 3 lions, 3 snow leopards, one cougar, 67 cats, 46 dogs, 3 gorillas and 16 mink listed. Recently it has been reported that pigs (*Sus scrofa domesticus*) are susceptible following oronasal inoculation [36]. Sixteen animals were tested, with viral RNA detected from the nasal wash and oral fluids from two animals and live virus from one. Antibodies were detected in two animals post inoculation, but were also found in the oral fluids indicating that there was viral secretion. Previous studies had reported that pigs were not susceptible [37,38]. Even though porcine cells lines were permissive to SARS-CoV-2, when a viral challenge was administered to five week old pigs (oral/intransal/intrattracheal) there was no susceptibility to the virus reported [37]. In the second study [38], intranasal inoculation of the virus was administered and it was concluded that pigs were not susceptible to SARS-Cov-2. The same study looked at ferrets (*Mustela putorius*), Egyptian fruit bats (*Rousettus aegyptiacus*) and chickens (*Gallus gallus domesticus*). Chickens were also found to be not susceptible, whilst 78% of the Egyptian fruit bats had transient infections. When contact animals were included in this study, one of the three introduced Egyptian fruit bats became infected. With ferrets more efficient transmission was seen than with the fruit bats as all three ferrets introduced became infected. It was concluded that the effects of the virus in ferrets resembled subclinical human infection with transmission between individual animals.

There is little doubt that some non-human species can become infected with SARS-CoV-2. Several groups have reported predictions of the susceptibility of animals to the virus, such as Kumar et al. [39] who tabled a susceptibility ranking of 66 species, based on ACE2 sequences. Ferrets (*Mustela putorius furo:* a mustelid related to mink) were ranked 53rd, although it is now known that mink are susceptible and transmit the virus [30]. This ranking is below rabbit, hamster and lion. Cat (*Felis catus)* was ranked 33rd and dog (*Canis lupus familiaris)* 56th. Non-human primates ranked highest, with gorilla (G*orilla gorilla)* being first. Camel (*Camelusferus)*, which was associated with MERS [40], was ranked 22nd for SARS-Cov-2 susceptibility. Other listed species of note include minke whale (*Balaenoptera acutorostrata:* 43rd, so higher than ferret), little brown bat (*Myotis lucifugus:* 62nd) and horse (*Equus caballus:* 64th). A more comprehensive survey was carried out by Damas et al. [41]. They classified species using the ACE2 receptor amino acid sequences, as very high, high, medium, low, or very low susceptibility. They assessed 410 vertebrates, including 252 mammals, but also birds, fish, reptiles and amphibians. As with Kumar et al. [39] great apes classified with very high susceptibility. Surprisingly, ferrets and mink were both listed in the very low category, even though there is substantial evidence of high SARS-CoV-2 infections in mustelids. Pangolins, which had originally been considered the intermediate host facilitating human infection, were also classified as very low, [6]. Several bat (*Chiroptera*) species were also very low. Of interest also are the marine mammals which were included in the study, several of which are listed in the high category, such as beluga whale (*Delphinapterus leucas*), dolphin (*Tursiops truncates*) and killer whale (*Orcinus orca*). Several rodents also frequent this “high” list, along with several species of deer. 

Others have also undertaken similar studies. Liu et al. [42] used this idea of studying protein similarities to look for animal species which may be intermediate hosts passing the virus to humans, and concluded that as well as pangolins and snakes, turtles ought to be considered. Further studies into the possible intermediate animal hosts include Zhao et al. [43].

Lack of correlation of classifying SARS-CoV-2 susceptibility with the ACE2 sequence data from animals may indicate that there are other proteins involved, or that amino acid changes are tolerated in the viral/receptor interactions, as suggested by Zhai et al. [44]. They also suggest that dogs and pigs have low susceptibility to SARS-CoV-2 due to low levels of expression of the ACE2 protein in the respiratory tracts of these animals.

Here, we align the sequences from four proteins thought to be instrumental to viral cell entry from a range of animal species. Amino acids which are known to either be in direct contact with interacting proteins, or have significant polymorphisms, are the focus of attention with a view to correlating this to known susceptibility to the SARS-CoV-2 virus.

## 2. Methods Used for Sequence Analysis

Representative animal species were chosen for a range of groups, including mammals, birds, reptiles and amphibians (38 in total, Table 1). The sequences for the four proteins (ACE2, TMPRSS2, neuropilin-1 and furin) were obtained from the *National Center for Biotechnology Information (NCBI)* database. In many cases there were several isoforms or variants available, those used here are listed in Table 1. For some animal species more than one was used. Alignments of the sequences were undertaken using Clustal Omega [45]. Clustal Omega is a multiple sequence alignment tool which uses seeded guide trees and Hidden Markov Model (HMM) profile-profile techniques. This tool’s core alignment engine uses the HHalign algorithm [46]. Sequence gaps were handled using the Gonnet transition matrix [47], with the following default settings: gap opening penalty—6 bits; gap extension—1 bit.

Amino acids highlighted from the literature as either being important for the catalytic sites of the proteins, or had previously been found to be important for SARS-CoV-2 binding, are listed in Table 2. Polymorphisms that have been reported by others as having an effect on protein function, as regards SAR-CoV-2, are also listed in Table 2 and are the focus of further discussions. Some polymorphism analysis was undertaken using gnomAD [48]. The Genome Aggregation Database (gnomAD) brings together data from multiple large-scale sequence alignment projects and uses the human GRcH37 reference sequence for alignments. The default settings were used for the data retrieval in this study. gnomAD was only used to ascertain if there are any other common polymorphisms listed that were not discussed by others previously. All amino acid numbering used here is based on the human sequences.

## 3. Sequence Analysis

There are reports of proteins other than ACE2, TMPRSS2, neuropilin-1, furin being involved in the viral entry to host cells. This includes a protein associated with tight junctions (PALS1) [63] and the endosomal/lysosomal cysteine proteases cathepsin B, L (CTSB, CTSL) [64]. *ADAM17, RPS6, HNRNPA1, SUMO1, NACA, BTF3* may also have a role [65]. Despite this, here, the focus is on four main proteins thought to be involved: ACE2, TMPRSS2, neuropilin-1 and furin [12,14,15,16], which are used for alignments and the data discussed. ACE2 was chosen as it is seen as an instrumental protein in the viral entry into cells [11]. There are also several polymorphisms which are reported to correlate with SARS-CoV-2 susceptibility (Table 2). Neuropilin was selected as it has very recently been found to be involved [14]. TMPRSS2 is also reported to be a key protein [13], particularly its protease activity, and again there are significant polymorphisms reported (Table 2). Along with furin, also reported as an important protein for virus/host interactions [15], it is hoped that analysis of these proteins, as well as ACE2, may give an insight into how the proteins may be used as susceptibility indicators for the virus. All four proteins chosen have had key amino acids highlighted as being of importance in the virus interaction or protein function. If such key amino acids were found to differ in any protein sequences which could be correlated to initial viral susceptibility of animals from specific species, or how infection progresses within individuals from different species, this would give a focus for further studies, including structural analysis. Phylogenic analysis was carried out using the auto-function of Clustal Omega which creates a simple phylogenetic tree using neighbor joining.

### 3.1. ACE2

**Figure 1:**
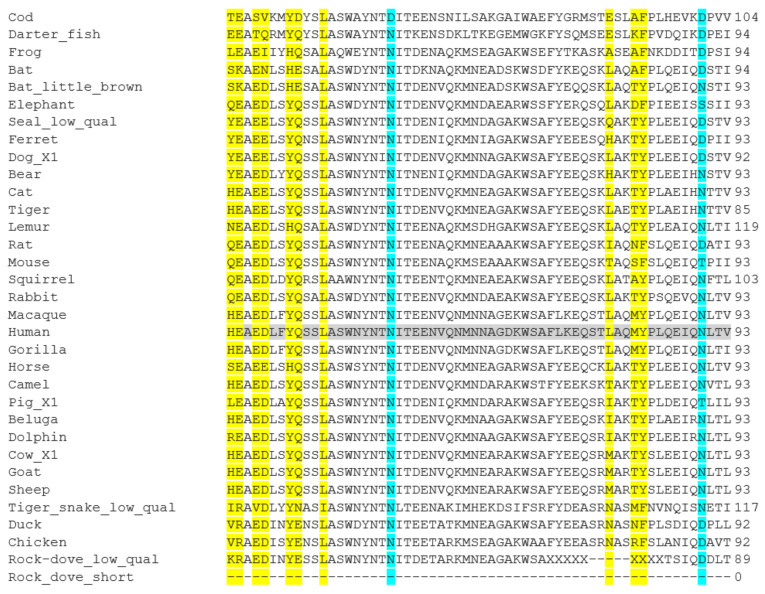
Amino acid sequence alignment of ACE2 polypeptides from a range of animal species: a short section encompassing two of the glycosylation sites is shown here. Full sequence alignments are shown in Supplementary Figure S1. Accession Numbers of the polypeptide sequences used are given in Table 1. Alignments were carried out using Clustal Omega [45]. Human sequence is highlighted in grey to aid comparison to other sequences. Important amino acids used for interaction with the SARS-CoV-2 viral proteins as reported by Damas et al. [41] (Table 2) are highlighted in yellow, with the highlighted glycosylated residues in blue.

Once the receptor which interacts with SARS-CoV-2 had been identified, there is the question as to which amino acids are important for the direct contact of the ACE2 and the spike proteins. Structural analysis of the interaction has been reported [50,66], whilst others have used an antibody approach to study this interaction [67]. Based on such data from several groups [36,38,48,63,64], lists of such amino acids have been created (Table 2). Damas et al. [41] highlight 25 amino acids across the 805 residues in the sequence. Sun et al. [51] have added four others: Q325; E329; R692; R710. The second question, which is the focus here, is whether these residues are conserved across animal species and whether this relates to SARS-CoV-2 susceptibility. All 29 amino acids are highlighted in the alignments carried out for the 38 animals used here (Supplementary Figure S1 and Figure 1). As discussed above, this analysis has been carried out by several groups for ACE2 [39,41,50,51,52]. As can be seen (Figure 1), it is no surprise that animals which have a close relationship to humans, such as non-human primates, have no difference in the ACE2 amino acids highlighted to be important, suggesting that they may be susceptible to SARS-CoV-2. On the other side of the evolutionary spectrum (only vertebrates analyzed here), the sequence differences indicate that birds, fish, amphibians and reptiles are all predicted to be safe from SARS-CoV-2, with the caveat that turtles have been suggested as a possible intermediate host in bat to human virus transmission [43]. Although some highlighted amino acids such as R393 are completely conserved, many animals have differences within the amino acids deemed to be of significance. For example, in the section shown in Figure 1, L45 is conserved, but H34 is very variable, appearing mainly in non-human primates and large mammals. However, H34Y and H34S substitutions are thought to decrease susceptibility [68]. H34S is seen in bat (*P. kuhli*), whilst H34Y is found in dog, bear, seal and ferret (contrary to mustelids being infected in farms). Interestingly, little brown bat (*P. kuhli*) has 8 differences in the region, when just looking at the highlighted amino acids, suggesting that susceptibility would be low. It may be more pertinent to concentrate on the glycosylated amino acids: N53, N90, N322. In the region in Figure 1, N53 is conserved except in cod. N90 is much more variable, often being changed to D. It is changed in birds, fish and amphibians, and even rodents, but the significance of this may be brought into focus when it is seen that it is also missing as an N in ferret and dog, both groups which are known to be susceptible to SARS-CoV-2. N322 is also well conserved, but is variant in fish, amphibian and dove. Interestingly, N322 is also variant in rodents and some farm animals, hinting that this may give some protection. Furthermore, the ACE2 protein of some animals have additional glycosylation [51]. In chicken residue L79 is a potential *N*-glycosylation site. M82 is a potentially glycosylated in rat, the horseshoe bat (*Rhinolophus sinicus*) and pangolins, whilst this post-translational modification prevents binding of spike protein to rat ACE2 [69]. As glycosylation is on the outside of cells and therefore can have a significant impact on the interactions taking place on the cell surface, such glycosylations will no doubt be important to focus on in the future.

Damas et al. [41] tabulated such data, including using some structural determination, into five categories: very high, high, medium, low, very low. As would be expected, non-human primates and some marine mammals were rated high, but the ranking did not particularly match reality, with ferret and mink classed as very low. This is an extremely useful overview of the ACE2 analysis, however there are doubts as to its usefulness, taken alone, for those seeking evidence which contributes to our understanding of which animals are likely to place humans, or other animals, at risk due to close contact. Whilst the findings in relation to fish and chickens, for example, appear to be supported by real world data, others ranked as relatively low in reality can become infected with SARS-CoV-2 (for example mink, in a real world context and pigs, at least in a laboratory setting. However, other animals predicted by the analysis to be susceptible have proven to be so, and therefore need to be treated with extra precautions (for example great apes)).

One region of ACE2 of interest is the short section 353-KGDFR-357 [57]. It was reported to be invariant in a range of mammals, and other animals except snake. In the alignments here it can be seen that it is indeed variant in snake, but also in birds, fish and amphibians (Supplementary Figure S1). It is not completely conserved across mammals either, with substitutions seen in rabbit, mustelids and seal.

As with all proteins there seem to be a range of polymorphisms listed for the ACE2 polypeptide [53,54,55,56,57,58,59,60]. Some of these are thought to increase or decrease SARS-CoV-2 spike protein affinity. A list of these relevant polymorphism was collated by Senapati et al. [49] and reported by others, along with some which can be found on the gnomAD site [48] are given in Table 2. Looking at those that decrease affinity first, S19P [53,60], S19 is variable across animals but none have P as a variant. However, others have suggested that the S19P substitution increases viral protein binding [56]. T27A is listed as both increasing and decreasing affinity [49], so it may be hard to use this as a measure, although MacGowan et al. [58] suggests it gives an increase. Although the substitution was not seen in any animal studied here, T27 was missing in snake, duck, dove, fish, and frog, and also missing in bats. K31R and N33I are again variable but not represented as variants seen in animals. E35K is seen in bats, suggesting this may indicate low affinity in these animals. D38V is only seen in cod, whilst Y50, N51 and D509 are invariant. Q388L is seen in fish, again, hinting perhaps at lower affinity. For other potential amino acid changes at the listed polymorphic sites there are some variation but none match the residue substitutions which would decrease affinity.

In the list which are thought to increase affinity for the spike proteins I21V is seen in frog and darter fish, but is next to an inserted D. M378R is invariant across species, whilst other residues listed are quite variable. Perhaps of relevance is K26R [53] seen in elephant, bat, frog and cod (although it was also reported that this substitution decreased affinity [61]); N64K in darter fish; T921I in elephant, ferret, mouse and pig; G326E in dog, bear and beluga; R559S seen in mink, ferret, rat, mouse, horse, camel, pig, elephant. Therefore, out of the residue changes which might increase spike protein affinity, mustelids and rodents contain two out of the fifteen listed, whilst elephant has three. The significance of this is not at present known but such changes may possibly contribute an increased viral susceptibility. 

Ali et al. [61] listed five substitutions in ranked order of increasing strength of binding. Looking at the 38 species analyzed here (Supplementary Figure S1) I468V was invariant in the analysis here except in dove, where it was C; R219C was only changed in darter fish (a G); K341R was a Q in frog and bat; D206G was N in several species, including beluga; G211R was never seen across the species seen here, but W was a quite common substitution, being seen in dog, cats, ferret and bat. Therefore, none of these substitutions particularly point to an increased susceptibility. 

**Figure 2:**
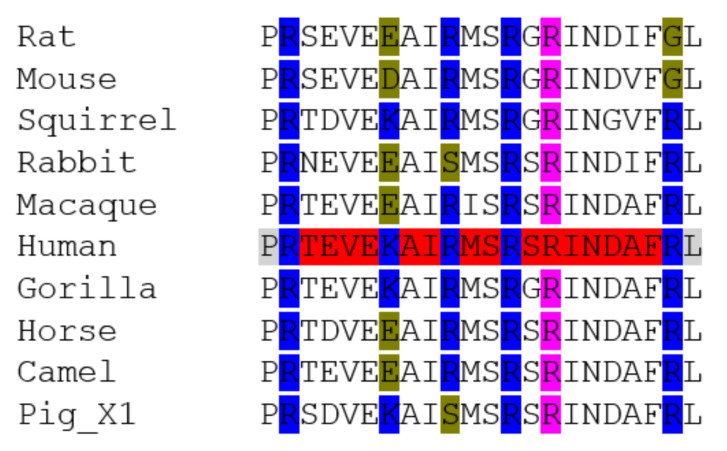
The alignment of the cleavage site of ACE2 (region 697–716). Human cleavage site is highlighted in red. Significant arginine and lysine residues are in blue with differences in animal species shown in brown. Grey highlights indicate human sequence.

As well as interacting proteins and polymorphisms, there is also a cleavage site highlighted in ACE2 [59]. Of note here are the Arg and Lys residues in the region 697 to 716, which are required for cleavage. A small section of the alignment of this region shows that in some species there are differences, as shown in brown here (Figure 2) (sequences for all animals in this study are shown in Figure S1):

Although the gorilla sequence is identical to humans with regard the basic amino acids (there is a S/G change), there are differences in the R and K residues in other species, such as rat and mouse, with conserved R/K changes in some species (not shown here). Such amino acids changes suggest that the mechanism of ACE2 modification, and therefore viral entry, may be less efficient in some species, such as rodents and rabbit.

In summary for the ACE2 sequences, it can be seen that there are similarities to humans in some species suggesting that they may be susceptible to SARS-CoV-2, such as non-human primates, as reported by others (for example, [41]). Melin et al. [54] used an alanine scanning mutagenesis analysis to identify important ACE2 amino acids for SARS-CoV-2 susceptibility, and then adding data from others [55], they focused on non-human primates. It was concluded that catarrhines, i.e., all African and Asian apes and monkeys are likely to be susceptible. On the other hand, it was suggested that American monkeys and some other primates, such as lemurs, may be less susceptible. There are also significant differences across animal groups when the ACE2 sequences are analyzed, suggesting that some species such as fish, amphibians, birds and reptiles may not be susceptible to SARS-CoV-2 infection. For other species, it may be hard to use ACE2 protein sequences as a prediction: reality seems different, for example with mustelids. A close look at polymorphisms may hint at why this may be, but clearly more work would need to be done to confirm this.

As the susceptibility to SARS-CoV-2 of at least some animal groups does not correlate with that suggested by the ACE2 sequence alignments and classifications of others [39,41], three further proteins were used for similar analysis, to ascertain if any data obtained will shed further light on virus susceptibility. If significant correlations were found, this would inform future research on these proteins, such as more detailed structural analysis (not carried out here).

### 3.2. TMPRSS2

As well as a direct interaction between ACE2 and the spike proteins of SARS-CoV-2, as outlined above, there is a need for the involvement of proteins which undertake cleavage of some of the proteins involved in viral cell entry. One of these proteins is TMPRSS2 [13] and many characteristics of this protein are pertinent here, as listed in Table 2.

**Figure 3:**
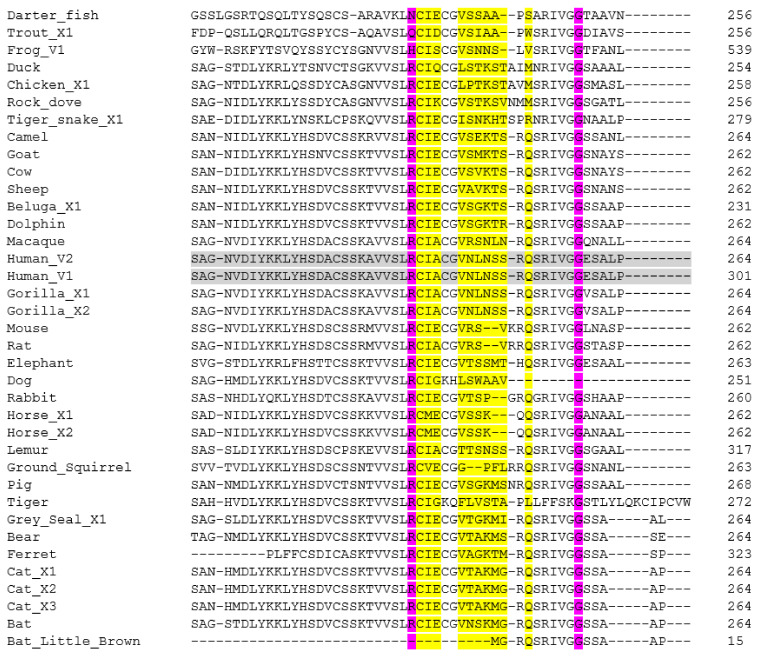
Amino acid sequence alignment of TMPRSS2 polypeptides from a range of animal species: a short section is shown here, whilst the full sequence alignments are shown in Supplementary Figure S2. Accession Numbers of the polypeptide sequences used are given in Table 1. Alignments were carried out using Clustal Omega [45]. Human sequence is highlighted in grey to aid comparison to other sequences. Polymorphisms reported by Hou et al. [20] are in purple. Amino acids involved in the TMPRSS2/SARS-CoV-2 interactions are highlighted in yellow [49].

Hou et al. [20] highlighted D435 as being important for TMPRSS2 activity. This amino acid is completely conserved across all the animal species looked at here (Figure 3—highlighted in blue). They point out a D435Y polymorphism, but clearly this is not evident in the animal sequences. They also picked out five other significant polymorphisms (V160M, G181R, R240C, G259S, P335L, G432A). V160M (human allele frequency 2.49e^−1^) is particularly interesting, as it is indicative of an increased affinity for the S proteins of SARS-CoV-2 [61]. In the animals it is totally conserved as a V, except in fish, where it is T or S, not M. G181 is conserved except in some animals (e.g., macaque and elephant) where it is A but this could be considered a conserved change. R240 is conserved except for in fish and frog, but none exhibit a C. G259 is totally conserved, except in dog. Here, the sequence is quite different, compared to all other animals, and if the same amino acid is counted along the sequence the G becomes a V. P335 is conserved except in fish and dog. Proline (P) is often thought to be an important amino acid for polypeptide topology because of its planar nature, and in dog has become a T. G432 and D435 are conserved. It is therefore tempting to suggest that the differences seen in dog may in some way account for its lack of infection severity and viral transmission.

Senapati et al. [49] report a list of 28 amino acids in TMPRSS2 (Table 2) which are in Supplementary Figure S2 highlighted in yellow (except F195 and F251 which were not in the human sequences used here). It should be noted that the amino acid numbers used from Senapati et al. [49] are from the variant 1 of the human sequence: variant 1 has a 37 amino acid extension at the N-terminal end. Looking at the consensus at these points in the sequences does show some interesting points. Some areas are well conserved, such as 190-192 (YGPN), especially in mammals, except for rodents, horse and bear. There is some variability also in non-mammal groups. However, other regions, such as R277-Q290 (RCIACGVNLNSS-RQ) are worth highlighting. This region is only totally conserved in humans and gorillas. However, even other primates such as macaques and lemurs are different. Some significant species differences are rodents where the sequence is RCIECGVRS--VKRQSR, but especially dog, where it is RCIGKHLSWAAV(−15)SW, but with a large gap alignment between the V and S. It is therefore, again, tempting to speculate that such differences may be significant in the way that the virus interacts with the cells of such hosts, but of course there may be other proteins involved, rather than TMPRSS2. Having said that, putting the human sequence (RCIACGVNLNSSRQ) through a BlastP search reveals no other proteins of significance, other than TMPRSS2.

**Figure 4:**
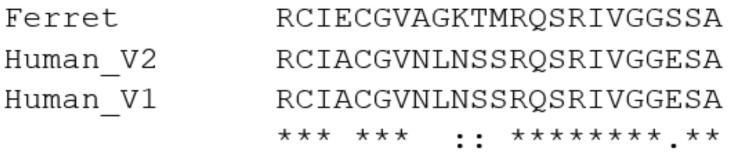
Alignment of human and ferret TMPRSS2 sequences, including the R277-Q290 region. (where * indicates consensus, : indicates conserved change, . is less conserved and a gap in analysis indicates no conservation, as deemed from amino acid characteristics).

One animal group worth focusing in are the mustelids. Here, ferrets were used as an example. Looking closely at the R277-Q290 sequence shows that ferret differs in many aspects in this region, as seen by this Clustal Omega alignment (Figure 4):

Of course, as it is known that mustelids are susceptible to SARS-CoV-2, can transmit the virus within populations, and even transmit it back to humans [32], the significance of these differences is hard to reconcile. It is known that the SARS-CoV-2 virus has mutated in mink [21], so any amino acid differences in the spike proteins also need to be taken into account in any future structural analysis. Even so, this simple analysis does not indicate why mustelids should be so susceptible, a conclusion supported by the data of others [39,41].

Additionally, of interest here are the polymorphisms highlighted by Senapati et al. [49]. They suggest that three lead to a decrease in SARS-Cov-2 affinity to TMPRSS2: G8V, R255S, S441G. Two polymorphisms are thought to increase the affinity, and this would be of particular concern, considering the human disease is so prevalent. V160, as mentioned above, is well conserved, whereas A28 is very variable (A28T being the polymorphism reported). A28 is conserved in gorilla, macaque, bears and birds. It is also conserved in ferret. Frog has the T variant, but is also very variable across the rest of that region, suggesting that this match of polymorphism at position 28 is not significant. gnomAD also lists a polymorphism with a frequency of 7.31e^−3^ (T112I) but the significance of this to SARS-Cov-2 susceptibility is not known. Therefore, although several species have the same polymorphisms as humans, there are no amino acid changes that would suggest increased affinity across the animals which have been used here. 

It should also be noted that although not considered here, Senapati et al. [49] also list a range of polymorphisms which will alter the expression levels of TMPRSS2, which may be crucially important for both TMPRSS2 activity and for SARS-CoV-2 interactions. Future work would need to assess any correlation of such base changes in animal species which may indicate whether predicting susceptibility is possible. 

Eighteen polymorphisms of interest, as seen in the gnomAD data and matching functional amino acids highlighted by others [49], are listed in Table 2. They are all rare, and the significance to SARS-CoV-2 susceptibility is not known, so no conclusions can be drawn here, but if any are found to increase or decrease viral affinity in the future their presence in animal sequences may need to be revisited. 

In summary, for this protein, there are no human polymorphisms which appear to give an indication of altered susceptibility to SARS-CoV-2 across the animal kingdom, although there are several polymorphisms listed, some which increase affinity for the protein to the virus in humans, and others which decrease it. However, a look across the alignments, and in particular focusing on those amino acids reported to be involved in the TMPRSS2/SARS-CoV-2 interaction, does show that the sequence of this protein in some animals is extremely similar to that of humans, such as gorillas. In some animals which have been found to be susceptible to the SARS-CoV-2 virus, such as dog [28], there are differences in their TMPRSS2 sequence, suggesting that further investigations based on this protein may be worthwhile so that the role of this protein in the viral/host interaction may be more fully understood.

### 3.3. Neuropilin-1

**Figure 5:**
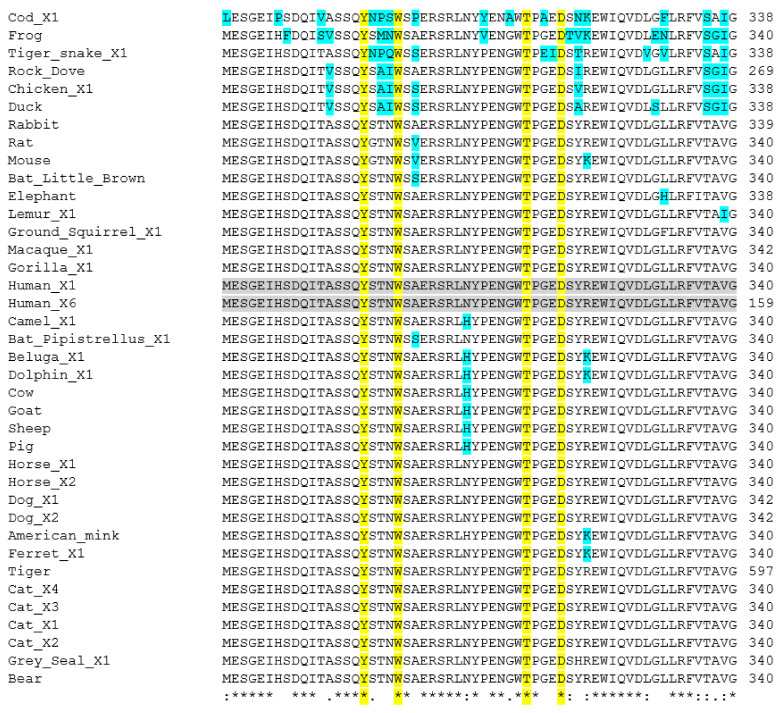
Amino acid sequence alignment of neuropilin-1 polypeptides from a range of animal species: a short section is shown here, whilst the full sequence alignments are shown in Supplementary Figure S3. Accession Numbers of the polypeptide sequences used are given in Table 1. Alignments were carried out using Clustal Omega [45]. The last row shown is the consensus analysis given by the algorithm. Human sequence (variant 2) is highlighted in grey to aid comparison to other sequences. Important amino acids used for interaction with the SARS-CoV-2 viral proteins [14] are highlighted in yellow. Amino acid differences from the human sequence in this region are highlighted in blue.

With the lack of correlation with susceptibility to the SARS-CoV-2 virus with sequences differences in either ACE2 or TMPRSS2 across animal species, it is worthwhile looking at other key proteins involved in the virus/host interaction. In this vein, a recent paper by Daly et al. [14] showed that the protein neuropilin-1 had a direct interaction with the SARS-CoV-2 spike proteins. Furthermore, they highlighted eight amino acids as being important (Table 2). In alignments of the 38 animal species here (Figure 5 and Supplementary Figure S3) showed that all eight amino acids are completely conserved, indicating that this analysis would not be useful for predicting the susceptibility of animals to the virus. gnomAD lists a rare polymorphism at one of these positions (T249S), but there is no suggestion of this being significant. There are other more common polymorphisms listed on gnomAD. V179A has an allele frequency of 1e^+0^. In the sequence alignments all animal species have A at this position: *Homo sapiens* is the only one with V. There are no reports of this polymorphism altering the SARS-CoV-2 interaction, but it may be a polymorphism which is worth revisiting in the future. Other polymorphisms include R563Q with a frequency of 1.7e^−1^, but this position is conserved as an R in all animals. V733I (frequency 1.2e^−1^) is a V in all animals, except cod, which has an I, although analysis using ACE2 suggests that fish are not susceptible to SARS-CoV-2, so this amino acid occurrence is almost certainly irrelevant. There are also polymorphisms in non-canonical transcripts, such as D592N (frequency 3.1e^−1^)—not shown here—but the relevance and importance of these is not presently known.

In summary, there is no evidence at the moment that amino acids in this protein that are involved the SARS-CoV-2 interaction can give any indication of assessing if animals can become infected with the virus. There are some polymorphisms, and although there is no evidence of these being significant for the role of this protein in the disease, they may be useful in the future as the impact of polymorphisms is assessed in the light of the correlation of ethnicity and the susceptibility of humans to SARS-CoV-2.

### 3.4. Furin

**Figure 6:**
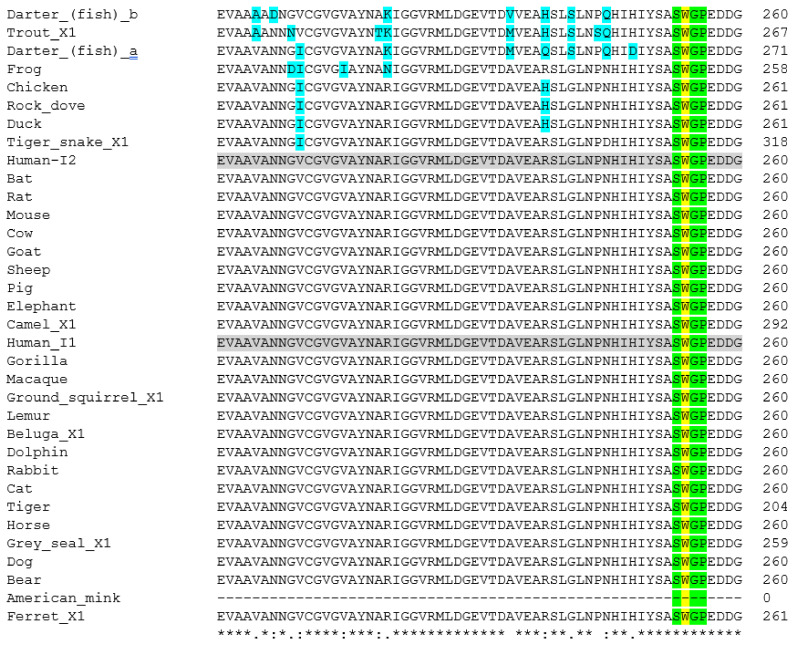
Amino acid sequence alignment of a region of the furin polypeptides from a range of animal species: a short section is shown here, whilst the full sequence alignments are shown in Supplementary Figure S4. Accession Numbers of the polypeptide sequences used are given in Table 1. Alignments were carried out using Clustal Omega [45]. The last row shown is the consensus analysis given by the algorithm. Human sequences are highlighted in grey to aid comparison to other sequences. Important amino acids used for catalytic site of the enzyme, as discussed [62] are highlighted in yellow, with the S253-P255 region in green. Differences in sequences from the human sequence are shown in blue.

With analysis of neuropilin-1 sequences failing to be useful for assessing SARS-CoV-2 susceptibility, a final protein investigated here is furin. Furin has been highlighted as an important enzyme which is involved in the entry of SARS-CoV-2 into cells [15,16]. Here, the sequences of a wide range of animals, as listed in Table 1, were aligned using Clustal Omega [45]. Not all the sequences were available or of a robust quality: the dog sequence was labelled as being of low quality, whilst the Neovison sequence was only partial. The data is presented in Figure 6, and Supplementary Figure S4.

Dahms et al. [62] highlight several amino acids as being important (Table 2). H194, S368 and N295 are part of the catalytic site, in what is referred to as the oxyanion hole. They also suggest that the β-strand at S253–G255 and P356 are important. This, along with W245, is important for substrate binding. They also highlight sodium binding site at T309 and S316.

As would be expected, all the amino acids highlighted by Dahms et al. [62] for being important for catalysis are totally conserved across all animal species looked at here. In fact, as can be seen in Figure 6, there are large regions of sequence which are totally conserved across all mammals. Although the American mink sequence is only partial, the ferret sequence is representative of the mustelids and it would be reasonable to assume that the mink sequence is not significantly different at the highlighted points. There are differences seen in the reptile, fish and amphibian sequences, and particularly in the bird sequence shown in Figure 6. Of course, it needs to be noted that this is only a short section of amino acids represented here, and there are significant sequence differences seen in other areas. For example, the pig sequence appears to have a large inserted region at residue 518, but the significance is not known.

Interrogating the gnomAD data revealed a rare polymorphism, T309I, but none at the other highlighted sites. However, as this amino acid is completely conserved across all the animals here, it is not likely to be useful as a marker of significance for looking at SARS-CoV-2 susceptibility. There are no other polymorphisms of furin for which the significance of viral entry into cells has been reported, although there are polymorphisms in the cleavage site of the viral proteins themselves [18].

In summary, furin appears, at least at the current understanding, to be a poor candidate protein for assessing an animal’s susceptibility to SARS-CoV-2.

### 3.5. Phylogenetic Analysis of ACE2 and TMPRSS2 Sequences

**Figure 7:**
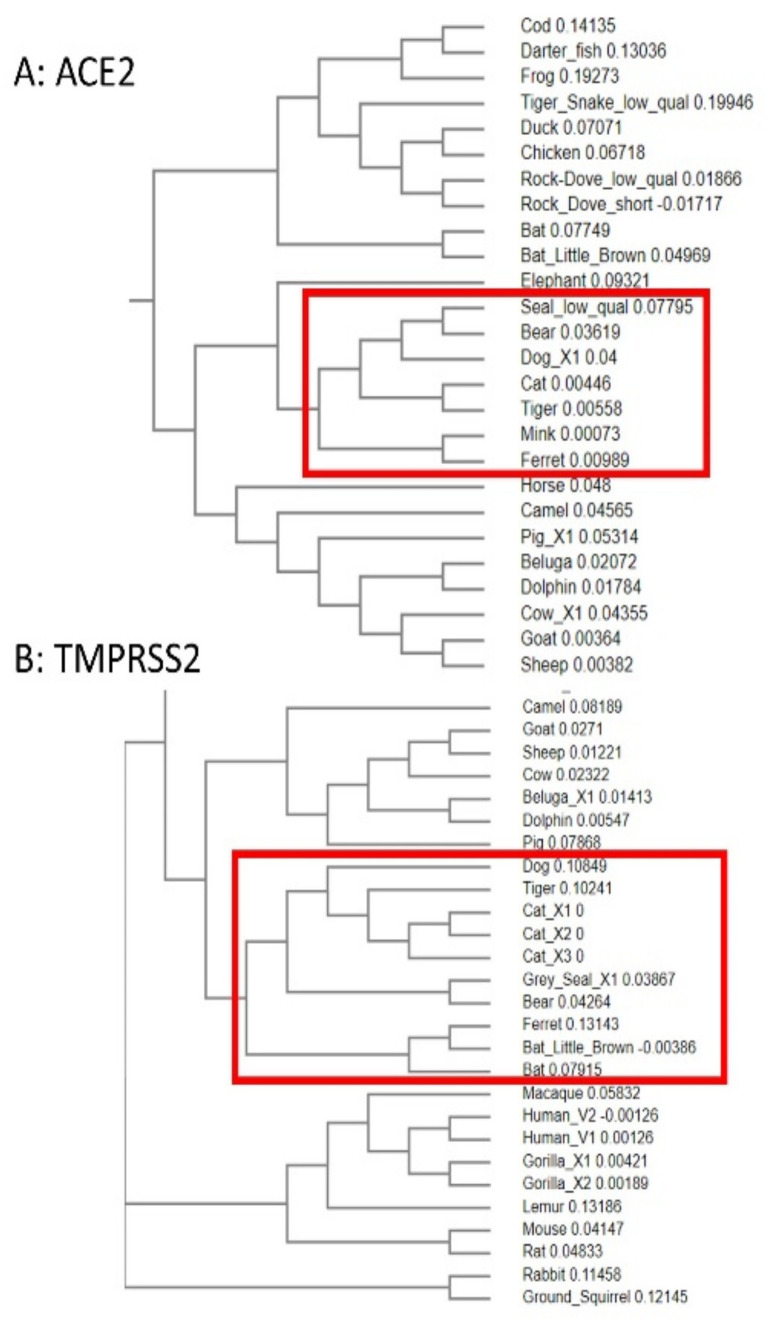
Phylogenetic analysis of the animals using the alignment sequences. Sequences as listed in Table 1 were analysed by Clustal Omega [45] and then relevant sections of the phylogenetic analysis shown here. A: analysis of the ACE2 sequences; B: analysis of the TMPRSS2 sequences.

To further the sequence analysis, the ACE2 and TMPRSS2 sequences were used to create phylogenetic trees (Figure 7). Interestingly, using ACE2, ferret, mink, tiger, cat and dog all appeared in a related group, and they are the species reported in the literature as having been tested positive for the SARS-CoV-2 virus [26,27,28,29,30]. Additionally, in this group were bear and seal, although the seal sequence was of low quality. When the analysis was repeated for the TMPRSS2 sequences, again ferret, cat, dog and tiger were grouped together, and again these were accompanied by bear and seal, along with bat. However, such analysis ignores the subtleties of the input of individual amino acids in the spike protein/host protein binding. Looking across the highlighted amino acids deemed to be important, there are 5 differences in the bear and 4 in the seal sequence (which is low quality and incomplete). Furthermore, bear and seal and bat all contain the ACE2 H34Y substitution which lowers viral susceptibility [68]. Therefore, such species may not be as susceptible as the phylogenic evidence seems to suggest.

However, with animals which are known to be susceptible [26,27,28,29,30] to the virus grouping together in this way, it might be worth in the future using the analysis with further animal species. Of particular interest may be some marine mammals (such as beluga whale (*Delphinapterus leucas*), dolphin (*Tursiops truncates*), killer whale (*Orcinus orca*), seal species (*Pinnepedia*), Atlantic Walrus (*Odobenus rosmarus*), some of which were listed in the highly susceptible group when ACE2 alignments were carried out by others [41]. It may also add further evidence that certain species groups may not be susceptible to the virus, such as birds and reptiles. As with the simple sequence alignments, phylogenetic analysis may inform a future focus on certain proteins and animal species which may be worthy of more protein structural studies.

## 4. Discussion

COVID-19, caused by the virus SARS-Cov-2 which interacts with the host proteins [70,71], is the latest in a series of related diseases, following SARS (SARS-CoV-1) in 2002–3 and MERS (MERS-CoV) in 2012 [72]. As it is a disease caused by a zoonotic virus there is the potential for the virus to transmit to animal populations [73]: the subject of a recent review [74].

There are several proteins known to be involved in the recognition, interaction and entry of the virus into host cells [49,50,51,52,53,54,55,56,57,58,59,60,61,62,63,64,65]. ACE2 is one of the most important [11], but others such as TMPRSS2 and furin are also key [15], with neuropilin-1 being recently highlighted [14].

Although the key amino acids involved in viral interactions in such proteins can be identified, mainly using elements of structural analysis, the situation may be muddied by the existence of mutations in the viral proteins [18,75]. The protein interactions are determined by the characteristics of individual amino acids, and if these are altered then the binding may be enhanced or reduced. A detailed analysis of the binding of the viral proteins to bat ACE2 makes a good example [76], where mutational analysis was used to show that Y41 and E42 of the ACE2 protein were key amino acids. If these are not present in other species, or there is a conserved change, this may influence the protein-protein interactions. However, it needs to be stressed that the genetics of coronaviruses are also crucial to understand, and are not static, so the exact nature of the viral proteins is important too. In an analysis of porcine deltacoronavirus (PDCoV) it was found that there were four distinct phylogenetic lineages [77], for example. Importantly, there were five amino acids which were important for adaptive evolution, highlighting that the viruses are able to change to facilitate their host interactions. Therefore, as the SARS-CoV-2 virus alters and adapts [78,79,80] this will have a major influence on whether it can interact with cells from different animal species, and will of course influence any use of the analysis carried out here and by others [39,41].

Here, although perhaps a relatively simple view of cell/virus interactions, simple and quick alignments of the human sequences of four proteins was undertaken (Figures S1–S4). Others have reported such alignments for the ACE2 protein from a range of animals, including mammals, fish, birds, reptiles and amphibians [39,41]. Here, this has been extended to TMPRSS2, neuropilin-1 and furin for a similar range of animals. However, despite differences seen in ACE2 and TMPRSS2 it is hard to correlate such data with a known susceptibility seen in animals in reality. For example, mustelids are not predicted to be highly susceptible, but there are concerns about the prevalence of the virus at mink farms [81], and significant culling of animals has taken place [82].

Four proteins were used here, and sequence alignment on two of them, neuropilin-1 and furin, gave little indication that they would be useful for virus susceptibility prediction. Furthermore, other proteins have been mooted to be important also, such as the tight junction protein PALS1 [63]. The endosomal/lysosomal cysteine proteases cathepsin B, L (CTSB, CTSL) may also be involved [64]. Clearly a full complement of the interacting proteins and any redundancy in their functionality will need to be known before too much significance can be attributed to the alignment of the sequences of one or two proteins.

Additionally, not considered in this simple analysis are other potential confounding factors such as the intracellular viral progression, age, health and immune status of the animals which may become hosts, environmental factors (such as the animal housing conditions in mink farms which may be ideal locations for the transmission, and even mutation, of the virus) or behavioral factors (such as grooming behavior in cats, who may thereby ingest virus from their fur). Even in humans the susceptibility of the virus is different between men and women [83], and ethnicity appears to be important to disease severity [84]. Therefore, there are clearly factors beyond the ones used here which are important. Virus susceptibility and transmission is a combination of biological and environmental/social factors. It is suggested here that if the understanding of these subtle differences across the human population, perhaps through an enhanced understanding of polymorphisms, can be translated across to animal species, then in the future animal susceptibility to coronaviruses may be easier to predict.

The prediction of animal susceptibility is important. It appears from all the sequence analysis that the great apes, such as gorillas, will be susceptible [54], although there is only one case reported to date, i.e., gorillas at San Diego zoo [85]. Certainly calls for caution are justified, [86,87], with geographical regions being isolated and personal protective equipment (PPE) being recommended, especially given our extremely limited current understanding of how animals within wild populations rather than in zoos may differentially respond to infection, as well as the difference in availability of high quality veterinary care. At the other end of the spectrum, the analysis of these sequences suggests that greatest differences from humans can be seen in birds, reptiles, amphibians and fish. This is no great surprise, as being more evolutionarily distanced the sequences would be expected to be different. Some conserved regions can be seen, but importantly the areas of the ACE2 and TMPRSS2 proteins which are used for SARS-CoV-2 interactions are variant in these animals, giving a potential reassurance that they are unlikely to be susceptible to SARS-CoV-2, and therefore, as far as can be assessed, further precautions are not needed when being in close proximity with, and managing, such animals.

One of the biggest issues is the need to determine the risk to, and from, companion animals, such as those kept in the home. Cats are known to test positive for SARS-CoV-2, and transmit it within their populations [25,26,27]. Dogs too, have been reported to be positive for SARS-CoV-2 [28]. Although other species such as hamsters can be infected, there are no reports of this happening in a domestic setting. It is of course a confused picture as some domestic animals, such as cats [88] and dogs [89] have known coronaviruses which can give COVID-19-like symptoms. 

Several marine mammals, including beluga whale (*Delphinapterus leucas*), dolphin (*Tursiops truncates*) and killer whale (*Orcinus orca*), are listed as highly susceptible by Damas et al. [41] using the ACE2 sequences. In a manner similar to many animals, these mammals are susceptible to other coronaviruses [90], and therefore there is concern about them being infected with SARS-CoV-2 [91]. With it being known that SARS-CoV-2 can exist in human faeces [92] it has been determined that the virus can survive transiently in wastewater [93] and seawater [94] although it has been disputed that this a significant route of virus transmission in the environment [95]. Although this might not sound too alarming, there is still the potential for marine mammals to be in contact with the virus near sewage outlets. Recently, the issue of the susceptibility of marine mammals to SARS-CoV-2 has been further explored [96]. Here, the ACE2 receptor was once again used as a predictor of virus susceptibility. Although some species were predicted to be of low susceptibility, such as the California sea lion* (Zalophus californianus*) and West Indian manatee (*Trichechus manatus*), other species were suggested to be of high or very high susceptibility. 18 out of the 21 *Cetacea* species looked at were classified as such, and this included the bottlenose dolphin (*Tursiops truncates*) and beluga whale (*Delphinapterus leucas*). Interestingly, the Sowerby’s beaked whale* (Mesoplodon bidens*) contained unique mutations (S19P and Q24K) and were said to be of low susceptibility. Furthermore, eight out of nine seal species (*Pinnepedia*) along with the Atlantic Walrus (*Odobenus rosmarus*) were all classed as highly susceptible. The ramifications of this paper were further discussed in a letter to the journal, where it was argued that water contamination may be less of a risk than *originally *stated [95]. However, in the light of the failure of previous ACE2 analysis to accurately predict susceptibility (such as mink) caution should be exercised before accepting such arguments, and the precautionary principle should be applied in the absence of further clear evidence, such as observational data, which validates these predictions of a lack of susceptibility of these specified marine mammals.

Lastly, SARS-Cov-2 was thought to have its origins in bats, and therefore this group of animals is important for consideration. Here, only two species were used: *Pipistrellus kuhlii* and *Myotis lucifugus*. There was no strong evidence that they would be particularly susceptible, whilst others put many bats in the very low category [41]. Despite this, there remains concern that bats may become infected with SARS-CoV-2 [97] and there are also concerns about how the disease is communicated in case there are any welfare issues for bat populations which become seen as a threat [98]. These concerns are supported by welfare issues related to other species, such as reports of pets being abandoned for fear that they could carry the virus (although it does not appear that same fear has decreased the market for pangolin scales—human understandings and behaviors in this respect are of course complex). Moving forwards, SARS-CoV-2 may throw a spotlight on welfare issues such as the conditions prevailing in mink farms (or other intensive farming settings). Even if a vaccine for animal reservoirs (such as mink) becomes available, questions will inevitably be asked about the conditions which led to these populations becoming so easily a reservoir for the virus, and mutation, (which could threaten humans and animals alike). Evidence in this respect will be important as a consideration for the prevention, and handling, of any future pandemics relating to diseases caused by zoonotic viruses.

## 5. Final Thoughts and Future Perspectives

The amino acid sequences of the proteins of focus here are available for a wide range of animals, from amphibians to non-human primates. However, there are several animals which are known to be in close contact with humans for which this information is not currently available. The Indian elephant (*Elephas maximus indicus*) which is used widely across the Indian subcontinent, lions (*Panthera leo*) which may be held in zoos and are known to be able to become infected with SARS-CoV-2 [29], and sloths (*Folivora*) which may be in rescue centers are just some examples. Therefore, during a SARS-CoV-2 pandemic the welfare of such animals may be of particular concern as there may not be an easy way to determine if they are safe. No doubt future genome sequencing projects will aid to counter this problem.

It should also be noted that many of the sequences used here, and that will be used by others, are predicted sequences or not complete. The dog (*Canis lupus familiaris*) furin sequence here, for example, is annotated as being of low quality, as is the ACE2 sequence from *Halichoerus grypus*. Again, future sequencing, structural and functional protein work will address such issues. Having said that, there is an indication of the susceptibility of animals which can be gleaned from using the protein sequences, as used by others [39,41] and here. Proteins which can be identified as being in direct interaction with the SARS-CoV-2 spike proteins are able to give some useful clues, such as ACE2 and TMPRSS2. However, the amino acid sequences of neuropilin-1 and furin from different animals is unlikely to be fruitful as a way to determine SARS-CoV-2 susceptibility. 

Polymorphisms in the sequences will exist in the animal population too [99], and therefore just because a sequence shown here does not indicate an increased or decreased susceptibility to SARS-Cov-2 it does not mean that all individuals of that species can be assumed to be the same. It is interesting that animals such as cats and dogs can be infected with SARS-CoV-2 but it does not appear to be rampant across their populations. Even though studies have shown that cats can transmit the virus between individuals [25] it does not seem to be spreading in cat populations (although there is no systematic surveillance), even though cats are often left to freely roam their neighborhoods. It may be the case that there are individual differences in virus susceptibility of individual animals (for example related to their health status and living conditions). To determine this would require an extensive study of animal gene sequences and correlation to known SARS-CoV-2 positive and negative animals, combined with study of relevant factors in the individual animal’s lived environment.

In the future, as more is known about the mechanism of the entry of SARS-CoV-2 into human and animal cells, the differences seen in the polypeptide sequences from a range of animals may be useful to predict if animals may be susceptible. Furthermore, as polymorphisms in such proteins come to light, and some of them increase or decrease the likelihood of viral entry, this information may be able to be translated across to an animal species.

Outside the scope of this paper, but what has not been considered here is the expression levels of these proteins in different animal cells. Expression levels of ACE2 in human airways has been studied [100], as has the expression of this proteins in a range of tissues [101], it being found prevalent in the kidney, gallbladder, heart, male reproductive cells, eye, and vasculature. The expression profiles of other important proteins such as TMPRSS2 has also been investigated [64]. It has been suggested that the relatively low susceptibility of dogs and pigs may be due to ACE2 expression levels in these animals [46]. As more is known in a range of animals’ patterns of protein expression may give further clues to which animals may become infected or transmit the virus.

COVID-19, caused by SARS-CoV-2, is the latest in a series of coronavirus epidemics, which included SARS-Cov-1 and MERS-CoV [72]. Therefore, the more that is known about the subtle differences in proteins such as those highlighted here, the more likely it is that we can predict the susceptibility of animals to future coronaviruses. This will be particularly important for animals in close contact with humans, such as companion and farm animals, and wild animals which come into contact with humans. Such work can relatively easily give a guide as to whether some animals are likely to be susceptible, such as non-human primates [54], whilst others may be unlikely to be infected, such as fish [41]. Even though some animals have been known to be infected with SARS-CoV-2, and others are predicted to be susceptible, it has been suggested, at least to date, unlikely to be a serious problem [102]. Having said that, very recently another zoo animal, this time a snow leopard, has been reported to be positive for SARS-CoV-2 [103]. More worrying is the report that the first wild animal has been tested positive [104], in this case a mustelid. It has been suggested that it is a good time to embrace the One Health agenda [105]. SARS-CoV-2 has unequivocally demonstrated the full extent of integration of animals and humans, both have suffered health and welfare impacts as a consequence. Given the events surrounding mutated virus emerging in mink farms, it is essential that the disease in both animals and humans are considered in tandem, for the health and welfare of both. 

To conclude, some of the pertinent points about the four proteins which have been the focus of this paper are summarized in Table 3, whilst some of the salient points raised about particular animals have been summarized in Table 4.

The challenge for the future is to determine whether new coronaviruses are likely to cause disease in animals which interact with humans, and whether a rapid estimation of this can be given to quickly prevent the spread and re-transmission of mutated virus back to humans and/or other animals. The identification of the host’s interacting proteins, and analysis of their similarity to humans, is a relatively quick and easy mechanism to derive information. Certainly, it would not give a robust prediction of virus susceptibility, based on the evidence about this so far on the SARS-CoV-2 virus, as reported in this paper. However, especially when augmented by further analysis, such protein structure determination, it may be a useful input when combined with other forms of data, including clinical and observational studies. What we learn now from SARS-CoV-2 about the usefulness of types of analysis as part of any predictive model is important for future coronavirus outbreaks.

## 6. Conclusions

In summary, looking at the sequence alignments of proteins such as ACE2 and TMPRSS2 can give only an estimation of whether animal groups may be susceptible to SARS-CoV-2. However, analysis of proteins such as furin and neuropilin-1 may be of very limited use. Some animals appear by this analysis to be highly susceptible, such as great apes, whilst others are predicted to be safe from the viral infection, such as fish, birds, reptiles and amphibians [41]. There are several animal groups in between these extreme predictions for which susceptibility at the moment cannot be accurately predicted from this analysis, such as the mustelids, which of course throws into question how useful such analysis is. However, further genomic analysis, in combination with data on animals which have been shown to be infected, either in the laboratory or environment, may be useful to inform further research in this area. Clearly more work needs to be carried out to understand why mustelids are susceptible to infection, which is not elucidated by analysis of the ACE2 sequences, for example. Combined with an understanding of environmental factors, a more in-depth molecular analysis may help elucidate this in the future.

## Figures and Tables

**Table 1:** Animal species and Accession Numbers of the sequences used for the amino acid alignments shown in the Figures. Data were obtained from The National Center for Biotechnology Information (NCBI). X#, I# or V# indicates the isoforms chosen. N/A: not available from NCBI; * indicates that for the furin sequence this was from a *Bos indicus × Bos taurus* (hybrid cattle).

Common Name	Animal	ACE2	TMPRSS2	Neuropilin-1	Furin
Human	*Homo sapiens*	BAB40370.1	V1: NM_001135099.1	X1: NM_003873.7	I1: NP_001276753.1
			V2: NM_005656.4	X6: XM_017016866.2	I2: NP_001369551.1
Gorilla	*Gorilla gorilla*	XP_018874749.1	X1: XM_019017901.2	X1: XM_004049248.3	XP_018866183.1
			X2: XM_004062839.3		
Macaque	*Macaca nemestrina*	XP_024647450.1	XM_011725990.2	X1: XM_011738246.1	XP_011750505.1
Lemur (Grey mouse)	*Microcebus murinus*	XP_020140826.1	XM_012749836.2	X1: XP_020139780.1	XP_012618739.1
Camel	*Camelus ferus*	XP_006194263.1	XM_032461838.1	X1: XP_032329703.1	X1: XP_032325184.1
Horse	*Equus caballus*	XP_001490241.1	X1: XM_005606160.3	X1: XM_023632110.1	XP_023505390.1
			X2: XM_014736385.2	X2: XM_001915381.4	
African elephant	*Loxodonta africana*	XP_023410960.1	XM_023559111.1	XM_023539786.1	XM_003413876.3
Goat	*Capra hircus*	AHI85757.1	X1: XM_013966670.2	X1: XP_017912559.1	XP_017921808.1
Cow	*Bos taurus* *	X1: XP_024843618.1	X1: XM_015473655.2	X1: XP_024856535.1	XP_027377940.1
Sheep	* Ovis aries *	XP_011961657.1	XM_027960704.1	X1: XP_027832377.1	CAJ29337.1
Pig	* Sus scrofa *	X1: XP_020935033.1	NM_001386131.1	XP_020920629.1	X1: XP_020954578.1
Black rat	*Rattus rattus*	XP_032746145.1	XM_032900351.1	XP_032743292.1	XP_032749231.1
Mouse	*Mus musculus*	EDL40761.1	NM_015775.2	AAH60129.1	CAA37988.1
Rabbit	*Oryctolagus cuniculus*	QHX39726.1	NM_001386128.1	XP_008272962.1	XP_002721548.2
Cat (domestic)	*Felis catus*	AAX59005.1	X1: XM_023238709.1	X1: XP_006933414.1	XP_023110662.1
			X2: XM_023238710.1	X2: XP_003988264.1	
			X3: XM_023238711.1	X3: XP_003988265.1	
				X4: XP_006933415.1	
Bear (grizzly)	* Ursus arctos horribilis *	XP_026333866.1	X1: XM_026499970.1	X1: XM_026483469.1	XM_026510505.1
Tiger (Siberian)	*Panthera tigris altaica*	XP_007090142.1	XM_015541202.1	XP_007092922.2	XP_015391216.1
Dog (domestic)	*Canis lupus familiaris*	X1: XP_013966804.1	XM_022413273.1	X1: XP_005617003.1	XP_022272656.1
				X2: XP_535142.3	
American mink	*Neovison vison*	CCP86723.1	N/A	CCP78142.1	CCP82836.1
Ferret	*Mustela putorius furo*	BAE53380.1	XM_013061267.1	X1: XP_004774343.2	X1: XP_004763758.1
Ground squirrel	*Ictidomys tridecemlineatus*	XP_005316051.3	XM_021725256.1	X1: XM_005330824.1	X1: XP_021578591.1
Kuhl’s pipistrelle bat	*Pipistrellus kuhlii*	XP_036295422.1	XM_036446834.1	X1: XP_036309963.1	XP_036271438.1
Little brown bat	*Myotis lucifugus*	X1: XP_023609438.1	XM_006104440.3	XP_023607944.1	N/A
Beluga whale	*Delphinapterus leucas*	XP_022418360.1	X1: XM_022552959.1	X1: XP_030617644.1	X1: XP_022419719.1
Grey seal	*Halichoerus grypus*	XP_035963182.1	X1: XM_036108025.1	X1: XP_035974500.1	X1: XP_035951550.1
Bottlenosed dolphin	*Tursiops truncatus*	XP_019781177.2	XM_033856384.1	X1: XP_033708297.1	XP_019801399.1
Cod (fish)	*Gadus morhua*	XP_030232530.1	N/A	X1: XP_030204747.1	N/A
Arkansas darter (fish)	*Etheostoma cragini*	XP_034732342.1	XM_034889577.1	N/A	a: XP_034734947.1
					b: XP_034734346.1
Rainbow trout (fish)	* Oncorhynchus mykiss *	N/A	X1: XM_021618133.2	N/A	X1: XP_021428901.2
Chicken (domestic)	*Gallus gallus domesticus*	QEQ50331.1	X1: XM_416737.6	X1: XP_015136776.1	NP_990046.1
Duck	* Anas platyrhynchos *	XP_012949915.2	X1: XM_027448371.1	X1: XP_027306331.1	X1: XP_027321808.1
Rock dove	*Columba livia*	XP_021154486.1	XM_021282586.1	PKK32102.1	XP_021155000.1
		PKK30539.1			
Tiger snake	*Notechis scutatus*	XP_026530754.1	X1: XM_026673992.1	X1: XP_026528008.1	X1: XP_026540426.1
African clawed frog	*Xenopus laevis*	XP_018104311.1	V1: NM_001087958.1	NP_001081380.1	AAW83022.1
Sloth	* Folivora *	N/A	N/A	N/A	N/A
African lion	* Panthera leo *	N/A	N/A	N/A	N/A
Indian elephant	*Elephas maximus indicus*	N/A	N/A	N/A	N/A

**Table 2:** Amino acids deemed to be important in selected proteins and polymorphisms which have been found to be significant. Glycosylated residues highlighted in red. Note: Senapati et al. [49] is a preprint at the time of writing this manuscript.

Protein	Reference(s)/Comments	List of Amino Acids Thought to Be Important for Viral/Host Interactions	Polymorphisms of Interest	Comments and Refs for PMs
ACE2	[50]	S19, Q24, T27, F28, D30, K31, H34, E35, E37, D38, Y41, Q42, L45, L79, M82, Y83, N330, K353, G354, D355, R357, R393	S19P, T27A, K31R, N33I, H34R, E35K, E37K, D38V, Y50F, N51S, M62V, K68E, F72V, Y83H, E329G, G352V, D355N, Q388L, P389H, D509Y	Decreased S protein affinity: [49] and refs within
	[51]	Q24, K31, H34, E35, D38, Y41, Q42, N53, L79, M82, Y83, N90, N322, Q325, E329, N330, K353, R652, R710	I21V, E23K, K26R, T27A, N64K, T92I, Q102P, D206G, G211R, R219C, G326E, K341R, H378R, V447F, I468V, A501T, R559S	Increased S protein affinity: [49] and refs within
	[52]	S19, Q24, T27, F28, K31, H34, E35, E37, D38, Y41, Q42, L45, L79, M82, Y83, E329, N330, K353, G354, D355, R357	E300Ter, A627V, N638S, L656Ter, S692P, N720D, L731I/F, E668L	Affinity not reported [49] and refs within
	[41]: glycosylation sites in red	S19, Q24, T27, F28, D30, K31, H34, E35, E37, D38, Y41, Q42, L45, N53, L79, M82, Y83, N90, N322, N330, K353, G354, D355, R357, R393	M82I, E329G, D355N, R652K, R710C, R710H: No other significant polymorphisms found	from gnomAD
	[39]: concentrated on residues 36–53 AEDLFYQSSLA SWNYNTN	Q24, F28, D30, K31, H34, E35, E37, D38, Y41, Q42, L79, M82, Y83, K353, G354, D355, R357	S19P (increases affinity), K26R (decreases affinity)	[53]
	[54] with input from [55]	Q24, D30, H34, E37, D38, Y41, Q42, M82, Y83, K353, D355, R357	S19P, I21V, E23K, K26R, T27A, N64K, T92I, Q102P, H378R	Increase [56]
	[49]	E23, Q24, K26, T27, D30, K31, H34, E35, E37, D38, Y41, Q42, K68, L79, M82, Y83, D206, G211, R219, K317, G326, E329, K341, G352, K353, D355, R357, P389, V447, I468, R559, D442, N437, T478, F486,	K31R, N33I, H34R, E35K, E37K, D38V, Y50F, N51S, M62V, K68E, F72V, Y83H, G326E, G352V, D355N, Q388L, D509Y	Decrease [56]
	[57]	353-KGDFR-357	E37K, G352V, D355N (38 others mentioned)	Decrease [58]
	[59]	R and K residues within 697–716 needed for cleavage	T27R, G326E	Increase [58]
			S19P, E329G	Decrease [60]
			K26R (decrease). Increasing affinity in order: I468V, R219C, K341R, D206G, G211R	[61]
TMPRSS2	[20]	D435	V160M, G181R, R240C, G259S, P335L, G432A, D435Y	[20]
	[49]	G190, N192, P191, F195, Y189, S234, K399, D396, N395, T324, A280, C278, R277, F251, I279, F394, Y232, N284, V283, Q290, L285, P325, N488, S287, S288, I489, N286, T393	G8V, R255S, S441G	Decreased S protein affinity: [49] and refs within
			V197M/V160M, A65T/A28T	Increased S protein affinity: [49] and refs within
			G190R, P191Q, Y189C, S234G, D396N, T324N, A280D, R277H, R277P, R277C, I279T, I279V, F394S, N284K, N284S, P325T, I489T, N286Y,	from gnomAD
Neuropilin-1	[14]	Y297, W301, T316, D320, S346, E348, T249, Y353	T249S (rare), V179A, R563Q, D592N (non-canonical), V733I	from gnomAD
			No polymorphisms matching other highlighted amino acids	
Furin	[62]	H194, W254, N295, S368 (b-strand at S253-P256), T309, S316	T309I (rare)	from gnomAD
			No polymorphisms matching other highlighted amino acids	

**Table 3:** A summary of pertinent points about the proteins analyzed.

Protein of Interest	Comments and Conclusions
ACE2	Key protein for viral interactions Several human polymorphisms identified which alter viral susceptibility, either causing an increase or decrease Sequence similarities between animals and human has been used to try to predict susceptibility of vertebrates No polymorphisms which alter viral susceptibility in humans found to correlate to susceptibility in animals to date Sequence variants do not correlate with reported SARS-CoV-2 susceptibility to infection in animals More work on mustelids would be interesting
TMPRSS2	Some differences in sequences across animal groups Several human polymorphisms identified which alter viral susceptibility Sequence variants do not correlate with reported susceptibility to SARS-CoV-2 infection in animals
Neuropilin-1	Little variation across animal species and with humans Limited or no use for predicting animal susceptibility to SARS-CoV-2
Furin	Little variation across animal species and with humans Limited or no use for predicting animal susceptibility to SARS-CoV-2

**Table 4:** A summary of the susceptibility predictions for a selected range of animals and how this correlates to real-life reporting to-date.

Animal Group	Susceptibility Predicted	Real-Life Outcomes Reported/Comments
Non-human primates (especially great apes)	Very high—sequences from several species very similar to humans	Some concern [54] Only one case reported: gorillas at San Diego zoo [85]
Marine mammals e.g., beluga whale (*Delphinapterus leucas*), dolphin (*Tursiops truncates*), killer whale (Orcinus orca), seal species (*Pinnepedia*), Atlantic Walrus (*Odobenus rosmarus*)	High	Some concerns, especially as sea water can be contaminated [94] No reports to-date of positive animals
Cats Including domestic *(Felis catus*) and big cats, e.g., Malayan tigers (*Panthera tigris jacksoni*), Amur tigers (*Panthera tigris altaica*) and African lions (*Panthera leo krugeri*)	Medium	Several reports of cats testing positive [25,27] Several big cats testing positive, including tigers, lions, snow leopards and a cougar [35] Some concerns that cats can transmit the virus [25]
Dogs Including domestic (*Canis lupus familiaris)*	Medium	Dogs found to be virus positive [28] Low expression of ACE2 in respiratory tract [44] No reports of dogs transmitting the virus
Mustelids Including mink (*Neovison vison*)	Low	Large numbers of animals tested positive Large number of animals culled Can transmit virus back to humans [21] Mutation of virus in mustelids First case of a wild animal being virus positive [104]
Marine mammals e.g., California sea lion (*Zalophus californianus*) and West Indian manatee (*Trichechus manatus*), Sowerby’s beaked whale *(Mesoplodon bidens*)	Low	Predicted using the ACE2 receptor [96] No reports of animals testing positive
Bats including fruit bat (*Rousettus aegytiacus*), Chinese rufous horseshoe bat (*Rhinolophus sinicus*), great roundleaf bat (*Hipposideros armiger*), big brown bat (*Eptesicus fuscus*)	Low/very low	37 chiropteran species predicted susceptibility as low or very low [44] No cases of bats being SARS-CoV-2 positive to date Experimentally fruit bats (*Rousettus aegytiacus*) have shown some infection [34,38] Focus on bats as being potential original source, but no conclusive evidence to date
Birds/reptiles/amphibians/fish	Very low	No cases of any of these groups being SARS-CoV-2 positive to date

## Data Availability

Data created is available in the Supplementary figures.

## Supplementary Materials

The following are available online at https://www.mdpi.com/2076-2615/11/3/797/s1, Figure S1: Amino acid sequence alignment of ACE2 polypeptides from a range of animal species, Figure S2: Amino acid sequence alignment of TMPRSS2 polypeptides from a range of animal species, Figure S3: Amino acid sequence alignment of neuropilin-1 polypeptides from a range of animal species, Figure S4: Amino acid sequence alignment of a region of the furin polypeptides from a range of animal species.
