# Supplementary material for: Interacting Proteins, Polymorphisms and the Susceptibility of Animals to SARS-CoV-2
Source: Animals (Basel). 2021 Mar 12;11(3):797. doi: 10.3390/ani11030797 (PMC8000148; doi:10.3390/ani11030797)
Supplement: Supplementary file 1 [file animals-11-00797-s001.pdf]

animals MDPI

```
CLUSTAL O(1.2.4) multiple sequence alignment

Cod                         ----------------MSTAGRVAAGAAAMLLLVVALLTPGLRAQVDTETRARAFLEKFS 44
Darter_fish                 --------------------------MFARVLVVLLAVSCTVSAQSDVENKAKEFLQMFD 34
Frog                        --------------------------MSALLWLFSVGLLLATGTSQDVTSQARDFLKQFE 34
Bat                         --------------------------MSGSFWLFLSLVAVAAAQSSTTEGEAREFLDKFN 34
Bat_Little_Brown            --------------------------MSGSSWLFLSLVAVAAAQSST-EEKAKIFLENFN 33
Elephant                    --------------------------MSGSFWLFLSLMAVTAAQST-TEDLARTFLDTFN 33
Seal_low_qual               --------------------------MLGSSWLLLSLAALTAAQST-TEDLVKTFLEKFN 33
Mink                        ------------------------------------------------------------ 0
Ferret                      --------------------------MLGSSWLLLSLAALTAAQST-TEDLAKTFLEKFN 33
Dog_X1                      --------------------------MSGSSWLLLSLAALTAAQS--TEDLVKTFLEKFN 32
Bear                        --------------------------MLGSSWLLLSLAALTAAQST-TEDLAETFLEKFN 33
Cat                         --------------------------MSGSFWLLLSFAALTAAQST-TEELAKTFLEKFN 33
Tiger                       ----------------------------------LSFAALTAAQST-TEELAKTFLEKFN 25
Lemur                       MRENLMRRFYSRERLFSGCDLGSPGKMSSSFWLLLSLISVTAAQST-TEEQAKTFLENFN 59
Rat                         --------------------------MSRSPWLLLSLVAVATAQSL-IEEKAESFLNKFN 33
Mouse                       --------------------------MSSSSWLLLSLVAVTTAQSL-TEENAKTFLNNFN 33
Squirrel                    ----------------MGSCPGARGKMLGSSWLLLSFVAVTAAQST-IEELAKTFLDKFN 43
Rabbit                      --------------------------MSGSSWLLLSLVAVTAAQST-IEELAKTFLEKFN 33
Macaque                     --------------------------MSGSSWLLLSLVAVTAAQST-IEEQAKTFLDKFN 33
Human                       --------------------------MSSSSWLLLSLVAVTAAQST-IEEQAKTFLDKFN 33
Gorilla                     --------------------------MSGSSWLLLSLVAVTAAQST-IEEQAKTFLDKFN 33
Horse                       --------------------------MSGSSWLLLSLVAVTAAQST-TEDLAKTFLEKFN 33
Camel                       --------------------------MSGSFWLLLSLVAVTAAQST-TEELAKTFLEEFN 33
Pig_X1                      --------------------------MSGSFWLLLSLIPVTAAQST-TEELAKTFLEKFN 33
Beluga                      --------------------------MSGSFWLLLSLVAVTAAPST-TEEQAKTFLQKFD 33
Dolphin                     --------------------------MSGSFWLLLSLVAVTAAQSA-TEERAKTFLQKFD 33
Cow_X1                      --------------------------MTGSFWLLLSLVAVTAAQST-TEEQAKTFLEKFN 33
Goat                        --------------------------MTGSFWLLLSLVAVTAAQST-TEEQAKTFLEKFN 33
Sheep                       --------------------------MTGSFWLLLSLVAVTAAQST-TEGQAKTFLEKFN 33
Tiger_Snake_low_qual        MKQALVRKPSSR-SFTHPAFSDXKGNMLSWLCLTWSLVVLALAQDE--TQKAAEFLKQFD 57
Duck                        --------------------------MLAHVLLLCGLSTVVVPQDV--TNQAKMFLAEFN 32
Chicken                     --------------------------MLLHFWLLCGLSAVVTPQDV--TQEAQTFLAEFN 32
Rock-Dove_low_qual          ------------------------MDMLVCIWLLCGLIAVVSPQTV--TQQAQMFLEEFN 34
Rock_Dove_short             ------------------------------------------------------------ 0
```

```
Cod                       TEASVKMYDYSLASWAYNTDITEENSNILSAKGAIWAEFYGRMSTESLAFPLHEVKDPVV 104
Darter_fish               EEATQRMYQYSLASWAYNTNITKENSDKLTKEGEMWGKFYSQMSEESLKFPVDQIKDPEI 94
Frog                      LEAEIIYHQSALAQWEYNTNITDENAQKMSEAGAKWSEFYTKASKASEAFNKDDITDPSI 94
Bat                       SKAENLSHESALASWDYNTNITDKNAQKMNEADSKWSDFYKEQSKLAQAFPLQEIQDSTI 94
Bat_Little_Brown          SKAEDLSHESALASWNYNTNITDENVQKMNEADSKWSAFYEQQSKLAQTYPLQEIQNSTI 93
Elephant                  QEAEDLSYQSSLASWDYNTNITDENVQKMNDAEARWSSFYERQSQLAKDFPIEEISSSII 93
Seal_low_qual             YEAEELSYQSSLASWNYNTNITDENIQKMNDAGAKWSAFYEEQSKQAKTYPLEEIQDSTV 93
Mink                      ------------------------------------------------------------ 0
Ferret                    YEAEELSYQNSLASWNYNTNITDENIQKMNIAGAKWSAFYEEESQHAKTYPLEEIQDPII 93
Dog_X1                    YEAEELSYQSSLASWNYNINITDENVQKMNNAGAKWSAFYEEQSKLAKTYPLEEIQDSTV 92
Bear                      YEAEDLYYQSSLASWNYNTNITNENIQKMNDAGAKWSAFYEEQSKHAKTYPLEEIHNSTV 93
Cat                       HEAEELSYQSSLASWNYNTNITDENVQKMNEAGAKWSAFYEEQSKLAKTYPLAEIHNTTV 93
Tiger                     HEAEELSYQSSLASWNYNTNITDENVQKMNEAGAKWSAFYEEQSKLAETYPLAEIHNTTV 85
Lemur                     NEAEDLSHQSALASWDYNTNITEENAQKMSDHGAKWSAFYEEQSKLAQTYPLEAIQNLTI 119
Rat                       QEAEDLSYQSSLASWNYNTNITEENAQKMNEAAAKWSAFYEEQSKIAQNFSLQEIQDATI 93
Mouse                     QEAEDLSYQSSLASWNYNTNITEENAQKMSEAAAKWSAFYEEQSKTAQSFSLQEIQTPII 93
Squirrel                  QEAEDLDYQRSLAAWNYNTNITEENTQKMNEAEAKWSAFYEEQSKLATAYPLQEIQNFTL 103
Rabbit                    QEAEDLSYQSALASWDYNTNITEENVQKMNDAEAKWSAFYEEQSKLAKTYPSQEVQNLTV 93
Macaque                   HEAEDLFYQSSLASWNYNTNITEENVQNMNNAGEKWSAFLKEQSTLAQMYPLQEIQNLTV 93
Human                     HEAEDLFYQSSLASWNYNTNITEENVQNMNNAGDKWSAFLKEQSTLAQMYPLQEIQNLTV 93
Gorilla                   HEAEDLFYQSSLASWNYNTNITEENVQNMNNAGDKWSAFLKEQSTLAQMYPLQEIQNLTI 93
Horse                     SEAEELSHQSSLASWSYNTNITDENVQKMNEAGARWSAFYEEQCKLAKTYPLEEIQNLTV 93
Camel                     HEAEDLSYQSSLASWNYNTNITDENVQKMNDARAKWSTFYEEKSKTAKTYPLEEIQNVTL 93
Pig_X1                    LEAEDLAYQSSLASWNYNTNITDENIQKMNDARAKWSAFYEEQSRIAKTYPLDEIQTLIL 93
Beluga                    HEAEDLSYQSSLASWNYNTNITDENVQKMNAAGAKWSAFYEEQSKIAKTYPLAEIRNLTL 93
Dolphin                   REAEDLSYQSSLASWNYNTNITDENVQKMNAAGAKWSAFYEEQSRIAKTYPLEEIRNLTL 93
Cow_X1                    HEAEDLSYQSSLASWNYNTNITDENVQKMNEARAKWSAFYEEQSRMAKTYSLEEIQNLTL 93
Goat                      HEAEDLSYQSSLASWNYNTNITDENVQKMNEARAKWSAFYEEQSRMARTYSLEEIQNLTL 93
Sheep                     HEAEDLSYQSSLASWNYNTNITDENVQKMNEARAKWSAFYEEQSRMARTYSLEEIQNLTL 93
Tiger_Snake_low_qual      IRAVDLYYNASIASWNYNTNLTEENAKIMHEKDSIFSRFYDEASRNASMFNVNQISNETI 117
Duck                      VRAEDINYENSLASWDYNTNITEETATKMNEAGAKWSAFYEEASRNASNFPLSDIQDPLL 92
Chicken                   VRAEDISYENSLASWNYNTNITEETARKMSEAGAKWAAFYEEASRNASRFSLANIQDAVT 92
Rock-Dove_low_qual        KRAEDINYESSLASWNYNTNITDETARKMNEAGAKWSAXXXXX-----XXXXTSIQDDLT 89
Rock_Dove_short           ------------------------------------------------------------ 0

Cod                       KLQLISLQDKGSGALSPEKSAHLGRVMSEMSTIYSTAEVCLKDRPTDCQTLEPGLEAVMA 164
Darter_fish               KLQLISLQDQGSGALSPDKAAQLSKVMSEMSTIYSTATVCLIDDPFNCQTLEPGLEHVMA 154
Frog                      KLQLIFLSEKGSAILPANKYARLNQVLNEMSTIYSTHTVCKADGAKDCLQLEPGLDLIMA 154
Bat                       KLQLQILQQNGSSVLTAEKSKRLSTILTTMSTIYSTGKVCNPNNPQQCFTL-SGLEDIME 153
Bat_Little_Brown          KRQLQVLQQNGSSVLSADKSKRLNTILTTMSTIYSTGKVCNPNNPQECFTL-AGLEEIME 152
Elephant                  KLQLQVLQQSGSSVLSPDKSKRLSTILNAMSTIYSTGKTCNPNNPQECLLLEPGLDHIME 153
Seal_low_qual             KRQLQTLQHSGSSVLSADKSQRLNTILNAMSTIYSTGKACKPNNPQECLLLEPGLDDIMA 153
Mink                      ------------------------------------------------------------ 0
Ferret                    KRQLRALQQSGSSVLSADKRERLNTILNAMSTIYSTGKACNPNNPQECLLLEPGLDDIME 153
Dog_X1                    KRQLRALQHSGSSVLSADKNQRLNTILNSMSTIYSTGKACNPSNPQECLLLEPGLDDIME 152
Bear                      KRQLQALQHSGSSVLSADKSQRLNTILNAMSTIYSTGKACNPNNPQECLLLEPGLDDIME 153
Cat                       KRQLQALQQSGSSVLSADKSQRLNTILNAMSTIYSTGKACNPNNPQECLLLEPGLDDIME 153
Tiger                     KRQLQALQQSGSSVLSADKSQRLNTILNAMSTIYSTGKACNPNNPQECLLLEPGLDDIME 145
Lemur                     KRQLRVLQQSGSSGLSADKNKQLNTILNTMSTIYSTGKVCNPNNPQDCLLLEPGLDTIMA 179
Rat                       KRQLKALQQSGSSALSPDKNKQLNTILNTMSTIYSTGKVCNSMNPQECFVLEPGLDEIMA 153
Mouse                     KRQLQALQQSGSSALSADKNKQLNTILNTMSTIYSTGKVCNPKNPQECLLLEPGLDEIMA 153
Squirrel                  KRQLQALQQSGSSALSANKREQLNTILNTMSTIYSTGKVCNPKKPQECLLLEPGLDEIMA 163
Rabbit                    KRQLQALQQSGSSALSADKSKQLNTILSTMSTIYSTGKVCNQSNPQECFLLEPGLDEIMA 153
Macaque                   KLQLQALQQNGSSVLSEDKSKRLNTILNTMSTIYSTGKVCNPNNPQECLLLDPGLNEIME 153
Human                     KLQLQALQQNGSSVLSEDKSKRLNTILNTMSTIYSTGKVCNPDNPQECLLLEPGLNEIMA 153
Gorilla                   KLQLQALQQNGSSVLSEDKSKRLNTILNTMSTIYSTGKVCNPNNPQECLLLEPGLNEIMA 153
Horse                     KRQLQALQQSGSSVLSADKSKRLNEILNTMSTIYSTGKVCNPSNPQECLLLEPGLDAIME 153
Camel                     KRQLQALQQSGASALSADKSKRLTTVLSTMSTIYSSGEVCDPNNPQECLVLEPGLDDIME 153
Pig_X1                    KRQLQALQQSGTSGLSADKSKRLNTILNTMSTIYSSGKVLDPNNPQECLVLEPGLDEIME 153
Beluga                    KRQLQVLQQSGTSVLSADKSKRLNTILSTMSTIYSSGKVLDPN-TQECLVLEPGLDDIME 152
Dolphin                   KRQLQVLQQSGTSVLSADKSKRLNAILSTMSTIYSSGKVLDPN-TQESLVLEPGLDDIME 152
Cow_X1                    KRQLKALQHSGTSALSAEKSKRLNTILNKMSTIYSTGKVLDPN-TQECLALEPGLDDIME 152
Goat                      KRQLKALQHSGTSVLSAEKSKRLNTILNKMSTIYSTGKVLDPN-TQECLALEPGLDDIME 152
Sheep                     KRQLKALQHSGTSVLSAEKSKRLNTILNKMSTIYSTGKVLDPN-TQECLALEPGLDDIME 152
Tiger_Snake_low_qual      KLQIRLLQNGPTDS---STKDQLDTVLRKMSTLYSTGTVCKQDDPFNCLPLEPGLDHIMA 174
Duck                      RLQIQSLQDKGSSVLSPEKYSRLNTVLNTMSTIYSTGTVCKTTAPFDCMVLEPGLDSIMA 152
Chicken                   RLQIQSLQDRGSSVLSPEKYSRLNSVMNSMSTIYSTGVVCKATEPFDCLVLEPGLDDIMA 152
Rock-Dove_low_qual        RLQIQTLQERGSSVLSPEKYSRLSTVLNTMSTIYSTGTVCKINEPSECLVLEPGLDTIMA 149
Rock_Dove_short           -----------------------------MSTIYSTGTVCKINEPSECLVLEPGLDTIMA 31
```

```
Cod                     DSRDYNERLHVWEGWRRETGRKMRPLYEDYVDLKNEAARLNGFEDYGAYWRSNYETVGED 224
Darter_fish             DSTDYFERLHVWEGWRREVGKKMRPLYEDYVDLKNEAAKLNGFEDYGAYWRYNYETIEEE 214
Frog                    ESTDYEERLWAWEGWRAGAGKKMRSLYEEYVDLENEAARLNGYNDYGDYWRGNYETLAAD 214
Bat                     KSKDYHQRLWVWEGWRSEVGKQLRPLYEEYVVLKNEMARGNNYKDYGDYWRGDYETEWGD 213
Bat_Little_Brown        KSKDYNQRLWVWEGWRSEVGKQLRPLYEEYVDLKNEMARGNNYEDYGDYWRGDYETEGED 212
Elephant                NSTDYDQRLWAWEGWRSEVGRQLRPLYEEYVDLKNEMARGNGYEDYGDYWRGDYEAD--- 210
Seal_low_qual           NSRDYNERLWAWEGWRSEVGKQLRPLYEEYVALKNEMARANNYEDYGDYWRGDYEEEWTN 213
Mink                    ------------------------------------------------------------ 0
Ferret                  NSKDYNERLWAWEGWRSEVGKQLRPLYEEYVALKNEMARANNYEDYGDYWRGDYEEEWAD 213
Dog_X1                  NSKDYNERLWAWEGWRSEVGKQLRPLYEEYVALKNEMARANNYEDYGDYWRGDYEEEWEN 212
Bear                    NSKDYNERLWAWEGWRSEVGKQLRPLYEEYVALKNEMARANNYEDYGDYWRGDYEEEWTD 213
Cat                     NSKDYNERLWAWEGWRAEVGKQLRPLYEEYVALKNEMARANNYEDYGDYWRGDYEEEWTD 213
Tiger                   NSKDYNERLWAWEGWRAEVGKQLRPLYEEYVALKNEMARANNYEDYGDYWRGDYEEEWTD 205
Lemur                   NSRDYSQRLWAWEGWRSEVGKQLRPLYEEYVVLKNEMARANNYEDYGDYWRADYEAEGES 239
Rat                     TSTDYNRRLWAWEGWRAEVGKQLRPLYEEYVVLKNEMARANNYEDYGDYWRGDYEAEGVE 213
Mouse                   TSTDYNSRLWAWEGWRAEVGKQLRPLYEEYVVLKNEMARANNYNDYGDYWRGDYEAEGAD 213
Squirrel                NSTDYNERLWVWEGWRSEVGKQLRPLYEEYVVLKNEMARANNYEDYGDYWRGDYEAEGAD 223
Rabbit                  KSTDYNERLWAWEGWRSVVGKQLRPLYEEYVVLKNEMARANNYEDYGDYWRADYEAEGAD 213
Macaque                 KSLDYNERLWAWEGWRSEVGKQLRPLYEEYVVLKNEMARANHYKDYGDYWRGDYEVNGVD 213
Human                   NSLDYNERLWAWESWRSEVGKQLRPLYEEYVVLKNEMARANHYEDYGDYWRGDYEVNGVD 213
Gorilla                 NSLDYSERLWAWESWRSEVGKQLRPLYEEYVVLKNEMARANHYEDYGDYWRGDYEVNGVD 213
Horse                   NSKDYNQRLWAWEGWRSEVGKQLRPLYEEYVVLKNEMARANNYEDYGDYWRGDYEAEGPS 213
Camel                   NSKDYNQRLWAWEGWRAEVGKQLRPLYEEYVVLKNEMARANNYEDYGDYWRGDYEVMWAG 213
Pig_X1                  NSKDYSRRLWAWESWRAEVGKQLRPLYEEYVVLENEMARANNYEDYGDYWRGDYEVTGTG 213
Beluga                  NSKDYNRRLWAWEGWRAEVGKQLRPFYEEYVVLENEMARANNYEDYGDYWRGNYEVTGAG 212
Dolphin                 NSKDYNRRLWAWEGWRAEVGKQLRPLYEEYVVLENEMARANNYEDYGDYWRGDYEVTGAG 212
Cow_X1                  NSRDYNRRLWAWEGWRAEVGKQLRPLYEEYVVLENEMARANNYEDYGDYWRGDYEVTGAG 212
Goat                    NSRDYNRRLWAWEGWRAEVGKQLRPLYEEYVVLENEMARANNYEDYGDYWRGDYEVTGAG 212
Sheep                   NSRDYNRRLWAWEGWRAEVGKQLRPLYEEYVVLENEMARANNYEDYGDYWRGDYEVTGAG 212
Tiger_Snake_low_qual    NNWNYSERLWAWESWRADVGKKMRPLYETYVELKNKYARLRGYDDYGDYWRANYEVDLPG 234
Duck                    NSIDYHERLWAWEGWRADVGRMMRPLYEEYVDLKNEAAKLNGYADYGDYWRANYEADYPE 212
Chicken                 NSIDYHERLWAWEGWRADVGRMMRPLYEEYVELKNEAARLNNYSDYGDYWRANYETDYPE 212
Rock-Dove_low_qual      NSTDYHERLWAWEGWRADVGRMMRPLYKEYVELKNEVATLNGYSDYGDYWRANYEADYLE 209
Rock_Dove_short         NSTDYHERLWAWEGWRADVGRMMRPLYKEYVELKNEVATLNGYSDYGDYWRANYEADYLE 91


Cod                     PPYNYTRDELMGDVRSIYKEIMPLYKELHAYVRSKLIETYPGGHIHPEGPLPAHLLGDMW 284
Darter_fish             IQYKYTGDGLMKDVRSLYNEILPLYMELHAYVRARLMEVYPG-HIDSEGPLPAHLLGDMW 273
Frog                    -KYAYSRDDLIQDVERTYQEILPLYKELHAYVRGNLQKVYGSQYISDTGCLPAHLLGDMW 273
Bat                     -GYNYSRNHLMEDVDRIFLEIKPLYEQLHAYVRAKLMNAYP-SRISPTGCLPAHLLGDMW 271
Bat_Little_Brown        -GYNYSRNQLTEDVERIFLEIKPLYEHLHAYVRAKLVNAYP-SRISPTGYLPAHLLGDMW 270
Elephant                ---NYDRSQLIKDVEETFAQIKPLYEHLHAYVRRKLMDVYP-NRINQTGCLPAHLLGDMW 266
Seal_low_qual           -GYNYSRDQLIKDVEQTFTQIQPLYQHLHAYVRQKLMDTYP-SHISPTGCLG-HLLSDMW 270
Mink                    ------------------------------------------------------------ 0
Ferret                  -GYSYSRNQLIEDVEHTFTQIKPLYEHLHAYVRAKLMDAYP-SRISPTGCLPAHLLGDMW 271
Dog_X1                  -GYNYSRNQLIDDVEHTFTQIMPLYQHLHAYVRTKLMDTYP-SYISPTGCLPAHLLGDMW 270
Bear                    -GYNYSRNQLIEDVEHTFTQIKPLYEHLHAYVRAKLMDTYP-SRISPTGCLPAHLLGDMW 271
Cat                     -GYNYSRSQLIKDVEHTFTQIKPLYQHLHAYVRAKLMDTYP-SRISPTGCLPAHLLGDMW 271
Tiger                   -GYNYSRSQLIKDVEHTFTQIKPLYQHLHAYVRAKLMDSYP-SRISPTGCLPAHLLGDMW 263
Lemur                   -GYNYNRSQLMEDVEHTFAQIKPLYEHLHAYVRAKLMNVYP-SHINPNGCLPAHLLGDMW 297
Rat                     -GYNYNRNQLIEDVENTFKEIKPLYEQLHAYVRTKLMDVYP-SYISPTGCLPAHLLGDMW 271
Mouse                   -GYNYNRNQLIEDVERTFAEIKPLYEHLHAYVRRKLMDTYP-SYISPTGCLPAHLLGDMW 271
Squirrel                -GYGYNRNQLIEDVERTFAEIKPLYEHLHAYVRAKLMNTYP-SYISPTGCLPAHLLGDMW 281
Rabbit                  -GYDYSRSQLIDDVERTFSEIKPLYEQLHAYVRTKLMDAYP-SRISPTGCLPAHLLGDMW 271
Macaque                 -GYDYNRDQLIEDVERTFEEIKPLYEHLHAYVRAKLMNAYP-SYISPTGCLPAHLLGDMW 271
Human                   -GYDYSRGQLIEDVEHTFEEIKPLYEHLHAYVRAKLMNAYP-SYISPIGCLPAHLLGDMW 271
Gorilla                 -GYDYSRGQLIEDVEHTFEEIKPLYEHLHAYVRAKLMNAYP-SYISPIGCLPAHLLGDMW 271
Horse                   -GYDYSRDQLIEDVERTFAEIKPLYEHLHAYVRAKLMDTYP-SHINPTGCLPAHLLGDMW 271
Camel                   -DYDYSRDQLMGDVEHTFAEIKPLYEHLHAYVRAKLMDVYP-SHISPTGCLPAHLLGDMW 271
Pig_X1                  -DYDYSRNQLMEDVERTFAEIKPLYEHLHAYVRAKLMDAYP-SRISPTGCLPAHLLGDMW 271
Beluga                  -DYDYSRDQLITDVERTFAEIKPLYEQLHAYVRAKLMDAYP-SRISPTGCIPAHLLGDMW 270
Dolphin                 -DYDYSRDQLIRDVERTFAEIKPLYEQLHAYVRAKLMDAYP-SRISPTGCLPAHLLGDMW 270
Cow_X1                  -DYDYSRDQLMKDVERTFAEIKPLYEQLHAYVRAKLMHTYP-SYISPTGCLPAHLLGDMW 270
Goat                    -DYDYSRDQLMKDVERTFAEIKPLYEQLHAYVRAKLMNTYP-SYISPTGCLPAHLLGDMW 270
Sheep                   -DYDYSRDQLMKDVERTFEEIKPLYEQLHAYVRAKLMDTYP-SYISPTGCLPAHLLGDMW 270
Tiger_Snake_low_qual    -KFQYQRAQLITDVENTFKQILPLYEQLHAYVRRHLYKRYGPELINPKGAIPAHLLGDMW 293
Duck                    -EYKYSRDQLIQDVEKTFEQIKPLYQQLHAYVRHRLEQAYGSQFISSTGCLPAHLLGDMW 271
Chicken                 -EYKYSRDQLVQDVEKTFEQIKPLYQHLHAYVRHRLEQVYGSELINPTGCLPAHLLGDMW 271
Rock-Dove_low_qual      -EYKYSRDQLVEDVEKTFEQIKPLYQQLHAYVRHRLEQVYGPKLISSTGCLPAHLLGDMW 268
Rock_Dove_short         -EYKYSRDQLVEDVEKTFEQIKPLYQQLHAYVRHRLEQVYGPKLISSTGCLPAHLLGDMW 150
```

```
Cod                     GRFWTSLYPLSTPYPLKPDIDVSTAMVDQKWVPERLFREAEKFFMSVGLYKMEPDFWKNS 344
Darter_fish             GRFWTNLYRLSVPYPNKEDIDVSNTMVDKGWIEERLFKEAEKFFMSVGLYEMFPNFWNNS 333
Frog                    GRFWTNLYPLMIPYANKESIDVTPNMVAQGWTIERMFKEAEIFFKSVDLFALNENFWNNS 333
Bat                     GRFWTNLYNLTVPFEQKQNIDVTETMKKQSWDADKIFKEAEKFYLSVGLRNMTPGFWNKS 331
Bat_Little_Brown        GRFWTNLYNLTVPFEQKPNIDVTGAMVEQSWDAEKIFKEAEKFYISVGLPSMTPGFWNNS 330
Elephant                GRFWTNLYPLTVPFGHKPNIDVTEAMVKQDWNATKIFKEAEKFFMSVGLPPMTQGFWENS 326
Seal_low_qual           GRFWTNLYPLTVPXGQKPNIDVTDAMVNQFVSNRIQ-PC-PSLYI--------------- 313
Mink                    -----------------------------------------------GLPNMTEGFWQNS 13
Ferret                  GRFWTNLYPLMVPFRQKPNIDVTDAMVNQSWDARRIFEEAETFFVSVGLPNMTEGFWQNS 331
Dog_X1                  GRFWTNLYPLTVPFGQKPNIDVTNAMVNQSWDARKIFKEAEKFFVSVGLPNMTQEFWENS 330
Bear                    GRFWTNLYPLTIPFGQKPNIDVTDAMVNQNWDARRIFEEAEKFFVSVGLPNMTQEFWENS 331
Cat                     GRFWTNLYPLTVPFGQKPNIDVTDAMVNQSWDARRIFKEAEKFFVSVGLPNMTQGFWENS 331
Tiger                   GRFWTNLYPLTVPFGQKPNIDVTDAMVNQSWDARRIFKEAEKFFVSVGLPNMTQGFWENS 323
Lemur                   GRFWTNLYSLTVPFEQKPNIDVTDAMVNQAWDANRILKEAENFFVSVGLPNMTQGFWEKS 357
Rat                     GRFWTNLYPLTTPFLQKPNIDVTDAMVNQSWDAERIFKEAEKFFVSVGLPQMTPGFWTNS 331
Mouse                   GRFWTNLYPLTVPFAQKPNIDVTDAMMNQGWDAERIFQEAEKFFVSVGLPHMTQGFWANS 331
Squirrel                GRFWTNLYSLTVPFPEKPNIDVTDAMINQNWNAVRIFKEAEKFFVSVGLPNMTQGFWENS 341
Rabbit                  GRFWTNLYSLTVPFGQKPNIDVTDTMVNQGWDAERIFKEAEKFFVSVGLPSMTHGFWENS 331
Macaque                 GRFWTNLYSLTVPFGQKPNIDVTDAMVNQAWNAQRIFKEAEKFFVSVGLPNMTQGFWENS 331
Human                   GRFWTNLYSLTVPFGQKPNIDVTDAMVDQAWDAQRIFKEAEKFFVSVGLPNMTQGFWENS 331
Gorilla                 GRFWTNLYSLTVPFGQKPNIDVTDAMVDQAWDAQRIFKEAEKFFVSVGLPNMTQGFWENS 331
Horse                   GRFWTNLYSLTVPFGQKPNIDVTDAMVDQSWDAKRIFEEAEKFFVSVGLPNMTQGFWENS 331
Camel                   GRFWTNLYSLTVPFGQKPNIDVTEAMENQSWDAKRIFKEAEKFFVSIGLPNMTQGFWDNS 331
Pig_X1                  GRFWTNLYPLTVPFGEKPSIDVTEAMVNQSWDAIRIFEEAEKFFVSIGLPNMTQGFWNNS 331
Beluga                  GRFWTNLYPLTVPFGERPSIDVTKEMQNQSWDAKRIFKEAEKFFVSIGLPNMTQEFWDNS 330
Dolphin                 GRFWTNLYPLTVPFGERPSIDVTKEMQNQSWDAKRIFKEAEKFFVSIGLPNMTQGFWDNS 330
Cow_X1                  GRFWTNLYSLTVPFEHKPSIDVTEKMENQSWDAERIFKEAEKFFVSISLPYMTQGFWDNS 330
Goat                    GRFWTNLYSLTVPFEHKPSIDVTEKMKNQSWDAERIFKEAEKFFVSIGLPYMTQGFWNNS 330
Sheep                   GRFWTNLYSLTVPFEHKPSIDVTEKMKNQSWDAERIFKEAEKFFVSIGLPYMTQGFWDNS 330
Tiger_Snake_low_qual    GRFWTNLYPLMVPYPNKTSIDVSSAMVEKKWTVDSIFKAAEHFFISIGLFNMTESFWKNS 353
Duck                    GRFWTNLYPLTVPYPAKPNIDVTDAMVQKNWDAVKIFKAAEAFFSSIGLYNMTEGFWKNS 331
Chicken                 GRFWTNLYNLTVPYPEKPNIDVTSAMAQKNWDAMKIFKTAEAFFASIGLYNMTEGFWTNS 331
Rock-Dove_low_qual      GRFWTNLYPLTVPYPAKPNIDVTSAMVEKKWDAIKIFKAAEAFFVSIGLFEMTEGFWNNS 328
Rock_Dove_short         GRFWTNLYPLTVPYPAKPNIDVTSAMVEKKWDAIKIFKAAEAFFVSIGLFEMTEGFWNNS 210

Cod                     MLEKPND-RKVVCHPTAWDMGNGKDYRIKMCTQVNMDHFLTAHHEMGHNQYQTAYQNLSY 403
Darter_fish             MLVKPSDGRKVVCHPTAWDMGNREDFRIKMCTKVNMDDFLTAHHEMGHNQYQMAYRNLSY 393
Frog                    MLEEPKDGRQVVCHPTAWDLGMN-DFRIKMCTKVNMEDFLTVHHELGHIQYDMAYAKQPF 392
Bat                     MLTEPSDGRQVVCHPTAWDLGKD-DFRIKMCTKVTMDDFLTAHHEMGHIQYDMAYAKQPY 390
Bat_Little_Brown        MLTEPGDGRKVVCHPTAWDLGKG-DFRIKMCTKVTMDDFLTAHHEMGHIQYDMAYATQPY 389
Elephant                MLTEPGDGRKVVCHPTAWDLGKG-DFRIKMCTKVTMDDFLTAHHEMGHIQYDMAYAIQPY 385
Seal_low_qual           ---------------EHLVLFLG-HKRIKMCTKVTMDDFLTAHHEMGHIQYDMAYAVQPF 357
Mink                    MLTEPGDNRKVVCHPTAWDLGKH-DFRIKMCTKVTMDDFLTAHHEMGHIQYDMAYAAQPF 72
Ferret                  MLTEPGDNRKVVCHPTAWDLGKR-DFRIKMCTKVTMDDFLTAHHEMGHIQYDMAYAEQPF 390
Dog_X1                  MLTEPSDSRKVVCHPTAWDLGKG-DFRIKMCTKVTMDDFLTAHHEMGHIQYDMAYAAQPF 389
Bear                    MLTEPGDGQKVVCHPTAWDLGKG-DFRIKMCTKVTMDDFLTAHHEMGHIQYDMAYAEQPF 390
Cat                     MLTEPGDSRKVVCHPTAWDLGKG-DFRIKMCTKVTMDDFLTAHHEMGHIQYDMAYAVQPF 390
Tiger                   MLTEPGNSQKVVCHPTAWDLGKG-DFRIKMCTKVTMDDFLTAHHEMGHIQYDMAYAVQPF 382
Lemur                   MLTEPEDGRKVICHPTAWDLGKG-DFRIKMCTKVTMDNFLTAHHEMGHIQYDMAYAIQPF 416
Rat                     MLTEPGDDRKVVCHPTAWDLGHG-DFRIKMCTKVTMDNFLTAHHEMGHIQYDMAYAKQPF 390
Mouse                   MLTEPADGRKVVCHPTAWDLGHG-DFRIKMCTKVTMDNFLTAHHEMGHIQYDMAYARQPF 390
Squirrel                MLTEPTDGRKVVCHPTAWDLQKG-DFRIKMCTKVTMDNFLTAHHEMGHIQYDMAYAMQPY 400
Rabbit                  MLPESGDGRKVVCHPTAWDLGKR-DFRIKMCTKVTMDNFLTAHHEMGHIQYDMAYATQPF 390
Macaque                 MLTDPGNVQKVVCHPTAWDLGKG-DFRIIMCTKVTMDDFLTAHHEMGHIQYDMAYAAQPF 390
Human                   MLTDPGNVQKAVCHPTAWDLGKG-DFRILMCTKVTMDDFLTAHHEMGHIQYDMAYAAQPF 390
Gorilla                 MLTDPGNVQKAVCHPTAWDLGKG-DFRILMCTKVTMDDFLTAHHEMGHIQYDMAYAAQPF 390
Horse                   MLTEPGDGRKVVCHPTAWDLGKG-DFRIKMCTKVTMDDFLTAHHEMGHIQYDMAYAVQPY 390
Camel                   MLTEPGDGRKVVCHPTAWDLGKG-DFRIKMCTKVTMDDFLTAHHEMGHIQYDMAYAIQPF 390
Pig_X1                  MLTEPGDGRKVVCHPTAWDLGKG-DFRIKMCTKVTMDDFLTAHHEMGHIQYDMAYAIQPY 390
Beluga                  MLTEPGDGRKVVCHPTAWDLGKG-DFRIKMCTKVTMDGFLTAHHEMGHIQYDMAYATQPY 389
Dolphin                 MLTEPGDGRKVVCHPTAWDLGKG-DFRIKMCTKVTMDDFLTAHHEMGHIQYDMAYATQPY 389
Cow_X1                  MLTEPGDGRKVVCHPTAWDLGKG-DFRIKMCTKVTMDDFLTAHHEMGHIQYDMAYAAQPY 389
Goat                    MLTEPGDGRKVVCHPTAWDLGKG-DFRIKMCTKVTMDDFLTAHHEMGHIQYDMAYATQPY 389
Sheep                   MLTEPGDGRKVVCHPTAWDLGKG-DFRIKMCTKVTMDDFLTAHHEMGHIQYDMAYATQPY 389
Tiger_Snake_low_qual    MLEEPKDGRKVVCHPTAWDMGKE-DYRIKMCTKINMEDFLTAHHEMGHIEYDMAYADQPF 412
Duck                    MLTEPTDNRKVVCHPTAWDMGKN-DYRIKMCTKVTMDDFLTAHHEMGHIEYDMAYSQQPF 390
Chicken                 MLTEPTDNRKVVCHPTAWDMGKN-DYRIKMCTKVTMDDFLTAHHEMGHIEYDMAYSVQPF 390
Rock-Dove_low_qual      MLTEPADNRKVVCHPTAWDLGKN-DYRIKMCTKVTMDDFLTAHHEMGHIEYDMAYSVQPY 387
Rock_Dove_short         MLTEPADNRKVVCHPTAWDLGKN-DYRIKMCTKVTMDDFLTAHHEMGHIEYDMAYSVQPY 269
                                           :    . ** ***::.*: ***.***:** :*: **    :
```

```
Cod                     LLRDGANEGFHEAVGEIMSLSAATPDHLKSLGLLAADFTADKETEINFLMKQALTIVATL 463
Darter_fish             LLREGANEGFHEAVGEIMSLSAATPKHLQSLNLLAPDFIYDNETEINFLLKQALTIVATL 453
Frog                    MLRDGANEGFHEAVGEIMSLSAATPKHLKHIKLLDENFIEDQEVEINFLLRQALAIVGTL 452
Bat                     LLRNGANEGFHEAVGEVMSLSVATPKHLKGMGLLPSDFSEDLETEKNFLLKQALNIVGTL 450
Bat_Little_Brown        LLRNGANEGFHEAVGEVMSLSVATPKHLKGMGLLPPDFSEDNETEINFLLKQALNIVGTL 449
Elephant                LLRNGANEGFHEAVGEIMSLSAATPEHLKAIGLLPSDFQEDTETELNFLLKQALTIVGTL 445
Seal_low_qual           LLRNGANEGFHEAVGEIMSLSAATPKHLKNIGLLPPGFSEDNETDINFLFKQALTIVGTL 417
Mink                    LLRNGANEGFHEAVGEIMSLSAATPNHLKNIGLLPPDFSEDSETDINFLLKQALTIVGTL 132
Ferret                  LLRNGANEGFHEAVGEIMSLSAATPNHLKNIGLLPPDFSEDSETDINFLLKQALTIVGTL 450
Dog_X1                  LLRNGANEGFHEAVGEIMSLSAATPNHLKNIGLLPPSFFEDSETEINFLLKQALTIVGTL 449
Bear                    LLRNGANEGFHEAVGEIMSLSAATPNHLKNIGLLPPGFSEDNETEINFLLKQALTIVGTL 450
Cat                     LLRNGANEGFHEAVGEIMSLSAATPNHLKTIGLLSPGFSEDSETEINFLLKQALTIVGTL 450
Tiger                   LLRNGANEGFHEAVGEIMSLSAATPNHLKTIGLLPPGFSEDSETEINFLLKQALTIVGTL 442
Lemur                   LLRNGANEGFHEAVGEIMSLSAATPKHLKSIGLLPPDFQEDDETEINFLLKQALTIVGTL 476
Rat                     LLRNGANEGFHEAVGEIMSLSAATPKHLKSIGLLPSNFQEDDETEINFLLKQALIIVGTL 450
Mouse                   LLRNGANEGFHEAVGEIMSLSAATPKHLKSIGLLPSDFQEDSETEINFLLKQALTIVGTL 450
Squirrel                LLRNGANEGFHEAVGEIMSLSASTPKHLKSIGLLPSDFREDSETEINFLLKQALTIVGTL 460
Rabbit                  LLRNGANEGFHEAVGEIMSLSAATPEHLKSIGLLPYDFHEDNETEINFLLKQALTIVGTL 450
Macaque                 LLRNGANEGFHEAVGEIMSLSAATPKHLKSIGLLSPDFQEDNETEINFLLKQALTIVGTL 450
Human                   LLRNGANEGFHEAVGEIMSLSAATPKHLKSIGLLSPDFQEDNETEINFLLKQALTIVGTL 450
Gorilla                 LLRNGANEGFHEAVGEIMSLSAATPKHLKSIGLLSPDFQEDNETEINFLLKQALTIVGTL 450
Horse                   LLRNGANEGFHEAVGEIMSLSAATPNHLKAIGLLPPDFYEDSETEINFLLKQALTIVGTL 450
Camel                   LLRNGANEGFHEAVGEIMSLSAATPHYLKALGLLPADFYEDSETEINFLLKQALTIVGTL 450
Pig_X1                  LLRNGANEGFHEAVGEIMSLSAATPHYLKALGLLPPDFYEDSETEINFLLKQALTIVGTL 450
Beluga                  LLRNGANEGFHEAVGEIMSLSAATPHYLKALGLLPPDFYEDRVTEINFLLKQALTIVGTL 449
Dolphin                 LFRNGANEGFHEAVGEIMSLSAATPHYLKALGLLPPDFYEDSATEINFLLKQALTIVGTL 449
Cow_X1                  LLRNGANEGFHEAVGEIMSLSAATPHYLKALGLLAPDFHEDNETEINFLLKQALTIVGTL 449
Goat                    LLRNGANEGFHEAVGEIMSLSAATPHYLKALGLLAPDFYEDNETEINFLLKQALTIVGTL 449
Sheep                   LLRNGANEGFHEAVGEIMSLSAATPHYLKALGLLAPDFYEDNETEINFLLKQALTIVGTL 449
Tiger_Snake_low_qual    LLRNGANEGFHEAVGEIMSLSAATPKYLQSLGLLESTFQEDAETDINFLLRQALTIVGTM 472
Duck                    LLRGGANEGFHEAVGEIMSLSAATPEHLKSLDLLEPTFQEDEETEINFLLKQALTIVGTM 450
Chicken                 LLRDGANEGFHEAVGEIMSLSAATPQHLKSLDLLEPTFQEDEETEINFLLKQALTIVGTM 450
Rock-Dove_low_qual      LLRDGANEGFHEAVGEIMSLSAATPQHLKSLDLLEPTFQEDEETEINFLLKQALTIIGTM 447
Rock_Dove_short         LLRDGANEGFHEAVGEIMSLSAATPQHLKSLDLLEPTFQEDEETEINFLLKQALTIIGTM 329
                        ::* ************:****.:**.:*: : **   *  *  .: ***::*** *:.*:


Cod                     PFTYMLEEWRWQVFNENIPKNQWMKRWWEMKRDLVGVVEPVPRDETYCDPPALFHVSGDY 523
Darter_fish             PFTYMLEEWRWQVFAGNITKDNWMKRWWEMKRELVGVVEPVPRDETYCDPPALFHVSGDY 513
Frog                    PFTYMLEQWRWKAFRGEIPKEQWMKTWWQMKRDLVGVVEPVPHDETYCDPPALFHVSNDY 512
Bat                     PFTYMLEKWRWMVFEGKIPKEQWMEKWWEMKREIVGVVEPLPHDETYCDPASLFHVANDY 510
Bat_Little_Brown        PFTYMLEKWRWMVFKGEIPKEQWMKKWWEMKRDIVGVMEPLPHDETYCDPASLFHVANDY 509
Elephant                PFTYMLEKWRWMVFKGEIPREQWMKKWWEMKREIVGVVEPVPHDESYCDPATLFHVANDY 505
Seal_low_qual           PFTYMLEKWRWMVFKGEIPKEQWIKKWWEMKRDLVGVVEPLPHDETYCDPASLFHVANDY 477
Mink                    PFTYMLEKWRWMVFKGEIPKEQWMQKWWEMKRDIVGVVEPLPHDETYCDPAALFHVANDY 192
Ferret                  PFTYMLEKWRWMVFKGEIPKEQWMQKWWEMKRDIVGVVEPLPHDETYCDPAALFHVANDY 510
Dog_X1                  PFTYMLEKWRWMVFKGEIPKDQWMKTWWEMKRNIVGVVEPVPHDETYCDPASLFHVANDY 509
Bear                    PFTYMLEKWRWMVFQGKIPKEQWMKKWWEMKRDIVGVVEPLPHDETYCDPASLFHVANDY 510
Cat                     PFTYMLEKWRWMVFKGEIPKEQWMQKWWEMKREIVGVVEPVPHDETYCDPASLFHVANDY 510
Tiger                   PFTYMLEKWRWMVFKGEIPKEQWMQKWWEMKREIVGVVEPVPHDETYCDPASLFHVANDY 502
Lemur                   PFTYMLEKWRWMVFKGEIPKDQWMKKWWEMKREIVGVVEPVPHDETYCDPASLFHVSNDY 536
Rat                     PFTYMLEKWRWMVFQDKIPREQWTQKWWEMKREIVGVVEPLPHDETYCDPASLFHVSNDY 510
Mouse                   PFTYMLEKWRWMVFRGEIPKEQWMKKWWEMKREIVGVVEPLPHDETYCDPASLFHVSNDY 510
Squirrel                PFTYMLEKWRWMVFKGEIPKDQWMKKWWEMKREIVGVMEPVPHDETYCDPAALYHVSNDF 520
Rabbit                  PFTYMLEKWRWMVFKGEIPKEQWMQKWWEMKREIVGVVEPMPHDETYCDPAALFHVANDY 510
Macaque                 PFTYMLEKWRWMVFKGEIPKDQWMKKWWEMKREIVGVVEPVPHDETYCDPASLFHVSNDY 510
Human                   PFTYMLEKWRWMVFKGEIPKDQWMKKWWEMKREIVGVVEPVPHDETYCDPASLFHVSNDY 510
Gorilla                 PFTYMLEKWRWMVFKGEIPKDQWMKKWWEMKREIVGVVEPVPHDETYCDPASLFHVSNDY 510
Horse                   PFTYMLEKWRWMVFKGEIPKEEWMKKWWEMKREIVGVVEPVPHDETYCDPAALFHVANDY 510
Camel                   PFTYMLEKWRWMVFKGEIPKEQWMQKWWEMKREIVGVVEPLPHDETYCDPACLFHVAEDY 510
Pig_X1                  PFTYMLEKWRWMVFKGEIPKEQWMQKWWEMKREIVGVVEPLPHDETYCDPACLFHVAEDY 510
Beluga                  PFTYMLEKWRWMVFKGEIPKEQWMQKWWEMKREIVGVVEPLPHDETYCDPACLFHVAEDY 509
Dolphin                 PFTYMLEKWRWMVFKGEIPKEQWMQKWWEMKREIVGVVEPLPHDETYCDPACLFHVAEDY 509
Cow_X1                  PFTYMLEKWRWMVFKGEIPKQQWMEKWWEMKREIVGVVEPLPHDETYCDPACLFHVAEDY 509
Goat                    PFTYMLEKWRWMVFKGEIPKQQWMEKWWEMKREIVGVVEPLPHDETYCDPACLFHVAEDY 509
Sheep                   PFTYMLEKWRWMVFKGEIPKQQWMEKWWEMKREIVGVVEPLPHDETYCDPACLFHVAEDY 509
Tiger_Snake_low_qual    PFTYMLEKWRWMVFAEQIPKDQWMKKWWEMKREIVGVVEPLPHNEEYCDPAALFHVANDY 532
Duck                    PFTYMLEKWRWMVFRGEITKQEWTKQWWEMKRDIVGVVEPVPHDETYCDPAALFHVANDY 510
Chicken                 PFTYMLEKWRWMVFNGEITKQEWTKRWWKMKREIVGVVEPVPHDETYCDPAALFHVANDY 510
Rock-Dove_low_qual      PFTYMLEKWRWMVFMNFCF-------SFILRREIVGVVEPVPHDETYCDPAALFHVANDY 500
Rock_Dove_short         PFTYMLEKWRWMVFM---------------KREIVGVVEPVPHDETYCDPAALFHVANDY 374
                        *******:*** .*                :*::***:**:*::* ****  *:**: *:
```

```
Cod                           SFIRYFTRTIYQFQFQKALCKEAGHKGELFTCDITNSTLAGTKLRNMLTLGRSKSWTKAL 583
Darter_fish                   SFIRYFTRTIYQFQFQKALCDAANHPGALSTCDITNSTVAGTKLRNMLELGRSKSWTRAL 573
Frog                          SFIRYYTRTIYQFQFQDALCKAAGHTGPLHSCDITNSKEAGAKLRAMLELGKAKSWTEAL 572
Bat                           SFIRYFTRTILEFQFQEALCKIAKHQGPLHKCDISNSTEAGKKLKDMLELGKSKPWTLAL 570
Bat_Little_Brown              SFIRYFTRTIFEFQFQEALCQIAKHQGPLHKCDISNSKEAGNKLLEMLKLGKSEPWTLAL 569
Elephant                      SFIRYYTRTIYQFQFQEALCQVAKHEGPLYKCDISNSTEAGQKLLGMLSLGKSEPWTSAL 565
Seal_low_qual                 SFIRYYTRTIYQFQFQEALCQIAKHEGPLHKCDISNSSEAGQKLLQMLKLGRSKPXTLAL 537
Mink                          SFIRYYTRTIYQFQFQEALCQIAKHEGPLYKCDISNSREAGQKLHEMLSLGRSKPWTFAL 252
Ferret                        SFIRYYTRTIYQFQFQEALCQIAKHEGPLYKCDISNSSEAGQKLHEMLSLGRSKPWTFAL 570
Dog_X1                        SFIRYYTRTIYQFQFQEALCQIAKHEGPLHKCDISNSSEAGQKLLEMLKLGKSKPWTYAL 569
Bear                          SFIRYYTRTIYQFQFQEALCQIAKHEGPLHKCDISNSSEAGKTLLQMLRLGRSKPWTLAL 570
Cat                           SFIRYYTRTIYQFQFQEALCRIAKHEGPLHKCDISNSSEAGKKLLQMLTLGKSKPWTLAL 570
Tiger                         SFIRYYTRTIYQFQFQEALCRIAKHEGPLHKCDISNSSEAGKKLLQMLTLGKSKPWTLAL 562
Lemur                         SFIRYYTRTIYQFQFQEALCRAAKHEGPLHRCDISNSTEAGQKLLNMLRLGKSEPWTLAL 596
Rat                           SFIRYYTRTIYQFQFQEALCQAAKHDGPLHKCDISNSTEAGQKLLNMLSLGNSGPWTLAL 570
Mouse                         SFIRYYTRTIYQFQFQEALCQAAKYNGSLHKCDISNSTEAGQKLLKMLSLGNSEPWTKAL 570
Squirrel                      SFIRYYTRTIYQFQFQEALCQAAKHEGPLHKCDISNSTEAGQKLLNMLRLGKSKPWTLAL 580
Rabbit                        SFIRYYTRTIYQFQFQEALCQAAQHEGPLHKCDISNSTEAGQKLLNMLRLGRSEPWTLAL 570
Macaque                       SFIRYYTRTLYQFQFQEALCQAAKHEGPLHKCDISNSTEAGQKLLNMLKLGKSEPWTLAL 570
Human                         SFIRYYTRTLYQFQFQEALCQAAKHEGPLHKCDISNSTEAGQKLFNMLRLGKSEPWTLAL 570
Gorilla                       SFIRYYTRTLYQFQFQEALCQAAKHEGPLHKCDISNSTEAGQKLFNMLRLGKSEPWTLAL 570
Horse                         SFIRYYTRTIYQFQFQEALCQTAKHEGPLHKCDISNSTEAGQKLLQMLSLGKSEPWTLAL 570
Camel                         SFIRYYTRTIYQFQFHEALCQIAKHEGPLYKCDISNSTEAGQKLLQMLSLGKSEPWTLAL 570
Pig_X1                        SFIRYYTRTIYQFQFHEALCRTAKHEGPLYKCDISNSTEAGQKLLQMLSLGKSEPWTLAL 570
Beluga                        SFIRYYTRTIYQFQFHEALCQTAKHEGPLYKCDISNSTEAGQRLLQMLRLGKSEPWTSAL 569
Dolphin                       SFIRYYTRTIYQFQFHEALCQTAKHEGPLYKCDISNSTEAGQRLLQMLHLGKSEPWTSAL 569
Cow_X1                        SFIRYYTRTIYQFQFHEALCKTAKHEGALFKCDISNSTEAGQRLLQMLRLGKSEPWTLAL 569
Goat                          SFIRYYTRTIYQFQFHEALCKTAKHEGALFKCDISNSTEAGQRLLQMLRLGKSEPWTLAL 569
Sheep                         SFIRYYTRTIYQFQFHEALCKTAKHEGALFKCDISNSTEAGQRLLQMLRLGKSEPWTLAL 569
Tiger_Snake_low_qual          SFIRYYTRTIYQFQFQEALCKAAGHTEELYKCDISNSTNAGRILKDMLALGSSQPWTKAL 592
Duck                          SFIRYYTRTIYQFQFHEALCKAANHTGPLHTCDITNSTAAGGSLRELLKLGRSKPWTQAL 570
Chicken                       SFIRYYTRTIYQFQFQEALCKAANHTGPLHKCDITNSTAAGGNLRQLLELGKSKPWTQAL 570
Rock-Dove_low_qual            SFIRYYTRTIYQFQFQEALCKAANHTGPLHTCDITNSTAAGQNLRELLELGRSKPWTQAL 560
Rock_Dove_short               SFIRYYTRTIYQFQFQEALCKAANHTGPLHTCDITNSTAAGQNLRELLELGRSKPWTQAL 434
                              *****:***: :***:.***  * :   *  ***:**  **  *  :* ** :   * **

Cod                           EMISGDTKMDAKPLLDYFKTLYVWLVAENKNNN-------------RRVGWEKNIDPYSA 630
Darter_fish                   YTISGDFKMNARPLLDYFQPLHDWLKAENKKNN-------------RTVGWKTAVDPYSK 620
Frog                          ESITGGTKMDSQPLLKYFEPLFVWLQKNNKDNG-------------RKSTWNTAWTPFSE 619
Bat                           EQIAGTKEMDAKPLLNYFEPLFSWLKEQNGNSV----------------GWSADWSPYSE 614
Bat_Little_Brown              DKIVGTKKMDAKPLLNYFEPLFTWLKEQNGNSVGWNSGNSVESRSGNSVGWFSDWSPYAE 629
Elephant                      ESIVGAKNMDVRPLLNYFEPLFTWLKEQNRNSS---------------VGWSTDWTPYAA 610
Seal_low_qual                 YNVVGAKNMDVRPLLNYFDPLFTWLKEQNRNSF---------------VGWNTDWSPYAD 582
Mink                          ERVVGAKTMDVRPLLNYFEPLFTWLKEQNRNSF---------------VGWNTDWSPYAD 297
Ferret                        ERVVGAKTMDVRPLLNYFEPLFTWLKEQNRNSF---------------VGWNTDWSPYAD 615
Dog_X1                        EIVVGAKNMDVRPLLNYFEPLFTWLKEQNRNSF---------------VGWNTDWSPYAD 614
Bear                          EHVVGAKNMDVRPLLNYFEPLFTWLKEQNRNSF---------------VGWNTDWSPYAD 615
Cat                           EHVVGEKKMNVTPLLKYFEPLFTWLKEQNRNSF---------------VGWNTDWRPYAD 615
Tiger                         EHVVGEKNMNVTPLLKYFEPLFTWLKEQNRNSF---------------VGWNTDWRPYAD 607
Lemur                         ENVVGARNMNVTPLLNYFEPLFTWLKDQNRNSF---------------VGWNTNWSPYAD 641
Rat                           ENVVGSRNMDVKPLLNYFQPLFVWLKEQNSNST---------------VGWNTDWSPCDF 615
Mouse                         ENVVGARNMDVKPLLNYFQPLFDWLKEQNRNSF---------------VGWNTEWSPYAD 615
Squirrel                      ENVVGARNMDVRPLLNYFEPLFGWLKDQNRNSF---------------VGWNTNWSPYTD 625
Rabbit                        ENVVGAKNMDVRPLLNYFEPLFTWLKEQNRNSF---------------VGWSTEWTPYAD 615
Macaque                       ENVVGAKNMNVRPLLNYFEPLFTWLKDQNKNSF---------------VGWSTDWSPYAD 615
Human                         ENVVGAKNMNVRPLLNYFEPLFTWLKDQNKNSF---------------VGWSTDWSPYAD 615
Gorilla                       ENVVGAKNMNVRPLLNYFEPLFTWLKDQNKNSF---------------VGWSTDWSPYAD 615
Horse                         ERIVGVKNMDVRPLLNYFEPLFTWLKDQNKNSF---------------VGWSTNWSPYAD 615
Camel                         EGLVGVKTMDVKPLLNYFEPLLTWLKDQNRNSF---------------VGWSTDWTPYTD 615
Pig_X1                        ENIVGVKTMDVKPLLSYFEPLLTWLKAQNGNSS---------------VGWNTDWTPYAD 615
Beluga                        ESIAGVKTMDVKPLLNYFEPLLTWLKDQNRNSF---------------VGWNTDWTPYSD 614
Dolphin                       ERIVGVKTMDVKPLLNYFEPLLTWLKGQNRNSF---------------VGWRTDWTPYSN 614
Cow_X1                        ENIVGIKTMDVKPLLNYFEPLFTWLKEQNRNSF---------------VGWSTEWTPYSD 614
Goat                          ENIVGIKTMDVKPLLNYFEPLFTWLKEQNRNSF---------------VGWSTEWTPYSD 614
Sheep                         ENIVGIKTMDVKPLLNYFEPLFTWLKEQNRNSF---------------VGWSTEWTPYSD 614
Tiger_Snake_low_qual          ESITGSQKMDAKPFCQYFDPLLKWLEKANSNEN---------------VGWNVNWTPYSK 637
Duck                          ESLTGEKYMNATPLLHYFEPLFNWLQKNNSGRY---------------IGWNTDWTPYSE 615
Chicken                       ESATGEKYMNATPLLHYFEPLFNWLQKNNSGRS---------------IGWNTDWTPYSD 615
Rock-Dove_low_qual            EIATGEKYMNAAPLLHYFEPLYEWLQKNNSGRY---------------IGWKTDWAPYSD 605
Rock_Dove_short               EIATGEKYMNAAPLLHYFEPLYEWLQKNNSGRY---------------IGWKTDWAPYSD 479
                                  *   *:  *:  **. *  **   *                      *     *
```

```
Cod                     -NGIKVRISLKAALGADAYQW--------------------NDNELYLFKTNIAYAMRQY 669
Darter_fish             -YAIKVRISLKAALGDNAYSW--------------------DANMLYLFKANIAYALRHY 659
Frog                    AESIKVRISLKSGLGDNAYEW--------------------NANELYLFKSSIAYAMSKY 659
Bat                     -QSIKVRISLISALGEKAYEW--------------------NDNEMYLFRSSVAYAMRVY 653
Bat_Little_Brown        -QSIKVRISLQSALGEKAYKW--------------------NDNEMYLFQSSVAYAMREY 668
Elephant                -QYIKVRISLKTALGDKAYEW--------------------NDNEMYLFRSSIAYAMREY 649
Seal_low_qual           -XSIKVRISLNRS-XEKAYEW--------------------NDNEMYLFRSSIAYAMREY 620
Mink                    -QSIKVRISLKSALGEKAYEW--------------------NDNEMYFFQSSIAYAMREY 336
Ferret                  -QSIKVRISLKSALGEKAYEW--------------------NDNEMYFFQSSIAYAMREY 654
Dog_X1                  -QSIKVRISLKSALGEKAYEW--------------------NNNEMYLFRSSIAYAMRQY 653
Bear                    -QSIKVRISLKSALGEKAYEW--------------------NDNEMYLFRSSIAYAMRKY 654
Cat                     -QSIKVRISLKSALGDEAYEW--------------------NDNEMYLFRSSVAYAMREY 654
Tiger                   -QSIKVRISLKSALGDKAYEW--------------------NDNEMYLFRSSVAYAMREY 646
Lemur                   -ESIKVRISLKSALGTNAVSVFSLFFQLTIFFTCRPNKHNGMTKELYLFPVICGICMRKV 700
Rat                     -------------TDCTHYEW--------------------TDNEMYLFRSSVAYAMREY 642
Mouse                   -QSIKVRISLKSALGANAYEW--------------------TNNEMFLFRSSVAYAMRKY 654
Squirrel                -QSIKVRISLKSALGEEAYQW--------------------NDNEMYLFRSSVAYAMRMY 664
Rabbit                  -QSIKVRISLKTALGDQAYEW--------------------NDSEMYLFRSSVAYAMRKY 654
Macaque                 -QSIKVRISLKSALGDKAYEW--------------------NDNEMYLFRSSVAYAMRTY 654
Human                   -QSIKVRISLKSALGDRAYEW--------------------NDNEMYLFRSSVAYAMRQY 654
Gorilla                 -QSIKVRISLKSALGDKAYEW--------------------NDNEMYLFRSSVAYAMRQY 654
Horse                   -QSIKVRISLKSALGEKSYEW--------------------NDNEMYLFQSSVAYAMRVY 654
Camel                   -QSIKVRISLKSALGDKAYEW--------------------NDNEMYLFQSSLAYAMRKY 654
Pig_X1                  -QSIKVRISLKSALGKEAYEW--------------------NDNEMYLFRSSIAYAMRNY 654
Beluga                  -QSIKVRISLKSALGEKAYEW--------------------NDNEMYLFRSSVAYAMREY 653
Dolphin                 -QSIKVRISLKSALGEKAYEW--------------------NDNEMYLFRSSVAYAMREY 653
Cow_X1                  -QSIKVRISLKSALGENAYEW--------------------NDNEMYLFQSSVAYAMRKY 653
Goat                    -QSIKVRISLKSALGENAYEW--------------------NDNEMYLFRSSVAYAMRKY 653
Sheep                   -QSIKVRISLKSALGENAYEW--------------------NDNEMYLFRSSVAYAMRKY 653
Tiger_Snake_low_qual    -DAIKVRISLKTALGDDAYNW--------------------DESEMYLFKSTIAYAMQKY 676
Duck                    -NAIKVRISLKAA--GQTYEW--------------------NKSELFLFKSTIAYAMRTY 652
Chicken                 -NAIKVRISLKAALGDDAYVW--------------------DASELFLFKSSIAYAMRKY 654
Rock-Dove_low_qual      -NAIKVRISLKSALGAQAYEW--------------------NENELFFFKSSVAYAMRKY 644
Rock_Dove_short         -NAIKVRISLKSALGAQAYEW--------------------NENELFFFKSSVAYAMRKY 518
                                                                   . :::*    . .:


Cod                     YTSQMNKTTDFTAKDVWTYEETPRISFFIQVSDPSSPSVLIRRDEVEAAIRLSRGRINDA 729
Darter_fish             YS-QKNKTLQFTPEDVLVYDETPRISFYIVVTNPSTPTEYIPKDDVEAAIRLSRGRINQA 718
Frog                    YSVVMKQDIPFTYENVHVYDETPRVSFMFYVTISNN--TVVSKLEVENAVRLSKDRINSI 717
Bat                     FSK-MNKTIPFRAEDVRVSDEKKRVSFKFFVTSPTNISDIIPRSEVEDAIRMSRSRINDA 712
Bat_Little_Brown        FLKEKNQTIPFGVENVRVNDIKPRVSFKFYVTSPKNMSVVIPRSEVEDAIRMSRSRINDA 728
Elephant                FSKVKNQTILFGEDDVWVSDLKPRISFNFFVTVPKNASDIIPKAEVEEAIRMSRGRINDA 709
Seal_low_qual           FSKVKNQTIPFVEDNVRVNDLKPRISFTFFVTLPGNVSGIIPRADVEEAIRMSRGRINDA 680
Mink                    FSKVKKQTIPFVDKDVRVSDLKPRISFNFIVTSPENMSDIIPRADVEEAIRKSRGRINDA 396
Ferret                  FSKVKNQTIPFVGKDVRVSDLKPRISFNFIVTSPENMSDIIPRADVEEAIRKSRGRINDA 714
Dog_X1                  FSEVKNQTIPFVEDNVWVSDLKPRISFNFFVTSPGNVSDIIPRTEVEEAIRMYRSRINDV 713
Bear                    FSEAKNQMIPFVEDNVWVNDLKPRISFNFFVTSPGNVSDVIPRADVEGAIKMSRDRINDA 714
Cat                     FSKVKNQTIPFVEDNVWVSNLKPRISFNFFVTASKNVSDVIPRSEVEEAIRMSRSRINDA 714
Tiger                   FSKVKNQTIPFVEDNVWVSNLKPRISFNFFVTASKNVSDVIPRREVEEAIRMSRSRINDA 706
Lemur                   FLEAKSQTVPFGVEDVWVNDFKPRISFTFFVTAPKNVSDIIPRTEVEKAISMSRSRINDV 760
Rat                     FSREKNQTVPFGEADVWVSDLKPRVSFNFFVTSPKNVSDIIPRSEVEEAIRMSRGRINDI 702
Mouse                   FSIIKNQTVPFLEEDVRVSDLKPRVSFYFFVTSPQNVSDVIPRSEVEDAIRMSRGRINDV 714
Squirrel                FSKVKNQTIPFGEKDVWVSDEKPRISFNFFVTAPQNVSDIIPRTDVEKAIRMSRGRINGV 724
Rabbit                  FSEVKNQTILFGEEDVRVSDLKPRISFNFFVTAPNNVNDIIPRNEVEEAISMSRSRINDI 714
Macaque                 FLEIKHQTILFGEEDVRVADLKPRISFNFYVTAPKNVSDIIPRTEVEEAIRISRSRINDA 714
Human                   FLKVKNQMILFGEEDVRVANLKPRISFNFFVTAPKNVSDIIPRTEVEKAIRMSRSRINDA 714
Gorilla                 FLEVKKQMILFGEEDVRVANLKPRISFNFFVTAPKNVSDIIPRTEVEKAIRMSRGRINDA 714
Horse                   FLKAKNQTILFGEEDVWVSDLKPRISFNFFVTSPKNASDIIPRTDVEEAIRMSRSRINDA 714
Camel                   FLKVQNQTILFGVEDVWVSDLKPRISFSFFVTSPKNVSDIIPRTEVEEAIRMSRSRINDA 714
Pig_X1                  FSSAKNETIPFGAEDVWVSDLKPRISFNFFVTSPANMSDIIPRSDVEKAISMSRSRINDA 714
Beluga                  FSKVRNETIPFGGEDVRVSDLKPRISFNFFVTSPKNMSDIIPRTEVEEAIRMSRGRINDA 713
Dolphin                 FSKVRNKTIPFGEKDVWVSDLKPRISFNFFVTSPKNMSDIIPRTEVEEAIRMSRGRINDA 713
Cow_X1                  FSEARNETVLFGEDNVWVSDKKPRISFKFFVTSPNNVSDIIPRTEVENAIRLSRDRINDV 713
Goat                    FLEDRNETIPFGEENVWVSDKKPRISFKFFVTSPNNVSDIIPRTEVENAIRLCRDRINDA 713
Sheep                   FLKERNETIPFGEENVWVSDKKPRISFKFFVTSPNNVSDIIPRTEVENAIRLCRDRINDA 713
Tiger_Snake_low_qual    FLEVKNKTVLFQTDNVHVSDMTVRISFYFTVSMPNNISELVPKSEVEEAISLSRDRINEA 736
Duck                    FAQK-QQLIDFEATDVHVSEETQRVSFYITVSMPGNASNIVPKADVESAISMSRGRINEA 711
Chicken                 FAKEKEQNVDFQVTDIHVGEETQRVSFYLTVSMPGNVSDIVPRADVEKAIRMSRGRISEA 714
Rock-Dove_low_qual      FADKKKQEVDFQITDIHVGEQTQRISFYLTVSMPGNISDIVPKADVENAIRMSRGRINEA 704
Rock_Dove_short         FADKKKQEVDFQITDIHVGEQTQRISFYLTVSMPGNISDIVPKADVENAIRMSRGRINEA 578
                        :     :   *   :: . : . *:** : *:   .    : : :** *:   :.**.
```

```
Cod                     FMLNDKTLEFQGIPATLAPPVEQPVTVWLVVYGVVMGIVVLVGAYLIISGIRARKTQQP- 788
Darter_fish             FQLDDKTLEFVGIPATLAPPVEQPVTVWLVVFGVVMGVVVLAGIYLVISGVKERKKSAN 778
Frog                    FKLDDNALEFVGIPPTLAAPAEQPVTVWLVVFGIVAGIIVVALIVLIVLGHKDRQKKRKS 777
Bat                     FRLDDNTLEFLGVQPTLGPPYQPPVSIWLIIFGVVMGVVVIGIGVLIFTGIRDRKKKNQA 772
Bat_Little_Brown        FRLDDNTLEFLGIQPTLGPPNQPPVTIWLIVFGVVMGVAVIGIAVLIFTGIRDRKKKKQA 788
Elephant                FRLDDKTLEFVGIQPTLAPPNQPPVVVWLVVFGVVMGVVVIGIVLLVISGIRDRRKKTQA 769
Seal_low_qual           FRLDDKSLEFLGIQPTLGPPYQPPVTIWLIVFGVVMGVVVVGIVLLIFSGIRNRRKNDQA 740
Mink                    FRLDDNSLEFLGIQPTLEPPYQPPVTIWLIVFGVVMGVVVVGIFLLIFSGIRNRRKNNQA 456
Ferret                  FRLDDNSLEFLGIQPTLEPPYQPPVTIWLIVFGVVMGVVVVGIFLLIFSGIRNRRKNNQA 774
Dog_X1                  FRLDDNSLEFLGIQPTLGPPYEPPVTIWLIVFGVVMGVVVVGIVLLIFSGIRNRRKNDQA 773
Bear                    FQLDDNSLEFLGIHPTLGPPYQPPVTIWLIVFGVVMGLVVIGIILLIFSGIRNRRKNDQA 774
Cat                     FRLDDNSLEFLGIQPTLSPPYQPPVTIWLIVFGVVMGVVVVGIVLLIVSGIRNRRKNNQA 774
Tiger                   FRLDDNSLEFLGIQPTLSPPYQPPVTIWLIVFGVVMGVVVVGIVLLIVSGIRNRRKNNQA 766
Lemur                   FGLDDNTLEFLGIQPTLSPPYESPISIWLIAFGIVMGLVVVATVVLIFTGIRNRKKKSEE 820
Rat                     FGLNDNSLEFLGIYPTLKPPYEPPVTIWLIIFGVVMGMVVVGIVILIVTGIKGRKKKNET 762
Mouse                   FGLNDNSLEFLGIHPTLEPPYQPPVTIWLIIFGVVMALVVVGIIILIVTGIKGRKKKNET 774
Squirrel                FRLDDNSLEFLGIQPTLGPPYQPPVTIWLIVFGVVMGLIVVGIVILIFTGIRDRRRKNQT 784
Rabbit                  FRLDDNSLEFVGIQPTLEPPYESPVPIWLVVFGVVMGMIVIGIVVLIFTGIKDRRKQKQA 774
Macaque                 FRLNDNSLEFLGIQTTLAPPYQSPVTTWLIVFGVVMGVIVAGIVVLIFTGIRDRKKKNQA 774
Human                   FRLNDNSLEFLGIQPTLGPPNQPPVSIWLIVFGVVMGVIVVGIVILIFTGIRDRKKKNKA 774
Gorilla                 FRLNDNSLEFLGIQPTLGPPNQPPVSIWLIVFGVVMGVIVVGIVILIFTGIRDRKKKNKA 774
Horse                   FRLDDNTLEFLGIQPTLGPPYQPPVTVWLIAFGVVMGLVVVGIVVLIATGIRGRRKKNQA 774
Camel                   FRLDDNSLEFLGIQPTLGPPYEPPVTVWLIIFGIVMGLVVVGIVVLIFTGIRDRRKKKQA 774
Pig_X1                  FRLDDNTLEFLGIQPTLGPPDEPPVTVWLIIFGVVMGLVVVGIVVLIFTGIRDRRKKKQA 774
Beluga                  FRLDDSSLEFLGVQPTLAPPYEPPVTVWLIIFGVVMGVVVIGIVVLIFTGIRGRRKKNQA 773
Dolphin                 FRLDDSSLEFLGVQPTLAPPYEPPVTVWLIIFGVVMGVVVIGIVVLIFTGIRDRRKKNQA 773
Cow_X1                  FQLDDNSLEFLGIQPTLGPPYEPPVTIWLIIFGVVMGVVVIGIVVLIFTGIRNRRKHDND 773
Goat                    FQLDDNSLEFLGIQPTLRPPYEPPVTIWLIIFGVVMGVVVIGIVVLIFTGIRDQRKKNQA 773
Sheep                   FQLDDNSLEFLGIQPTLRPPYEPPVTIWLIIFGVVMGVVVIGIVVLIFTGIRDQRKKNQA 773
Tiger_Snake_low_qual    FKLTDQTLEFVGLLPTLAPPYESPITVWLIAFGVVIGLVVIGIITLLIIGQKDRKKKKRA 796
Duck                    FGLDDDTLEFVGIIPTLAAPYEPPVTIWLIIFGVVISLVVIGVIVLIVSGQRDRKKKAKG 771
Chicken                 FRLDDNTLEFDGIVPTLATPYKPPVTIWLILFGVVMSLIVIGVIVLIITGQRDRKKKARG 774
Rock-Dove_low_qual      FRLDDNTLEFVGILPTLATPYEPPVTIWLIVFGVVISLVVVGVIVLIITGQRDRKKRARE 764
Rock_Dove_short         FRLDDNTLEFVGILPTLATPYEPPVTIWLIVFGVVISLVVVGVIVLIITGQRDRKKRARE 638
                        * * *.:*** *:  **  * : *:  **: :*:* .: *     *:  * : :: .

Cod                     ESKVENPYTPQ---------------------------ATGLTNLSYQTDEA-------- 813
Darter_fish             RGSTENPYDTH---------------------------IDGKTNKAFEVTDD-------- 803
Frog                    -EGDKEAMALSQ---TTNNPEPATCEMTQENTSQMNKAFDGNDNKAFDGNDNKAFDGNDN 833
Bat                     -ENEENPYSSVNF-------------------------SKGENNPGFQSGDD-------- 798
Bat_Little_Brown        -GNEENPYSSVNL-------------------------SKGENNPGFQNGDD-------- 814
Elephant                -SCEENPYASVDL-------------------------SKGENNPGYQSTDD-------- 795
Seal_low_qual           -RSEENPYASVNL-------------------------SKGETNPGFQNVGA-------- 766
Mink                    -RSEENPYASVDL-------------------------SKG------------------- 471
Ferret                  -RSEENPYASVDL-------------------------SKGENNPGFQNVDD-------- 800
Dog_X1                  -RGEENPYASVDL-------------------------SKGENNPGFQNVDD-------- 799
Bear                    -RSEENPYASVDL-------------------------SKGENNPGFQNADD-------- 800
Cat                     -RSEENPYASVDL-------------------------SKGENNPGFQHADD-------- 800
Tiger                   -RSEENPYASVDL-------------------------SKGENNPGFQHADD-------- 792
Lemur                   -RSEENPYASMDI-------------------------NRGEHNPGFQNSDD-------- 846
Rat                     -KREENPYDSVDI-------------------------GKGESNAGFQNSDD-------- 788
Mouse                   -KREENPYDSMDI-------------------------GKGESNAGFQNSDD-------- 800
Squirrel                -KREENPYAESSM-------EM----------------GKGENNPGYQNNDD-------- 812
Rabbit                  -KREENPYGFVDM-------------------------SKGENNSGFQNSDD-------- 800
Macaque                 -RSEENPYASIDI-------------------------NKGENNPGFQNTDD-------- 800
Human                   -RSGENPYASIDI-------------------------SKGENNPGFQNTDD-------- 800
Gorilla                 -RSEENPYASIDI-------------------------SKGENNPGFQNTDD-------- 800
Horse                   -RSEENPYASVDL-------------------------SKGENNPGFQNGDD-------- 800
Camel                   -STEENPYGSVDL-------------------------SKGENNSGFQNGDD-------- 800
Pig_X1                  -SSEENPYGSMDL-------------------------SKGESNSGFQNGDD-------- 800
Beluga                  -SSEENPYDSMGL-------SK------------------GENNSGFQNSDD-------- 799
Dolphin                 -SSEENPYGSVGL-------SK------------------GENNPGFQNSDD-------- 799
Cow_X1                  -GLQNDENLRVQQQAVKVDISR----------------NSLKAPVPFSNSHE-------- 808
Goat                    -SSEENPYGSV-------DLNK----------------GE--NNSGFQNTDD-------- 799
Sheep                   -SSEENPYGSV-------DLNK----------------GE--NNSGFQNTDD-------- 799
Tiger_Snake_low_qual    -AKTNS---M-----ETAAIHE----------------DCGQSNSTFQLDEA-------- 823
Duck                    -RERETESNC-----EV-NPYD----------------DDGRSNKGFELSDE-------- 800
Chicken                 -RANEAGSNC-----EV-NPYD----------------EDGRSNKGFEQSEE-------- 803
Rock-Dove_low_qual      -SRSEVGSNC-----EVANPYN----------------EEGKSNLGFEPSEE-------- 794
Rock_Dove_short         -SRSEVGSNC-----EVANPYN----------------EEGKSNLGFEPSEE-------- 668
                            :
```

Cod	---------------ENTGL	818
Darter_fish	---------------EQTGF	808
Frog	KAFDGNDNKASDEADMNTTF	853
Bat	---------------VQTSF	803
Bat_Little_Brown	---------------VQTSF	819
Elephant	---------------VQTSF	800
Seal_low_qual	---------------VQTSF	771
Mink	--------------------	471
Ferret	---------------VQTSF	805
Dog_X1	---------------AQTSF	804
Bear	---------------VQTSF	805
Cat	---------------VQTSF	805
Tiger	---------------VQTSF	797
Lemur	---------------TQTSF	851
Rat	---------------AQTSF	793
Mouse	---------------AQTSF	805
Squirrel	---------------VQTSF	817
Rabbit	---------------IQTSF	805
Macaque	---------------VQTSF	805
Human	---------------VQTSF	805
Gorilla	---------------VQTSF	805
Horse	---------------VQTSF	805
Camel	---------------VQTSF	805
Pig_X1	---------------IQTSF	805
Beluga	---------------VQTSF	804
Dolphin	---------------VQTSF	804
Cow_X1	---------------KLK--	811
Goat	---------------VQTSL	804
Sheep	---------------VQTSL	804
Tiger_Snake_low_qual	---------------ATTTF	828
Duck	---------------TQTSF	805
Chicken	---------------TQTSF	808
Rock-Dove_low_qual	---------------TQTPF	799
Rock_Dove_short	---------------TQTPF	673

Figure S1. Amino acid sequence alignment of ACE2 polypeptides from a range of animal species. Accession Numbers of the polypeptide sequences used are given in Table 1. Alignments were carried out *using Clustal Omega* [1]. The last row shown is the consensus analysis given by the algorithm. Human sequence is highlighted in grey to aid comparison to other sequences. Important amino acids used for interaction with the SARS-CoV-2 viral proteins as reported by Damas et al. [2] are highlighted in yellow, with the highlighted glycosylated residues in blue. The extra residues of importance reported by Sun et al. [3] are in purple. The region reported to be important for ACE2 cleavage is highlighted in red in the human sequence, with the R or K residues being coloured Blue: changes from R or K are coloured brown.

```
CLUSTAL O(1.2.4) multiple sequence alignment


Darter_fish          ------------------------------------------------------------    0
Trout_X1             ------------------------------------------------------------    0
Frog_V1              MEPAVYVIPDNNLEQPPVYDVVSNTQVQPAPLPYNTQVHSNQFVAASPISPFQWSFRKKV    60
Duck                 ------------------------------------------------------------    0
Chicken_X1           ------------------------------------------------------------    0
Rock_dove            ------------------------------------------------------------    0
Tiger_snake_X1       ------------------------------------------------------------    0
Camel                ------------------------------------------------------------    0
Goat                 ------------------------------------------------------------    0
Cow                  ------------------------------------------------------------    0
Sheep                ------------------------------------------------------------    0
Beluga_X1            ------------------------------------------------------------    0
Dolphin              ------------------------------------------------------------    0
Macaque              ------------------------------------------------------------    0
Human_V2             ------------------------------------------------------------    0
Human_V1             -------------------------------MPP--------------------------    3
Gorilla_X1           ------------------------------------------------------------    0
Gorilla_X2           ------------------------------------------------------------    0
Mouse                ------------------------------------------------------------    0
Rat                  ------------------------------------------------------------    0
Elephant             ------------------------------------------------------------    0
Dog                  ------------------------------------------------------------    0
Rabbit               ------------------------------------------------------------    0
Horse_X1             ------------------------------------------------------------    0
Horse_X2             ------------------------------------------------------------    0
Lemur                -------------------------------MPLNQQAKERA------------------    11
Ground_Squirrel      ------------------------------------------------------------    0
Pig                  ------------------------------------------------------------    0
Tiger                ------------------------------------------------------------    0
Grey_Seal_X1         ------------------------------------------------------------    0
Bear                 ------------------------------------------------------------    0
Ferret               ------------------------------------------------------------    0
Cat_X1               ------------------------------------------------------------    0
Cat_X2               ------------------------------------------------------------    0
Cat_X3               ------------------------------------------------------------    0
Bat                  ------------------------------------------------------------    0
Bat_Little_Brown     ------------------------------------------------------------    0
```

```
Darter_fish        ------------------------------------------------------------   0
Trout_X1           ------------------------------------------------------------   0
Frog_V1            LISIGTLSIVAAVIVASVLTGYFVSTTQSVPYCQKRCSYSSKCIYGYQICDGVQDCPYGD   120
Duck               ------------------------------------------------------------   0
Chicken_X1         ------------------------------------------------------------   0
Rock_dove          ------------------------------------------------------------   0
Tiger_snake_X1     ------------------------------------------------------------   0
Camel              ------------------------------------------------------------   0
Goat               ------------------------------------------------------------   0
Cow                ------------------------------------------------------------   0
Sheep              ------------------------------------------------------------   0
Beluga_X1          ------------------------------------------------------------   0
Dolphin            ------------------------------------------------------------   0
Macaque            ------------------------------------------------------------   0
Human_V2           ------------------------------------------------------------   0
Human_V1           ------------------------------------------------------APPGGE   9
Gorilla_X1         ------------------------------------------------------------   0
Gorilla_X2         ------------------------------------------------------------   0
Mouse              ------------------------------------------------------------   0
Rat                ------------------------------------------------------------   0
Elephant           ------------------------------------------------------------   0
Dog                ------------------------------------------------------------   0
Rabbit             ------------------------------------------------------------   0
Horse_X1           ------------------------------------------------------------   0
Horse_X2           ------------------------------------------------------------   0
Lemur              -----------------------------------------------AAWAGVTDPAHRE   24
Ground_Squirrel    ------------------------------------------------------------   0
Pig                ------------------------------------------------------------   0
Tiger              ------------------------------------------------------------   0
Grey_Seal_X1       ------------------------------------------------------------   0
Bear               ------------------------------------------------------------   0
Ferret             -------------------------------------------------------MEEET   5
Cat_X1             ------------------------------------------------------------   0
Cat_X2             ------------------------------------------------------------   0
Cat_X3             ------------------------------------------------------------   0
Bat                ------------------------------------------------------------   0
Bat_Little_Brown   ------------------------------------------------------------   0
```

```
Darter_fish        -----------------------------------------MNR--NQVNGP---IYD--   12
Trout_X1           -----------------------------------------MNN--NQTPGP---QYV--   12
Frog_V1            DERNCATTCLYCSYSYTCIYYYQICNGVQDCPYGDDERNCATKTPSTPTCQMYCSYYYTC   180
Duck               -------------------------------------------MTSTVGPPP---YFE--   12
Chicken_X1         -------------------------------------------MTSTVNPPP---YYE--   12
Rock_dove          -------------------------------------------MTSTVGLPP---YYE--   12
Tiger_snake_X1     ----MIF--EPQKGVYSKLA----II-------QLSKIKMAS----NLRPTP---YYE--   34
Camel              ---------------------------------------MALNSGSPPGVGP---YYE--   16
Goat               ---------------------------------------MALNSGSSPGVGP---YYE--   16
Cow                ---------------------------------------MALNSGSSPGVGP---YYE--   16
Sheep              ---------------------------------------MALNSGSSPGVGP---YYE--   16
Beluga_X1          ------------------------------------------------------------   0
Dolphin            ---------------------------------------MALNSGSPPDVGP---YYE--   16
Macaque            ---------------------------------------MALNSGSPPGVGP---YYE--   16
Human_V2           ---------------------------------------MALNSGSPPAIGP---YYE--   16
Human_V1           SG--CEE--RGAAGHIEHSRYLSLLD-------AVDNSKMALNSGSPPAIGP---YYE--   53
Gorilla_X1         ---------------------------------------MALNSGSPPAIGP---YYE--   16
Gorilla_X2         ---------------------------------------MALNSGSPPAIGP---YYE--   16
Mouse              ---------------------------------------MALNSGSPPGIGP---CYE--   16
Rat                ---------------------------------------MALNSGSPPGIGP---YYE--   16
Elephant           ---------------------------------------MALDSGSPTGVGP---YYE--   16
Dog                ---------------------------------------MALNSGSPPGVGP---YYE--   16
Rabbit             ---------------------------------------MALNSGLSPGVGP---YYE--   16
Horse_X1           ---------------------------------------MALNSGSPPGVGP---YYE--   16
Horse_X2           ---------------------------------------MALNSGSPPGVGP---YYE--   16
Lemur              EA--RGP--LSCRGHLEHSKYLPLLD-------AVDKIRMALNSRSPPGAGP---YYE--   68
Ground_Squirrel    ---------------------------------------MALNSGPSPGVGP---YYE--   16
Pig                ---------------------------------------MALNSGSRPGVGP---YYE--   16
Tiger              ---------------------------------------MALNSGPPPGVGP---FYE--   16
Grey_Seal_X1       ---------------------------------------MALNSGSPPGVGP---YYE--   16
Bear               ---------------------------------------MALNSGSPPGDGP---YYE--   16
Ferret             GK--MLA--VPLPGHLERPESLPSPD-------AADNTGMALNAGSPPGVGP---YYE--   49
Cat_X1             ---------------------------------------MALNSGPPPGVGP---FYE--   16
Cat_X2             ---------------------------------------MALNSGPPPGVGP---FYE--   16
Cat_X3             ---------------------------------------MALNSGPPPGVGP---FYE--   16
Bat                ---------------------------------------MALNSGLPPSVAP---YYE--   16
Bat_Little_Brown   ------------------------------------------------------------   0
```

```
Darter_fish         NMGFQHEEGRP-------------------------------------------------    23
Trout_X1            NRGFQQGEGRP-------------------------------------------------    23
Frog_V1             IYGYQICNGVQDCPYGDDERNCATKTPSIPTCQMYCSYTSTCIYGYQICNGVQDCAYGDD    240
Duck                NHGFQPE----------------------------------N-YS---------------    22
Chicken_X1          NHGFQTE----------------------------------NYYS---------------    23
Rock_dove           NGGFQPE----------------------------------NVYS---------------    23
Tiger_snake_X1      NYAYQAE----------------------------------NVPP---------------    45
Camel               NHGYQPE----------------------------------SFYP---------------    27
Goat                NHGYQPE----------------------------------SLYP---------------    27
Cow                 NHGYQPE----------------------------------SLYP---------------    27
Sheep               NHGYQPE----------------------------------SLYP---------------    27
Beluga_X1           ------------------------------------------------------------    0
Dolphin             NHGFQPE----------------------------------SLYP---------------    27
Macaque             NHGYQPE----------------------------------NPYP---------------    27
Human_V2            NHGYQPE----------------------------------NPYP---------------    27
Human_V1            NHGYQPE----------------------------------NPYP---------------    64
Gorilla_X1          NHGYQPE----------------------------------NPYP---------------    27
Gorilla_X2          NHGYQPE----------------------------------NPYP---------------    27
Mouse               NHGYQSE----------------------------------HICP---------------    27
Rat                 NHGYQSE----------------------------------HVYS---------------    27
Elephant            NHGYQPE----------------------------------NFYP---------------    27
Dog                 NHGYQPE----------------------------------SLYP---------------    27
Rabbit              NHGYQPE----------------------------------NLYA---------------    27
Horse_X1            NHGYQPE----------------------------------SFYS---------------    27
Horse_X2            NHGYQPE----------------------------------SFYS---------------    27
Lemur               NHGYQPE----------------------------------NFYT---------------    79
Ground_Squirrel     NHGYQPE----------------------------------ALYP---------------    27
Pig                 NHGYQPE----------------------------------SVYP---------------    27
Tiger               NHGYQPE----------------------------------GLYP---------------    27
Grey_Seal_X1        NHGYQPE----------------------------------SLYP---------------    27
Bear                NHGYQPE----------------------------------SLYP---------------    27
Ferret              NHGYQPE----------------------------------SLYP---------------    60
Cat_X1              NHGYQPE----------------------------------GLYP---------------    27
Cat_X2              NHGYQPE----------------------------------GLYP---------------    27
Cat_X3              NHGYQPE----------------------------------GLYP---------------    27
Bat                 NHGYQPE----------------------------------NLYP---------------    27
Bat_Little_Brown    ------------------------------------------------------------    0
```

```
Darter_fish          -----HSNAPQQGLYPVLP-QETPSYVA-VGTKTINTHHTATPGT-----PHNIGQRKGI   71
Trout_X1             -----PPYASAPKMHNVPP-SICPQYAPQYAPKTINTHHTVTPGL-----PHPVPEQKAV   72
Frog_V1              ERNCATKTPSIPT-CQLYCSYYY-TCIYAYQ--IC-------NGV--LDCPFVDDERNCV   287
Duck                 -----ARPPAGANPYPPYFSTN-APSVPTYVPRVST--YQSTSPV----AP--STGRTCS   68
Chicken_X1           -----ARPQVGANPYPQYFSTN-VPSVPTYIPRVST--HQSSIPV----APPSSSSRMCS   71
Rock_dove            -----ARQPVGAYPYPQNFSTN-APSVPSYVPRVAT--YQSTRPV----AH--SSRRTCA   69
Tiger_snake_X1       ------RFTRDHNMYPHYTPTYYPSSVPHYVPKVST--QQSIPVPVLQT-----KSAKCT   92
Camel                -----LKPSAASSAYMVYPAQYYPPAVPQYTPRVQT--HTSTPVI--RMQPKPPSETVCT   78
Goat                 -----QQLPTAPPAYEVYPTQYYPPMVPQYTPRVQT--HASTPVI--YRQPKPPSRTACT   78
Cow                  -----QQPPTAPPAFEVYPAQYYPPVVPQYTPRVQT--HASTPVI--YRQPKPPSRTACT   78
Sheep                -----RQLPTAPPAYQVYPAQYYPPMVPQYTPRVQT--HASTPVI--YRQPKPPSRTACT   78
Beluga_X1            ---------MAHRAYAGYPAQYYPPAVPQYAPRVQT--HTSTPII--LMQPKPPSGPACT   47
Dolphin              -----PQPPMAHRAYAGYPAQYYPPAVPQYAPRVQT--HTSTPVI--LMQPKPPSGPACT   78
Macaque              -----AQPTVAPNVYEVHPAQYYPSPVPQYTPRVLT--HASNPAV--CRQPKSPSGTVCT   78
Human_V2             -----AQPTVVPTVYEVHPAQYYPSPVPQYAPRVLT--QASNPVV--CTQPKSPSGTVCT   78
Human_V1             -----AQPTVVPTVYEVHPAQYYPSPVPQYAPRVLT--QASNPVV--CTQPKSPSGTVCT   115
Gorilla_X1           -----AQPTVAPTVYEVHPAQYYPSPVPQYAPRVLT--QASNPVV--RTQPKSPSGTVCT   78
Gorilla_X2           -----AQPTVAPTVYEVHPAQYYPSPVPQYAPRVLT--QASNPVV--RTQPKSPSGTVCT   78
Mouse                -----PRPPVAPNGYNLYPAQYYPSPVPQYAPRITT--QASTSVI--HTHPKS-SGALCT   77
Rat                  -----PRPPVSPNGCNLYPAQSHPSPVPQYAPRVTT--QASTPAI--HIQPRS-SGTLCT   77
Elephant             -----LQPPAAPGAYPAYPAQYYPSPVPQYVPTVLT--HASTPAI--HQQPNPPSGTVRT   78
Dog                  -----PQPATAPRAYPVYPAPYYPPAVPQYTPRVLT--HTSTPAI--HTQPKSPLGTGCT   78
Rabbit               -----RQPPVAPHVYTVYPAQYYPSPVPQYTPRVVT--QAATPAV--RRQPRSGSGTSCT   78
Horse_X1             -----RQPPVAPGAYGAYEAEYYPPTVPRYSPRVLT--QASTPVN--HTQPKLLSETVCT   78
Horse_X2             -----RQPPVAPGAYGAYEAEYYPPTVPRYSPRVLT--QASTPVN--HTQPKLLSETVCT   78
Lemur                -----RQPAGAPGVYPAHAAQYYPSPVPQYAPRVLT--NDSTPTI--RTRPKSPTGAACS   130
Ground_Squirrel      -----SQPAVAPNVYAAYPAQYCPSPVPQYTSRVPT--HTSTPGI--YRQPKPPPGTVCT   78
Pig                  -----PQPPGAHRPYGAYPAQYHPPSVPQYAPRVQT--HASTPAVVVSRQPKPRSRTMCS   80
Tiger                -----PRPAVVPSAYSVYPAPYYPPAVPQYAPRVLT--HTSTPSS--HTQPKSPSGTVCT   78
Grey_Seal_X1         -----LRPAMVPSAYPVYPVPYYPPEGPQSTPRVLT--HTSTPAI--RVQPKSPSGTACT   78
Bear                 -----ARPAAVPSAYPVYPAPYYPPVVPQYTPRVPT--HTSTPAV--RVQPKSPSGTTCT   78
Ferret               -----APPATVPSVYVAYPAAYYPPAVPQYTPRVLT--QASTPAV--RTQPKSPSGTACT   111
Cat_X1               -----PRPAVVPGAYSVYPAPYYPPAVPQYAPRVLT--HASTPSS--HTQPKSPSGTVCT   78
Cat_X2               -----PRPAVVPGAYSVYPAPYYPPAVPQYAPRVLT--HASTPSS--HTQPKSPSGTVCT   78
Cat_X3               -----PRPAVVPGAYSVYPAPYYPPAVPQYAPRVLT--HASTPSS--HTQPKSPSGTVCT   78
Bat                  -----PRPPVDPSAYTVYPAPYYPPTVPQYTPRVLT--NTSTPAV--RTQPKSPSRRLCT   78
Bat_Little_Brown     ------------------------------------------------------------   0
```

```
Darter_fish         -KKRQWKYVLCASLCVL-------LILAVVSLLLWYFLY---------------------   102
Trout_X1            -KKCHCKCIVASILSVL-------VFLGATGALVWYFLS---------------------   103
Frog_V1             ---------IAT------------TSTPTCQIYCWDFMFDYTCIYAYQMCDGVRQCYYGD   326
Duck                SGVKKTIIIIISIVIVICC------AVGAF--FIWYFVE---------------------   99
Chicken_X1          SSIKKIVIITLSILLVICC------AIAAF--LIWYFVE---------------------   102
Rock_dove           PSAKKTIVIILSILLVICC------AVAAF--FIWYFXE---------------------   100
Tiger_snake_X1      PRTKKALYVLSI-TVLIVG-----AGLSAF--LIWYFVT---------------------   123
Camel               SKTKKVLCVTLGLGAILVG-----AVLAAV--LLWKFKE---------------------   110
Goat                SKTKKALCITFTLAALLAG-----AILAAV--LLWKYME---------------------   110
Cow                 SKTKKALCITFTLVVLLAG-----AILAAV--LLWKYME---------------------   110
Sheep               SKTKKALCITFTLAALLTG-----AILAAV--LLWKYME---------------------   110
Beluga_X1           SRTKKVLCVTFSLGALLAG-----AVLAAM--LLWKLME---------------------   79
Dolphin             SRTKKVLCVTFSLGALLAG-----AVLAAM--LLWKLME---------------------   110
Macaque             SKTKKALCVTMTLGAVLVGA-----AL--AAGLLWKFMG---------------------   110
Human_V2            SKTKKALCITLTLGTFLVGA-----AL--AAGLLWKFMG---------------------   110
Human_V1            SKTKKALCITLTLGTFLVGA-----AL--AAGLLWKFMG---------------------   147
Gorilla_X1          SKTKKALCITLTLGTFLVGA-----AL--AAGLLWKFMG---------------------   110
Gorilla_X2          SKTKKALCITLTLGTFLVGA-----AL--AAGLLWKFMG---------------------   110
Mouse               SKSKKSLCLALALGTVLTG-----AAVAAV--LLWRFWD---------------------   109
Rat                 SKSKKSMFVALALGTVLAG-----AAVAAG--LLWKFRD---------------------   109
Elephant            SRTKRALCVTFALGAILAA-----VVLAAI--LLWRFLD---------------------   110
Dog                 AKTKKALCITVVLGTILAG-----AAVAAV--LLWKFME---------------------   110
Rabbit              PKAKKALCITFALGAVLVGA-------GLVIGLLWNFLE---------------------   110
Horse_X1            LKTKKVLCILLSLGLLLVV-----LVVAAI--LLWKFVE---------------------   110
Horse_X2            LKTKKVLCILLSLGLLLVV-----LVVAAI--LLWKFVE---------------------   110
Lemur               SKTKKGLCIALALGAVLVG-----GALAA--GLVWKFMA---------------------   162
Ground_Squirrel     TRARKVLCVTLALGTFLVV-----TVIAAV--LVWKFLE---------------------   110
Pig                 SKTKKALCITFALGAILAG-----AVLATV--LLWKFME---------------------   112
Tiger               SKAKKVLCITFTLGAVLAG-----AVLAAV--LLWKFME---------------------   110
Grey_Seal_X1        AKAKKALCITVSLGAVLAG-----AMVAAV--LLWKFME---------------------   110
Bear                AKAKRALCITVALGAVLAG-----AAVAAV--LLWRFME---------------------   110
Ferret              AS-RTLFCISFLQQNQRWGPSSLGALSPTVLPLCWGGSQ---------------------   149
Cat_X1              SKAKKVLCITFTLGAVLA-----GAVLAAV--LLWKFME---------------------   110
Cat_X2              SKAKKVLCITFTLGAVLA-----GAVLAAV--LLWKFME---------------------   110
Cat_X3              SKAKKVLCITFTLGAVLA-----GAVLAAV--LLWKFME---------------------   110
Bat                 SKTKKVLCIAVALLAVLA-----GAVAAAL--LLWRFTE---------------------   110
Bat_Little_Brown    ------------------------------------------------------------   0
```

```
Darter_fish          --YQCLL-----------------------------------------------------   107
Trout_X1             --SQCVL-----------------------------------------------------   108
Frog_V1              DERNCVTATTTTATTTSPPT--------CQIYCMNFMYYYTCIYAYQMCDGVRQCYYGDD   378
Duck                 --NPCLG-----------------------------------------------------   104
Chicken_X1           --NRCLG-----------------------------------------------------   107
Rock_dove            --NRCLG-----------------------------------------------------   105
Tiger_snake_X1       --DSCFG-----------------------------------------------------   128
Camel                --SRCSA-----------------------------------------------------   115
Goat                 --DGCS------------------------------------------------------   114
Cow                  --DECS------------------------------------------------------   114
Sheep                --DGCS------------------------------------------------------   114
Beluga_X1            --DECL------------------------------------------------------   83
Dolphin              --DECL------------------------------------------------------   114
Macaque              --SKCSD-----------------------------------------------------   115
Human_V2             --SKCSN-----------------------------------------------------   115
Human_V1             --SKCSN-----------------------------------------------------   152
Gorilla_X1           --SKCSD-----------------------------------------------------   115
Gorilla_X2           --SKCSD-----------------------------------------------------   115
Mouse                --SNCST-----------------------------------------------------   114
Rat                  --SKCSS-----------------------------------------------------   114
Elephant             ---KCSG-----------------------------------------------------   114
Dog                  --DKCSV-----------------------------------------------------   115
Rabbit               --GRC-------------------------------------------------------   113
Horse_X1             --NKCMG-----------------------------------------------------   115
Horse_X2             --NKCMG-----------------------------------------------------   115
Lemur                --SRCVG-----------------------------------------------------   167
Ground_Squirrel      --NKCSA-----------------------------------------------------   115
Pig                  --KKRCS-----------------------------------------------------   117
Tiger                --NKCLV-----------------------------------------------------   115
Grey_Seal_X1         --NKCLV-----------------------------------------------------   115
Bear                 --SKCSV-----------------------------------------------------   115
Ferret               --APPLSAVVTTVTALSPPARGRGERPGCRALPLLSL-----------------------   184
Cat_X1               --NKCLV-----------------------------------------------------   115
Cat_X2               --NKCLV-----------------------------------------------------   115
Cat_X3               --NKCLV-----------------------------------------------------   115
Bat                  --NRCLV-----------------------------------------------------   115
Bat_Little_Brown     ------------------------------------------------------------   0
```

```
Darter_fish           -------------GKSCRSGGKCL-SPSKWC------DGVKDCSHGEDESQCFRLHGTNF   147
Trout_X1              -------------GRSCRQGCVYL-SASKWC------DGIRDCPGGEDETQCLRLYGSGS   148
Frog_V1               ELNCDTRTTTAYCEKRCGSSVSCV-LSSQWC------DGVSDCPYGEDEMSCVSLYPADF   431
Duck                  ------------SL-ECGSSGVCV-PPSDWC------DGVTNCPNGEDENRCVRLFGPNF   144
Chicken_X1            ------------SLIECGSSGVCI-SPSVWC------DGVTDCPNGEDENRCVRLYGPNF   148
Rock_dove             ------------SLIECGSSGVCL-QPSRWC------DGVNHCPNGEDENRCVRLFGPNF   146
Tiger_snake_X1        ------------SKIQCGSLRVCV-LPSQWC------DGKKDCPNGEDENRCVRLYGPEF   169
Camel                 ------------SEMECGSSGTCI-SPSQWC------DGVLHCPGGEDENQCVRLYGPNF   156
Goat                  -------------GMECGSSGTCV-SPSLWC------DGIRHCPGGEDENHCVRLYGPNF   154
Cow                   -------------GMECSSSGTCV-SPSLWC------DGILHCPSGEDENRCVRLYGPNF   154
Sheep                 -------------GMECGSSGTCV-SPSLWC------DGILHCPGGEDENRCVRLYGPNF   154
Beluga_X1             -------------GMECSSSGTCV-SPSHWC------DGILHCPSGEDESRCVRLYGPNF   123
Dolphin               -------------GMECSSSGTCV-SPSHWC------DGILHCPSGEDESRCVRLYGPNF   154
Macaque               ------------SGIECDSSGTCI-SSSNWC------DGVSHCPNGEDENRCVRLYGPNF   156
Human_V2              ------------SGIECDSSGTCI-NPSNWC------DGVSHCPGGEDENRCVRLYGPNF   156
Human_V1              ------------SGIECDSSGTCI-NPSNWC------DGVSHCPGGEDENRCVRLYGPNF   193
Gorilla_X1            ------------SGIECDSSGTCI-SPSNWC------DGVSHCPSGEDENRCVRLYGPNF   156
Gorilla_X2            ------------SGIECDSSGTCI-SPSNWC------DGVSHCPSGEDENRCVRLYGPNF   156
Mouse                 ------------SEMECGSSGTCI-SSSLWC------DGVAHCPNGEDENRCVRLYGQSF   155
Rat                   ------------SEMECGTSGTCI-SSSLWC------DGVSQCPNGEDENRCVRLYGTSF   155
Elephant              ------------SGVECGSSGTCI-SASNWC------DGISHCPSGEDENRCVRLYGPNF   155
Dog                   ------------SGIECGSSGTCI-SPSQWC------DGVLHCPSGEDENRCVRLYGPNF   156
Rabbit                ------------SGMECGPSRICI-RTSQWC------DGVLHCPNGEDENRCVRLYGPNF   154
Horse_X1              ------------SRIECGNSGICT-SASQWC------DGILHCPNGEDENRCVRLYGLNF   156
Horse_X2              ------------SRIECGNSGICT-SASQWC------DGILHCPNGEDENRCVRLYGLNF   156
Lemur                 ------------SQVECGSSGMCV-SPDLWC------DGVSHCPGGEDETRCVRLYGPNF   208
Ground_Squirrel       ------------YEMECGSSGTCV-SPSFWC------DGIAHCPNGEDENQCVRLYGPNF   156
Pig                   -----------TPEMECGSSGTCI-SPSHWC------DGILHCPGGEDENQCVRLYGPNF   159
Tiger                 ------------SGIECGSSGTCI-SPAHWC------DGVLHCPSGEDENRCVRLYGPNF   156
Grey_Seal_X1          ------------SGVECGSSGTCI-SPSHWC------DGVLHCPSGEDENRCVRLYGPNF   156
Bear                  ------------SGIECGSSGTCI-SPSQWC------DGVLHCPSGEDENRCVRLYGPSF   156
Ferret                -TLLAQRGPSHPGGSISQAGPPCPLPASAACPCVLLNEGTASRSSQSLSPAAVRLYGPNF   243
Cat_X1                ------------SGIECGSSGTCV-SPSHWC------DGVLHCPSGEDENRCVRLYGPNF   156
Cat_X2                ------------SGIECGSSGTCV-SPSHWC------DGVLHCPSGEDENRCVRLYGPNF   156
Cat_X3                ------------SGIECGSSGTCV-SPSHWC------DGVLHCPSGEDENRCVRLYGPNF   156
Bat                   ------------SGMECGSSGTCV-SASLWC------DGILHCPSGEDENQCVRLYGPNF   156
Bat_Little_Brown      ------------------------------------------------------------   0
```

```
Darter_fish        LLETYSSHSQMWMPVCAENWNDNYGRAVCEHMGYKRQDYVSYTQTSAGSLASRGYMKLKA   207
Trout_X1           VLQSYSSDSQTWKPVCADDWNDNFGRATCKKMGYSSGSYVRSSQRNPGSLASEGYLKLSS   208
Frog_V1            QLQVYSTSVSAWLPVCSDYWNDDFGRFACQDFGYNGSSYNRYDT-LMSPYAPNGYFKLYS   490
Duck               ILEVYSPVSNTWYPVCQDNWTDDFGKIACKDIGYNVDTYYSSSG-VAAEVSFKSFMKLNT   203
Chicken_X1         ILEVYSPVSQTWYPVCQDDWTDDFGKIACEDMGYNVDTYYYSQG-VAAEVSFKSFMKLNT   207
Rock_dove          ILEVYSPVSKCWYPVCQDDWNDDYGKTACQDMGYSVDTYYYSQG-VAAEASFKSYMKLNT   205
Tiger_snake_X1     LLEVYSPENKDWYSICYDDWNNQYGKTACEDLGYNSKTYFTSTA-IPATTGSAKILRVNT   228
Camel              ILQVYSPQRKSWHPVCQEDWSESFGRAMCQDLGYG-NSFYSSQG-VVDDSGATSFMKLNI   214
Goat               ILQVYSAQRKSWHPVCQDDWSESYGRAACQDMGYR-NSFYSSQG-IADDSGATSFMKLNI   212
Cow                ILQVYSAQRKSWHPVCQDDWSESYGRAACQDMGYR-NSFYSSQG-IADDSGATSFMKLNI   212
Sheep              ILQVYSAQRKSWHPVCQDNWSESYGRAACQDMGYR-NSFYSSQG-IADDSGATSFMKLNI   212
Beluga_X1          ILQVYSTQRKSWHPVCQDDWSDNYGRAACQDMGYP-NSFFSSQG-IVDDSGATSFMKLNV   181
Dolphin            ILQVYSAQRKSWHPVCQDDWSDNYGRAACQDMGYP-NSFFSSQG-IVDDSGATSFMKLNV   212
Macaque            ILQVYSSQRKSWHPVCRDDWNENYARAACRDMGYK-NSFYSSQG-IVDNSGATSFMKLNT   214
Human_V2           ILQVYSSQRKSWHPVCQDDWNENYGRAACRDMGYK-NNFYSSQG-IVDDSGSTSFMKLNT   214
Human_V1           ILQVYSSQRKSWHPVCQDDWNENYGRAACRDMGYK-NNFYSSQG-IVDDSGSTSFMKLNT   251
Gorilla_X1         ILQVYSSQRKSWHPVCQDDWNENYGRAACRDMGYK-NNFYSSQG-IVDDSGSTSFMKLNT   214
Gorilla_X2         ILQVYSSQRKSWHPVCQDDWNENYGRAACRDMGYK-NNFYSSQG-IVDDSGSTSFMKLNT   214
Mouse              ILQVYSSQRKAWYPVCQDDWSESYGRAACKDMGYK-NNFYSSQG-IPDQSGATSFMKLNV   213
Rat                TLQVYSSQRKAWYPVCQDDWNESYGRAACKDMGYK-NSFYSSQG-IPDQSGATSFMKLNV   213
Elephant           ILQVYSAPRNSWHPVCWDNWRESYAWAACGDMGYK-QSFYSSQG-VVDDSGATSFLKLNT   213
Dog                ILQVYSSQRKSWHPVCQDNWSDSYGRAACQDMGYR-NSFYSSQG-IADDSGATSFMKLNI   214
Rabbit             ILQVYSSQRKSWHPVCQDDWNESYGRAACKDMGYQ--SFYTSSG-IADNSGATSFMKLNL   211
Horse_X1           ILQVYSSQRKSWHPVCEDDWSESYGRAVCRDMGYG-NSFYSSQG-IKDDSGATRFMKLNT   214
Horse_X2           ILQVYSSQRKSWHPVCEDDWSESYGRAVCRDMGYG-NSFYSSQG-IKDDSGATRFMKLNT   214
Lemur              ILQVYSSQRKSWHPVCWDYWSESYGRXACRDLGYGENNFYSSQG-IADDSGATSFMKLNA   267
Ground_Squirrel    ILQVYSPERQAWHPVCQDDWNEHFARAACKDMGYK-NSFYSTRG-IADDSGAASFMKLNA   214
Pig                ILQVYSAQRKSWYPVCQDDWTENYGRAACQDMGYR-NSFFSSQG-IADDSGATSFMKLNK   217
Tiger              ILQVYSSQRKSWHPVCQDDWSEGYGRAACQDMGYR-NSFYSSRG-VADDSGATSFMRVNA   214
Grey_Seal_X1       ILQVYSAQRKSWHPVCQDDWNDSYGRAACQDMGYR-NSFYSSQG-IVDDSGATSFMKLNL   214
Bear               ILQVYSAQRKSWHPVCQDDWNDSYGRAACQDMGYG-NSFYSSHG-IVDDSGATSFMKLNV   214
Ferret             ILQVYSAQRKSWHPVCQDDWSDSYGRAACQDMGYR-PEC---------------------   281
Cat_X1             ILQVYSSQRKSWHPVCQDDWSEGYGRAACQDMGYR-NSFYSSHG-VADDSGATSFMRVNT   214
Cat_X2             ILQVYSSQRKSWHPVCQDDWSEGYGRAACQDMGYR-NSFYSSHG-VADDSGATSFMRVNT   214
Cat_X3             ILQVYSSQRKSWHPVCQDDWSEGYGRAACQDMGYR-NSFYSSHG-VADDSGATSFMRVNT   214
Bat                ILQVYSSQRKSWHPVCHDDWSESYGRAACQDMGYR-NSFYSSQG-IADDSGATSFMKLNT   214
Bat_Little_Brown   ------------------------------------------------------------   0
```

```
Darter_fish          GSSLGSRTQSQLTYSQSCS-ARAVKLNCIECGVSSAA--PSARIVGGTAAVN--------   256
Trout_X1             FDP-QSLLQRQLTGSPYCS-AQAVSLQCIDCGVSIAA--PWSRIVGGDIAVS--------   256
Frog_V1              GYW-RSKFYTSVQYSSYCYSGNVVSLHCISCGVSNNS--LVSRIVGGTFANL--------   539
Duck                 SAG-STDLYKRLYTSNVCTSGKVVSLRCIQCGLSTKSTAIMNRIVGGSAAAL--------   254
Chicken_X1           SAG-NTDLYKRLQSSDYCASGNVVSLRCIECGLPTKSTAVMSRIVGGSMASL--------   258
Rock_dove            SAG-NIDLYKKLYSSDYCASGNVVSLRCIKCGVSTKSVNMMSRIVGGSGATL--------   256
Tiger_snake_X1       SAE-DIDLYKKLYNSKLCPSKQVVSLRCIECGISNKHTSPRNRIVGGNAALP--------   279
Camel                SAN-NIDLYKKLYHSDVCSSKRVVSLRCIECGVSEKTS-RQSRIVGGSSANL--------   264
Goat                 SAN-NIDLYKKLYHSNVCSSKTVVSLRCIECGVSMKTS-RQSRIVGGSNAYS--------   262
Cow                  SAN-DIDLYKKLYHSDVCSSKTVVSLRCIECGVSVKTS-RQSRIVGGSNAYS--------   262
Sheep                SAN-NIDLYKKLYHSDVCSSKTVVSLRCIECGVAVKTS-RQSRIVGGSNANS--------   262
Beluga_X1            SAN-NIDLYKKLYHSDVCSSKTVVSLRCIECGVSGKTS-RQSRIVGGSSAAP--------   231
Dolphin              SAN-NIDLYKKLYHSDVCSSKTVVSLRCIECGVSGKTR-RQSRIVGGSSAAP--------   262
Macaque              SAG-NVDIYKKLYHSDACSSKAVVSLRCIACGVRSNLN-RQSRIVGGQNALL--------   264
Human_V2             SAG-NVDIYKKLYHSDACSSKAVVSLRCIACGVNLNSS-RQSRIVGGESALP--------   264
Human_V1             SAG-NVDIYKKLYHSDACSSKAVVSLRCIACGVNLNSS-RQSRIVGGESALP--------   301
Gorilla_X1           SAG-NVDIYKKLYHSDACSSKAVVSLRCIACGVNLNSS-RQSRIVGGVSALP--------   264
Gorilla_X2           SAG-NVDIYKKLYHSDACSSKAVVSLRCIACGVNLNSS-RQSRIVGGVSALP--------   264
Mouse                SSG-NVDLYKKLYHSDSCSSRMVVSLRCIECGVRS--VKRQSRIVGGLNASP--------   262
Rat                  SAG-NIDLYKKLYHSDSCSSRMVVSLRCIACGVRS--VRRQSRIVGGSTASP--------   262
Elephant             SVG-STDLYKRLFHSTTCSSKTVVSLRCIECGVTSSMT-HQSRIVGGESAAL--------   263
Dog                  SAG-HMDLYKKLYHSDVCSSKTVVSLRCIGKHLSWAAV--------------------   251
Rabbit               SAS-NHDLYQKLYHSDTCSSKAVVSLRCIECGVTSP--GRQGRIVGGSHAAP--------   260
Horse_X1             SAD-NIDLYKKLYHSDVCSSKKVVSLRCMECGVSSK---QQSRIVGGANAAL--------   262
Horse_X2             SAD-NIDLYKKLYHSDVCSSKKVVSLRCMECGVSSK---QQSRIVGGANAAL--------   262
Lemur                SAS-SLDIYKKLYHSDSCPSKEVVSLRCIACGTTSNSS-RQSRIVGGSGAAL--------   317
Ground_Squirrel      SVV-TVDLYKKLYHSDSCSSNTVVSLRCVECGG--PFLRRQSRIVGGSNANL--------   263
Pig                  SAN-NMDLYKKLYHSDVCTSNTVVSLRCIECGVSGKMSNRQSRIVGGSSAAL--------   268
Tiger                SAH-HVDLYKKLYHSDVCSSKTVVSLRCIGKQFLVSTA-PLLFFSKGSTLYLQKCIPCVW   272
Grey_Seal_X1         SAG-SLDLYKKLYHSDVCSSKTVVSLRCIECGVTGKMI-RQSRIVGGSSA-----AL---   264
Bear                 TAG-NMDLYKKLYHSDVCSSKTVVSLRCIECGVTAKMS-RQSRIVGGSSA-----SE---   264
Ferret               ---------PLFFCSDICASKTVVSLRCIECGVAGKTM-RQSRIVGGSSA-----SP---   323
Cat_X1               SAN-HMDLYKKLYHSDVCSSKTVVSLRCIECGVTAKMG-RQSRIVGGSSA-----AP---   264
Cat_X2               SAN-HMDLYKKLYHSDVCSSKTVVSLRCIECGVTAKMG-RQSRIVGGSSA-----AP---   264
Cat_X3               SAN-HMDLYKKLYHSDVCSSKTVVSLRCIECGVTAKMG-RQSRIVGGSSA-----AP---   264
Bat                  SAG-STDLYKKLYHSDVCSSKTVVSLRCIECGVNSKMG-RQSRIVGGSSA-----AP---   264
Bat_Little_Brown     ------------------------------------MG-RQSRIVGGSSA-----AP---   15
```

```
Darter_fish          --GAWPWQVSLQI---------QHQ---HICGGS----IISPYWILSAAHCFE-RYSHPE    297
Trout_X1             --GAWPWQVSLHA---------RGQ---HLCGGS----IISPEWILTAAHCVE-TLSRPS    297
Frog_V1              --GNWPWQVNLQYI------------TGVLCGGS----IISPKWIVTAAHCVYGSYSSAS    581
Duck                 --GQWPWQLSLHV---------QGT---HVCGGS----IITREWLVTAAHCVEGQLSDPY    296
Chicken_X1           --GQWPWQVSLHV---------QDT---HVCGGS----IITREWLVTAAHCVEGLFSDPY    300
Rock_dove            --GQWPWQVSLHV---------QGT---HVCGGS----IITPQWIVTAAHCVEGQFSDPY    298
Tiger_snake_X1       --GEWPWQVSLHV---------RR---THLCGGS----IITPEWIVTAAHCVEGAHSDPS    321
Camel                --GDWPWQVSLHV---------QGI---HVCGGS----IITPEWIVTAAHCVEQPLSNAK    306
Goat                 --GQWPWQVSLHV---------QGI---HVCGGS----IITPEWIVTAAHCVEEPLNNPK    304
Cow                  --GEWPWQVSLHV---------QGI---HVCGGS----IITPEWIVTAAHCVEEPLNNPK    304
Sheep                --GEWPWQVSLHV---------QGI---HVCGGS----IITPEWIVTAAHCVEEPLNNPK    304
Beluga_X1            --GDWPWQVSLHV---------QGT---HVCGGS----IITPEWIVTAAHCVEEPLNNPK    273
Dolphin              --GDWPWQVSLHV---------QGT---HVCGGS----IITPEWIVTAAHCVEEPLNNPK    304
Macaque              --GAWPWQVSLHV---------QNI---HVCGGS----IITPEWIVTAAHCVEKPLNSPW    306
Human_V2             --GAWPWQVSLHV---------QNV---HVCGGS----IITPEWIVTAAHCVEKPLNNPW    306
Human_V1             --GAWPWQVSLHV---------QNV---HVCGGS----IITPEWIVTAAHCVEKPLNNPW    343
Gorilla_X1           --GAWPWQVSLHV---------QNV---HVCGGS----IITPEWIVTAAHCVEKPLNNPW    306
Gorilla_X2           --GAWPWQVSLHV---------QNV---HVCGGS----IITPEWIVTAAHCVEKPLNNPW    306
Mouse                --GDWPWQVSLHV---------QGV---HVCGGS----IITPEWIVTAAHCVEEPLSSPR    304
Rat                  --GDWPWQVSLHV---------QGI---HVCGGS----IITPEWIVTAAHCVEEPLSSPR    304
Elephant             --GAWPWQVSLHV---------QGV---HVCGGS----IITPQWIVTAAHCVEEPLSSPW    305
Dog                  ---SW--RCRQPRVQE----H-RPVRTSSSGDGRALAVPVSVSGLCMAPTLFPGPLHNPR    301
Rabbit               --GEWPWQVSLHV---------QGI---HVCGGS----IITPQWIVTAAHCLEEPLNSAR    302
Horse_X1             --GEWPWQVSLHV---------QDV---HVCGGS----IITPEWIVTAAHCVEPPLNSPR    304
Horse_X2             --GEWPWQVSLHV---------QDV---HVCGGS----IITPEWIVTAAHCVEPPLNSPR    304
Lemur                --GDWPWQVSLHV---------QNL---HVCGGS----VITPEWIVTAAHCVEQPLNSAR    359
Ground_Squirrel      --GDWPWQVSLHV---------QGV---HVCGGS----IITPEWIVTAAHCVEEPLNSPR    305
Pig                  --GDWPWQVSLHV---------QGI---HICGGS----IITPDWIVTAAHCVEEPLNNPK    310
Tiger                GGGVMGWDVSEPCARSVPQLHSRNTFLELRCPGDASGLGARDAAVLRGQAISGKPLNNPR    332
Grey_Seal_X1         --GDWPWQVSLHV---------QGT---HVCGGS----IITPEWIVTAAHCVEEPLNNPR    306
Bear                 --GDWPWQVSLHV---------QGT---HVCGGS----VITPEWIVTAAHCVEEPLNSPR    306
Ferret               --GDWPWQVSLHV---------QGT---HVCGGS----IITPEWIVTAAHCVEEPLNNPR    365
Cat_X1               --GDWPWQASLHV---------QGV---HVCGGS----IISPEWIVTAAHCVEEPLNNPR    306
Cat_X2               --GDWPWQASLHV---------QGV---HVCGGS----IISPEWIVTAAHCVEEPLNNPR    306
Cat_X3               --GDWPWQASLHV---------QGV---HVCGGS----IISPEWIVTAAHCVEEPLNNPR    306
Bat                  --GDWPWQVSLHV---------QGV---HVCGGS----IITPEWIVTAAHCVEEPLNHPR    306
Bat_Little_Brown     --GDWPWQVSLHV---------QGV---HVCGGS----IITPEWIVTAAHCVEEPLNNPR    57
                                                     *           :  .
```

```
Darter_fish             LWRVYFGDVSLNQMAFGSGKTVSRIISHEKFDKDTNDNDIALLKLNTPLTFTRTVKPVCL    357
Trout_X1                QWTVYAGYLALTQMDFATGNSVGHIISHEKYDKQTSDNDIALMKLSTPLTMSNTVRPVCL    357
Frog_V1                 GWRVFAGTLTKPSYYNASAYFVERIIVHPGYKSYTYDNDIALMKLRDEITFGYTTQPVCL    641
Duck                    VWRVYAGIVSQSDITLRAAYKVQQIISHPDYDTDTKDNDVALMKLETPLSFTDNVQPVCL    356
Chicken_X1              IWSVYAGILSQNEMHSRPGYRVQKIISHPNYDTDSKDNDVALMKLETPLSFTNTIRPVCL    360
Rock_dove               NWRVYAGILNQNEMFLGYGYRVQQIISHPDYDTDSKDNDVALMKLETPLSFTDTVRPVCL    358
Tiger_snake_X1          YWKVYAGILRQPEMVLSKGFSVARIISHPNYDTTSKNNDVALMKLQSPLSFNDFIRPVCL    381
Camel                   IWTAFAGILSQSLMIYGNGYQIAKVISHPNYDSKTKNNDIALMKLQTPLTFNDRVKPVCL    366
Goat                    IWAAFAGILKQSYMFYGSGYRVAKVISHPNYDSNTKNNDIALMKLQTPLTFN--------    356
Cow                     IWVAFAGILKQSYMFYGSGYRVAKVISHPNYDSKTKNNDIALMKLQTPLTFNDKVKPVCL    364
Sheep                   IWAAFAGILKQSYMFYGSGYRVAKVISHPNYDSKTKNNDIALMKLQKPLTFNDKVKPVCL    364
Beluga_X1               IWTAFAGILRQSFMFYGNGYRVAKVISHPNYDSKTKNNDIALMKLQTPLTINDRVKPVCL    333
Dolphin                 IWTAFAGILRQSFMFYGNGYRVAKVISHPNYDSKTKNNDIALMKLQTPLTFNDRVKPVCL    364
Macaque                 QWTAFVGTLRQSSMFYEKGHRVEKVISHPNYDSKTKNNDIALMKLHTPLTFNEVVKPVCL    366
Human_V2                HWTAFAGILRQSFMFYGAGYQVEKVISHPNYDSKTKNNDIALMKLQKPLTFNDLVKPVCL    366
Human_V1                HWTAFAGILRQSFMFYGAGYQVEKVISHPNYDSKTKNNDIALMKLQKPLTFNDLVKPVCL    403
Gorilla_X1              HWTAFAGILRQSFMFYEAGYQVEKVISHPNYDSKTKNNDIALMKLQKPLTFNDLVKPVCL    366
Gorilla_X2              HWTAFAGILRQSFMFYEAGYQVEKVISHPNYDSKTKNNDIALMKLQKPLTFNDLVKPVCL    366
Mouse                   YWTAFAGILRQSLMFYGSRHQVEKVISHPNYDSKTKNNDIALMKLQTPLAFNDLVKPVCL    364
Rat                     YWTAFAGILKQSLMFYGSRHQVERVISHPNYDSKTKNNDIALMKLQTPLAFNDVVKPVCL    364
Elephant                YWTAFAGILKQSSMVYGSGHQVEKVISHPNYDSKTKNNDVALMKLQAPLTFNDRVSPVCL    365
Dog                     YWTAVAGILRQSFMFYGHGHRVGKVISHTNYDSKTKNNDIALMKLQTPLTFNDRVKPVCL    361
Rabbit                  YWMAFAGILSQFSMLYPSGYQAEKVISHPNYDSKTKNNDIALIKLQTPLTFNDRVRPVCL    362
Horse_X1                YWTAFAGILSQSLMFYENGHRVGKVIAHPNYDSKTKNNDIALMKLEAPLTFNDRVKPVCL    364
Horse_X2                YWTAFAGILSQSLMFYENGHRVGKVIAHPNYDSKTKNNDIALMKLEAPLTFNDRVKPVCL    364
Lemur                   HWTAYAGILRQSFMSYGGGNRVGKVISHPNYDSKTKNNDIALMKLQTPLTFDHNVRPVCL    419
Ground_Squirrel         YWTVFAGLLRQSSMLYGSGYRVEKVISHPNYDSKTKNNDVALMKLQAPLTFNDRVKPVCL    365
Pig                     IWTAFAGILRQSFMFYGSGYRVAKVISHPNYDPKTKNNDIALMKLQTPMTFNDKVKPVCL    370
Tiger                   HWTAFVGILRQSFMFYGHGYRVGKVISHPNYDSKSKNNDIALMKLQTPLTFDDKVKPVCL    392
Grey_Seal_X1            YWTAFAGILRQSFMFYGHGYRVGKVISHPNYDSKTKNNDIALMKLQTPLTFNDKVKPVCL    366
Bear                    YWTAFAGILRQSFMFYGQGYRVGKVISHPNYDSKTKNNDIALMKLQTPLTFNDKVKPVCL    366
Ferret                  YWTVFAGVLRQSFMFYGHGYRVGKVISHPSYDSKTKNNDIALMKLQTPLTFSDKVKPVCL    425
Cat_X1                  HWTAFVGILRQSFMFYGHGYRVGKVISHPNYDSKTKNNDIALMKLQTPLTFDDKVKPVCL    366
Cat_X2                  HWTAFVGILRQSFMFYGHGYRVGKVISHPNYDSKTKNNDIALMKLQTPLTFDDKVKPVCL    366
Cat_X3                  HWTAFVGILRQSFMFYGHGYRVGKVISHPNYDSKTKNNDIALMKLQTPLTFDDKVKPVCL    366
Bat                     NWMAFAGILRQSAMFYGNAHRVGKVISHPHYDSKTKNNDIALMKLQTPLTFNDNVRPVCL    366
Bat_Little_Brown        YWMAFAGILRQSAMFYGNAYRVGKVISHPNYDSKTKNNDIALMKLQTPLTFNDKVKPVCL    117
                         * .  * :              ::* *  :.  : :**:**:**   :::
```

```
Darter_fish           PNIGLDLSAGRQAWITGWGALRSSGPSPDRLNQAQVTVYSRETCNSPQVLSGKISETMIC   417
Trout_X1              PNVGVNLTPQREAWISGWGSIRSGGSSSGNLRQAQITMYSRETCNASLVYNGLVTASMIC   417
Frog_V1               PNSGMFWEAGTTTWISGWGSTYEGGSVSTYLQYAAIPLIDSNVCNQSYVYNGQITSSMIC   701
Duck                  PNAGMTFQPNQQCWISGWGAESQGGKTSTNLNYVMVPLIERSKCNSIYIYNGLILPTMIC   416
Chicken_X1            PNPGMMFQPNQQCWISGWGAEYQGGKTANDLNYVMVPLIERSTCNSVYVYDGMVLPTMVC   420
Rock_dove             PNPGMMFEPNQQCWISGWGAEYQGGKTSNTLNYVMVSLIERSRCNSGYIYNGMILPTMIC   418
Tiger_snake_X1        PNAGMMFETDQQCWISGWGAKYQGGNTSKDLNAAAVPLIDPFICNGRYIYNGIILPTMIC   441
Camel                 PNPGMMLEATQSCWISGWGATYEKGKTSDVLNAVMVHLIEPWKCNSKYVYNNLITSAMIC   426
Goat                  ------------------------GKTSDDLNTAEVHLIEPRKCNSKYMYDNLITPAMIC   392
Cow                   PNPGMMLEPTQSCWISGWGATYEKGKTSDDLNAAKVHLIEPRKCNSKYMYDNLITPAMIC   424
Sheep                 PNPGMMLEPTQSCWISGWGATYEKGKTSDDLNAAKVHLIEPRKCNSKYMYDNLITPAMIC   424
Beluga_X1             PNPGMMLEPAQSCWISGWGSTYEKGKTSDRLNAAMVQLIEPWRCDSKQVYSDLITPAMIC   393
Dolphin               PNPGMMLEPTQACWISGWGSTYEKGKTSDRLNAAMVQLIEPWRCDSKQVYSDLITPAMIC   424
Macaque               PNPGMMLEPEQHCWISGWGATQEKGKTSDVLNAAMVPLIEPRRCNNKYVYDGLITPAMIC   426
Human_V2              PNPGMMLQPEQLCWISGWGATEEKGKTSEVLNAAKVLLIETQRCNSRYVYDNLITPAMIC   426
Human_V1              PNPGMMLQPEQLCWISGWGATEEKGKTSEVLNAAKVLLIETQRCNSRYVYDNLITPAMIC   463
Gorilla_X1            PNPGMMLEPEQLCWISGWGATEEKGKTSEVLNAARVLLIETQKCNSRYVYDNLITPAMIC   426
Gorilla_X2            PNPGMMLEPEQLCWISGWGATEEKGKTSEVLNAARVLLIETQKCNSRYVYDNLITPAMIC   426
Mouse                 PNPGMMLDLDQECWISGWGATYEKGKTSDVLNAAMVPLIEPSKCNSKYIYNNLITPAMIC   424
Rat                   PNPGMMLDLAQECWISGWGATYEKGKTSDVLNAAMVPLIEPSKCNSKYIYNNLITPAMIC   424
Elephant              PNPGLMLEPTQTCWISGWGATQEKGKTSNELNAAKVPLIETWICNNRYIYNDLVTPTMIC   425
Dog                   PNPGMMLEPEQSCWISGWGATYEKGKTSDELNAVMVPLIEPWRCNSKYVYNNLVTPAMIC   421
Rabbit                PNPGLMLEPEQPCWISGWGATYEKGKTSDLLNAAMVPLIESWKCNSKYVYNNLITPSMIC   422
Horse_X1              PNPGMRLEPKQSCWISGWGATYEKGKTSELLNAAMVPLIEPEQCNNKFIYNDLITPAMIC   424
Horse_X2              PNPGMRLEPKQSCWISGWGATYEKGKTSELLNAAMVPLIEPEQCNNKFIYNDLITPAMIC   424
Lemur                 PNPGLMLAPEQPCWISGWGATEEKGKTSDTLNAAQVRLIEPSKCNSKYVYNNLVTPAMVC   479
Ground_Squirrel       PNPGMMLEAQQPCWISGWGATYEKGKTSDVLNAAMVYLIEHSRCNSKYVYNNLITPAMIC   425
Pig                   PNPGMMLEPTQSCWISGWGATYEKGKTSEVLNAAMVRLIEPWSCNSKQVYNNLITPAMIC   430
Tiger                 PNPGLMLEPEQPCWISGWGATHEKGKTSDELNAVMVPLIEPWRCNSKYVYNNLVTPAMIC   452
Grey_Seal_X1          PNPGMMLEPDQSCWISGWGATYEKGTTSDELNAVMVPLIEPGRCNSKYVYNNLVTPAMVC   426
Bear                  PNPGLMLEPDQSCWISGWGATHEKGKTSDELNAAMVPLIEPQHCNSKYVYNNLVTPAMVC   426
Ferret                PNPGMMLEPNQSCWISGWGATHEKGKTSDELNAVMVPLIEPWRCNSKYVYNSLVTPAMIC   485
Cat_X1                PNPGLMLEPEQPCWISGWGATHEKGKTSDELNAVMVPLIEPWRCNSKYVYNNLVTPAMIC   426
Cat_X2                PNPGLMLEPEQPCWISGWGATHEKGKTSDELNAVMVPLIEPWRCNSKYVYNNLVTPAMIC   426
Cat_X3                PNPGLMLEPEQPCWISGWGATHEKGKTSDELNAVMVPLIEPWRCNSKYVYNNLVTPAMIC   426
Bat                   PNPGLMLEPRQACWISGWGATYEKGKTSDVLNAARVTLIEPARCNSRFVYNNLVTPAMLC   426
Bat_Little_Brown      PNPGLMLEPRQACWISGWGATYEKGKTSDMLNAAMVPLIEPGQCNGRYVYNNLVTPTMVC   177
                                              *     *. . : : .   *:   : .. :  :*:*
```

```
Darter_fish        AGRLEGGVDSCQGDSGGPLVVKEGNVWWLAGDTSWGIGCAWRNKPGVYGNVTYFINWVYE   477
Trout_X1           AGTLAGGVDSCQGDSGGPMVAKEGSVWWLVGDTSWGIGCALKNKPGIYGNVTHFLPWIYE   477
Frog_V1            AGYLSGGVDTCQGDSGGPLVNKRNGTWWLVGDTSWGDGCARANKPGVYGNVTTFLEWIYS   761
Duck               AGYLEGGIDSCQGDSGGPLVTNESSVWWLVGDTSWGSGCATPNRPGVYGNMTVFTDWIYK   476
Chicken_X1         AGYLQGGIDSCQGDSGGPLVTNKNSVWWLVGDTSWGTGCASPNRPGVYGNMTVFTDWIYK   480
Rock_dove          AGFLEGGVDSCQGDSGGPLVTNKNSIWWLVGDTSWGTGCASPNRPGVYGNMTVFTDWIYK   478
Tiger_snake_X1     AGYLKGMVDSCQGDSGGPLVTMKNLLWWLVGDTSWGTGCATQYKPGVYGNITMFTEWIYR   501
Camel              AGYLQGGVDSCQGDSGGPLVTLKNSVWWLLGDTSWGSGCAKAYRPGVYGNMTVFTDWIYR   486
Goat               AGYLRGTIDSCQGDSGGPLVTLKSSVWWLIGDTSWGSGCAKAYRPGVYGNVTVFTDWIYR   452
Cow                AGYLRGTVDSCQGDSGGPLVTLKSSVWWLIGDTSWGSGCAKAYRPGVYGNVTVFTDWIYQ   484
Sheep              AGYLQGTVDSCQGDSGGPLVTLKSSVWWLIGDTSWGSGCAKAYRPGVYGNVTVFTDWIYR   484
Beluga_X1          AGYLQGTVDSCQGDSGGPLVTLKSSVWWLIGDTSWGSGCAKAYRPGVYGNVTVFTDWIYR   453
Dolphin            AGYLQGTVDSCQGDSGGPLVTLKSSVWWLIGDTSWGSGCAKAYRPGVYGNVTVFTDWIYR   484
Macaque            AGFLQGTVDSCQGDSGGPLVTLKNDVWWLIGDTSWGSGCAQANRPGVYGNVTVFTDWIYR   486
Human_V2           AGFLQGNVDSCQGDSGGPLVTSKNNIWWLIGDTSWGSGCAKAYRPGVYGNVMVFTDWIYR   486
Human_V1           AGFLQGNVDSCQGDSGGPLVTSKNNIWWLIGDTSWGSGCAKAYRPGVYGNVMVFTDWIYR   523
Gorilla_X1         AGFLQGNVDSCQGDSGGPLVTLKNNVWWLIGDTSWGSGCAKAYRPGVYGNVTVFTDWIYR   486
Gorilla_X2         AGFLQGNVDSCQGDSGGPLVTLKNNVWWLIGDTSWGSGCAKAYRPGVYGNVTVFTDWIYR   486
Mouse              AGFLQGSVDSCQGDSGGPLVTLKNGIWWLIGDTSWGSGCAKALRPGVYGNVTVFTDWIYQ   484
Rat                AGFLQGSVDSCQGDSGGPLVTLKNEIWWLIGDTSWGSGCAKAFRPGVYGNVTVFTDWIYQ   484
Elephant           AGYLEGNIDSCQGDSGGPLVTLKSSVWWLIGDTSWGSGCAKVNRPGVYGNMTVFTDWIYQ   485
Dog                AGFLRGGVDSCQGDSGGPLVTLKSRVWWLIGDTSWGSGCAKANRPGVYGNVTVFTDWIYR   481
Rabbit             AGYLQGTIDSCQGDSGGPLVTLKNDIWWLIGDTSWGSGCAKVNRPGAYGNVTAFTDWIYQ   482
Horse_X1           AGYLEGKVDSCQGDSGGPLVTLKSSIWWLIGDTSWGSGCAKMNRPGVYGNVTVFTDWIYQ   484
Horse_X2           AGYLEGKVDSCQGDSGGPLVTLKSSIWWLIGDTSWGSGCAKMNRPGVYGNVTVFTDWIYQ   484
Lemur              AGYLQGTVDSCQGDSGGPLVTRKNSIWWLVGDTSWGSGCAKPNRPGVYGNVTVFADWIYQ   539
Ground_Squirrel    AGYLQGSIDSCQGDSGGPLVTLKNEVWWLIGDTSWGSGCAKPNRPGVYGNMTVFTDWIYR   485
Pig                AGYLQGSVDSCQGDSGGPLVTLKSSIWWLIGDTSWGSGCAKAYRPGVYANVTLFTDWIYR   490
Tiger              AGYLQGTIDSCQGDSGGPLVTMKSHIWWLIGDTSWGSGCAKANRPGVYGNVTVFTDWIYR   512
Grey_Seal_X1       AGYLQGSIDSCQGDSGGPLVTLKSRIWWLIGDTSWGSGCAKANRPGVYGNVTVFTDWIYR   486
Bear               AGYLQGSVDSCQGDSGGPLVTLKSRIWWLIGDTSWGSGCAKANRPGVYGNMTVFTDWIYR   486
Ferret             AGYLRGGTDSCQGDSGGPLVTLKSRIWWLIGDTSWGSGCAKANRPGVYGNVTVFTDWIYR   545
Cat_X1             AGYLQGTIDSCQGDSGGPLVTMKSHIWWLIGDTSWGSGCAKANRPGVYGNVTVFTDWIYR   486
Cat_X2             AGYLQGTIDSCQGDSGGPLVTMKSHIWWLIGDTSWGSGCAKANRPGVYGNVTVFTDWIYR   486
Cat_X3             AGYLQGTIDSCQGDSGGPLVTMKSHIWWLIGDTSWGSGCAKANRPGVYGNVTVFTDWIYR   486
Bat                AGYLAGSVDSCQGDSGGPLVTLKSSIWWLIGDTSWGSGCAKANRPGVYGNVTMFTDWIYR   486
Bat_Little_Brown   AGYLRGSVDSCQGDSGGPLVTLKNSIWWLIGDTSWGSGCAKANRPGVYGNVTMFTDWIYR   237
                   ** * *  *:********:*  ..  *** ****** ***   :** *.*:  *  *:*
```

```
Darter_fish           QMQNE--------------------------------------- 482
Trout_X1              QMQKN--------------------------------------- 482
Frog_V1               QMRTYR-------------------------------------- 767
Duck                  NMQANR-------------------------------------- 482
Chicken_X1            NMQANR-------------------------------------- 486
Rock_dove             NMQANR-------------------------------------- 484
Tiger_snake_X1        NMQANR-------------------------------------- 507
Camel                 QMRANS-------------------------------------- 492
Goat                  QMRANS-------------------------------------- 458
Cow                   QMRVTFPSS----------------------------------- 493
Sheep                 QMRANS-------------------------------------- 490
Beluga_X1             QMRVTFPSSSLGSLASLCQSLGGVVPVPGGIPSPTVQGPPLVFG 497
Dolphin               QMRANS-------------------------------------- 490
Macaque               QMRADD-------------------------------------- 492
Human_V2              QMRADG-------------------------------------- 492
Human_V1              QMRADG-------------------------------------- 529
Gorilla_X1            QMRVTILSSF---------------------------------- 496
Gorilla_X2            QMRADG-------------------------------------- 492
Mouse                 QMRANS-------------------------------------- 490
Rat                   QMRANS-------------------------------------- 490
Elephant              QMRVISPTAPPP-------------------------------- 497
Dog                   QMRANS-------------------------------------- 487
Rabbit                QMRAHS-------------------------------------- 488
Horse_X1              EMRANS-------------------------------------- 490
Horse_X2              EMRANS-------------------------------------- 490
Lemur                 QMKANS-------------------------------------- 545
Ground_Squirrel       QMRVTSCPFHWVFVSLR---GGQLLLWEAG-------------- 512
Pig                   QMRANS-------------------------------------- 496
Tiger                 QMRANG-------------------------------------- 518
Grey_Seal_X1          QMRANS-------------------------------------- 492
Bear                  QMRANS-------------------------------------- 492
Ferret                QMRANS-------------------------------------- 551
Cat_X1                QMRANG-------------------------------------- 492
Cat_X2                QMRANG-------------------------------------- 492
Cat_X3                QMRANG-------------------------------------- 492
Bat                   QMKANS-------------------------------------- 492
Bat_Little_Brown      QMKANS-------------------------------------- 243
                      .*.
```

Figure S2. Amino acid sequence alignment of TMPRSS2 polypeptides from a range of animal species. Accession Numbers of the polypeptide sequences used are given in Table 1. Alignments were carried out using Clustal Omega [1]. Human sequence is highlighted in grey to aid comparison to other sequences. D435, important for the catalytic site of the enzyme, is highlighted in blue [4]. The V160 is highlighted green. Other polymorphisms reported by Hou et al [4] are in purple. Amino acids involved in the TMPRSS2/SARS-CoV-2 interactions are highlighted in yellow [5].

```
CLUSTAL O(1.2.4) multiple sequence alignment

Cod_X1                   ------------------------------------------------------------ 0
Frog                     ------------------------------------------------------------ 0
Tiger_snake_X1           ------------------------------------------------------------ 0
Rock_Dove                ------------------------------------------------------------ 0
Chicken_X1               ------------------------------------------------------------ 0
Duck                     ------------------------------------------------------------ 0
Rabbit                   ------------------------------------------------------------ 0
Rat                      ------------------------------------------------------------ 0
Mouse                    ------------------------------------------------------------ 0
Bat_Little_Brown         ------------------------------------------------------------ 0
Elephant                 ------------------------------------------------------------ 0
Lemur_X1                 ------------------------------------------------------------ 0
Ground_Squirrel_X1       ------------------------------------------------------------ 0
Macaque_X1               ------------------------------------------------------------ 0
Gorilla_X1               ------------------------------------------------------------ 0
Human_X1                 ------------------------------------------------------------ 0
Human_X6                 ------------------------------------------------------------ 0
Camel_X1                 ------------------------------------------------------------ 0
Bat_Pipistrellus_X1      ------------------------------------------------------------ 0
Beluga_X1                ------------------------------------------------------------ 0
Dolphin_X1               ------------------------------------------------------------ 0
Cow                      ------------------------------------------------------------ 0
Goat                     ------------------------------------------------------------ 0
Sheep                    ------------------------------------------------------------ 0
Pig                      ------------------------------------------------------------ 0
Horse_X1                 ------------------------------------------------------------ 0
Horse_X2                 ------------------------------------------------------------ 0
Dog_X1                   ------------------------------------------------------------ 0
Dog_X2                   ------------------------------------------------------------ 0
American_mink            ------------------------------------------------------------ 0
Ferret_X1                ------------------------------------------------------------ 0
Tiger                    MTNKRNPSPLTTACSRDGHINQADPSGFYGRCSDLFTSRTNSLGGGSEGIYPLKGLIGGR 60
Cat_X4                   ------------------------------------------------------------ 0
Cat_X3                   ------------------------------------------------------------ 0
Cat_X1                   ------------------------------------------------------------ 0
Cat_X2                   ------------------------------------------------------------ 0
Grey_Seal_X1             ------------------------------------------------------------ 0
Bear                     ------------------------------------------------------------ 0
```

```
Cod_X1                  ------------------------------------------------------------ 0
Frog                    ------------------------------------------------------------ 0
Tiger_snake_X1          ------------------------------------------------------------ 0
Rock_Dove               ------------------------------------------------------------ 0
Chicken_X1              ------------------------------------------------------------ 0
Duck                    ------------------------------------------------------------ 0
Rabbit                  ------------------------------------------------------------ 0
Rat                     ------------------------------------------------------------ 0
Mouse                   ------------------------------------------------------------ 0
Bat_Little_Brown        ------------------------------------------------------------ 0
Elephant                ------------------------------------------------------------ 0
Lemur_X1                ------------------------------------------------------------ 0
Ground_Squirrel_X1      ------------------------------------------------------------ 0
Macaque_X1              ------------------------------------------------------------ 0
Gorilla_X1              ------------------------------------------------------------ 0
Human_X1                ------------------------------------------------------------ 0
Human_X6                ------------------------------------------------------------ 0
Camel_X1                ------------------------------------------------------------ 0
Bat_Pipistrellus_X1     ------------------------------------------------------------ 0
Beluga_X1               ------------------------------------------------------------ 0
Dolphin_X1              ------------------------------------------------------------ 0
Cow                     ------------------------------------------------------------ 0
Goat                    ------------------------------------------------------------ 0
Sheep                   ------------------------------------------------------------ 0
Pig                     ------------------------------------------------------------ 0
Horse_X1                ------------------------------------------------------------ 0
Horse_X2                ------------------------------------------------------------ 0
Dog_X1                  ------------------------------------------------------------ 0
Dog_X2                  ------------------------------------------------------------ 0
American_mink           ------------------------------------------------------------ 0
Ferret_X1               ------------------------------------------------------------ 0
Tiger                   NVCSLFLLSISSEALCSSSGKAFGDGTGGHPVHSEDAPPREVGVDRSWRTGMTIACGLGR 120
Cat_X4                  ------------------------------------------------------------ 0
Cat_X3                  ------------------------------------------------------------ 0
Cat_X1                  ------------------------------------------------------------ 0
Cat_X2                  ------------------------------------------------------------ 0
Grey_Seal_X1            ------------------------------------------------------------ 0
Bear                    ------------------------------------------------------------ 0
```

```
Cod_X1                   ------------------------------------------------------------ 0
Frog                     ------------------------------------------------------------ 0
Tiger_snake_X1           ------------------------------------------------------------ 0
Rock_Dove                ------------------------------------------------------------ 0
Chicken_X1               ------------------------------------------------------------ 0
Duck                     ------------------------------------------------------------ 0
Rabbit                   ------------------------------------------------------------ 0
Rat                      ------------------------------------------------------------ 0
Mouse                    ------------------------------------------------------------ 0
Bat_Little_Brown         ------------------------------------------------------------ 0
Elephant                 ------------------------------------------------------------ 0
Lemur_X1                 ------------------------------------------------------------ 0
Ground_Squirrel_X1       ------------------------------------------------------------ 0
Macaque_X1               ------------------------------------------------------------ 0
Gorilla_X1               ------------------------------------------------------------ 0
Human_X1                 ------------------------------------------------------------ 0
Human_X6                 ------------------------------------------------------------ 0
Camel_X1                 ------------------------------------------------------------ 0
Bat_Pipistrellus_X1      ------------------------------------------------------------ 0
Beluga_X1                ------------------------------------------------------------ 0
Dolphin_X1               ------------------------------------------------------------ 0
Cow                      ------------------------------------------------------------ 0
Goat                     ------------------------------------------------------------ 0
Sheep                    ------------------------------------------------------------ 0
Pig                      ------------------------------------------------------------ 0
Horse_X1                 ------------------------------------------------------------ 0
Horse_X2                 ------------------------------------------------------------ 0
Dog_X1                   ------------------------------------------------------------ 0
Dog_X2                   ------------------------------------------------------------ 0
American_mink            ------------------------------------------------------------ 0
Ferret_X1                ------------------------------------------------------------ 0
Tiger                    APCEASQEPSYPATVLPSSRGTETGAPGLLIANPREHLGSTETRVWAGSGDGCPLAEGAP 180
Cat_X4                   ------------------------------------------------------------ 0
Cat_X3                   ------------------------------------------------------------ 0
Cat_X1                   ------------------------------------------------------------ 0
Cat_X2                   ------------------------------------------------------------ 0
Grey_Seal_X1             ------------------------------------------------------------ 0
Bear                     ------------------------------------------------------------ 0
```

```
Cod_X1                   ------------------------------------------------------------ 0
Frog                     ------------------------------------------------------------ 0
Tiger_snake_X1           ------------------------------------------------------------ 0
Rock_Dove                ------------------------------------------------------------ 0
Chicken_X1               ------------------------------------------------------------ 0
Duck                     ------------------------------------------------------------ 0
Rabbit                   ------------------------------------------------------------ 0
Rat                      ------------------------------------------------------------ 0
Mouse                    ------------------------------------------------------------ 0
Bat_Little_Brown         ------------------------------------------------------------ 0
Elephant                 ------------------------------------------------------------ 0
Lemur_X1                 ------------------------------------------------------------ 0
Ground_Squirrel_X1       ------------------------------------------------------------ 0
Macaque_X1               ------------------------------------------------------------ 0
Gorilla_X1               ------------------------------------------------------------ 0
Human_X1                 ------------------------------------------------------------ 0
Human_X6                 ------------------------------------------------------------ 0
Camel_X1                 ------------------------------------------------------------ 0
Bat_Pipistrellus_X1      ------------------------------------------------------------ 0
Beluga_X1                ------------------------------------------------------------ 0
Dolphin_X1               ------------------------------------------------------------ 0
Cow                      ------------------------------------------------------------ 0
Goat                     ------------------------------------------------------------ 0
Sheep                    ------------------------------------------------------------ 0
Pig                      ------------------------------------------------------------ 0
Horse_X1                 ------------------------------------------------------------ 0
Horse_X2                 ------------------------------------------------------------ 0
Dog_X1                   ------------------------------------------------------------ 0
Dog_X2                   ------------------------------------------------------------ 0
American_mink            ------------------------------------------------------------ 0
Ferret_X1                ------------------------------------------------------------ 0
Tiger                    LPTLPRSLCPAGEGPRSELTMEPPVRGGHPGTTGARQRALLRDREAVCGCSTASPARIPR 240
Cat_X4                   ------------------------------------------------------------ 0
Cat_X3                   ------------------------------------------------------------ 0
Cat_X1                   ------------------------------------------------------------ 0
Cat_X2                   ------------------------------------------------------------ 0
Grey_Seal_X1             ------------------------------------------------------------ 0
Bear                     ------------------------------------------------------------ 0
```

```
Cod_X1                    ----------------------MYCGLDFILLLGFVLAVRAFKNDKCGDNIRITKANYLT 38
Frog                      -----------------MLLRLLSCCCW-LL--CSLRSSWASRNDKCGDTIKITSPSYLT 40
Tiger_snake_X1            -----------------MAWGLLLHSAA-----LIVAFGKALKSDRCGENIQILEPGYLT 38
Rock_Dove                 ------------------------------------------------------------ 0
Chicken_X1                -----------------MDWGLFLHCAA-L----TFTLSRALRSDKCGDTIKILSPGYLT 38
Duck                      -----------------MDWGLFLHCAA-L----TFTLAGAPRSDKCGDTIKILNPGYLT 38
Rabbit                    ------------------MERAPLLCAA-LA--LALAAAGAFRSDKCGDTIKIESPGYLT 39
Rat                       -----------------MERGLPLLCAT-LA--LALALAGAFRSDKCGGTIKIENPGYLT 40
Mouse                     -----------------MERGLPLLCAT-LA--LALALAGAFRSDKCGGTIKIENPGYLT 40
Bat_Little_Brown          -----------------MERRLLLFGAA-L--TLALAQASAFRNDKCGNTIKIENPGYLT 40
Elephant                  -----------------MERGLLLLCAA-L----ALALASAFRNDKCGGTIKIENPGYLT 38
Lemur_X1                  -----------------MESGLPLLCAA-LALALAL--AGAFRSDKCGDTIKIESPGYLT 40
Ground_Squirrel_X1        -----------------MERGLPLLCAA-LALALAL--TSAFRNDKCGGTIKIESPGYLT 40
Macaque_X1                -----------------MEKGLPLLCAA-LALALALAPAGAFRNDKCGDTIKIESPGYLT 42
Gorilla_X1                -----------------MERGLPLLCAV-LA--LVLAPAGAFRNDKCGDTIKIESPGYLT 40
Human_X1                  -----------------MERGLPLLCAV-LA--LVLAPAGAFRNDKCGDTIKIESPGYLT 40
Human_X6                  ------------------------------------------------------------ 0
Camel_X1                  -----------------MERGLPLLCAA-LA--LAVAAAAAFRNDKCGDTIKIESPGYLT 40
Bat_Pipistrellus_X1       -----------------MERGLLLLGAA-LA--LALAQAGAFRNDKCGNTIKIENPGYLT 40
Beluga_X1                 -----------------MERGLPLLCAA-LA--LVLSAAGAFRNDKCGDTIKIESPGYLT 40
Dolphin_X1                -----------------MERGLPLLCAA-LA--LVLSAAGAFRNDKCGDTIKIESPGYLT 40
Cow                       -----------------MEKGLQLLCAA-LA--LVLGAAGAFRNDKCGDTIKIESPGYLT 40
Goat                      -----------------MEKGLQLLCAA-LA--LVLGAAGAFRNDKCGDTIKIESPGYLT 40
Sheep                     -----------------MEKGLQLLCAA-LA--LVLGAAGAFRNDKCGDTIKIESPGYLT 40
Pig                       -----------------MERGLPLLCAA-LA--LALGAAGALRNDKCGDTIKIESPGYLT 40
Horse_X1                  -----------------MERGLPLLCAA-L--ALALALAGAFRNDKCGDTIKIESPGYLT 40
Horse_X2                  -----------------MERGLPLLCAA-L--ALALALAGAFRNDKCGDTIKIESPGYLT 40
Dog_X1                    -----------------MERGPPLLSAA-LAVAVAVALAGAFRNDKCGDTIKIESPGYLT 42
Dog_X2                    -----------------MERGPPLLSAA-LAVAVAVALAGAFRNDKCGDTIKIESPGYLT 42
American_mink             -----------------MERGLPLLCAA-LA--VALAPASAFRNDKCGDTIKIESPGYLT 40
Ferret_X1                 -----------------MERGLPLLCAA-LA--VALALASAFRNDKCGDTIKIESPGYLT 40
Tiger                     FALFSPHVLVLPRLPGDPAKGEWRGGCA-LA--VAFAAASAFRNDKCGDTIKIESPGYLT 297
Cat_X4                    -----------------MERGLPLLCAA-LA--VAFAPASAFRNDKCGDTIKIESPGYLT 40
Cat_X3                    -----------------MERGLPLLCAA-LA--VAFAPASAFRNDKCGDTIKIESPGYLT 40
Cat_X1                    -----------------MERGLPLLCAA-LA--VAFAPASAFRNDKCGDTIKIESPGYLT 40
Cat_X2                    -----------------MERGLPLLCAA-LA--VAFAPASAFRNDKCGDTIKIESPGYLT 40
Grey_Seal_X1              -----------------MERGLPLLCAA-LA--VALAPASAFRNDKCGDTIKIESPGYLT 40
Bear                      -----------------MERGLPLLSAA-LA--VALALASAFRNDKCGDTIKIESPGYLT 40
```

```
Cod_X1                SPGYPASYSPSHKCVWVITAPGPHQRILINFNPHFDLEDRECKYDYVEVRDGVDESGQLV 98
Frog                  SAGYPHSYPPSQRCEWLIQAPEHYQRIMINFNPHFDLEDRECKYDYVEVIDGDNANGQLL 100
Tiger_snake_X1        SPGYPNSYHPSQKCEWLIQAPQPHQRIMINFNPHFDLEDRECKYDYVEVIDGDSANGRVW 98
Rock_Dove             -------------------------------MERRATTVTQMLYDYVEVIDGDNAEGRLW 29
Chicken_X1            SPGYPQSYHPSQKCEWLIQAPEPYQRIMINFNPHFDLEDRDCKYDYVEVIDGDNAEGRLW 98
Duck                  SPGYPQSYHPSQKCEWLIQAPEPYQRIMINFNPHFDLEDRDCKYDYVEVIDGDNAEGRLW 98
Rabbit                SPGYPHSYHPSEKCEWLLQAPDPYQRILINFNPPFDLEDRDCKYDYVEVIDGESEHGRPA 99
Rat                   SPGYPHSYHPSEKCEWLIQAPEPYQRIMINFNPHFDLEDRDCKYDYVEVIDGENEGGRLW 100
Mouse                 SPGYPHSYHPSEKCEWLIQAPEPYQRIMINFNPHFDLEDRDCKYDYVEVIDGENEGGRLW 100
Bat_Little_Brown      SPGYPHSYHPSEKCEWLIQAPEPYQRIMINFNPHFDLEDRDCKYDYVEVIDGENEDGRLW 100
Elephant              SPGYPHSYHPSEKCEWLIQAPYPYQRIMINFNPHFDLEDRDCKYDYVEVIDGDNPNGRLW 98
Lemur_X1              SPGYPHSYHPSEKCEWLIEAPDPYQRIMINFNPHFDLEDRDCKYDYVEVIDGRNENGRLW 100
Ground_Squirrel_X1    SPGYPHSYHPSEKCEWLIQAPDQYQRIMINFNPHFDLEDRDCKYDYVEVIDGENENGRPW 100
Macaque_X1            SPGYPHSYHPSEKCEWLIQAPDPYQRIMINFNPHFDLEDRDCKYDYVEVFDGENENGRLW 102
Gorilla_X1            SPGYPHSYHPSEKCEWLIQAPDPYQRIMINFNPHFDLEDRDCKYDYVEVFDGENENGHFR 100
Human_X1              SPGYPHSYHPSEKCEWLIQAPDPYQRIMINFNPHFDLEDRDCKYDYVEVFDGENENGHFR 100
Human_X6              ------------------------------------------------------------ 0
Camel_X1              SPGYPHSYHPSEKCEWLIQAPDPYQRIMINFNPHFDLEDRDCKYDYVEVIDGENENGRLW 100
Bat_Pipistrellus_X1   SPGYPHSYHPSEKCEWLIQAPEPYQRIMINFNPHFDLEDRDCKYDYVEVIDGENESGRLW 100
Beluga_X1             SPGYPHSYHPSEKCEWLIQAPDPYQRIMINFNPHFDLEDRDCKYDYVEVIDGENENGRLW 100
Dolphin_X1            SPGYPHSYHPSEKCEWLIQAPDPYQRIMINFNPHFDLEDRDCKYDYVEVIDGENENGRLW 100
Cow                   SPGYPHSYHPSEKCEWLIQAPDPYQRIMINFNPHFDLEDRDCKYDYVEVIDGENESGRVL 100
Goat                  SPGYPHSYHPSEKCEWLIQAPDPYQRIMINFNPHFDLEDRDCKYDYVEVIDGENENGRVL 100
Sheep                 SPGYPHSYHPSEKCEWLIQAPDPYQRIMINFNPHFDLEDRDCKYDYVEVIDGENENGRVL 100
Pig                   SPGYPHSYHSSEKCEWLIQAPEPYQRIMINFNPHFDLEDRDCKYDYVEVIDGENENGRLW 100
Horse_X1              SPGYPHSYHPSEKCEWLIQAPDPYQRIMINFNPHFDLEDRDCKYDYVEVIDGENENGRLW 100
Horse_X2              SPGYPHSYHPSEKCEWLIQAPDPYQRIMINFNPHFDLEDRDCKYDYVEVIDGENENGRLW 100
Dog_X1                SPGYPHSYHPSEKCEWLIQAPDPYQRIMINFNPHFDLEDRDCKYDYVEVIDGENENGRPW 102
Dog_X2                SPGYPHSYHPSEKCEWLIQAPDPYQRIMINFNPHFDLEDRDCKYDYVEVIDGENENGRPW 102
American_mink         SPGYPHSYHPSEKCEWLIQAPDPYQRIMINFNPHFDLEDRDCKYDYVEVIDGENENGRLW 100
Ferret_X1             SPGYPHSYHPSEKCEWLIQAPDPYQRIMINFNPHFDLEDRDCKYDYVEVIDGENENGRLW 100
Tiger                 SPGYPHSYHPSEKCEWLIQAPDPYQRIMINFNPHFDLEDRDCKYDYVEVIDGDNENGRLW 357
Cat_X4                SPGYPHSYHPSEKCEWLIQAPDPYQRIMINFNPHFDLEDRDCKYDYVEVIDGDNENGRLW 100
Cat_X3                SPGYPHSYHPSEKCEWLIQAPDPYQRIMINFNPHFDLEDRDCKYDYVEVIDGDNENGRLW 100
Cat_X1                SPGYPHSYHPSEKCEWLIQAPDPYQRIMINFNPHFDLEDRDCKYDYVEVIDGDNENGRLW 100
Cat_X2                SPGYPHSYHPSEKCEWLIQAPDPYQRIMINFNPHFDLEDRDCKYDYVEVIDGDNENGRLW 100
Grey_Seal_X1          SPGYPHSYHPSEKCEWLIQAPDPYQRIMINFNPHFDLEDRDCKYDYVEVIDGENENGRLW 100
Bear                  SPGYPHSYHPSEKCEWLIRAPDPYQRIMINFNPHFDLEDRDCKYDYVEVIDGENENGRLW 100
```

```
Cod_X1                   GKYCGKIAPSPVISSGNQLFIKFVSDYETHGAGFSIRYEIFKTGPECSRNFTSNSGVIKS 158
Frog                     GKYCGKIAPSPLVSTGPSIFIRFVSDYETPGAGFSIRYEVFKTGPECSRNFTSSNGVIKS 160
Tiger_snake_X1           GKFCGKIAPPPVISTGPYLLIKFVSDYETHGAGFSIRYEIFKRGPECSRNFTSMTGMIKS 158
Rock_Dove                GKYCGKIAPPPLVSSGPYLFIKFVSDYETHGAGFSIRYEVFKRGPECSRNFTSSSGVIKS 89
Chicken_X1               GKYCGKIAPPPLVSSGPYLFIKFVSDYETHGAGFSIRYEVFKRGPECSRNFTSSSGMIKS 158
Duck                     GKYCGKIAPPPLVSSGPYLFIKFVSDYETHGAGFSIRYEVFKRGPECSRNFTSSSGVIKS 158
Rabbit                   GKFCGKIAPPPVVSSGPFLFVRFVSDYETHGAGFSIRYEIYKRGPECSQNYTSPSGVIKS 159
Rat                      GKFCGKIAPSPVVSSGPFLFIKFVSDYETHGAGFSIRYEIFKRGPECSQNYTAPTGVIKS 160
Mouse                    GKFCGKIAPSPVVSSGPFLFIKFVSDYETHGAGFSIRYEIFKRGPECSQNYTAPTGVIKS 160
Bat_Little_Brown         GKFCGKIAPSPIVSSGPFLFIKFVSDYETHGAGFSIRYEIFKRGPECSQNYTSPSGVIKS 160
Elephant                 GKFCGKIAPSPVVSLGPFLFIRFVSDYETHGAGFSIRYEIFKRGPECSQNYTTPSGVIKS 158
Lemur_X1                 GKFCGKIAPSPVVSSGPFLFIRFVSDYETHGAGFSIRYEIFKRGPECSQNYTMSSGVIKS 160
Ground_Squirrel_X1       GKFCGKIAPSPVVSSGPFLFIKFVSDYETHGAGFSIRYEIFKRGPECSQNYTTSSGVIKS 160
Macaque_X1               GKFCGKIAPPPVVSSGQFLFIKFVSDYETHGAGFSIRYEIFKRGPECSQNYTTSSGVIKS 162
Gorilla_X1               GKFCGKIAPPPVVSSGPFLFIKFVSDYETHGAGFSIRYEIFKRGPECSQNYTTPSGVIKS 160
Human_X1                 GKFCGKIAPPPVVSSGPFLFIKFVSDYETHGAGFSIRYEIFKRGPECSQNYTTPSGVIKS 160
Human_X6                 ------------------------------------------------------------ 0
Camel_X1                 GKFCGKIAPSPVVSSGPFLFIKFVSDYETHGAGFSIRYEIFKRGPECSQNYTAPSGVIKS 160
Bat_Pipistrellus_X1      GKFCGKIAPSPIVSSGPFLFIKFVSDYETHGAGFSIRYEIFKRGPECSQNYTSPSGVIKS 160
Beluga_X1                GKFCGKIAPSPVVSSGPFLFIKFVSDYETHGAGFSIRYEIFKRGPECSQNYTTPSGVIKS 160
Dolphin_X1               GKFCGKIAPSPVVSSGPFLFIKFVSDYETHGAGFSIRYEIFKRGPECSQNYTTPSGVIKS 160
Cow                      GKFCGKIAPSSFVSSGPFLFIKFVSDYETHGAGFSIRYEIFKKGPECSQNYTMPSGIIKS 160
Goat                     GKFCGKIAPSSFVSSGPFLFIKFVSDYETHGAGFSIRYEIFKKGPECSQNYTMPSGVIKS 160
Sheep                    GKFCGKIAPSSFVSSGPFLFIKFVSDYETHGAGFSIRYEIFKKGPECSQNYTMPSGVIKS 160
Pig                      GKFCGKIAPSPVVSSGPFLFIKFVSDYETHGAGFSIRYEIFKRGPECSQNYTTPSGVIKS 160
Horse_X1                 GKFCGKIAPSPIVSSGPFLFIKFVSDYETHGAGFSIRYEIFKRGPECSQNYTTPSGVIKS 160
Horse_X2                 GKFCGKIAPSPIVSSGPFLFIKFVSDYETHGAGFSIRYEIFKRGPECSQNYTTPSGVIKS 160
Dog_X1                   GKFCGKIAPSPVVSSGPFLFIKFVSDYETHGAGFSIRYEIFKRGPECSQNYTAPSGVIKS 162
Dog_X2                   GKFCGKIAPSPVVSSGPFLFIKFVSDYETHGAGFSIRYEIFKRGPECSQNYTAPSGVIKS 162
American_mink            GKFCGKIAPSPVVSSGPFLFIKFVSDYETHGAGFSIRYEIFKRGPECSQNYTASSGVIKS 160
Ferret_X1                GKFCGKIAPSPVVSSGPFLFIKFVSDYETHGAGFSIRYEIFKRGPECSQNYTASSGVIKS 160
Tiger                    GKFCGKIAPSPVVSSGPFLFIKFVSDYETHGAGFSIRYEIFKRGPECSQNYTAPSGVIKS 417
Cat_X4                   GKFCGKIAPSPVVSSGPFLFIKFVSDYETHGAGFSIRYEIFKRGPECSQNYTAPSGVIKS 160
Cat_X3                   GKFCGKIAPSPVVSSGPFLFIKFVSDYETHGAGFSIRYEIFKRGPECSQNYTAPSGVIKS 160
Cat_X1                   GKFCGKIAPSPVVSSGPFLFIKFVSDYETHGAGFSIRYEIFKRGPECSQNYTAPSGVIKS 160
Cat_X2                   GKFCGKIAPSPVVSSGPFLFIKFVSDYETHGAGFSIRYEIFKRGPECSQNYTAPSGVIKS 160
Grey_Seal_X1             GKFCGKIAPSPVVSSGPFLFIKFVSDYETHGAGFSIRYEIFKRGPECSQNYTAPNGVIKS 160
Bear                     GKFCGKIAPSPVVSSGPFLFIKFVSDYETHGAGFSIRYEIFKRGPECSQNYTAPSGVIKS 160
```

```
Cod_X1                   PGFPEKYPNNLDCTFMIFAPKMSEIVVEFESFELEPDTTPPTGVFCRYDRLEIWDGFPGV 218
Frog                     PKYPEKYPNALECTYIIFAPKMQEIVLEFESFELEADSNAPGGQTCRYDWLGIWDGFPGV 220
Tiger_snake_X1           PGFPEKYPNSLECTYIIFAPKMSEIILEFESFELEPDTNPPGGAFCRYDRLEVWEGFPEV 218
Rock_Dove                PGFPEKYPNSLECTYIIFAPKMSEIILEFESFELEPDSNTPGGAFCRYDRLEIWDGFPDV 149
Chicken_X1               PGFPEKYPNSLECTYIIFAPKMSEIILEFESFELEPDSNTPGGAFCRYDRLEIWDGFPDV 218
Duck                     PGFPEKYPNSLECTYIIFAPKMSEIILEFESFELEPDSNTPGGAFCRYDRLEIWDGFPDV 218
Rabbit                   PGFPEKYPNSLECTYIIFAPKMSEIILEFESFDLEPDSNPPGGMFCRYDRLEIWDGFPDV 219
Rat                      PGFPEKYPNSLECTYIIFAPKMSEIILEFESFDLEQDSNPPGGMFCRYDRLEIWDGFPEV 220
Mouse                    PGFPEKYPNSLECTYIIFAPKMSEIILEFESFDLEQDSNPPGGMFCRYDRLEIWDGFPEV 220
Bat_Little_Brown         PGFPEKYPNSLECTYIIFAPKMSEIILEFESFDLEPDSNSPGGMFCRYDRLEIWDGFPDV 220
Elephant                 PGFPEKYPNSLECTYIIFAPKMSEILLEFESFDLEPDSNPPGGIFCRYDRLEIWDGFPEV 218
Lemur_X1                 PGFPEKYPNSLECTYIIFAPKMSEIILEFESFDLEPDSNPPGGIFCRYDRLEIWDGFPEV 220
Ground_Squirrel_X1       PGFPEKYPNGLECTYIIFAPKMSEIILEFESFDLEPDSNPPGGMFCRYDRLEIWDGFPDV 220
Macaque_X1               PGFPEKYPNSLECTYIVFAPKMSEIILEFESFDLEPDSNPPGGMFCRYDRLEIWDGFPDV 222
Gorilla_X1               PGFPEKYPNSLECTYIVFAPKMSEIILEFESFDLEPDSNPPGGMFCRYDRLEIWDGFPDV 220
Human_X1                 PGFPEKYPNSLECTYIVFVPKMSEIILEFESFDLEPDSNPPGGMFCRYDRLEIWDGFPDV 220
Human_X6                 ---------------------MSEIILEFESFDLEPDSNPPGGMFCRYDRLEIWDGFPDV 39
Camel_X1                 PGFPEKYPNSLECTYIIFAPKMSEIILEFESFDLEPDSNPPGGMICRYDRLEIWDGFPNV 220
Bat_Pipistrellus_X1      PGFPEKYPNSLECTYIIFAPKMSEIILEFESFDLELDSNAPGGMFCRYDRLEIWDGFPDV 220
Beluga_X1                PGFPEKYPNSLECTYIIFAPKMSEIILEFESFDLEADSNPPGGMFCRYDRLEIWDGFPDV 220
Dolphin_X1               PGFPEKYPNSLECTYIIFAPKMSEIILEFESFDLEADSNPPGGMFCRYDRLEIWDGFPDV 220
Cow                      PGFPEKYPNSLECTYIIFAPKMSEIILEFESFDLEPDSNPPGGMFCRYDRLEIWDGFPDV 220
Goat                     PGFPEKYPNSLECTYIIFAPKMSEIILEFESFDLEPDSNPPGGVFCRYDRLEIWDGFPDV 220
Sheep                    PGFPEKYPNSLECTYIIFAPKMSEIILEFESFDLEPDSNPPGGVFCRYDRLEIWDGFPDV 220
Pig                      PGFPEKYPNSLECTYIIFAPKMSEIILEFESFDLEPDSNPPGGMFCRYDRLEIWDGFPDV 220
Horse_X1                 PGFPEKYPNSLECTYIIFAPKMSEIILEFESFDLEPDSNPPGGMFCRYDRLEIWDGFPDV 220
Horse_X2                 PGFPEKYPNSLECTYIIFAPKMSEIILEFESFDLEPDSNPPGGMFCRYDRLEIWDGFPDV 220
Dog_X1                   PGFPEKYPNGLECTYIIFAPKMSEIILEFESFDLELDSNPPGGMFCRYDRLEIWDGFPDV 222
Dog_X2                   PGFPEKYPNGLECTYIIFAPKMSEIILEFESFDLELDSNPPGGMFCRYDRLEIWDGFPDV 222
American_mink            PGFPEKYPNGLECTYIIFAPKMSEIILEFESFDLELDSNPPGGMFCRYDRLEIWDGFPDV 220
Ferret_X1                PGFPEKYPNGLECTYIIFAPKMSEIILEFESFDLELDSNPPGGMFCRYDRLEIWDGFPDV 220
Tiger                    PGFPEKYPNSLECTYIIFAPKMSEIILEFESFDLELDSNPPGGMFCRYDRLEIWDGFPDV 477
Cat_X4                   PGFPEKYPNSLECTYIIFAPKMSEIILEFESFDLELDSNPPGGMFCRYDRLEIWDGFPDV 220
Cat_X3                   PGFPEKYPNSLECTYIIFAPKMSEIILEFESFDLELDSNPPGGMFCRYDRLEIWDGFPDV 220
Cat_X1                   PGFPEKYPNSLECTYIIFAPKMSEIILEFESFDLELDSNPPGGMFCRYDRLEIWDGFPDV 220
Cat_X2                   PGFPEKYPNSLECTYIIFAPKMSEIILEFESFDLELDSNPPGGMFCRYDRLEIWDGFPDV 220
Grey_Seal_X1             PGFPEKYPNSLECTYIIFAPKMSEIILEFESFDLELDSNPPGGMFCRYDRLEIWDGFPDV 220
Bear                     PGFPEKYPNSLECTYIIFAPKMSEIILEFESFDLELDSNPPGGMFCRYDRLEIWDGFPDV 220
                                              *.**::*****:** *:. * *  **** * :*:*** *
```

```
Cod_X1                   GPYVGRYCGQNMPGRIISYTGILALTIFTDSAIAKEGFSANFTVIERTVPEDFDCTDPLG 278
Frog                     GPHIGRYCGQNTPGRVRSFTGILSMIFHTDSAIAKEGFFANFSVVQSNTDEDFQCKEPLG 280
Tiger_snake_X1           GPHTGRYCGQNTPGRVLSSTGILSMVFYTDSAIAKEGFSANYTVVHNNVPKDFQCMEPLG 278
Rock_Dove                GPHIGRYCGQNNPGRVRSSTGILSMVFYTDSAIAKEGFSANYSVSQSSVSEDFQCMEPLG 209
Chicken_X1               GPHIGRYCGQNNPGRVRSSTGILSMVFYTDSAIAKEGFSANYSVSQSSVSEDFQCMEPLG 278
Duck                     GPHIGRYCGQNNPGRVRSSTGILSMVFYTDSAIAKEGFSANYSVSQSSVSEDFQCMEPLG 278
Rabbit                   GPHIGRYCGQKTPGRIRASSGILSMVFYTDSAIAKEGFSANYSVLQGSVAEDFKCMEALG 279
Rat                      GPHIGRYCGQKTPGRIRSSSGILSMVFYTDSAIAKEGFSANYSVLQSSISEDFKCMEALG 280
Mouse                    GPHIGRYCGQKTPGRIRSSSGVLSMVFYTDSAIAKEGFSANYSVLQSSISEDFKCMEALG 280
Bat_Little_Brown         GPHIGRYCGQKTPGRVRSSSGILSMVFYTDSAIAKEGFSANYSVLQSSLSEDFKCMEALG 280
Elephant                 GPHIGRYCGQKTPGRIRSSSGILSMVFYTDSAIAKEGFSANYSVLQSSVSEDFKCMEALG 278
Lemur_X1                 GPHIGRYCGQKTPGRIRSSSGILSMVFYTDSAIAKEGFSANYSVLQSSVSEDFKCMEALG 280
Ground_Squirrel_X1       GPHIGRYCGQKTPGRIRSSSGILSMVFYTDSAIAKEGFSANYSVLQSSVSEDFKCMEALG 280
Macaque_X1               GPHIGRYCGQKTPGRIRSSSGILSMVFYTDSAIAKEGFSANYSVLQSSVSEDFKCMEAMG 282
Gorilla_X1               GPHIGRYCGQKTPGRIRSSSGILSMVFYTDSAIAKEGFSANYSVLQSSVSEDFKCMEALG 280
Human_X1                 GPHIGRYCGQKTPGRIRSSSGILSMVFYTDSAIAKEGFSANYSVLQSSVSEDFKCMEALG 280
Human_X6                 GPHIGRYCGQKTPGRIRSSSGILSMVFYTDSAIAKEGFSANYSVLQSSVSEDFKCMEALG 99
Camel_X1                 GPHIGHYCGQKTPGRIRASSGILSMVFYTDSAIAKEGFSANYSVLQSSISEDFKCMEALG 280
Bat_Pipistrellus_X1      GPHIGRYCGQKTPGRVRSSSGILSMVFYTDSAIAKEGFSANYSTLQSSLSEDFKCMEALG 280
Beluga_X1                GPHIGRYCGQKTPGRIHSSSGILSMVFYTDSAIAKEGFSANYSVSQSSVSEDFKCMEALG 280
Dolphin_X1               GPHIGRYCGQKTPGRIHSSSGILSMVFYTDSAIAKEGFSANYTVSQSSVSEDFKCMEALG 280
Cow                      GPHIGRYCGQKTPGRILSSSGILSMLFYTDSAIAKEGFSANYSVLQSSVSEDFKCMEALG 280
Goat                     GPHIGRYCGQKTPGRILSSSGILSMVFYTDSAIAKEGFSANYSVLQSSVSEDFKCMEALG 280
Sheep                    GPHIGRYCGQKTPGRILSSSGILSMVFYTDSAIAKEGFSANYSVLQSSVSEDFKCMEALG 280
Pig                      GPHIGRYCGQKTPGRVRSSSGILSMVFYTDSAIAKEGFSANYSVLQSSVSEDFKCMEALG 280
Horse_X1                 GPHIGRYCGQKTPGRIRSSSGILSMVFYTDSAIAKEGFSANYSILQSGVSEDFRCMEALG 280
Horse_X2                 GPHIGRYCGQKTPGRIRSSSGILSMVFYTDSAIAKEGFSANYSILQSGVSEDFRCMEALG 280
Dog_X1                   GPHIGRYCGQKTPGRIRSSSGILSMVFYTDSAIAKEGFSANYSVLQSSVSEDFKCMEALG 282
Dog_X2                   GPHIGRYCGQKTPGRIRSSSGILSMVFYTDSAIAKEGFSANYSVLQSSVSEDFKCMEALG 282
American_mink            GPHIGRYCGQKTPGRIRSSSGILSMVFYTDSAIAKEGFSANYSVLQSSVSEDFKCMEALG 280
Ferret_X1                GPHIGRYCGQKTPGRIRSSSGILSMVFYTDSAIAKEGFSANYSVLQSSVSEDFKCMEALG 280
Tiger                    GPHIGRYCGQKTPGRIRSSSGILSMVFYTDSAIAKEGFSANYSVLQSSVSEDFKCMEALG 537
Cat_X4                   GPHIGRYCGQKTPGRIRSSSGILSMVFYTDSAIAKEGFSANYSVLQSSVSEDFKCMEALG 280
Cat_X3                   GPHIGRYCGQKTPGRIRSSSGILSMVFYTDSAIAKEGFSANYSVLQSSVSEDFKCMEALG 280
Cat_X1                   GPHIGRYCGQKTPGRIRSSSGILSMVFYTDSAIAKEGFSANYSVLQSSVSEDFKCMEALG 280
Cat_X2                   GPHIGRYCGQKTPGRIRSSSGILSMVFYTDSAIAKEGFSANYSVLQSSVSEDFKCMEALG 280
Grey_Seal_X1             GPHIGRYCGQKTPGRIRSSSGILSMVFYTDSAIAKEGFSANYSVLQSSVSEDFKCMEALG 280
Bear                     GPHIGRYCGQKTPGRIRSSSGILSMVFYTDSAIAKEGFSANYSVLQSSVSEDFKCMEALG 280
                         **: *:****: ***: : :*:*:: :.********** **::  .    :** * : :*
```

```
Cod_X1                 LESGEIPSDQIVASSQYNPSWSPERSRLNYYENAWTPAEDSNKEWIQVDLGFLRFVSAIG 338
Frog                   MESGEIHFDQISVSSQYSMNWSAERSRLNYVENGWTPGEDTVKEWIQVDLENLRFVSGIG 340
Tiger_snake_X1         MESGEIHSDQITASSQYNPQWSSERSRLNYPENGWTPEIDSTREWIQVDVGVLRFVSAIG 338
Rock_Dove              MESGEIHSDQITVSSQYSAIWSAERSRLNYPENGWTPGEDSIREWIQVDLGLLRFVSGIG 269
Chicken_X1             MESGEIHSDQITVSSQYSAIWSSERSRLNYPENGWTPGEDSVREWIQVDLGLLRFVSGIG 338
Duck                   MESGEIHSDQITVSSQYSAIWSSERSRLNYPENGWTPGEDSAREWIQVDLSLLRFVSGIG 338
Rabbit                 MESGEIHSDQITASSQYSTNWSAERSRLNYPENGWTPGEDSYREWIQVDLGLLRFVTAVG 339
Rat                    MESGEIHSDQITASSQYGTNWSVERSRLNYPENGWTPGEDSYREWIQVDLGLLRFVTAVG 340
Mouse                  MESGEIHSDQITASSQYGTNWSVERSRLNYPENGWTPGEDSYKEWIQVDLGLLRFVTAVG 340
Bat_Little_Brown       MESGEIHSDQITASSQYSTNWSSERSRLNYPENGWTPGEDSYREWIQVDLGLLRFVTAVG 340
Elephant               MESGEIHSDQITASSQYSTNWSAERSRLNYPENGWTPGEDSYREWIQVDLGHLRFITAVG 338
Lemur_X1               MESGEIHSDQITASSQYSTNWSAERSRLNYPENGWTPGEDSYREWIQVDLGLLRFVTAIG 340
Ground_Squirrel_X1     MESGEIHSDQITASSQYSTNWSAERSRLNYPENGWTPGEDSYREWIQVDLGFLRFVTAVG 340
Macaque_X1             MESGEIHSDQITASSQYSTNWSAERSRLNYPENGWTPGEDSYREWIQVDLGLLRFVTAVG 342
Gorilla_X1             MESGEIHSDQITASSQYSTNWSAERSRLNYPENGWTPGEDSYREWIQVDLGLLRFVTAVG 340
Human_X1               MESGEIHSDQITASSQYSTNWSAERSRLNYPENGWTPGEDSYREWIQVDLGLLRFVTAVG 340
Human_X6               MESGEIHSDQITASSQYSTNWSAERSRLNYPENGWTPGEDSYREWIQVDLGLLRFVTAVG 159
Camel_X1               MESGEIHSDQITASSQYSTNWSAERSRLHYPENGWTPGEDSYREWIQVDLGLLRFVTAVG 340
Bat_Pipistrellus_X1    MESGEIHSDQITASSQYSTNWSSERSRLNYPENGWTPGEDSYREWIQVDLGLLRFVTAVG 340
Beluga_X1              MESGEIHSDQITASSQYSTNWSAERSRLHYPENGWTPGEDSYKEWIQVDLGLLRFVTAVG 340
Dolphin_X1             MESGEIHSDQITASSQYSTNWSAERSRLHYPENGWTPGEDSYKEWIQVDLGLLRFVTAVG 340
Cow                    MESGEIHSDQITASSQYSTNWSAERSRLHYPENGWTPGEDSYREWIQVDLGLLRFVTAVG 340
Goat                   MESGEIHSDQITASSQYSTNWSAERSRLHYPENGWTPGEDSYREWIQVDLGLLRFVTAVG 340
Sheep                  MESGEIHSDQITASSQYSTNWSAERSRLHYPENGWTPGEDSYREWIQVDLGLLRFVTAVG 340
Pig                    MESGEIHSDQITASSQYSTNWSAERSRLHYPENGWTPGEDSYREWIQVDLGLLRFVTAVG 340
Horse_X1               MESGEIHSDQITASSQYSTNWSAERSRLNYPENGWTPGEDSYREWIQVDLGLLRFVTAVG 340
Horse_X2               MESGEIHSDQITASSQYSTNWSAERSRLNYPENGWTPGEDSYREWIQVDLGLLRFVTAVG 340
Dog_X1                 MESGEIHSDQITASSQYSTNWSAERSRLNYPENGWTPGEDSYREWIQVDLGLLRFVTAVG 342
Dog_X2                 MESGEIHSDQITASSQYSTNWSAERSRLNYPENGWTPGEDSYREWIQVDLGLLRFVTAVG 342
American_mink          MESGEIHSDQITASSQYSTNWSAERSRLHYPENGWTPGEDSYKEWIQVDLGLLRFVTAVG 340
Ferret_X1              MESGEIHSDQITASSQYSTNWSAERSRLNYPENGWTPGEDSYKEWIQVDLGLLRFVTAVG 340
Tiger                  MESGEIHSDQITASSQYSTNWSAERSRLNYPENGWTPGEDSYREWIQVDLGLLRFVTAVG 597
Cat_X4                 MESGEIHSDQITASSQYSTNWSAERSRLNYPENGWTPGEDSYREWIQVDLGLLRFVTAVG 340
Cat_X3                 MESGEIHSDQITASSQYSTNWSAERSRLNYPENGWTPGEDSYREWIQVDLGLLRFVTAVG 340
Cat_X1                 MESGEIHSDQITASSQYSTNWSAERSRLNYPENGWTPGEDSYREWIQVDLGLLRFVTAVG 340
Cat_X2                 MESGEIHSDQITASSQYSTNWSAERSRLNYPENGWTPGEDSYREWIQVDLGLLRFVTAVG 340
Grey_Seal_X1           MESGEIHSDQITASSQYSTNWSAERSRLNYPENGWTPGEDSHREWIQVDLGLLRFVTAVG 340
Bear                   MESGEIHSDQITASSQYSTNWSAERSRLNYPENGWTPGEDSYREWIQVDLGLLRFVTAVG 340
                       :*****  *** .****.  ** *****:* **.***  *: :******:  ***::.:*
```

```
Cod_X1               TQGAVSQETKKTYYVKSYKVDVSSNGEDWITLKEGS-KQKVFQGNTNPKEVARAMLPKPT 397
Frog                 TQGAISKETKKKYFVKSYKVDISSNGEDWITLKDGN-KHLVFTGNTDATDVVYRPFSKPV 399
Tiger_snake_X1       TQGAISKETKKMYFLKTYRVDISSNGEDWISIKDGNNKPLLFMGNTNPTEVAYRSFPKPV 398
Rock_Dove            TQGAISKETKKEYYLKTYRVDVSSNGEDWITLKEGN-KPAVFQGNSNPTEVVYRPFAKPV 328
Chicken_X1           TQGAISKETKKEYYLKTYRVDVSSNGEDWITLKEGN-KPVVFQGNSNPTDVVYRPFPKPV 397
Duck                 TQGAISKETKKEYYLKTYRVDVSSNGEDWITLKEGN-KPVVFQGNSNPTDVVYRPFAKPV 397
Rabbit               TQGAISKETKKRYFVKTYRVDVSSNGEDWITLKEAN-KALIFQGNSNPTDVVFGIFPKPL 398
Rat                  TQGAISKETKKKYYVKTYRVDISSNGEDWITLKEGN-KAIIFQGNTNPTDVVFGVFPKPL 399
Mouse                TQGAISKETKKKYYVKTYRVDISSNGEDWISLKEGN-KAIIFQGNTNPTDVVLGVFSKPL 399
Bat_Little_Brown     TQGAISKETKKKYYVKTYRIDISSNGEDWITIKGGN-KPVVSLAAIPSTNLVFE------ 393
Elephant             TQGAISKETKKKYYVRTYRIDISSNGEDWITIKEGN-KPVIFQGNTNPTDVVFGIFPKPL 397
Lemur_X1             TQGAISKETKKKYYVKTYRIEISSNGEDWITIKEGN-KPVVFQGNTNPTDVVYGVFPKPL 399
Ground_Squirrel_X1   TQGAISKETKKKYYVKTYRIDISSNGEDWITIKEGN-KPVIFQGNTNPTDVALGILPKPL 399
Macaque_X1           TQGAISKETKKKYYVKTYKIDISSNGEDWITIKEGN-KPVLFQGNTNPTDVVVAVFPKPL 401
Gorilla_X1           TQGAISKETKKKYYVKTYKIDVSSNGEDWITIKEGN-KPVLFQGNTNPTDVVVAVFPKPL 399
Human_X1             TQGAISKETKKKYYVKTYKIDVSSNGEDWITIKEGN-KPVLFQGNTNPTDVVVAVFPKPL 399
Human_X6             TQGAISKETKKKYYVKTYKIDVSSNGEDWITIKEGN-KPVLFQGNTNPTDVVVAVFPKPL 218
Camel_X1             TQGAISKETKKKYYVKTYRIEISSNGEDWITIKEGN-KPLIFQGNTNPTDVVFGVFPKPL 399
Bat_Pipistrellus_X1  TQGAISKETKKKYYVTTYKIDISSNGEDWITIKEGN-KPVIFQGNTNPTDVVFGVFPKQL 399
Beluga_X1            TQGAISKETKKKYYVKTYRIDISSNGEDWITIKEGN-KPVIFQGNTNPTDVVIGVFPKPL 399
Dolphin_X1           TQGAISKETKKKYYVKTYRIDISSNGEDWITIKEGN-KPVIFQGNTNPTDVVIGVFPKPL 399
Cow                  TQGAISKETRKKYYVKTYRIDISSNGEDWITIKEGN-KPVIFHGNTNPTDVVIGVFPKPL 399
Goat                 TQGAISKETRRKYYVKTYRIDISSNGEDWITIKEGN-KPVIFQGNTNPTDVVIGVFPKPL 399
Sheep                TQGAISKETRRKYYVKTYRIDISSNGEDWITIKEGN-KPVIFQGNTNPTDVVIGVFPKPL 399
Pig                  TQGAISKETKKKYYVKTYRIDISSNGEDWITIKEGN-KPVIFQGNTNPTDVVFGVLPKPL 399
Horse_X1             TQGAISKETKKKYYVKTYRIDISSNGEDWITIKEGS-KPVIFQGNTNPTDVVFGVFPKPL 399
Horse_X2             TQGAISKETKKKYYVKTYRIDISSNGEDWITIKEGS-KPVIFQGNTNPTDVVFGVFPKPL 399
Dog_X1               TQGAISKETKKKYYVKTYRIDISSNGEDWITIKEGN-KPVIFQGNTNPTDVVFAVFPKPL 401
Dog_X2               TQGAISKETKKKYYVKTYRIDISSNGEDWITIKEGN-KPVIFQGNTNPTDVVFAVFPKPL 401
American_mink        TQGAISKETKKKYYVKTYRVDISSNGEDWITIKEGS-KPVIFQGNTNPTDVVFGVFPKPL 399
Ferret_X1            TQGAISKETKKKYYVKTYRVDISSNGEDWITIKEGS-KPVIFQGNTNPTDVVFGVFPKPL 399
Tiger                TQGAISKETKKKYYVKTYRIDISSNGEDWITIKEGN-KPVIFQGNTNPTDVVFGVFPKPL 656
Cat_X4               TQGAISKETKKKYYVKTYRIDISSNGEDWITIKEGN-KPVIFQGNTNPTDVVFGVFPKPL 399
Cat_X3               TQGAISKETKKKYYVKTYRIDISSNGEDWITIKEGN-KPVIFQGNTNPTDVVFGVFPKPL 399
Cat_X1               TQGAISKETKKKYYVKTYRIDISSNGEDWITIKEGN-KPVIFQGNTNPTDVVFGVFPKPL 399
Cat_X2               TQGAISKETKKKYYVKTYRIDISSNGEDWITIKEGN-KPVIFQGNTNPTDVVFGVFPKPL 399
Grey_Seal_X1         TQGAISKETKKKYYVKTYRIDISSNGEDWITIKEGN-KPVIFQGNTNPTDVVFGVFPKPL 399
Bear                 TQGAISKETKKKYYVKTYRIDISSNGEDWITIKEGN-KPVIFQGNTNPTDVVFGVFPKPL 399
                     ****:*:**:: *:: :*::::********::* .. *  :  .    .::.
```

```
Cod_X1                  LTRYLRIRPVAWETGIALRFEVYGCKISEYPCSGMLGMVSGLINDAQITASSHTDRSWSP 457
Frog                    ITRFVRLRPVTWENGISLRFELYGCKITDYPCSRMLGMVSGLISDSQITASSQVDRNWVP 459
Tiger_snake_X1          LTRFIRIKPVNWETGISLRFEIYGCKITDYPCSGMLGMVSGLITDSQITASNELDRNWLP 458
Rock¬_Dove              LTRFVRIRPVSWENGVSLRFEVYGCKITDYPCSGMLGMVSGLIPDSQITASTQVDRNWIP 388
Chicken_X1              LTRFVRIKPVSWENGVSLRFEVYGCKITDYPCSGMLGMVSGLIPDSQITASTQVDRNWIP 457
Duck                    LTRFVRIRPLTWENGVSLRFEVYGCKITDYPCSGMLGMVSGLIPDSQITASTQVDRNWIP 457
Rabbit                  ITRFVRIKPLTWETGISMRFEVYGCKITDYPCSGMLGLVSGLISDSQITASNQGDRNWMP 458
Rat                     ITRFVRIKPASWETGISMRFEVYGCKITDYPCSGMLGMVSGLISDSQITASNQGDRNWMP 459
Mouse                   ITRFVRIKPVSWETGISMRFEVYGCKITDYPCSGMLGMVSGLISDSQITASNQADRNWMP 459
Bat_Little_Brown        ---------------MDDQFYLFGSFQSDYPCSGMLGMVSGLISDSQITASNQGDRNWMP 438
Elephant                ITRFVRIKPMTWETGISMRFEVYGCKITDYPCSGMLGMVSGLISDSQITASNQADRNWMP 457
Lemur_X1                ITRFVRIKPMTWETGISMRFEVYGCKITDYPCSGMLGMVSGLISDSQITASNQADRNWMP 459
Ground_Squirrel_X1      ITRFVRIKPITWETGISMRFEIYGCKITDYPCSGMLGMVSGLISDSQIAASNQGERNWMP 459
Macaque_X1              ITRFVRIKPATWETGISMRFEVYGCKITDYPCSGMLGMVSGLISDSQITSSNQGDRNWMP 461
Gorilla_X1              ITRFVRIKPATWETGISMRFEVYGCKITDYPCSGMLGMVSGLISDSQITSSNQGDRNWMP 459
Human_X1                ITRFVRIKPATWETGISMRFEVYGCKITDYPCSGMLGMVSGLISDSQITSSNQGDRNWMP 459
Human_X6                ITRFVRIKPATWETGISMRFEVYGCKITDYPCSGMLGMVSGLISDSQITSSNQGDRNWMP 278
Camel_X1                ITRFVRIKPMTWETGISLRFEVYGCKITDYPCSGMLGMVSGLISDSQITASNQGERNWMP 459
Bat_Pipistrellus_X1     ITRFVRIKPLTWENGISMRFEVYGCKITDYPCSGMLGMVSGLISDSQITASNQGDRNWMP 459
Beluga_X1               ITRFVRIKPVTWETGISMRFEVYGCKVTDYPCSGMLGMVSGLISDSQITASNQGDRNWMP 459
Dolphin_X1              ITRFVRMKPMTWETGISMRFEVYGCKITDYPCSGMLGMVSGLISDSQITASNQGDRNWMP 459
Cow                     ITRFVRIKPVTWETGISMRFEVYGCKITDYPCSGMLGMVSGLISDSQITASNQADRNWMP 459
Goat                    ITRFVRIKPVTWETGISMRFEVYGCKITDYPCSGMLGMVSGLISDSQITASNQGDRNWMP 459
Sheep                   ITRFVRIKPVTWETGISMRFEVYGCKITDYPCSGMLGMVSGLISDSQITASNQGDRNWMP 459
Pig                     ITRFVRIKPVTWETGISMRFEVYGCKITDYPCSGMLGMVSGLISDSQITASNQGDRNWMP 459
Horse_X1                ITRFVRIKPLTWETGISLRFEVYGCKMTDYPCSGMLGMVSGLISDSQITASNQGDRNWMP 459
Horse_X2                ITRFVRIKPLTWETGISLRFEVYGCKMTDYPCSGMLGMVSGLISDSQITASNQGDRNWMP 459
Dog_X1                  ITRFVRIKPMTWETGISMRFEVYGCKITDYPCSGMLGMVSGLISDSQITASNQGERNWMP 461
Dog_X2                  ITRFVRIKPMTWETGISMRFEVYGCKITDYPCSGMLGMVSGLISDSQITASNQGERNWMP 461
American_mink           IARFVRIRPATWETGISMRFEVYGCKITDYPCSGMLGMVSGLISDSQITASNQGERNWMP 459
Ferret_X1               ITRFVRIRPATWETGISMRFEVYGCKITDYPCSGMLGMVSGLISDSQITASNQGERNWMP 459
Tiger                   ITRFVRIKPVTWETGISMRFEVYGCKITDYPCSGMLGMVSGLISDSQITASNQGDRNWMP 716
Cat_X4                  ITRFVRIKPVTWETGISMRFEVYGCKITDYPCSGMLGMVSGLISDSQITASNQGDRNWMP 459
Cat_X3                  ITRFVRIKPVTWETGISMRFEVYGCKITDYPCSGMLGMVSGLISDSQITASNQGDRNWMP 459
Cat_X1                  ITRFVRIKPVTWETGISMRFEVYGCKITDYPCSGMLGMVSGLISDSQITASNQGDRNWMP 459
Cat_X2                  ITRFVRIKPVTWETGISMRFEVYGCKITDYPCSGMLGMVSGLISDSQITASNQGDRNWMP 459
Grey_Seal_X1            ITRFVRIKPVTWETGISMRFEVYGCKITDYPCSGMLGMVSGLISDSQITASNQGERNWMP 459
Bear                    ITRFVRIKPVTWETGISMRFEVYGCKITDYPCSGMLGMVSGLISDSQITASNQGERNWMP 459
                                       :  :* ::*.  ::**** ***:***** *:**::*.. :*.* *
```

```
Cod_X1                   ENSRLLTSRSGWTLLP-QPQPFANEWLQVDLGQEKLLRGLVIQGGKHRDNKVFMKKFRLG 516
Frog                     ELARLVTSRSGWALPPSNTHPYTKEWLQIDLAEEKIVRGVIIQGGKHKENKVFMRKFKIG 519
Tiger_snake_X1           ENVRLITSRIGWALPPPVGHTYTKEWLQIDLGEEKRVQGIIVQGGKHRENKVFMRKFKIE 518
Rock_Dove                ENARLITSRSGWALPP-TTHPYTNEWLQIDLGEEKQVRGIIVQGGKHRENKVFMKKFKIG 447
Chicken_X1               ENARLITSRSGWALPP-TTHPYTNEWLQIDLGEEKIVRGIIVQGGKHRENKVFMKKFKIG 516
Duck                     ENARLITSRSGWALPP-TTHPYTNEWLQIDLGEEKKVRGIIVQGGKHRENKVFMKKFKIG 516
Rabbit                   ENVRLVTSRSGWALPP-APHPYTNEWLQVDLGEEKVVRGVIVQGGKHRENKVFMKKFKIG 517
Rat                      ENIRLVTSRTGWALPP-SPHPYINEWLQVDLGDEKIVRGVIIQGGKHRENKVFMRKFKIA 518
Mouse                    ENIRLVTSRTGWALPP-SPHPYTNEWLQVDLGDEKIVRGVIIQGGKHRENKVFMRKFKIA 518
Bat_Little_Brown         ENIRLVTSRSGWVLPP-APHPYINEWLQVDLGEEKIVRGIIIQGGKHRENKVSMRKFKIG 497
Elephant                 ENIRLVTSRSGWALPP-APHTYVNQWLQVDLGEEKIVRGVIIQGGKHRENKVFMRKFKIG 516
Lemur_X1                 ENIRLVTSRSGWALPP-TPHPYVNEWLQVDLGEERIVRGIIIQGGKHRENKVFMRKFKVG 518
Ground_Squirrel_X1       ENIRLVTSRSGWALPP-APHPYVNEWLQVDLGEEKIVRGIIIQGGKHRENKVFMRKFKIG 518
Macaque_X1               ENIRLVTSRSGWALPP-APHSYVNEWLQIDLGEEKIVRGIIIQGGKHRENKVFMRKFKIG 520
Gorilla_X1               ENIRLVTSRSGWALPP-APHSYVNEWLQIDLGEEKIVRGIIIQGGKHRENKVFMRKFKIG 518
Human_X1                 ENIRLVTSRSGWALPP-APHSYINEWLQIDLGEEKIVRGIIIQGGKHRENKVFMRKFKIG 518
Human_X6                 ENIRLVTSRSGWALPP-APHSYINEWLQIDLGEEKIVRGIIIQGGKHRENKVFMRKFKIG 337
Camel_X1                 ENIRLVTSRSGWALPP-APHPYVNEWLQVDLGEEKIVRGVIIQGGKHRENKVFMRKFKIG 518
Bat_Pipistrellus_X1      ENIRLVTSRSGWVLPP-APHPYINEWLQVDLGDEKIVRGIIIQGGKHRENKVSMRKFKIG 518
Beluga_X1                ENIRLVTSRSGWALPP-APHPYVNEWLQVDLAEEKIVQGVIIQGGKHRENKVFMRKFKIG 518
Dolphin_X1               ENIRLVTSRSGWALPP-APHPYVNEWLQVDLAEEKIVRGVIIQGGKHRENKVFMRKFKIG 518
Cow                      ENIRLVTSRSGWALPP-APHPYVNEWLQVDLAEEKIVRGVIIQGGKHRENKVFMRKFKIG 518
Goat                     ENIRLVTSRSGWALPP-APHPYVNEWLQVDLAEEKIVRGVIIQGGKHRENKVFMRKFKIG 518
Sheep                    ENIRLVTSRSGWALPP-APHPYVNEWLQVDLAEEKIVRGVIIQGGKHRENKVFMRKFKIG 518
Pig                      ENIRLVTSRSGWALPP-APHPYVNEWLQVDLGEEKIVRGIIIQGGKHRENKVYMRKFKIG 518
Horse_X1                 ENIRLVTSRSGWALPP-APHPYINEWLQVDLGEEKIVRGVIIQGGKHRENKVFMRKFKIG 518
Horse_X2                 ENIRLVTSRSGWALPP-APHPYINEWLQVDLGEEKIVRGVIIQGGKHRENKVFMRKFKIG 518
Dog_X1                   ENIRLVTSRSGWTLPP-APHPYINEWLQVDLGEEKIVRGIIIQGGKHRENKVFMRKFKIG 520
Dog_X2                   ENIRLVTSRSGWTLPP-APHPYINEWLQVDLGEEKIVRGIIIQGGKHRENKVFMRKFKIG 520
American_mink            ENIRLVTSRSGWALPP-APHPYINEWLQVDLGEEKIVRGIIIQGGKHRENKVFMRKFKIG 518
Ferret_X1                ENIRLVTSRSGWALPP-APHPYINEWLQVDLGEEKIVRGIIIQGGKHRENKVFMRKFKIG 518
Tiger                    ENIRLVTSRSGWALPP-APHPYINEWLQVDLGEEKIVRGIIIQGGKHRENKVFMRKFKIG 775
Cat_X4                   ENIRLVTSRSGWALPP-APHPYINEWLQVDLGEEKIVRGIIIQGGKHRENKVFMRKFKIG 518
Cat_X3                   ENIRLVTSRSGWALPP-APHPYINEWLQVDLGEEKIVRGIIIQGGKHRENKVFMRKFKIG 518
Cat_X1                   ENIRLVTSRSGWALPP-APHPYINEWLQVDLGEEKIVRGIIIQGGKHRENKVFMRKFKIG 518
Cat_X2                   ENIRLVTSRSGWALPP-APHPYINEWLQVDLGEEKIVRGIIIQGGKHRENKVFMRKFKIG 518
Grey_Seal_X1             ENIRLVTSRSGWALPP-APHPYINEWLQVDLGEEKIVRGIIIQGGKHRENKVFMRKFKIG 518
Bear                     ENIRLVTSRSGWALPP-APHPYINEWLQVDLGEEKIVRGIIIQGGKHRENKVFMRKFKIG 518
                         *  **:*** **.* *   : : ::***:**.:*: ::*:::*****::*** *:**::
```

```
Cod_X1                    HSNNGSHFSMLMDDSGSRPK-----IFEGNMNYDTPELRTLEPLLTRYIRVYPERAAPAG 571
Frog                      YSNNGTEWEMIMDSSKNKPK-----TFEGNTNYDTPELRTFAHITTRFIRIIPERASASG 574
Tiger_snake_X1            YSYNGSDWKWIMDASKKKAK-----IFEGNTNYDTPELRTFDPLTTRFIRVYPERATHAG 573
Rock_Dove                 YSNNGSDWKMIMDSSKKKIK-----TFEGNTNYDTPELRTFEPVVTRFIRVYPERATHGG 502
Chicken_X1                YSNNGSDWKMIMDSSKKKIK-----TFEGNTNYDTPELRTFEPVSTRFIRVYPERATHAG 571
Duck                      YSNNGSDWKMIMDSSKKKIK-----TFEGNTNYDTPELRTFEPVSTRFIRVYPERATHGG 571
Rabbit                    HSHNGSDWRMITDDSKRKAK-----SFEGNSNYDTPELRTFPPVSTRFIRIYPERATHGG 572
Rat                       YSNNGSDWKMIMDDSKRKAKVRPWVFFEGNNNFDTPELRAFTPLSTRFIRIYPERATHSG 578
Mouse                     YSNNGSDWKTIMDDSKRKAK-----SFEGNNNYDTPELRTFSPLSTRFIRIYPERATHSG 573
Bat_Little_Brown          YSNNGSDWKMIMDDSKRKAK-----SFEGNSNYDTPELRTFQPLSTRFIRIYPERATHGG 552
Elephant                  YSNNGSDWKMIMDDSKRKAK-----SFEGNNNYDTPELRTFPPLSTRFIRIYPERATHGG 571
Lemur_X1                  YSNNGSDWKMIMDDSKRKAK-----SFEGNNNYDTPELRTFPALSTRFIRIYPERATHGG 573
Ground_Squirrel_X1        YSNNGSDWKMIMDDSKRKPK-----SFEGNNNYDTPELRTFPPLSTRFIRIYPERATHGG 573
Macaque_X1                YSNNGSDWKMIMDDSKRKAK-----SFEGNNNYDTPELRTFPALSTRFIRIYPERATHGG 575
Gorilla_X1                YSNNGSDWKMIMDDSKRKAK-----SFEGNNNYDTPELRTFPALSTRFIRIYPERATHGG 573
Human_X1                  YSNNGSDWKMIMDDSKRKAK-----SFEGNNNYDTPELRTFPALSTRFIRIYPERATHGG 573
Human_X6                  YSNNGSDWKMIMDDSKRKAK-----SFEGNNNYDTPELRTFPALSTRFIRIYPERATHGG 392
Camel_X1                  YSNNGSDWKMLMDEGRHKPK-----SFEGNNNYDTPELRTFPPLSTRFVRIYPERATHGG 573
Bat_Pipistrellus_X1       YSNNGSDWKMIMDDSKRKAK-----SFEGNSNYDTPELRTFQPLSTRFIRIYPERATHGG 573
Beluga_X1                 YSNNGSDWKMVMDDSKRKAK-----SFEGNNNYDTPELRTFPPLSTRFIRIYPERATHGG 573
Dolphin_X1                YSNNGSDWKMVMDDSKRKAK-----SFEGNNNYDTPELRTFPPLSTRFIRIYPERATHGG 573
Cow                       YSNNGSDWKMIMDDSKRKAK-----SFEGNNNYDTPELRTFPPLSTRFIRIYPERATHGG 573
Goat                      YSNNGSDWKMIMDDSKRKAK-----SFEGNNNYDTPELRTFPPLSTRFIRIYPERATHGG 573
Sheep                     YSNNGSDWKMIMDDSKRKAK-----SFEGNNNYDTPELRTFPPLSTRFIRIYPERATHGG 573
Pig                       YSNNGSDWKMIMDDGKRKAK-----SFEGNNNYDTPELRTFPPLSTRFIRIYPERATHGG 573
Horse_X1                  YSNNGSDWKMIMDDSKRKAK-----SFEGNNNYDTPELRTFPPLSTRFIRIYPERATHGG 573
Horse_X2                  YSNNGSDWKMIMDDSKRKAK-----SFEGNNNYDTPELRTFPPLSTRFIRIYPERATHGG 573
Dog_X1                    YSNNGSDWRMIMDDSKRKAK-----SFEGNNNYDTPELRTFPPLSTRFIRIYPERATHGG 575
Dog_X2                    YSNNGSDWRMIMDDSKRKAK-----SFEGNNNYDTPELRTFPPLSTRFIRIYPERATHGG 575
American_mink             YSNNGSDWKMIMDDSKRKAK-----SFEGNNNYDTPELRTFPPLSTRFIRIYPERATHGG 573
Ferret_X1                 YSNNGSDWKMIMDDSKRKAK-----SFEGNNNYDTPELRTFPPLSTRFIRIYPERATHGG 573
Tiger                     YSNNGSDWKMIMDDSKRKAK-----SFEGNNNYDTPELRTFPPLSTRFIRIYPERATHGG 830
Cat_X4                    YSNNGSDWKMIMDDSKRKAK-----SFEGNNNYDTPELRTFPPLSTRFIRIYPERATHGG 573
Cat_X3                    YSNNGSDWKMIMDDSKRKAK-----SFEGNNNYDTPELRTFPPLSTRFIRIYPERATHGG 573
Cat_X1                    YSNNGSDWKMIMDDSKRKAK-----SFEGNNNYDTPELRTFPPLSTRFIRIYPERATHGG 573
Cat_X2                    YSNNGSDWKMIMDDSKRKAK-----SFEGNNNYDTPELRTFPPLSTRFIRIYPERATHGG 573
Grey_Seal_X1              YSNNGSDWKMIMDDSKRKAK-----SFEGNNNYDTPELRTFPPLSTRFVRIYPERATHGG 573
Bear                      YSNNGSDWKMIMDDSKRKAK-----SFEGNNNYDTPELRTFPPLSTRFIRIYPERATHGG 573
                          :* **:.:  : * .  : *      **** *:******::  : **::*: ****: .*
```

```
Cod_X1                  MGLRLEILGCEIEAPTIPPTTQPPSAPPADECEDGRASCHSSTGDDYDPTDGTTTPETTT 631
Frog                    LALRLELLGCEVETPTSIPTT--PEVNGGDECEGDLANCHSGTDEGFKLTVGATGQSTET 632
Tiger_snake_X1          LGLRLELLGCEIEVTTSVPTT--ADTSPVDECDDDQANCYSGTGDDFHLTGSTTATITEK 631
Rock_Dove               LGLRMELLGCELEAPTAVPTV--SEGKPMDECDDDQANCHSGTGDDYQLTGGTTVLNTEK 560
Chicken_X1              LGLRMELLGCELEAPTAVPTV--SEGKPVDECDDDQANCHSGTGDDYQLTGGTTVLNTEK 629
Duck                    LGLRMELLGCELEAPTAVPTV--SEGKPVDECDDDQANCHSGTGDDYQLTGGTTVLNTEK 629
Rabbit                  LGLRMELLGCELEVPTAGPTT--PNGNPMDECDDDQATCHSGTGDDLQLTGGTTVLTTEK 630
Rat                     LGLRMELLGCEVEAPTAGPTT--PNGNPVDECDDDQANCHSGTGDDFQLTGGTTVLATEK 636
Mouse                   LGLRMELLGCEVEAPTAGPTT--PNGNPVDECDDDQANCHSGTGDDFQLTGGTTVLATEK 631
Bat_Little_Brown        LGLRMELLGCEVEAPTAGPTT--PNGNLVDECDDDQANCHSGTGDDFQLTGGTTVLTTEK 610
Elephant                LGLRMELLGCEVEVPTAGPTT--PNGNPIDECDDDQANCHSGTGDDFQLTGGTTVLTTEK 629
Lemur_X1                LGLRMELLGCEVEAPTAGPTT--PNGNPVDECDDDQANCHSGTGDDFQLTGGTTVLSTEK 631
Ground_Squirrel_X1      LGLRMELLGCEVEAPTAGPTT--PNGNPVDECDDDQANCHSGTGDDFQLTGGTTVLATEK 631
Macaque_X1              LGLRMELLGCEVEAPTAGPTT--PNGNPVDECDDDQANCHSGTGDDFQLTGGTTVLATEK 633
Gorilla_X1              LGLRMELLGCEVEAPTAGPTT--PNGNLVDECDDDQANCHSGTGDDFQLTGGTTVLATEK 631
Human_X1                LGLRMELLGCEVEAPTAGPTT--PNGNLVDECDDDQANCHSGTGDDFQLTGGTTVLATEK 631
Human_X6                LGLRMELLGCEVEAPTAGPTT--PNGNLVDECDDDQANCHSGTGDDFQLTGGTTVLATEK 450
Camel_X1                LGLRMELLGCELEAPTAGPTT--PNGNLVDECDDDQTSCHSGTGDDFQLTGGTTVLTTEK 631
Bat_Pipistrellus_X1     LGLRMELLGCEVEAPTAGPTT--PNANLVDECDDDQANCHSGTGDDFQLTGGTTVLTTEK 631
Beluga_X1               LGLRMELLGCEVEAPTAGPTT--PNGNLVDECDDDQANCHSGTGDDFQLTGGTTVLTTEK 631
Dolphin_X1              LGLRMELLGCEVEAPTAGPTT--PNGNLVDECDDDQANCHSGTGDDFQLTGGTTVLTTEK 631
Cow                     LGLRMELLGCEVEAPTAGPTT--PNGNLVDECDDDQANCHSGTGDDFQLTGGTTVLTTEK 631
Goat                    LGLRMELLGCEVEAPTAGPTT--PNGNLVDECDDDQANCHSGTGDDFQLTGGTTVLTTEK 631
Sheep                   LGLRMELLGCEVEAPTAGPTT--PNGNLVDECDDDQANCHSGTGDDFQLTGGTTVLTTEK 631
Pig                     LGLRMELLGCEVEAPTAGPTT--PNGNLVDECDDDQASCHSGTGDDFQLTGGTTVLTTEK 631
Horse_X1                LGLRMELLGCEVEAPTAGPTT--PNGNLVDECDDDQANCHSGTGDDFQLTGGTTVLTTEK 631
Horse_X2                LGLRMELLGCEVEAPTAGPTT--PNGNLVDECDDDQANCHSGTGDDFQLTGGTTVLTTEK 631
Dog_X1                  LGLRMELLGCEVEAPTAGPTT--PNGNLVDECDDDQANCHSGTGDDFQLTGGTTVLTTER 633
Dog_X2                  LGLRMELLGCEVEAPTAGPTT--PNGNLVDECDDDQANCHSGTGDDFQLTGGTTVLTTER 633
American_mink           LGLRMELLGCELEAPTAGPTT--PNGNLVDECDDDQANCHSGT----------------- 614
Ferret_X1               LGLRMELLGCELEAPTAGPTT--PNGNLVDECDDDQANCHSGTGDDFQLTGGTTVLTTEK 631
Tiger                   LGLRMELLGCEVEAPTAGPTT--PNGNLVDECDDDQANCHSGTGDDFQLTGGTTVLTTEK 888
Cat_X4                  LGLRMELLGCEVEAPTAGPTT--PNGNLVDECDDDQANCHSGT-------GGTTVLTTEK 624
Cat_X3                  LGLRMELLGCEVEAPTAGPTT--PNGNLVDECDDDQANCHSGT-------GGTTVLTTEK 624
Cat_X1                  LGLRMELLGCEVEAPTAGPTT--PNGNLVDECDDDQANCHSGTGDDFQLTGGTTVLTTEK 631
Cat_X2                  LGLRMELLGCEVEAPTAGPTT--PNGNLVDECDDDQANCHSGTGDDFQLTGGTTVLTTEK 631
Grey_Seal_X1            LGLRMELLGCEVEAPTAGPTT--PNGNLVDECDDDQANCHSGTGDDFQLTGGTTVLATEK 631
Bear                    LGLRMELLGCEVEAPTAGPTT--PNGNLVDECDDDQANCHSGTGDDFQLTGGTTVLTTEK 631
                        :.**:*:****:*. *  **.   .    ***:.. :.*:*.*
```

```
Cod_X1                  AELD-------------TVPAFLWFACDFGWASDPSFCSWTSED-TGSRWQIQSSGTPTL 677
Frog                    PTVEASPE--------EPDMTHSDLDCKFGWGSQKTVCNWQHDISSGLKWAVLN-----S 679
Tiger_snake_X1          PVPTDTTVQPAIHYSITEELPAYGFNCGFGYSSQKTICRWEHDAQLDLRWAVLT-----S 686
Rock_Dove               PTVIDNTL--------QPELPLYNFNCGFGWGSQKTLCHWEHDNQVDLKWAILT-----S 607
Chicken_X1              PTVIDNTV--------QPELPPYNLNCGFGWGSHKTLCQWEHDNQVDLKWAILT-----S 676
Duck                    PTVIDNTL--------QPELPIYNFNCGFGWGSHKTLCHWEHDNQVDLKWAILT-----S 676
Rabbit                  PTVIDSTM--------QSEFPTYGFNCEFGWGSHKTFCRWEHDSHVQLRWSVLT-----S 677
Rat                     PTIIDSTI--------QSEFPTYGFNCEFGWGSHKTFCHWEHDSHAQLRWSVLT-----S 683
Mouse                   PTIIDSTI--------QSEFPTYGFNCEFGWGSHKTFCHWEHDSHAQLRWSVLT-----S 678
Bat_Little_Brown        PTIIDSTI--------QSEFSTYGFNCEFGWGSHKTFCHWEHDNHVQLKWSVLT-----S 657
Elephant                PTVLDSTI--------QSEFPTYGFNCEFGWGSHKTFCHWEHDNHVQLKWSVLT-----S 676
Lemur_X1                PTVLDSTV--------QSEFPTYGFNCEFGWGSHKTFCHWEHDNHVQLKWSVLT-----S 678
Ground_Squirrel_X1      PTVLDSTI--------QSEFPTYGFNCEFGWGSHKTFCHWEHDNHVQLKWSVLT-----S 678
Macaque_X1              PTVIDSTI--------QSEFPTYGFNCEFGWGSHKTFCHWEHDNHVQLKWSVLT-----S 680
Gorilla_X1              PTVIDSTI--------QSEFPTYGFNCEFGWGSHKTFCHWEHDNHVQLKWSVLT-----S 678
Human_X1                PTVIDSTI--------QSEFPTYGFNCEFGWGSHKTFCHWEHDNHVQLKWSVLT-----S 678
Human_X6                PTVIDSTI--------QSEFPTYGFNCEFGWGSHKTFCHWEHDNHVQLKWSVLT-----S 497
Camel_X1                PTAIDSTP--------QPEFPTYGFNCEFGWGSHKTFCHWEHDNHVQLKWSVLT-----S 678
Bat_Pipistrellus_X1     PTILDSTI--------QSEFPTYGFNCEFGWGSHKTFCHWEHDNHVQLKWSVLT-----S 678
Beluga_X1               PTVIDSTI--------QSEFPTYGFNCEFGWGSHKTFCHWEHDSHVQLRWSVLT-----S 678
Dolphin_X1              PTVIDSTI--------QSEFPTYGFNCEFGWGSHKTFCHWEHDNHVQLRWSVLT-----S 678
Cow                     PTVIDSTI--------QSEFPTYGFNCEFGWGSHKTFCHWEHDNHVQLKWSVLT-----S 678
Goat                    PTVIDSTI--------QSEFPTYGFNCEFGWGSHKTFCHWEHDNHVQLKWSVLT-----S 678
Sheep                   PTVIDSTI--------QSEFPTYGFNCEFGWGSHKTFCHWEHDNHVQLKWSVLT-----S 678
Pig                     PTIIDSTI--------QSEFPTYGFNCEFGWGSHKTFCHWEHDSHVQLKWSVLT-----S 678
Horse_X1                PTVLDSTI--------QSEFPTYGFNCEFGWGSHKTFCHWEHDNHVQLKWSVLT-----S 678
Horse_X2                PTVLDSTI--------QSEFPTYGFNCEFGWGSHKTFCHWEHDNHVQLKWSVLT-----S 678
Dog_X1                  PTVIDSTI--------QSEFPTYGFNCEFGWGSHKTFCRWEHDNQVQLKWSVLT-----S 680
Dog_X2                  PTVIDSTI--------QSEFPTYGFNCEFGWGSHKTFCRWEHDNQVQLKWSVLT-----S 680
American_mink           ------------------------------------------------------------ 614
Ferret_X1               PTVIDSTI--------QSEFPTYGFNCEFGWGSHKTFCHWEHDNRVQLKWSVLT-----S 678
Tiger                   PTIIDSTI--------QSEFPTYGFNCEFGWGSHKTFCHWEHDNHVQLKWSVLT-----S 935
Cat_X4                  PTIIDSTI--------QSEFPTYGFNCEFGWGSHKTFCHWEHDNHVQLKWSVLT-----S 671
Cat_X3                  PTIIDSTI--------QSEFPTYGFNCEFGWGSHKTFCHWEHDNHVQLKWSVLT-----S 671
Cat_X1                  PTIIDSTI--------QSEFPTYGFNCEFGWGSHKTFCHWEHDNHVQLKWSVLT-----S 678
Cat_X2                  PTIIDSTI--------QSEFPTYGFNCEFGWGSHKTFCHWEHDNHVQLKWSVLT-----S 678
Grey_Seal_X1            PTIIDSTI--------QSEFPTYGFNCEFGWGSHKTFCHWEHDNHVQLKWSVLT-----S 678
Bear                    PTIIDSTI--------QSEFPTYGFNCEFGWGSHKTFCHWEHDNHVQLKWSVLT-----S 678
```

```
Cod_X1                   NTGPNMDHTGGSGNFIYTLATGHQETAVARLVSPMVSSPDADLCVSFWYHMFGPRIGMLH 737
Frog                     KTGPVQDHT-GDGNFIYSEADERHEGRAARLMSPVVSSSRSAHCLTFWYHMDGSHVGTLS 738
Tiger_snake_X1           KTGPIQDHT-GDGNFIYSQVDENQKSKIVRLISPIINLQNYALCMTFWYHMSGPHVGTLR 745
Rock_Dove                KTGPIQDHTVGDGNFIYSQADESQKGKVARLLSPVIYSQNSAHCMTFWYHMSGPHVGTLK 667
Chicken_X1               KTGPIQDHT-GDGNFIYSQADESQKGKVARLLSPIIYSQNSAHCMTFWYHMSGPHVGTLK 735
Duck                     KTGPIQDHTAGDGNFIYSQADESQKGKVARLLSPVIYSQNSAHCMTFWYHMSGPHVGTLK 736
Rabbit                   KTGPIQDHT-GDGNFIYSQADENQKGKVARLVSPVVSSQSSAHCMTFWYHMSGSHVGTLR 736
Rat                      KTGPIQDHT-GDGNFIYSQADENQKGKVARLVSPVVYSQSSAHCMTFWYHMSGSHVGTLR 742
Mouse                    KTGPIQDHT-GDGNFIYSQADENQKGKVARLVSPVVYSQSSAHCMTFWYHMSGSHVGTLR 737
Bat_Little_Brown         KTGPIQDHT-GDGNFIYSQADENQKGKVARLVSPVVYSQNSAHCMTFWYHMSGSHVGTLR 716
Elephant                 KTGPIQDHTAGDGNFIYSQADENQKGKVARLVSPVVYSQNSAHCLTFWYHMSGSHVGTLR 736
Lemur_X1                 KTGPIQDHTAGDGNFIYSQADENQKGKVARLVSPVVYSQNSAHCMTFWYHMSGSHVGTLR 738
Ground_Squirrel_X1       KTGPIQDHT-GDGNFIYSQADENQKGKVARLVSPVVYSQNSAHCMTFWYHMSGSHVGTLR 737
Macaque_X1               KTGPIQDHT-GDGNFIYSQADENQKGKVARLVSPVVYSQNSAHCMTFWYHMSGSHVGTLR 739
Gorilla_X1               KTGPIQDHT-GDGNFIYSQADENQKGKVARLVSPVVYSQNSAHCMTFWYHMSGSHVGTLR 737
Human_X1                 KTGPIQDHT-GDGNFIYSQADENQKGKVARLVSPVVYSQNSAHCMTFWYHMSGSHVGTLR 737
Human_X6                 KTGPIQDHT-GDGNFIYSQADENQKGKVARLVSPVVYSQNSAHCMTFWYHMSGSHVGTLR 556
Camel_X1                 KTGPIQDHTAGDGNFLYSQADENQKGKVARLVSPVVYSQNSAHCMTFWYHMSGSHVGTLR 738
Bat_Pipistrellus_X1      KTGPIQDHTAGDGNFIYSQADENQKGKVARLVSPVVYSQNSAHCLTFWYHMSGFQVGTLR 738
Beluga_X1                KTGPIQDHTAGDGNFIYSQADENQKGKVARLVSPVVYSQNSAHCMTFWYHMSGSHVGTLR 738
Dolphin_X1               KTGPIQDHTAGDGNFIYSQADENQKGKVARLVSPVVYSQNSAHCMTFWYHMSGSHVGTLR 738
Cow                      KTGPIQDHTAGDGNFIYSQADENQKGKVARLVSPVVYSQNAAHCMTFWYHMSGSHVGTLR 738
Goat                     KTGPIQDHTAGDGNFIYSQADENQKGKVARLVSPVVYSQNSAHCMTFWYHMSGSHVGTLR 738
Sheep                    KTGPIQDHTAGDGNFIYSQADENQKGKVARLVSPVVYSQNSAHCMTFWYHMSGSHVGTLR 738
Pig                      KTGPIQDHTAGDGNFIYSQADENQKGKVARLVSPVVYSQNSAHCMTFWYHMSGSHVGTLR 738
Horse_X1                 KTGPIQDHTAGDGNFIYSQADENQKGKVARLVSPVVYSQNSAHCMTFWYHMSGTHVGTLR 738
Horse_X2                 KTGPIQDHT-GDGNFIYSQADENQKGKVARLVSPVVYSQNSAHCMTFWYHMSGTHVGTLR 737
Dog_X1                   KTGPIQDHTAGDGNFIYSQADENQKGKVARLVSPVVYSQNSAHCMTFWYHMSGSHVGTLR 740
Dog_X2                   KTGPIQDHT-GDGNFIYSQADENQKGKVARLVSPVVYSQNSAHCMTFWYHMSGSHVGTLR 739
American_mink            ------------------------------------------------------------ 614
Ferret_X1                KTGPIQDHTAGDGNFIYSQADENQKGKVARLVSPVVYSQNSAHCMTFWYHMSGSHVGTLR 738
Tiger                    KTGPIQDHTAGDGNFIYSQADENQKGKVARLVSPVVYSQNSAHCMTFWYHMSGSHVGTLR 995
Cat_X4                   KTGPIQDHT-GDGNFIYSQADENQKGKVARLVSPVVYSQNSAHCMTFWYHMSGSHVGTLR 730
Cat_X3                   KTGPIQDHTAGDGNFIYSQADENQKGKVARLVSPVVYSQNSAHCMTFWYHMSGSHVGTLR 731
Cat_X1                   KTGPIQDHTAGDGNFIYSQADENQKGKVARLVSPVVYSQNSAHCMTFWYHMSGSHVGTLR 738
Cat_X2                   KTGPIQDHT-GDGNFIYSQADENQKGKVARLVSPVVYSQNSAHCMTFWYHMSGSHVGTLR 737
Grey_Seal_X1             KTGPIQDHTAGDGNFIYSQADENQKGKVARLVSPVVYSQNSAHCMTFWYHMSGSHVGTLR 738
Bear                     KTGPIQDHTAGDGNFIYSQADENQKGKVARLVSPVVYSQNSAHCMTFWYHMSGAHVGTLR 738
```

```
Cod_X1                 LKQRKQTPEGPADVVLWTVSGHQGNRWREGRILIPHSNKPYQVVIEGLVERKSWGDIAVD 797
Frog                   IKLKYEMEED-FDQTLWTVSGNQGDQWKEARVVLHKTMKQYQVIVEGTVGKGSAGGIAVD 797
Tiger_snake_X1         IKLRYQVPKE-YDRVLWTLSGNQGNCWKEGRVFLHKSVKHYQVMVEGEIGKGN-GGIAVD 803
Rock_Dove              IKLRYQKPDE-YDQVLWTLSGNQANSWKEGRVLLHKSVKHYQVVIEGEIGKGT-GGIAVD 725
Chicken_X1             IKLRYQKPDE-YDQVLLSLNGHQANCWKEGRVLLHKSVKLYQVVIEGEIGKGN-GGIAVD 793
Duck                   IKLRYQKPDE-YDQVLWTLSGHQANCWKEGRVLLHKSVKHYQVVIEGEIGKGN-GGIAVD 794
Rabbit                 VKLRYQKPEE-YDQLVWMAIGHQGDHWKEGRVLLHKSLKLYQVIFEGEIGKGNLGGIAVD 795
Rat                    VKLHYQKPEE-YDQLVWMVVGHQGDHWKEGRVLLHKSLKLYQVIFEGEIGKGNLGGIAVD 801
Mouse                  VKLRYQKPEE-YDQLVWMVVGHQGDHWKEGRVLLHKSLKLYQVIFEGEIGKGNLGGIAVD 796
Bat_Little_Brown       VKLRYQKPEE-YDQLVWRAIGHQGDHWQEGRVLLRKSLKLYQVIFEGEIGKGNLGGIAVD 775
Elephant               VKLRYQKPEE-YDQLVWMAIGHQGDHWKEGRVLLHKSLKLYQVIFEGEIGKGNLGGIAVD 795
Lemur_X1               VKLRYQKPEE-YDQLVWMAIGHQGDHWKEGRVLLHKSLKLYQVIFEGEIGKGNLGGIAVD 797
Ground_Squirrel_X1     VKLRYQKPEE-YDQLVWMAIGHQGDHWKEGRVLLHKSLKLYQVIFEGEIGKGNLGGIAVD 796
Macaque_X1             VKLRYQKPEE-YDQLVWMAIGHQGDHWKEGRVLLHKSLKLYQVIFEGEIGKGNLGGIAVD 798
Gorilla_X1             VKLRYQKPEE-YDQLVWMAIGHQGDHWKEGRVLLHKSLKLYQVIFEGEIGKGNLGGIAVD 796
Human_X1               VKLRYQKPEE-YDQLVWMAIGHQGDHWKEGRVLLHKSLKLYQVIFEGEIGKGNLGGIAVD 796
Human_X6               VKLRYQKPEE-YDQLVWMAIGHQGDHWKEGRVLLHKSLKLYQVIFEGEIGKGNLGGIAVD 615
Camel_X1               VKLRYQKPEE-YDQLVWMAIGHQGDHWKEGRVLLHKSLKLYQVIFEGEIGKGSLGGIAVD 797
Bat_Pipistrellus_X1    VKLRYQKPEE-YDQLVWRAIGHQGDHWKEGRVLLHKSLKLYQVIFEGEIGKGNLGGIAVD 797
Beluga_X1              VKLRYQKPEE-YDQLVWMAIGHQGDHWKEGRVLLHKSLKLYQVIFEGEIGKGNLGGIAVD 797
Dolphin_X1             VKLRYQKPEE-YDQLVWMAIGHQGDHWKEGRVLLHKSLKLYQVIFEGEIGKGNLGGIAVD 797
Cow                    VKLRYQKPEE-YDQLVWMAIGHQGDHWKEGRVLLHKSLKLYQVIFEGEIGKGNLGGIAVD 797
Goat                   VKLRYQKPEE-YDQLVWMAIGHQGDHWKEGRVLLHKSLKLYQVIFEGEIGKGNLGGIAVD 797
Sheep                  VKLRYQKPEE-YDQLVWMAIGHQGDHWKEGRVLLHKSLKLYQVIFEGEIGKGNLGGIAVD 797
Pig                    VKLRYQKPEE-YDQLVWMAIGHQGDHWKEGRVLLHKSLKLYQVIFEGEIGKGSLGGIAVD 797
Horse_X1               VKLHYQKPED-YDQLVWMAIGHQGDHWKEGRVWLHKSLKLYQVIFEGEIGKGNLGGIAVD 797
Horse_X2               VKLHYQKPED-YDQLVWMAIGHQGDHWKEGRVWLHKSLKLYQVIFEGEIGKGNLGGIAVD 796
Dog_X1                 VKLHYQKPEE-YDQLVWMAIGHQGDHWKEGRVLLHKSLKLYQVIFEGEIGKGNLGGIAVD 799
Dog_X2                 VKLHYQKPEE-YDQLVWMAIGHQGDHWKEGRVLLHKSLKLYQVIFEGEIGKGNLGGIAVD 798
American_mink          ------------------------------------------------------------ 614
Ferret_X1              VKLHYQKPEE-YDQLVWMAIGHQGDHWKEGRVLLHKSLKLYQVIFEGEIGKGNLGGIAVD 797
Tiger                  VKLHYQKPEE-YDQLVWMAIGHQGDHWKEGRVLLHKSLKLYQVIFEGEIGKGNLGGIAVD 1054
Cat_X4                 VKLHYQKPEE-YDQLVWMAIGHQGDHWKEGRVLLHKSLKLYQVIFEGEIGKGNLGGIAVD 789
Cat_X3                 VKLHYQKPEE-YDQLVWMAIGHQGDHWKEGRVLLHKSLKLYQVIFEGEIGKGNLGGIAVD 790
Cat_X1                 VKLHYQKPEE-YDQLVWMAIGHQGDHWKEGRVLLHKSLKLYQVIFEGEIGKGNLGGIAVD 797
Cat_X2                 VKLHYQKPEE-YDQLVWMAIGHQGDHWKEGRVLLHKSLKLYQVIFEGEIGKGNLGGIAVD 796
Grey_Seal_X1           VKLHYQKPEE-YDQLVWMAIGHQGDHWKEGRVLLHKSLKLYQVIFEGEIGKGNLGGIAVD 797
Bear                   VKLHYQKPEE-YDQLVWMAIGHQGDHWKEGRVLLHKSLKLYQVIFEGEIGKGNLGGIAVD 797
```

```
Cod_X1                  DIKILDGISMAECHDPFVPTEPMLPEDRLNK----------------------------- 828
Frog                    DIIIANHISPSQCRAPEIDDSAN-KIGE-------------------------------- 824
Tiger_snake_X1          DINFDSHITQEECRKFTP------------------------------------------ 821
Rock_Dove               DIKIDNHVAQEDCRKSTDVENEI-DEDDDP------------------------------ 754
Chicken_X1              DINIDNHISQEDCQKSTDVESEI-VE-EDP------------------------------ 821
Duck                    DINIDNHVAQEDCRKSTDVENEI-DE-EDP------------------------------ 822
Rabbit                  DISINNHISPEDCAKPADLDRKN-PEIKIDE----------------------------- 825
Rat                     DISINNHIPQEDCAKPTDLDKKN-TEIKIDE----------------------------- 831
Mouse                   DISINNHISQEDCAKPTDLDKKN-TEIKIDE----------------------------- 826
Bat_Little_Brown        DISINNHISQEDCARPADLDTKN-PENKIDE----------------------------- 805
Elephant                DISINNHISQEDCAKPADLDKKH-PENKIDEAELQRQLPNTLTSHASVPNSESDRDLGGH 854
Lemur_X1                DISINNHISQEDCAKPADLDKKN-PEIKIDE----------------------------- 827
Ground_Squirrel_X1      DISINNHISQEDCAKPADPDKKN-TEIKIDE----------------------------- 826
Macaque_X1              DISINNHISQEDCAKPADLDKKN-PEIKIDE----------------------------- 828
Gorilla_X1              DISINNHISQEDCAKPADLDKKN-PEIKIDE----------------------------- 826
Human_X1                DISINNHISQEDCAKPADLDKKN-PEIKIDE----------------------------- 826
Human_X6                DISINNHISQEDCAKPADLDKKN-PEIKIDE----------------------------- 645
Camel_X1                DIRINNHIPQEDCTKPEDLDKKN-PENKMDE----------------------------- 827
Bat_Pipistrellus_X1     DISINNHISQEDCARQADLDKKN-PENKIDE----------------------------- 827
Beluga_X1               DISINNHIPQEDCTKPADLDKKN-PESKIDE----------------------------- 827
Dolphin_X1              DISINNHIPQEDCTKPADLDKKN-PESKIDE----------------------------- 827
Cow                     DISINNHIPQEDCAKPADLDKKN-PESKIDE----------------------------- 827
Goat                    DISINNHIPQEDCTKPADLDKKN-PESKIDE----------------------------- 827
Sheep                   DISINNHIPQEDCAKPADLDKKN-PESKIDE----------------------------- 827
Pig                     DISISNHIPQEDCAKPGDLDKKN-PESKMDE----------------------------- 827
Horse_X1                DISINNHISQEDCAKPADLDKKN-PESKIDE----------------------------- 827
Horse_X2                DISINNHISQEDCAKPADLDKKN-PESKIDE----------------------------- 826
Dog_X1                  DISINNHISQEDCAKPSDLDKRN-PENKIDE----------------------------- 829
Dog_X2                  DISINNHISQEDCAKPSDLDKRN-PENKIDE----------------------------- 828
American_mink           ------------------------------------------------------------ 614
Ferret_X1               DISINNHISQEDCAKPADLDKRN-PENKIDE----------------------------- 827
Tiger                   DISINNHISQEDCAKPADLDKRN-PENKIDE----------------------------- 1084
Cat_X4                  DISINNHISQEDCAKPADLDKRN-PENKIDE----------------------------- 819
Cat_X3                  DISINNHISQEDCAKPADLDKRN-PENKIDE----------------------------- 820
Cat_X1                  DISINNHISQEDCAKPADLDKRN-PENKIDE----------------------------- 827
Cat_X2                  DISINNHISQEDCAKPADLDKRN-PENKIDE----------------------------- 826
Grey_Seal_X1            DISINNHISQEDCAKPADLDKRN-PENKIDE----------------------------- 827
Bear                    DISINNHISQEDCAKPADLDKRN-PENKIDE----------------------------- 827
```

```
Cod_X1                   -----------------I---IEETTDYP---DLVVSNEISGPGNMLKTLDPILITIIAM 865
Frog                     --------------EDSEIDKTGSTPNYA--LNEFNESISKKPGNVLKTLDPILITIIAM 868
Tiger_snake_X1           --------------------------DYRENGEPHNDNISRKPGNVLKTLDPILITIIAM 855
Rock_Dove                -----------------ESNQTGFTPPYH-TGEDYDDNISRKPGNVLKTLDPILITIIAM 796
Chicken_X1               -----------------ESNQTGFTPSYR-TDEDYDD-ISRKPGNVLKTLDPILITIIAM 862
Duck                     -----------------ESNQTGFTPRYH-TDEDYDD-ISRKPGNVLKTLDPILITIIAM 863
Rabbit                   ---------------------TGSTPGYE-GEGEGDRNISRKPGSVLKTLDPILITIIAM 863
Rat                      ---------------------TGSTPGYE--EGKGDKNISRKPGNVLKTLDPILITIIAM 868
Mouse                    ---------------------TGSTPGYE-GEGEGDKNISRKPGNVLKTLDPILITIIAM 864
Bat_Little_Brown         ---------------------TGSTPGYE-GLGEGDENISKKPGNVLKTLDPILITIIAM 843
Elephant                 RRWGSPAPLHSASAGSWETTGKGSTPGYK-GTEDGEENISRKPGNVLKTLDPILITIIAM 913
Lemur_X1                 ---------------------TGSTPGYE-GEGEGDKNISRKPGNVLKTLDPILITIIAM 865
Ground_Squirrel_X1       ---------------------TGSTPGYE-GEGESDKNISRKPGNVLKTLDPILITIIAM 864
Macaque_X1               ---------------------TGSTPGYE-GEGEGDKNISRKPGNVLKTLDPILITIIAM 866
Gorilla_X1               ---------------------TGSTPGYE-GEGEGDKNISRKPGNVLKTLDPILITIIAM 864
Human_X1                 ---------------------TGSTPGYE-GEGEGDKNISRKPGNVLKTLDPILITIIAM 864
Human_X6                 ---------------------TGSTPGYE-GEGEGDKNISRKPGNVLKTLDPILITIIAM 683
Camel_X1                 ---------------------TGSTQGYE-GVGEGEEKISRKPGSVLKTLDPILITIIAM 865
Bat_Pipistrellus_X1      ---------------------TGSTPGYE-SLGEGDENISKKPGNVLKTLDPILITIIAM 865
Beluga_X1                ---------------------TGSTPGYQ-GTGDGDENISRKPGNVLRTLDPILITIIAM 865
Dolphin_X1               ---------------------TGSTPGYQ-GTGDGDENISRKPGNVLKTLDPILITIIAM 865
Cow                      ---------------------TGSTPGYQ-GAGEGDENISRKPGNVLKTLDPILITIIAM 865
Goat                     ---------------------TGSTPGYQ-GAGEGDENISRKPGNVLKTLDPILITIIAM 865
Sheep                    ---------------------TGSTPGYQ-GAGEGDENISRKPGNVLKTLDPILITIIAM 865
Pig                      ---------------------TGSTPGYE-GRGEGDENISRKPGSVLKTLDPILITIIAM 865
Horse_X1                 ---------------------TGSTPGYE-GTGGGDENISRKPGNVLKTLDPILITIIAM 865
Horse_X2                 ---------------------TGSTPGYE-GTGGGDENISRKPGNVLKTLDPILITIIAM 864
Dog_X1                   ---------------------TGSTPGYE-GTGEGDENISRKPGNVLKTLDPILITIIAM 867
Dog_X2                   ---------------------TGSTPGYE-GTGEGDENISRKPGNVLKTLDPILITIIAM 866
American_mink            ------------------------------------------------------------ 614
Ferret_X1                ---------------------TGSTPSYK-GPGEGDENISRKPGNVLKTLDPILITIIAM 865
Tiger                    ---------------------TGSTPGYE-GTGESNENISRKPGNVLKTLDPILITIIAM 1122
Cat_X4                   ---------------------TGSTPGYE-GTGESNENISRKPGNVLKTLDPILITIIAM 857
Cat_X3                   ---------------------TGSTPGYE-GTGESNENISRKPGNVLKTLDPILITIIAM 858
Cat_X1                   ---------------------TGSTPGYE-GTGESNENISRKPGNVLKTLDPILITIIAM 865
Cat_X2                   ---------------------TGSTPGYE-GTGESNENISRKPGNVLKTLDPILITIIAM 864
Grey_Seal_X1             ---------------------TGSTPGYE-GPGEGDENISRKPGNVLKTLDPILITIIAM 865
Bear                     ---------------------TGSTPGYE-GPGEGDENISRKPGNVLKTLDPILITIIAM 865
```

```
Cod_X1                SALGVFLGAICGVVLYCACSHGAMSDRNLSALENYNFELVDGVKLKKDKLNVQNSYSEA- 924
Frog                  SALGVLLGAICGVVLYCACWHNGMSERNLSALENYNFELVDGVKLKKGQIKHTELIFRSM 928
Tiger_snake_X1        SALGVLLGAICGVILYCACWHNGMSDRNLSALENYNFELVDGVKLKKDKLNTQNTYSEA- 914
Rock_Dove             SALGVLLGAICGVVLYCACWHNGMSERNLSALENYNFELVDGVKLKKDKLNTQNSYSEA- 855
Chicken_X1            SALGVLLGAICGVVLYCACWHNGMSERNLSALENYNFELVDGVKLKKDKLNTQNSYSEA- 921
Duck                  SALGVLLGAICGVVLYCACWHNGMSERNLSALENYNFELVDGVKLKKDKLNTQNSYSEA- 922
Rabbit                SALGVLLGAVCGVVLYCACWHNGMSDRNLSALENYNFELVDGVKLKKDKLNTQSSYSEA- 922
Rat                   SALGVLLGAVCGVVLYCACWHNGMSERNLSALENYNFELVDGVKLKKDKLNPQSNYSEA- 927
Mouse                 SALGVLLGAVCGVVLYCACWHNGMSERNLSALENYNFELVDGVKLKKDKLNPQSNYSEA- 923
Bat_Little_Brown      SALGVLLGAVCGVVLYCACWHNGMSENYSSALENYNFELVDGVKLKKDKLNTQSTYSEA- 902
Elephant              SALGVLLGAVCGVVLYCACWHNGMSERNLSALENYNFELVDGVKLKKDKLTTQSTYSEA- 972
Lemur_X1              SALGVLLGAVCGVVLYCACWHNGMSERNLSALENYNFELVDGVKLKKDKLNTQNTYSEA- 924
Ground_Squirrel_X1    SALGVLLGAVCGVVLYCACWHNGMSERNLSALENYNFELVDGVKLKKDKLNTQSTYSEA- 923
Macaque_X1            SALGVLLGAVCGVVLYCACWHNGMSERNLSALENYNFELVDGVKLKKDKLNTQSTYSEA- 925
Gorilla_X1            SALGVLLGAVCGVVLYCACWHNGMSERNLSALENYNFELVDGVKLKKDKLNAQSTYSEA- 923
Human_X1              SALGVLLGAVCGVVLYCACWHNGMSERNLSALENYNFELVDGVKLKKDKLNTQSTYSEA- 923
Human_X6              SALGVLLGAVCGVVLYCACWHNGMSERNLSALENYNFELVDGVKLKKDKLNTQSTYSEA- 742
Camel_X1              SALGVLLGAVCGVVLYCACWHNGMSERNLSALENYNFELVDGVKLKKDKLNSQSTYSEA- 924
Bat_Pipistrellus_X1   SALGVLLGAVCGVVLYCACWHNGMSERNLSALENYNFELVDGVKLKKDKLNTQNTYSEA- 924
Beluga_X1             SALGVLLGAVCGVVLYCACWHNGMSERNLSALENYNFELVDGVKLKKDKLNPQSTYSEA- 924
Dolphin_X1            SALGVLLGAVCGVVLYCACWHNGMSERNLSALENYNFELVDGVKLKKDKLNPQSTYSEA- 924
Cow                   SALGVLLGAVCGVVLYCACWHNGMSERNLSALENYNFELVDGVKLKKDKLNPQSTYSEA- 924
Goat                  SALGVLLGAVCGVVLYCACWHNGMSERNLSALENYNFELVDGVKLKKDKLNPQSTYSEA- 924
Sheep                 SALGVLLGAVCGVVLYCACWHNGMSERNLSALENYNFELVDGVKLKKDKLNPQSTYSEA- 924
Pig                   SALGVLLGAVCGVVLYCACWHNGMSERNLSALENYNFELVDGVKLKKDKLNPQSTYSEA- 924
Horse_X1              SALGVLLGAVCGVVLYCACWHNGMSERNLSALENYNFELVDGVKLKKDKLNTQSSYSEA- 924
Horse_X2              SALGVLLGAVCGVVLYCACWHNGMSERNLSALENYNFELVDGVKLKKDKLNTQSSYSEA- 923
Dog_X1                SALGVLLGAVCGVVLYCACWHNGMSERNLSALENYNFELVDGVKLKKDKLNTQSTYSEA- 926
Dog_X2                SALGVLLGAVCGVVLYCACWHNGMSERNLSALENYNFELVDGVKLKKDKLNTQSTYSEA- 925
American_mink         ------------------------------------------------------------ 614
Ferret_X1             SALGVLLGAVCGVVLYCACWHNGMSERNLSALENYNFELVDGVKLKKDKLNTQSTYSEA- 924
Tiger                 SALGVLLGAVCGVVLYCACWHNGMSERNLSALENYNFELVDGVKLKKDKLNTQSTYSEA- 1181
Cat_X4                SALGVLLGAVCGVVLYCACWHNGMSERNLSALENYNFELVDGVKLKKDKLNTQSTYSEA- 916
Cat_X3                SALGVLLGAVCGVVLYCACWHNGMSERNLSALENYNFELVDGVKLKKDKLNTQSTYSEA- 917
Cat_X1                SALGVLLGAVCGVVLYCACWHNGMSERNLSALENYNFELVDGVKLKKDKLNTQSTYSEA- 924
Cat_X2                SALGVLLGAVCGVVLYCACWHNGMSERNLSALENYNFELVDGVKLKKDKLNTQSTYSEA- 923
Grey_Seal_X1          SALGVLLGAVCGVVLYCACWHNGLSERNLSALENYNFELVDGVKLKKDKLNPQSTYSEA- 924
Bear                  SALGVLLGAVCGVVLYCACWHNGMSERNLSALENYNFELVDGVKLKKDKLNTQTTYSEA- 924
```

Cod_X1	-------------------------	924
Frog	NLEEEYGGFFADTLKRLQSIGIEGQ	953
Tiger_snake_X1	-------------------------	914
Rock_Dove	-------------------------	855
Chicken_X1	-------------------------	921
Duck	-------------------------	922
Rabbit	-------------------------	922
Rat	-------------------------	927
Mouse	-------------------------	923
Bat_Little_Brown	-------------------------	902
Elephant	-------------------------	972
Lemur_X1	-------------------------	924
Ground_Squirrel_X1	-------------------------	923
Macaque_X1	-------------------------	925
Gorilla_X1	-------------------------	923
Human_X1	-------------------------	923
Human_X6	-------------------------	742
Camel_X1	-------------------------	924
Bat_Pipistrellus_X1	-------------------------	924
Beluga_X1	-------------------------	924
Dolphin_X1	-------------------------	924
Cow	-------------------------	924
Goat	-------------------------	924
Sheep	-------------------------	924
Pig	-------------------------	924
Horse_X1	-------------------------	924
Horse_X2	-------------------------	923
Dog_X1	-------------------------	926
Dog_X2	-------------------------	925
American_mink	-------------------------	614
Ferret_X1	-------------------------	924
Tiger	-------------------------	1181
Cat_X4	-------------------------	916
Cat_X3	-------------------------	917
Cat_X1	-------------------------	924
Cat_X2	-------------------------	923
Grey_Seal_X1	-------------------------	924
Bear	-------------------------	924

Figure S3. Amino acid sequence alignment of neuropilin-1 polypeptides from a range of animal species. Accession Numbers of the polypeptide sequences used are given in Table 1. Alignments were carried out using Clustal Omega [1]. The last row shown is the consensus analysis given by the algorithm. Human sequence (variant 2) is highlighted in grey to aid comparison to other sequences. Important amino acids used for interaction with the SARS-CoV-2 viral proteins [6] are highlighted in yellow. Amino acid differences from the human sequence in this region are highlighted in blue. Polymorphisms reported in the human sequence are highlighted in purple.

```
CLUSTAL O(1.2.4) multiple sequence alignment

Darter_(fish)_b         ---------------------------------------------------------MDA 3
Trout_X1                ---------------------------------------------------------MDA 3
Darter_(fish)_a         -----------------------------------------MASGTPCPSVGRRLRLLEL 19
Frog                    -----------------------------------------------------------M 1
Chicken                 -----------------------------------------------------------M 1
Rock_dove               -----------------------------------------------------------M 1
Duck                    -----------------------------------------------------------M 1
Tiger_snake_X1          MATPPPSCRPLGSRWQVLCSRRVAQSRPPCFLRPTEPLLGTARASPGCPALLSLLAFMDR 60
Human-I2                -----------------------------------------------------------M 1
Bat                     -----------------------------------------------------------M 1
Rat                     -----------------------------------------------------------M 1
Mouse                   -----------------------------------------------------------M 1
Cow                     -----------------------------------------------------------M 1
Goat                    -----------------------------------------------------------M 1
Sheep                   -----------------------------------------------------------M 1
Pig                     -----------------------------------------------------------M 1
Elephant                -----------------------------------------------------------M 1
Camel_X1                -----------------------------------------------------------M 1
Human_I1                -----------------------------------------------------------M 1
Gorilla                 -----------------------------------------------------------M 1
Macaque                 -----------------------------------------------------------M 1
Ground_squirrel_X1      -----------------------------------------------------------M 1
Lemur                   -----------------------------------------------------------M 1
Beluga_X1               -----------------------------------------------------------M 1
Dolphin                 -----------------------------------------------------------M 1
Rabbit                  -----------------------------------------------------------M 1
Cat                     -----------------------------------------------------------M 1
Tiger                   ------------------------------------------------------------ 0
Horse                   -----------------------------------------------------------M 1
Grey_seal_X1            -----------------------------------------------------------M 1
Dog                     -----------------------------------------------------------M 1
Bear                    -----------------------------------------------------------M 1
American_mink           ------------------------------------------------------------ 0
Ferret_X1               -----------------------------------------------------------M 1


Darter_(fish)_b         RVVQ---LHLWTALVLLIAELKW----IEAAEVYTNTWAVQINGGPEEADRIARQHGFIN 56
Trout_X1                RLAL---LQLGTLLGLLSLALEFTAEPTAAEEVYTNTWAVHIEGGPQEADRISRKHGFIN 60
Darter_(fish)_a         LLGPLLVL------------LVSGLRPALGQKVYTNTWAVHIPGGPEEADQIASKHGFIN 67
Frog                    DLSPTLLLMLWTL-------LSVLFKEITGHKVYTNTWAVHISGGSAEADRLSRKYGFIN 54
Chicken                 DLRPCSLLLLWTL----VVALALLAREVLAQRIYTNTWAVLVPAGPLEANRLARKHGFLN 57
Rock_dove               DLRPCSLLLLWTL----VVALTLLAQEVLAQRIYTNTWAVLVPAGPQEADRLARKHGFLN 57
Duck                    DLRPCSLLLLWTL----LVAFVLLAQEVLAQRIYTNTWAVLVPAGPQEADRLARKHGFLN 57
Tiger_snake_X1          RLSL---LLFWW-AM--TVPLALLVSEGLSQHIYTNTWAVLVPAGLQEAERMARKHGFLN 114
Human-I2                ELRP---WLLWVVAA--TGTLVLLAADAQGQKVFTNTWAVRIPGGPAVANSVARKHGFLN 56
Bat                     ELRP---WLLWVIAA--AGPLVLLAAGAQGQQVFTNTWAVHISGGPAMADRLARKHGFLN 56
Rat                     ELRP---WLLWVVAA--AGALVLLAADARGQKIFTNTWAVHISGGPAVADSVARKHGFHN 56
Mouse                   ELRS---WLLWVVAA--AGAVVLLAADAQGQKIFTNTWAVHIPGGPAVADRVAQKHGFHN 56
Cow                     ELRP---WLFWVVAA--AGALVLLVADARGEKVFTNTWAVHIPGGPAVADRVARKHGFLN 56
Goat                    ELRP---WLFWVVAI--AGALVLLVADARGEKVFTNTWAVHIPGGPAVADRVARKHGFLN 56
Sheep                   ELRP---WLFWVVAI--AGALILLVADARGEKVFTNTWAVHIPGGPAVADRVAHKHGFLN 56
Pig                     ELRP---WLLWVVAA--AGALVLLAADARGQKVFTNTWAVHIPGGPAVADRVARKHGFVN 56
Elephant                ELRP---WVLWVVAA--AGALVLLAADACGQKVFTNTWAVHIPGGQTVADSLARKHGFLN 56
Camel_X1                ELRP---WLLWVVA---AGALVLLVADARGQKVFTNTWAVRIPGGPAVADSVARKHGFLN 55
Human_I1                ELRP---WLLWVVAA--TGTLVLLAADAQGQKVFTNTWAVRIPGGPAVANSVARKHGFLN 56
Gorilla                 ELRP---WLLWVVAA--TGTLVLLAADAQGQKVFTNTWAVRIPGGPAVANSVAQKHGFLN 56
Macaque                 ELRS---WLLWVVAA--TGTLVLLAADAQGQKVFTNTWAVHIPGGPAVANSVARKHGFLN 56
Ground_squirrel_X1      ELRP---WLLWVVAA--AGVLVLLTANACGQKVFTNTWAVHIPGGLAVADSVARKHGFHN 56
Lemur                   ELRP---WLLWVVAA--AGALVLLAAEARAQKVFTNTWAVHIPGGPAVADGVARKHGFLN 56
Beluga_X1               ELRP---WLLWVVAA--AGALVLLAADARGQKVFTNTWAVRIPGGPAVADSVARKHGFLN 56
Dolphin                 ELRP---WLLWVVAA--AGALVLLAADTRGQKVFTNTWAVRIPGGPAVADSVARKHGFLN 56
Rabbit                  ELRP---WLLWVLAT--AGALVLLAANACSQRVFTNTWAVRIPGGLAVADGVARKHGFLN 56
Cat                     ELRP---WLLWVVAA--AGALVLLAADACGQKVYTNTWAVHIPGGPAVADSLARKHGFLN 56
Tiger                   ------------------------------------------------------------ 0
Horse                   ELRP---WLLWVVAA--AIALVLLAADARGQKVFTNTWAVRIAGGPAVADSVARKHGFLN 56
Grey_seal_X1            ELRP---WLLWVVA---AGALVLLAADARAQKVYTNTWAVHVPGGPAVADSLARKHGFLN 55
Dog                     ELRP---WLLWVVAA--AGALVLLAADARGQKVYTNTWAVHIPGGPAVADSLARKHGFLN 56
Bear                    ELRP---WLLWVVAA--AGALVLLAADARAQKVYTNTWAVHIPGGPAVADSLARKHGFLN 56
American_mink           ------------------------------------------------------------ 0
Ferret_X1               ELRP---WLLWVVAA--AGALVLLAADARAQKVYTNTWAVHIPGGPAVADRLARKHGFLN 56
```

```
Darter_(fish)_b      HG---------------------------------NVFGDYYHFRHHAVEKRALSGHRGM  83
Trout_X1             HG---------------------------------NVFGGYYHFRHHTVVKRSLSGHRGT  87
Darter_(fish)_a      QG---------------------------------HVFGDYYHFRHRTVVKRSLSDHRGT  94
Frog                 HG---------------------------------QIFEDHYHFSHRAVMKRSLTPKRTR  81
Chicken              LG---------------------------------PIFGDYYHFQHRGVVKRSLSPHQPW  84
Rock_dove            LG---------------------------------PIFGDYYHFRHSGVVKRSLSPHQSW  84
Duck                 LG---------------------------------PIFGDYYHFRHRGVVKRSLSPHQPW  84
Tiger_snake_X1       LG---------------------------------PIIGDYYHFYHRAVAKRSLSPHWSW  141
Human-I2             LG---------------------------------QIFGDYYHFWHRGVTKRSLSPHRPR  83
Bat                  QG---------------------------------QIFGDYYHFWHRAVTKRSLSPHRLR  83
Rat                  LG---------------------------------QIFGDYYHFWHRAVTKRSLSPHRPR  83
Mouse                LG---------------------------------QIFGDYYHFWHRAVTKRSLSPHRPR  83
Cow                  LG---------------------------------QIFGDYYHFWHRAVTKRSLSPHRLR  83
Goat                 LG---------------------------------QIFGDYYHFWHRAVTKRSLSPHLSR  83
Sheep                LG---------------------------------QIFGDYYHFWHRAVTKRSLSPHLPR  83
Pig                  LG---------------------------------QIFGDYYHFWHRAVTKRSLSPHRLR  83
Elephant             LG---------------------------------QIFGDYYHFWHRAVTKRSLSPHRPR  83
Camel_X1             LGQGHLGRKRVLARDESRVLSCPWASNSVDQHIPGRIFGDYYHFWHRAVTKRSLSPHHPR  115
Human_I1             LG---------------------------------QIFGDYYHFWHRGVTKRSLSPHRPR  83
Gorilla              LG---------------------------------QIFGDYYHFWHRGVTKRSLSPHRPR  83
Macaque              LG---------------------------------QIFGDYYHFWHRGVTKRSLSPHRPR  83
Ground_squirrel_X1   LG---------------------------------QIFGDYYHFWHRAVTKRSLSPHRLR  83
Lemur                LG---------------------------------QIFGDYYHFWHRAVTKRSLSPHRPR  83
Beluga_X1            LG---------------------------------QIFGDYYHFWHRAVTKRSLSPHRPR  83
Dolphin              LG---------------------------------QIFGDYYHFWHRAVTKRSLSPHRPR  83
Rabbit               LG---------------------------------QIFGDYYHFWHRAVTKRSLSPHRLR  83
Cat                  LG---------------------------------QIFGDYYHFWHRAVTKRSLSPHRPR  83
Tiger                MG---------------------------------AIFGDYYHFWHRAVTKRSLSPHRPR  27
Horse                LG---------------------------------QIFSDYYHFWHRAVTKRSLSPHRPR  83
Grey_seal_X1         LG---------------------------------QIFGDYYHFWHRAVTKRSLSPHRLR  82
Dog                  LG---------------------------------QIFGDYYHFWHRAVTKRSLSPHHPR  83
Bear                 LG---------------------------------QIFGDYYHFWHRAVTKRSLSPHHPR  83
American_mink        ------------------------------------------------------------  0
Ferret_X1            LG---------------------------------QIFGDYYHFWHRAVTKRSLSPHHPR  83
Darter_(fish)_b      HIRLQQEPQVQWAEQQVVKIRKKRDIYEDPTDPDFPKQWYLT---NPTHEDLNTKVAWAQ  140
Trout_X1             HVRLQKEPQVRWAEQQVSRRRKKRDVYTEPSDPKFPQQWYLVSKSNPSQADLNAKGAWSQ  147
Darter_(fish)_a      QVRLSRDPKVTWAEQQVSKRRKKRDVFAEPLDPKFRDQWYL-Y--NSNHRDLNAKSAWQL  151
Frog                 QVLLKREPQVHWLEQQVAKKRKKRDIFIDPTDPKFMQQWYL-L--DTNRHDLHVKEAWEQ  138
Chicken              HSRLAREPQVHWLEQQVAKRRTKRDIFMEPTDPKFPQQWYL-Y--NTNQRDLNVRQAWEQ  141
Rock_dove            HSRLAREPQVQWLEQQVAKRRTKRDVFMEPTDPKFPQQWYL-Y--NTNQRDLNVRQAWAQ  141
Duck                 HSRLAREPQVHWLEQQVAKRRTKRDVFMEPTDPKFPQQWYL-Y--NTNQRDLNVRQAWEQ  141
Tiger_snake_X1       HSSLSREPQVLWLEQQVAKRRTKRDAFIEPTDPKFPQQWYL-S--NNNQRDLNVQEAWQQ  198
Human-I2             HSRLQREPQVQWLEQQVAKRRTKRDVYQEPTDPKFPQQWYL-S--GVTQRDLNVKAAWAQ  140
Bat                  HSRLQREPQVQWLEQQVAKRRTKRDLYLEPTDPKFPQQWYL-S--GVAQRDLNVKEAWAQ  140
Rat                  HSRLQREPQVKWLEQQVAKQRAKRDVYQEPTDPKFPQQWYL-S--GVTQRDLNVKEAWAQ  140
Mouse                HSRLQREPQVKWLEQQVAKRRAKRDVYQEPTDPKFPQQWYL-S--GVTQRDLNVKEAWAQ  140
Cow                  HNRLQREPQVKWLEQQVAKRRAKRDIYQEPTDPKFPQQWYL-S--GVTQRDLNVKEAWAQ  140
Goat                 HNRLQREPQVKWLEQQVAKRRAKRDIYQEPTDPKFPQQWYL-S--GVTQRDLNVKEAWAQ  140
Sheep                HNRLQREPQVKWLEQQVAKRRAKRDIYQEPTDPKFPQQWYL-S--GVTQRDLNVKEAWGQ  140
Pig                  HSRLQREPQIQWLEQQVAKRRTKRDVYLEPTDPKFPQQWYL-S--GVTQRDLNVKEAWAQ  140
Elephant             HSRLQREPQVQWLEQQVAKRRTKRDVYQEPTDPKFPQQWYL-S--GITQRDLNVKEAWVQ  140
Camel_X1             HSRLQREPRVEWLEQQVAKRRTKRDIYQEPTDPKFPQQWYL-S--GITQRDLNVKDAWAQ  172
Human_I1             HSRLQREPQVQWLEQQVAKRRTKRDVYQEPTDPKFPQQWYL-S--GVTQRDLNVKAAWAQ  140
Gorilla              HSRLQREPQVQWLEQQVAKRRTKRDVYQEPTDPKFPQQWYL-S--GVTQRDLNVKAAWAQ  140
Macaque              HSRLQREPQVQWLEQQVAKRRTKRDLYQEPTDPKFPQQWYL-S--GVTQRDLNVKEAWAQ  140
Ground_squirrel_X1   HSRLQREPQVLWLEQQVAKRRSKRDVYQEPTDPKFPQQWYL-S--GVTQRDLNVKEAWAQ  140
Lemur                HSRLQREPQVQWLEQQVAKRRTKRDVYQEPTDPKFPQQWYL-S--GVTQRDLNVKEAWAQ  140
Beluga_X1            HSRLQREPQVQWLEQQVAKRRTKRDVYQEPTDPKFPQQWYL-S--GVTQRDLNVKEAWAQ  140
Dolphin              HSRLQREPQVQWLEQQVAKRRTKRDVYQEPTDPKFPQQWYL-S--GVTQRDLNVKEAWAQ  140
Rabbit               HSRLQREPQVQWLEQQVAKRRSKRDVYQEPTDPKFPQQWYL-S--GVTQRDLNVKEAWAQ  140
Cat                  HSRLQREPQVQWLEQQVAKRRTKRDVYQEPTDPKFPQQWYL-S--GVTQRDLNVKEAWAQ  140
Tiger                HSRLQREPQVQWLEQQVAKRRTKRDVYQEPTDPKFPQQWYL-S--GVTQRDLNVKEAWAQ  84
Horse                HSRLQREPQVQWLEQQVAKRRTKRDVYQEPTDPKFPQQWYL-S--GITQRDLNVKDAWAQ  140
Grey_seal_X1         HSRLQREPQVQWLEQQVVKRRTKRDVYQEPTDPKFPQQWYL-S--GVTQRDLNVKEAWAQ  139
Dog                  HSRLQREPQVQWLEQQVAKRRIKRDVYQEPTDPKFPQQWYL-S--GVTQRDLNVKEAWAQ  140
Bear                 HSRLQREPQVQWLEQQVAKRRTKRDVYQEPTDPKFPQQWYL-S--GVTQRDLNVKEAWAQ  140
American_mink        ------------------------------------------------------------  0
Ferret_X1            HSRLQREPQVQWLEQQVAKRRTKRDVYQEPTDPKFPQQWYLQS--GVTQRDLNVKEAWAQ  141
```

```
Darter_(fish)_b       GYTGRGVVVSILDDGIEKDHPDLVSNYDPEASYDVNDGDADPQPRYTQRNENRHGTRCAG  200
Trout_X1              GYTGRGVVVTILDDGIEKDHPDLATNYDPEASYDVNDGDSDPQPRYTQRNENRHGTRCAG  207
Darter_(fish)_a       GYTGKGVVVSILDDGIEKNHPDLMQNYDPDASYDVNDGDPDPQPRYTQLNDNRHGTRCAG  211
Frog                  GFTGKGIVVSILDDGIEKNHPDLQANYDPAASYDVNDQDPDPQPRYTQLNDNRHGTRCAG  198
Chicken               GYTGKGIVVSILDDGIEKNHPDLEANYDPGASFDVNDQDPDPQPRYTQMNDNRHGTRCAG  201
Rock_dove             GYTGKGIVVSILDDGIEKNHPDLEGNYDPGASFDVNDQDPDPQPRYTQMNDNRHGTRCAG  201
Duck                  GYTGKGIVVSILDDGIEKNHPDLEANYDPGASFDVNDQDPDPQPRYTQMNDNRHGTRCAG  201
Tiger_snake_X1        GYTGRGVVVSILDDGIEKNHPDLEANYDPAASFDVNDQDPDPQPRYTQTNDNRHGTRCAG  258
Human-I2              GYTGHGIVVSILDDGIEKNHPDLAGNYDPGASFDVNDQDPDPQPRYTQMNDNRHGTRCAG  200
Bat                   GFTGRGIVVSILDDGIEKNHPDLAGNYDPGASFDVNDQDPDPQPRYTQMNDNRHGTRCAG  200
Rat                   GFTGRGIVVSILDDGIEKNHPDLAGNYDPGASFDVNDQDPDPQPRYTQMNDNRHGTRCAG  200
Mouse                 GFTGHGIVVSILDDGIEKNHPDLAGNYDPGASFDVNDQDPDPQPRYTQMNDNRHGTRCAG  200
Cow                   GYTGRGIVVSILDDGIEKNHPDLAGNYDPGASFDVNDQDPDPQPRYTQMNDNRHGTRCAG  200
Goat                  GYTGRGIVVSILDDGIEKNHPDLAGNYDPGASFDVNDQDPDPQPRYTQMNDNRHGTRCAG  200
Sheep                 GYTGRGIVVSILDDGIEKNLPVLAGNYDPGASFDVNDQDPDPQPRVTQMNDNRHGTRCAG  200
Pig                   GYTGRGIVVSILDDGIEKNHPDLAGNYDPGASFDVNDQDPDPQPRYTQMNDNRHGTRCAG  200
Elephant              GYTGRGIVVSILDDGIEKNHPDLAGNYDPGASFDVNDQDPDPQPRYTQLNDNRHGTRCAG  200
Camel_X1              GYTGRGIVVSILDDGIEKNHPDLAGNYDPGASFDVNDQDPDPQPRYTQMNDNRHGTRCAG  232
Human_I1              GYTGHGIVVSILDDGIEKNHPDLAGNYDPGASFDVNDQDPDPQPRYTQMNDNRHGTRCAG  200
Gorilla               GYTGHGIVVSILDDGIEKNHPDLAGNYDPGASFDVNDQDPDPQPRYTQMNDNRHGTRCAG  200
Macaque               GYTGHGIVVSILDDGIEKNHPDLAGNYDPGASFDVNDQDPDPQPRYTQMNDNRHGTRCAG  200
Ground_squirrel_X1    GYTGHGIVVSILDDGIEKNHPDLAGNYDPGASFDVNDQDPDPQPRYTQMNDNRHGTRCAG  200
Lemur                 GYTGRGIVVSILDDGIEKNHPDLAGNYDPGASFDVNDQDPDPQPRYTQMNDNRHGTRCAG  200
Beluga_X1             GYTGRGIVVSILDDGIEKNHPDLAGNYDPGASFDVNDQDPDPQPRYTQMNDNRHGTRCAG  200
Dolphin               GYTGRGIVVSILDDGIEKNHPDLAGNYDPGASFDVNDQDPDPQPRYTQMNDNRHGTRCAG  200
Rabbit                GYTGHGIVVSILDDGIEKNHPDLAGNYDPGASFDVNDQDPDPQPRYTQMNDNRHGTRCAG  200
Cat                   GYTGHGIVVSILDDGIEKNHPDLAGNYDPGASFDVNDQDPDPQPRYTQMNDNRHGTRCAG  200
Tiger                 GYTGHGIVVSILDDGIEKNHPDLAGNYDPGASFDVNDQDPDPQPRYTQMNDNRHGTRCAG  144
Horse                 GYTGRGIVVSILDDGIEKNHPDLAGNYDPGASFDVNDQDPDPQPRYTQMNDNRHGTRCAG  200
Grey_seal_X1          GYTGHGIVVSILDDGIEKNHPDLAGNYDPGASFDVNDQDPDPQPRYTQMNDNRHGTRCAG  199
Dog                   GYTGHGIVVSILDDGIEKNHPDLAGNYDPGASFDVNDQDPDPQPRYTQMNDNRHGTRCAG  200
Bear                  GYTGHGIVVSILDDGIEKNHPDLAGNYDPGASFDVNDQDPDPQPRYTQMNDNRHGTRCAG  200
American_mink         ------------------------------------------------------------  0
Ferret_X1             GYTGHGIVVSILDDGIEKNHPDLAGNYDPGASFDVNDQDPDPQPRYTQMNDNRHGTRCAG  201

Darter_(fish)_b       EVAAAADNGVCGVGVAYNAKIGGVRMLDGEVTDVVEAHSLSLNPQHIHIYSASWGPEDDG  260
Trout_X1              EVAAAANNNVCGVGVAYNTKIGGVRMLDGEVTDMVEAHSLSLNSQHIHIYSASWGPEDDG  267
Darter_(fish)_a       EVAAVANNGICGVGVAYNAKIGGVRMLDGEVTDMVEAQSLSLNPQHIDIYSASWGPEDDG  271
Frog                  EVAAVANNDICGVGIAYNANIGGVRMLDGEVTDAVEARSLGLNPNHIHIYSASWGPEDDG  258
Chicken               EVAAVANNGICGVGVAYNARIGGVRMLDGEVTDAVEAHSLGLNPNHIHIYSASWGPEDDG  261
Rock_dove             EVAAVANNGICGVGVAYNARIGGVRMLDGEVTDAVEAHSLGLNPNHIHIYSASWGPEDDG  261
Duck                  EVAAVANNGICGVGVAYNARIGGVRMLDGEVTDAVEAHSLGLNPNHIHIYSASWGPEDDG  261
Tiger_snake_X1        EVAAVANNGICGVGVAYNAKIGGVRMLDGEVTDAVEARSLGLNPDHIHIYSASWGPEDDG  318
Human-I2              EVAAVANNGVCGVGVAYNARIGGVRMLDGEVTDAVEARSLGLNPNHIHIYSASWGPEDDG  260
Bat                   EVAAVANNGVCGVGVAYNARIGGVRMLDGEVTDAVEARSLGLNPNHIHIYSASWGPEDDG  260
Rat                   EVAAVANNGVCGVGVAYNARIGGVRMLDGEVTDAVEARSLGLNPNHIHIYSASWGPEDDG  260
Mouse                 EVAAVANNGVCGVGVAYNARIGGVRMLDGEVTDAVEARSLGLNPNHIHIYSASWGPEDDG  260
Cow                   EVAAVANNGVCGVGVAYNARIGGVRMLDGEVTDAVEARSLGLNPNHIHIYSASWGPEDDG  260
Goat                  EVAAVANNGVCGVGVAYNARIGGVRMLDGEVTDAVEARSLGLNPNHIHIYSASWGPEDDG  260
Sheep                 EVAAVANNGVCGVGVAYNARIGGVRMLDGEVTDAVEARSLGLNPNHIHIYSASWGPEDDG  260
Pig                   EVAAVANNGVCGVGVAYNARIGGVRMLDGEVTDAVEARSLGLNPNHIHIYSASWGPEDDG  260
Elephant              EVAAVANNGVCGVGVAYNARIGGVRMLDGEVTDAVEARSLGLNPNHIHIYSASWGPEDDG  260
Camel_X1              EVAAVANNGVCGVGVAYNARIGGVRMLDGEVTDAVEARSLGLNPNHIHIYSASWGPEDDG  292
Human_I1              EVAAVANNGVCGVGVAYNARIGGVRMLDGEVTDAVEARSLGLNPNHIHIYSASWGPEDDG  260
Gorilla               EVAAVANNGVCGVGVAYNARIGGVRMLDGEVTDAVEARSLGLNPNHIHIYSASWGPEDDG  260
Macaque               EVAAVANNGVCGVGVAYNARIGGVRMLDGEVTDAVEARSLGLNPNHIHIYSASWGPEDDG  260
Ground_squirrel_X1    EVAAVANNGVCGVGVAYNARIGGVRMLDGEVTDAVEARSLGLNPNHIHIYSASWGPEDDG  260
Lemur                 EVAAVANNGVCGVGVAYNARIGGVRMLDGEVTDAVEARSLGLNPNHIHIYSASWGPEDDG  260
Beluga_X1             EVAAVANNGVCGVGVAYNARIGGVRMLDGEVTDAVEARSLGLNPNHIHIYSASWGPEDDG  260
Dolphin               EVAAVANNGVCGVGVAYNARIGGVRMLDGEVTDAVEARSLGLNPNHIHIYSASWGPEDDG  260
Rabbit                EVAAVANNGVCGVGVAYNARIGGVRMLDGEVTDAVEARSLGLNPNHIHIYSASWGPEDDG  260
Cat                   EVAAVANNGVCGVGVAYNARIGGVRMLDGEVTDAVEARSLGLNPNHIHIYSASWGPEDDG  260
Tiger                 EVAAVANNGVCGVGVAYNARIGGVRMLDGEVTDAVEARSLGLNPNHIHIYSASWGPEDDG  204
Horse                 EVAAVANNGVCGVGVAYNARIGGVRMLDGEVTDAVEARSLGLNPNHIHIYSASWGPEDDG  260
Grey_seal_X1          EVAAVANNGVCGVGVAYNARIGGVRMLDGEVTDAVEARSLGLNPNHIHIYSASWGPEDDG  259
Dog                   EVAAVANNGVCGVGVAYNARIGGVRMLDGEVTDAVEARSLGLNPNHIHIYSASWGPEDDG  260
Bear                  EVAAVANNGVCGVGVAYNARIGGVRMLDGEVTDAVEARSLGLNPNHIHIYSASWGPEDDG  260
American_mink         ------------------------------------------------------------  0
Ferret_X1             EVAAVANNGVCGVGVAYNARIGGVRMLDGEVTDAVEARSLGLNPNHIHIYSASWGPEDDG  261
                      ****.*:*.:****:***:.************* ***:**.** :**.************
```

```
Darter_(fish)_b        KSLDGPAKLAKEAFLQGITKGRDGQGSIFVWASGNGGREQDSCNCDGYTNSIYTLSISST  320
Trout_X1               KTVDGPAKLAKEAFLRGVTEGRGRLGSIFVWASGNGGREKDSCNCDGYTNSIYTLSISST  327
Darter_(fish)_a        KTVDGPAKLAKEAFLRGVTEGRSGLGSIFVWASGNGGREKDSCNCDGYTNSIYTLSISSS  331
Frog                   KTVDGPAKLAEEAFYRRVTQGRGGLGSIYVWASGNGGRQHDSCNCDGYTNSIYTLSISST  318
Chicken                KTVDGPARLAEEAFFRGVSQGRGGLGSIFVWASGNGGREHDSCNCDGYTNSIYTLSISST  321
Rock_dove              KTVDGPARLAEEAFFRGVSQGRGGLGSIFVWASGNGGREHDSCNCDGYTNSIYTLSISST  321
Duck                   KTVDGPARLAEEAFFRGVSQGRGGLGSIFVWASGNGGREHDSCNCDGYTNSIYTLSISST  321
Tiger_snake_X1         KTVDGPAHLAEEAFRRGVSQGRNGLGSIFVWASGNGGRDRDSCNCDGYTNSIYTLSISST  378
Human-I2               KTVDGPARLAEEAFFRGVSQGRGGLGSIFVWASGNGGREHDSCNCDGYTNSIYTLSISSA  320
Bat                    KTVDGPARLAEEAFFRGVSQGRGGLGSIFVWASGNGGREHDSCNCDGYTNSIYTLSISSA  320
Rat                    KTVDGPARLAEEAFFRGVSQGRGGLGSIFVWASGNGGREHDSCNCDGYTNSIYTLSISSA  320
Mouse                  KTVDGPARLAEEAFFRGVSQGRGGLGSIFVWASGNGGREHDSCNCDGYTNSIYTLSISSA  320
Cow                    KTVDGPAHLAEEAFFRGVSQGRGGLGSIFVWASGNGGREHDSCNCDGYTNSIYTLSISSA  320
Goat                   KTVDGPAHLAEEAFFRGVSQGRGGLGSIFVWASGNGGREHDSCNCDGYTNSIYTLSISSA  320
Sheep                  KTVDGPAHLAEEAFFRGVSQGRGGLGSIFVWASGNGGREHDSCNCDGYTNSIYTLSISSA  320
Pig                    KTVDGPARLAEEAFFRGVSQGRGGLGSIFVWASGNGGREHDSCNCDGYTNSIYTLSISSA  320
Elephant               KTVDGPARLAEEAFFRGVSQGRGGLGSIFVWASGNGGREHDSCNCDGYTNSIYTLSISSA  320
Camel_X1               KTVDGPARLAEEAFFRGVSQGRGGLGSIFVWASGNGGREHDSCNCDGYTNSIYTLSISSA  352
Human_I1               KTVDGPARLAEEAFFRGVSQGRGGLGSIFVWASGNGGREHDSCNCDGYTNSIYTLSISSA  320
Gorilla                KTVDGPARLAEEAFFRGVSQGRGGLGSIFVWASGNGGREHDSCNCDGYTNSIYTLSISSA  320
Macaque                KTVDGPARLAEEAFFRGVSQGRGGLGSIFVWASGNGGREHDSCNCDGYTNSIYTLSISSA  320
Ground_squirrel_X1     KTVDGPARLAEEAFFRGVSQGRGGLGSIFVWASGNGGREHDSCNCDGYTNSIYTLSISSA  320
Lemur                  KTVDGPARLAEEAFFRGVSQGRGGLGSIFVWASGNGGREHDSCNCDGYTNSIYTLSISSA  320
Beluga_X1              KTVDGPARLAEEAFFRGVSQGRGGLGSIFVWASGNGGREHDSCNCDGYTNSIYTLSISSA  320
Dolphin                KTVDGPARLAEEAFFRGVSQGRGGLGSIFVWASGNGGREHDSCNCDGYTNSIYTLSISSA  320
Rabbit                 KTVDGPARLAEEAFFRGVSQGRGGLGSIFVWASGNGGREHDSCNCDGYTNSIYTLSISSA  320
Cat                    KTVDGPARLAEEAFFRGVSQGRGGLGSIFVWASGNGGREHDSCNCDGYTNSIYTLSISSA  320
Tiger                  KTVDGPARLAEEAFFRGVSQGRGGLGSIFVWASGNGGREHDSCNCDGYTNSIYTLSISSA  264
Horse                  KTVDGPARLAEEAFFRGVSQGRGGLGSIFVWASGNGGREHDSCNCDGYTNSIYTLSISSA  320
Grey_seal_X1           KTVDGPARLAEEAFFRGVSQGRGGLGSIFVWASGNGGREHDSCNCDGYTNSIYTLSISSA  319
Dog                    KTVDGPARLAEEAFFRGVSQGRGGLGSIFVWASGNGGREHDSCNCDGYTNSIYTLSISSA  320
Bear                   KTVDGPARLAEEAFFRGVSQGRGGLGSIFVWASGNGGREHDSCNCDGYTNSIYTLSISSA  320
American_mink          ----------------------------------------------GYTNSIYTLSISSA  14
Ferret_X1              KTVDGPARLAEEAFFRGVSQGRGGLGSIFVWASGNGGREHDSCNCDGYTNSIYTLSISSA  321
                                                                     *************:

Darter_(fish)_b        TQSGNVPWYSEPCSSTLATTFSSGNPGEKQIVTTDLRQKCTDSHTGTSASAPLAAGIIAL  380
Trout_X1               TQYGMVPWYSEACSSTLATTFSSGNPNEKQIVTTDLRQKCTDSHTGTSASAPLAAGIIAL  387
Darter_(fish)_a        TQNGNVPWYSEACSSTLATTYSSGNLNEKQIVTTDLKSKCTDSHTGTSASAPLAAGIIAL  391
Frog                   TQTGNVPWYSEACSSTLATTYSSGNQNEKQIVTTDLRQKCTDSHTGTSASAPLAAGIIAL  378
Chicken                TQYGNVPWYSEACSSTLATTYSSGNQNEKQIVTTDLRQKCTESHTGTSASAPLAAGIIAL  381
Rock_dove              TQYGNVPWYSEACSSTLATTYSSGNQNEKQIVTTDLRQKCTESHTGTSASAPLAAGIIAL  381
Duck                   TQYGNVPWYSEACSSTLATTYSSGNQNEKQIVTTDLRQKCTESHTGTSASAPLAAGIIAL  381
Tiger_snake_X1         TQYGNVPWYSEACSSTLASTYSSGSQNEKQIVTTDLWQKCTESHTGTSASAPLAAGIIAL  438
Human-I2               TQFGNVPWYSEACSSTLATTYSSGNQNEKQIVTTDLRQKCTESHTGTSASAPLAAGIIAL  380
Bat                    TQFGNVPWYSEACSSTLATTYSSGNQNEKQIVTTDLRQKCTESHTGTSASAPLAAGIIAL  380
Rat                    TQFGNVPWYSEACSSTLATTYSSGNQNEKQIVTTDLRQKCTESHTGTSASAPLAAGIIAL  380
Mouse                  TQFGNVPWYSEACSSTLATTYSSGNQNEKQIVTTDLRQKCTESHTGTSASAPLAAGIIAL  380
Cow                    TQFGNVPWYSEACSSTLATTYSSGNQNEKQIVTTDLRQKCTESHTGTSASAPLAAGIIAL  380
Goat                   TQFGNVPWYSEACSSTLATTYSSGNQNEKQIVTTDLRQKCTESHTGTSASAPLAAGIIAL  380
Sheep                  TQFGNVPWYSEACSSTLATTYSSGNQNEKQIVTTDLRQKCTESHTGTSASAPLAAGIIAL  380
Pig                    TQFGNVPWYSEACSSTLATTYSSGNQNEKQIVTTDLRQKCTESHTGTSASAPLAAGIIAL  380
Elephant               TQFGNVPWYSEACSSTLATTYSSGNQNEKQIVTTDLRQKCTESHTGTSASAPLAAGIIAL  380
Camel_X1               TQFGNVPWYSEACSSTLATTYSSGNQNEKQIVTTDLRQKCTESHTGTSASAPLAAGIIAL  412
Human_I1               TQFGNVPWYSEACSSTLATTYSSGNQNEKQIVTTDLRQKCTESHTGTSASAPLAAGIIAL  380
Gorilla                TQFGNVPWYSEACSSTLATTYSSGNQNEKQIVTTDLRQKCTESHTGTSASAPLAAGIIAL  380
Macaque                TQFGNVPWYSEACSSTLATTYSSGNQNEKQIVTTDLRQKCTESHTGTSASAPLAAGIIAL  380
Ground_squirrel_X1     TQFGNVPWYSEACSSTLATTYSSGNQNEKQIVTTDLRQKCTESHTGTSASAPLAAGIIAL  380
Lemur                  TQLGNVPWYSEACSSTLATTYSSGNQNEKQIVTTDLRQKCTESHTGTSASAPLAAGIIAL  380
Beluga_X1              TQSGNVPWYSEACSSTLATTYSSGNQNEKQIVTTDLRQKCTESHTGTSASAPLAAGIIAL  380
Dolphin                TQSGNVPWYSEACSSTLATTYSSGNQNEKQIVTTDLRQKCTESHTGTSASAPLAAGIIAL  380
Rabbit                 TQLGNVPWYSEACSSTLATTYSSGNQNEKQIVTTDLRQKCTESHTGTSASAPLAAGIIAL  380
Cat                    TQLGNVPWYSEACSSTLATTYSSGSQNEKQIVTTDLRQKCTESHTGTSASAPLAAGIIAL  380
Tiger                  TQLGNVPWYSEACSSTLATTYSSGNQNEKQIVTTDLRQKCTESHTGTSASAPLAAGIIAL  324
Horse                  TQLGNVPWYSEACSSTLATTYSSGNQNEKQIVTTDLRQKCTESHTGTSASAPLAAGIIAL  380
Grey_seal_X1           TQLGNVPWYSEACSSTLATTYSSGNQNEKQIVTTDLRQKCTESHTGTSASAPLAAGIIAL  379
Dog                    TQLGNVPWYSEACSSTLASTYSSGNQNEKQIVTTDLRQKCTESHTGTSASAPLAAGIIAL  380
Bear                   TQLGNVPWYSEACSSTLATTYSSGNQNEKQIVTTDLRQKCTESHTGTSASAPLAAGIIAL  380
American_mink          TQLGNVPWYSEACSSTLATTYSSGNQNEKQIVTTDLRQKCTESHTGTSASAPLAAGIIAL  74
Ferret_X1              TQLGNVPWYSEACSSTLATTYSSGNQNEKQIVTTDLRQKCTESHTGTSASAPLAAGIIAL  381
                       ** * ****** ******:*:***. .********* .***:******************
```

```
Darter_(fish)_b         ALEANMNLTWRDMQHLVVRTSRPGHLSAGDWKTNGVGRRVSHSYGYGLLDAGAMVALAQN  440
Trout_X1                ALEANMNLTWRDMQHLVVRTSHPAHLSTDDWSTNGVGRRVSHSYGYGLLDAGAMVALAQN  447
Darter_(fish)_a         ALEANKNLTWRDMQHLVVRTSHPAHLLTNDWRTNGVGRKVSHSYGYGLLDASAIVALAET  451
Frog                    ALEANKNLTWRDMQHLVVQTSNPASLNANDWITNGVGRKVSHSYGYGLLDAGAMVAMAKN  438
Chicken                 ALEANKNLTWRDMQHLVVQTSKPAHLNANDWVTNGVGRKVSHSYGYGLLDAGAMVSLARN  441
Rock_dove               ALEANKNLTWRDMQHLVVHTSKPAHLNANDWVTNGVGRKVSHSYGYGLLDAGAMVSLAKN  441
Duck                    ALEANKNLTWRDMQHLVVQTSKPAHLNANDWVTNGVGRKVSHSYGYGLLDAGAMVSLARN  441
Tiger_snake_X1          ALEANKNLTWRDMQHLVVRTSKPAHLNTNDWATNGVGRKVSHSYGYGLLDAGAMVALAKN  498
Human-I2                TLEANKNLTWRDMQHLVVQTSKPAHLNANDWATNGVGRKVSHSYGYGLLDAGAMVALAQN  440
Bat                     TLEANKNLTWRDMQHLVVQTSKPAHLNANDWATNGVGRKVSHSYGYGLLDAGAMVALAQN  440
Rat                     TLEANKNLTWRDMQHLVVQTSKPAHLNANDWATNGVGRKVSHSYGYGLLDAGAMVALAQN  440
Mouse                   TLEANKNLTWRDMQHLVVQTSKPAHLNADDWATNGVGRKVSHSYGYGLLDAGAMVALAQN  440
Cow                     TLEANKNLTWRDMQHLVVRTSKPAHLNANDWATNGVGRKVSHSYGYGLLDAGAMVALAQN  440
Goat                    TLEANKNLTWRDMQHLVVRTSKPAHLNANDWATNGVGRKVSHSYGYGLLDAGAMVALAQN  440
Sheep                   TLEANKNLTWRDMQHLVVRTSKPAHLNANDWATNGVGRKVSHSYGYGLLDAGAMVALAQN  440
Pig                     TLEANKNLTWRDMQHLVVQTSKPAHLNANDWTTNGVGRKVSHSYGYGLLDAGAMVALAQN  440
Elephant                TLEANKNLTWRDMQHLVVQTSKPAHLNANDWATNGVGRKVSHSYGYGLLDAGAMVALAQN  440
Camel_X1                TLEANKNLTWRDMQHLVVQTSKPAHLNANDWATNGVGRKVSHSYGYGLLDAGAMVALAQN  472
Human_I1                TLEANKNLTWRDMQHLVVQTSKPAHLNANDWATNGVGRKVSHSYGYGLLDAGAMVALAQN  440
Gorilla                 TLEANKNLTWRDMQHLVVQTSKPAHLNANDWATNGVGRKVSHSYGYGLLDAGAMVALAQN  440
Macaque                 TLEANKNLTWRDMQHLVVQTSKPAHLNANDWATNGVGRKVSHSYGYGLLDAGAMVALAQN  440
Ground_squirrel_X1      TLEANKNLTWRDMQHLVVQTSKPAHLNANDWATNGVGRKVSHSYGYGLLDAGAMVALAQN  440
Lemur                   TLEANKNLTWRDMQHLVVQTSKPAHLNANDWATNGVGRKVSHSYGYGLLDAGAMVALAQN  440
Beluga_X1               TLEANKNLTWRDMQHLVVQTSKPAHLNANDWATNGVGRKVSHSYGYGLLDAGAMVALAQN  440
Dolphin                 TLEANKNLTWRDMQHLVVQTSKPAHLNANDWATNGVGRKVSHSYGYGLLDAGAMVALAQN  440
Rabbit                  TLEANKNLTWRDMQHLVVQTSKPAHLNANDWTTNGVGRKVSHSYGYGLLDAGAMVALAQN  440
Cat                     TLEANKNLTWRDMQHLVVQTSKPAHLNANDWATNGVGRKVSHSYGYGLLDAGAMVTLAQN  440
Tiger                   TLEANKNLTWRDMQHLVVQTSKPAHLNANDWATNGVGRKVSHSYGYGLLDAGAMVTLAQN  384
Horse                   TLEANKNLTWRDMQHLVVQTSKPAHLNANDWATNGVGRKVSHSYGYGLLDAGAMVALAQN  440
Grey_seal_X1            TLEANKNLTWRDMQHLVVQTSKPAHLNANDWATNGVGRKVSHSYGYGLLDAGAMVTLAQN  439
Dog                     TLEANKNLTWRDMQHLVVQTSKPAHLNANDWATNGVGRKVSHSYGYGLLDAGAMVTLAHN  440
Bear                    TLEANKNLTWRDMQHLVVQTSKPAHLNANDWATNGVGRKVSHSYGYGLLDAGAMVTLAQN  440
American_mink           TLEANKNLTWRDMQHLVVQTSKPAHLNANDWATNGVGRKVSHSYGYGLLDAGAMVTLAQN  134
Ferret_X1               TLEANKNLTWRDMQHLVVQTSKPAHLNANDWATNGVGRKVSHSYGYGLLDAGAMVTLAQN  441
                        :**** ************:**.*. * :.** ******:************.*:*::*..

Darter_(fish)_b         WTTVGPQRQCVHTMLTDPRDIGNKLVFSRSVDACWGRPEYISSLEHVQARLTLSHNQRGK  500
Trout_X1                WTSVGPQHQCVLTMLSEPRDISSRLLFSKTLDACWGKPEYVSSLEHVQARLTLSYNHRGN  507
Darter_(fish)_a         WTSVRPQRKCVITMVSEPRNIGSRLLINKTVDACIGTDSHVTSLEHVQARLTLSYNRRGN  511
Frog                    WVTVGPQRKYVIDILSEPKDIGKRLEVRRKVEPCAGMSNYISTLEHVQARLSLSYNCRGD  498
Chicken                 WITVGPQRKCVIDVLTEPKDIGKRLEVRRKVDACLGKANYISRLEHAQARLTLSYNRRGD  501
Rock_dove               WTTVGPQRKCVIDVLTEPKDIGKRLEVRRKVDACLGKANYISRLEHAQARLTLSYNRRGD  501
Duck                    WTTVGPQRKCVIDVLTEPKDIGKRLEVRRKVDACLGKANYISRLEHAQARLTLSYNRRGD  501
Tiger_snake_X1          WTSVGPQRKCIIDILPEPKDIGKCLEVRQKVEACWGKANSVARLEHVQARLTLSYNRRGD  558
Human-I2                WTTVAPQRKCIIDILTEPKDIGKRLEVRKTVTACLGEPNHITRLEHAQARLTLSYNRRGD  500
Bat                     WTTVAPQRKCTVDILAEPRDIGKRLEVRKTVTACLGEAGHITRLEHAQARLTLSYNRRGD  500
Rat                     WTTVAPQRKCIIEILAEPKDIGKRLEVRKTVTACLGEPNHISRLEHVQARLTLSYNRRGD  500
Mouse                   WTTVAPQRKCIVEILVEPKDIGKRLEVRKAVTACLGEPNHITRLEHVQARLTLSYNRRGD  500
Cow                     WTTVAPQRKCTIDILTEPKDIGKRLEVRKTVTACLGEPSHITRLEHAQARLTLSYNRRGD  500
Goat                    WTTVAPQRKCIIDILTEPKDIGKRLEVRKTVTACLGQPSHITRLEHAQARLTLSYNRRGD  500
Sheep                   WTTVAPQRKCVIDILTEPKDIGKRLEVRKTVTACLGQPSHITRLEHAQARLTLSYNRRGD  500
Pig                     WTTVDPQRKCIIDILTEPKDIGKRLEVRKTVTACLGEPSHITRLEHAQARLTLSYNRRGD  500
Elephant                WTTVAPQRKCIIDILTEPKDIRKRLEVRKTVTACLGEPNHITRLEHAQARLTLSYNRRGD  500
Camel_X1                WTTVAPQRKCIIDILTEPKDIGKRLEVRKTVTACLGEPSHITQLEHAQARLTLSYNRRGD  532
Human_I1                WTTVAPQRKCIIDILTEPKDIGKRLEVRKTVTACLGEPNHITRLEHAQARLTLSYNRRGD  500
Gorilla                 WTTVAPQRKCIIDILTEPKDIGKRLEVRKTVTACLGEPNHITRLEHAQARLTLSYNRRGD  500
Macaque                 WTTVGPQRKCIIDILTEPKDIGKRLEVRKTVTACLGEPNHITRLEHAQARLTLSYNRRGD  500
Ground_squirrel_X1      WTTVAPQRKCIIDILTEPKDIGKRLEVRKTVTACLGEPNHITRLEHAQARLTLSYNRRGD  500
Lemur                   WTTVAPQRKCIIDILSEPKDIGKRLEVRKTVTACLGGPNHITRLEHAQARLTLSYNRRGD  500
Beluga_X1               WTTVTPQRKCTIDILTEPKDIGKRLEVRKTVTACVGEPSHITRLEHTQARLTLSYNRRGD  500
Dolphin                 WTTVTPQRKCIIDILTEPKDIGKRLEVRKTVTACVGEPSHITRLEHTQARLTLSYNRRGD  500
Rabbit                  WTTVAPQRKCIIDILTEPKDIGKRLEVRKTVTACLGEPSHITRLEHAQARLTLSYNRRGD  500
Cat                     WTTVAPQRKCIIDILTEPKDIGKRLEVRKMVTACLGEPSHITRLEHAQARLTLSYNRRGD  500
Tiger                   WTTVAPQRKCIIDILTEPKDIGKRLEVRKMVTACLGEASHINRLEHAQARLTLSYNRRGD  444
Horse                   WTTVAPQRKCIIDILTEPKDIGKRLEVRKTVTACLGEPSHITRLEHAQARLTLSYNRRGD  500
Grey_seal_X1            WTMVPPQRKCIIDILTEPKDIGKRLEVRKTVTACLGEPSHITRLEHAQARLTLSYNRRGD  499
Dog                     WTTXAPPRKCIIDILTEPKDIGKRLEVRKTVTACLGEPSHITRMEHAQARLTLSYNRRGD  500
Bear                    WTTVPPQRKCIIDILTEPKDIGKRLEVRKTVTACLGEPSHITRLEHAQARLTLSYNRRGD  500
American_mink           WTTVPPQRKCIIDILTEPKDIGKRLEVRKTVTACLGEPSHITRLEHAQARLTLSYNRRGD  194
Ferret_X1               WTTVPPQRKCIIDILTEPKDIGKRLEVRKTVTACLGEPSHITRLEHAQARLTLSYNRRGD  501
                        *    * ::    :: :*::* . * . : :  * *    :  :**.****:**:* **.
```

```
Darter_(fish)_b        LAIHLISPLGTRSTLLF-------------------------------------------  517
Trout_X1               LAIHLISPLGTRSTLLA-------------------------------------------  524
Darter_(fish)_a        LAIHLISPSGTRSTLLH-------------------------------------------  528
Frog                   LAIYLTSPMGTRSCLLA-------------------------------------------  515
Chicken                LAIHLVSPMGTRSTLLA-------------------------------------------  518
Rock_dove              LAIHLVSPMGTRSTLLA-------------------------------------------  518
Duck                   LAIHLVSPMGTRSTLLA-------------------------------------------  518
Tiger_snake_X1         LSIHLVSPMGTRSTLLA-------------------------------------------  575
Human-I2               LAIHLVSPMGTRSTLLA-------------------------------------------  517
Bat                    LAIHLVSPMGTRSTLLA-------------------------------------------  517
Rat                    LAIHLISPMGTRSTLLA-------------------------------------------  517
Mouse                  LAIHLISPMGTRSTLLA-------------------------------------------  517
Cow                    LAIHLVSPMGTRSTLLA-------------------------------------------  517
Goat                   LAIHLVSPMGTRSTLLA-------------------------------------------  517
Sheep                  LAIHLVSPMGTRSTLLA-------------------------------------------  517
Pig                    LAIHLVSPMGTRSTLLAARCVPSPLPSPSLPSHPCAFSHSLSQPQRQPSTAPRGIVGVLR  560
Elephant               LAIYLVSPMGTRSTLLA-------------------------------------------  517
Camel_X1               LAIHLVSPMGTRSTLLA-------------------------------------------  549
Human_I1               LAIHLVSPMGTRSTLLA-------------------------------------------  517
Gorilla                LAIHLVSPMGTRSTLLA-------------------------------------------  517
Macaque                LAIHLVSPMGTRSTLLA-------------------------------------------  517
Ground_squirrel_X1     LAIHLVSPMGTRSTLLA-------------------------------------------  517
Lemur                  LAIHLVSPMGTRSTLLA-------------------------------------------  517
Beluga_X1              LAIHLVSPMGTRSTLLA-------------------------------------------  517
Dolphin                LAIHLVSPMGTRSTLLA-------------------------------------------  517
Rabbit                 LAIHLVSPMGTRSTLLA-------------------------------------------  517
Cat                    LAIHLVSPMGTRSTLLA-------------------------------------------  517
Tiger                  LAIHLVSPMGTRSTLLA-------------------------------------------  461
Horse                  LAIHLVSPMGTRSTLLA-------------------------------------------  517
Grey_seal_X1           LAIHLVSPMGTRSTLLA-------------------------------------------  516
Dog                    LAIHLVSPMGTRSTMLA-------------------------------------------  517
Bear                   LAIHLVSPMGTRSTLLA-------------------------------------------  517
American_mink          LAIHLVSPMGTRSTLLA-------------------------------------------  211
Ferret_X1              LAIHLVSPMGTRSTLLA-------------------------------------------  518
                       *:*:* ** **** :*

Darter_(fish)_b        ------------------------------------------------------------  517
Trout_X1               ------------------------------------------------------------  524
Darter_(fish)_a        ------------------------------------------------------------  528
Frog                   ------------------------------------------------------------  515
Chicken                ------------------------------------------------------------  518
Rock_dove              ------------------------------------------------------------  518
Duck                   ------------------------------------------------------------  518
Tiger_snake_X1         ------------------------------------------------------------  575
Human-I2               ------------------------------------------------------------  517
Bat                    ------------------------------------------------------------  517
Rat                    ------------------------------------------------------------  517
Mouse                  ------------------------------------------------------------  517
Cow                    ------------------------------------------------------------  517
Goat                   ------------------------------------------------------------  517
Sheep                  ------------------------------------------------------------  517
Pig                    GLGFVLWDGPMSQESPILFAGGDVCAFLCVAPLHIFRVFLLRTFVLLGNNQRHFQPSGLG  620
Elephant               ------------------------------------------------------------  517
Camel_X1               ------------------------------------------------------------  549
Human_I1               ------------------------------------------------------------  517
Gorilla                ------------------------------------------------------------  517
Macaque                ------------------------------------------------------------  517
Ground_squirrel_X1     ------------------------------------------------------------  517
Lemur                  ------------------------------------------------------------  517
Beluga_X1              ------------------------------------------------------------  517
Dolphin                ------------------------------------------------------------  517
Rabbit                 ------------------------------------------------------------  517
Cat                    ------------------------------------------------------------  517
Tiger                  ------------------------------------------------------------  461
Horse                  ------------------------------------------------------------  517
Grey_seal_X1           ------------------------------------------------------------  516
Dog                    ------------------------------------------------------------  517
Bear                   ------------------------------------------------------------  517
American_mink          ------------------------------------------------------------  211
Ferret_X1              ------------------------------------------------------------  518
```

```
Darter_(fish)_b        -------------------PRPNDFSSEGFNDWAFMTTHSWGEDPQGEWTLEIENVAANG  558
Trout_X1               -------------------PRPKDYSSEGFNDWAFMTTHSWDEDPRGEWTLEIENVSEQG  565
Darter_(fish)_a        -------------------PRPHDYSSEGFNDWAFMTTHSWDENPTGVWTLEIENVAG-A  568
Frog                   -------------------PRLHDYSADGFNDWSFMTTHSWDEDPAGEWVLEIENVSK-N  555
Chicken                -------------------ARPHDYSADGFNDWAFMTTHSWDEDPSGEWLLEIENTSD-A  558
Rock_dove              -------------------ARPHDFSADGFNDWAFMTTHSWDEDPSGEWVLEIENTSD-A  558
Duck                   -------------------SRPHDYSADGFNDWAFMTTHSWDEDPSGDWLLEIENTSD-A  558
Tiger_snake_X1         -------------------ARPRDYSADGFNDWAFMTTHSWDEDPSGEWVLEIENTSE-A  615
Human-I2               -------------------ARPHDYSADGFNDWAFMTTHSWDEDPSGEWVLEIENTSE-A  557
Bat                    -------------------ARPHDYSADGFNDWAFMTTHSWDEDPSGEWVLEIENTSE-A  557
Rat                    -------------------ARPHDYSADGFNDWAFMTTHSWDEDPSGEWVLEIENTSE-A  557
Mouse                  -------------------ARPHDYSADGFNDWAFMTTHSWDEDPAGEWVLEIENTSE-A  557
Cow                    -------------------ARPHDYSADGFNDWAFMTTHSWDEDPSGEWVLEIENTSE-A  557
Goat                   -------------------ARPHDYSADGFNDWAFMTTHSWDEDPSGEWVLEIENTSE-A  557
Sheep                  -------------------ARPHDYSADGFNDWAFMTTHSWDEDPSGEWVLEIENTSE-A  557
Pig                    AALGEVVWSVCRGLSVPFLHRPHDYSADGFNDWAFMTTHSWDEDPSGEWVLEVENTSE-A  679
Elephant               -------------------ARPHDYSADGFNDWAFMTTHSWDEDPSGEWVLEIENTSE-A  557
Camel_X1               -------------------ARPHDYSADGFNDWAFMTTHSWDEDPSGEWVLEIENTSE-A  589
Human_I1               -------------------ARPHDYSADGFNDWAFMTTHSWDEDPSGEWVLEIENTSE-A  557
Gorilla                -------------------ARPHDYSADGFNDWAFMTTHSWDEDPSGEWVLEIENTSE-A  557
Macaque                -------------------ARPHDYSADGFNDWAFMTTHSWDEDPSGEWVLEIENTSE-A  557
Ground_squirrel_X1     -------------------ARPHDYSADGFNDWAFMTTHSWDEDPSGEWVLEIENTSE-A  557
Lemur                  -------------------ARPHDYSADGFNDWAFMTTHSWDEDPSGEWVLEIENTSE-A  557
Beluga_X1              -------------------ARPHDYSADGFNDWAFMTTHSWDEDPSGEWVLEIENTSE-A  557
Dolphin                -------------------ARPHDYSADGFNDWAFMTTHSWDEDPSGEWVLEIENTSE-A  557
Rabbit                 -------------------ARPHDYSADGFNDWAFMTTHSWDEDPSGEWVLEIENTSE-A  557
Cat                    -------------------ARPHDYSADGFNDWAFMTTHSWDEDPSGEWVLEIENTSE-A  557
Tiger                  -------------------ARPHDYSADGFNDWAFMTTHSWDEDPSGEWVLEIENTSE-A  501
Horse                  -------------------ARPHDYSADGFNDWAFMTTHSWDEDPSGEWVLEIENTSE-A  557
Grey_seal_X1           -------------------ARPHDYSADGFNDWAFMTTHSWDEDPSGEWVLEIENTSE-A  556
Dog                    -------------------ARPHDYSADGFNDWAFMTTHSWDEDPSGEWVLEIENTSE-A  557
Bear                   -------------------ARPHDYSADGFNDWAFMTTHSWDEDPSGEWVLEIENTSE-A  557
American_mink          -------------------ARPHDYSADGFNDWAFMTTHSWDEDPSGEWVLEIENTSE-A  251
Ferret_X1              -------------------ARPHDYSADGFNDWAFMTTHSWDEDPSGEWVLEIENTSE-A  558
                                            * .*:*::*****:*******.*:* * * **:**.:

Darter_(fish)_b        HDYAVLSQFTLFLWGTGPSVISPSSSDFPRPSNNSCKTFDAQQICIECSPGFSLFLQ---  615
Trout_X1               HDYGVLSQFTLILYGTGSSSINPLSPDFPRPSNNSCKTFDAQQICIECSPGFSLFLQ---  622
Darter_(fish)_a        SDYGTLTQFTLVLYGTASASS--SSSDKAQPGSDNCKTLDLRQICIECNAGYYLFKQ---  623
Frog                   NNYGTLTQFVLVLYGTASETP--GL--SRQFDGDGYRNVASSQSCIVCEEGYFLHQK---  608
Chicken                NNYGTLTKFTLVLYGTATDPP--GL--SNQLESSGCKTLTPSQTCVVCEEGYYLHQK---  611
Rock_dove              NNYGTLTKFTLVLYGTATESP--SL--SNQLESSGCKTLTPSQTCVVCEEGYYLHQK---  611
Duck                   NNYGTLTKFTLVLYGTATDSP--SL--SNQLESSGCKTLTPSQTCVVCEEGYYLHQK---  611
Tiger_snake_X1         NNYGTLTKFVLVLYGTGPDLP--DL--SNQFESSGCKTLVTNQACVVCEEGFYLHQK---  668
Human-I2               NNYGTLTKFTLVLYGTAPEG----L--PVPPESSGCKTLTSSQACCARKASPCTRRAVSS  611
Bat                    NNYGTLTKFTLVLYGTAPEG----P--PTPPESSGCKTLTSSQACVVCEEGFSLHEK---  608
Rat                    NNYGTLTKFTLVLYGTASEG----L--SVPPESSGCKTLTSSQACVVCEEGFSLHQK---  608
Mouse                  NNYGTLTKFTLVLYGTAPEG----L--STPPESSGCKTLTSSQACVVCEEGYSLHQK---  608
Cow                    NNYGTLTKFTLVLYGTAPEG----L--PTPPESIGCKTLTSSQACVVCEEGFSLHQK---  608
Goat                   KNYGTLTKFTLVLYGTAPEG----L--PTPPESIGCKTLTSSQACVVCEEGFSLHQK---  608
Sheep                  KNYGTLTKFTLVLYGTAPEG----L--PTPPESIGCKTLTSSQACVVCEEGFSLHQK---  608
Pig                    NNYGTLTKFTLVLYGTAPEG----L--PTPPESIGCKTLTSGQACVVCEEGFSLHQK---  730
Elephant               NNYGTLTKFTLVLYGTAPEG----L--PAPPESSGCKTLTSSQACVVCEEGFSLHQK---  608
Camel_X1               NNYGTLTKFTLVLYGTASEG----L--PTPPESIGCKTLTSGQACVVCEEGFSLHQK---  640
Human_I1               NNYGTLTKFTLVLYGTAPEG----L--PVPPESSGCKTLTSSQACVVCEEGFSLHQK---  608
Gorilla                NNYGTLTKFTLVLYGTAPEG----L--PVPPESSGCKTLTSSQACVVCEEGFSLHQK---  608
Macaque                NNYGTLTKFTLVLYGTAPEG----L--PIPPESSGCKTLTSSQACVVCEEGFSLHQK---  608
Ground_squirrel_X1     NNYGTLTKFTLVLYGTAPEG----L--PTPPESSGCKTLTSSQACVVCEEGFSLHQK---  608
Lemur                  NNYGTLTKFTLVLYGTAPEG----L--PTPPESSGCKTLTSSQACVVCEEGFSLHQK---  608
Beluga_X1              NNYGTLTKFTLVLYGTAPEG----L--PTPPESIGCKTLTSSQACVVCEEGFSLHQK---  608
Dolphin                NNYGTLTKFTLVLYGTAPEG----L--PTPPESIGCKTLTSSQACVVCEEGFSLHQK---  608
Rabbit                 NNYGTLTKFTLVLYGTAPEG----P--PAPPESSGCKTPTSSQACVVCEEGFSLHQK---  608
Cat                    NNYGTLTKFTLVLYGTAPEG----L--HAPPESSGCKTLTSSQACVVCEEGFSLHQK---  608
Tiger                  NNYGTLTKFTLVLYGTAPEG----L--HTPPESSGCKTLXXXXXXAVCEEGFSLHQK---  552
Horse                  NNYGTLTKFTLVLYGTAPEG----L--PTPPESSGCKTLTSSQACVVCEEGFSLHQK---  608
Grey_seal_X1           NNYGTLTKFTLVLYGTAPEG----S--HPPPESSGCKTLTTSQACVVCEEGFSLLQK---  607
Dog                    NNYGTLTKFTLVLYGTAPEG----L--HTPPESSGCKTLTTSQACVVCEEGFSLHQK---  608
Bear                   NNYGTLTKFTLVLYGTAPEG----P--HTPPESSGCKTLTTSQACVVCEEGFSLHQK---  608
American_mink          NNYGTLTKFTLVLYGTAPEG----P--HTPPESSGCKTLTTSQACVVCEEGFSLHQK---  302
Ferret_X1              NNYGTLTKFTLVLYGTAPEG----P--HTPPESSGCKTLTTSQACVVCEEGFSLHQK---  609
                        :*..*::*.*.*:**.                 . . :.          . .
```

```
Darter_(fish)_b       GCVKLCPPGFTSGPQLLNLSLENWVDLSSVQACLPCNPACLTCSGSGPTDCLSCPPHSHL  675
Trout_X1              GCVKLCPPGFTSGPQLLNLSLDNWVDLSSVQACLPCHPACLTCSGPGPNDCLSCPPHSHL  682
Darter_(fish)_a       GCVKECPEGFSVGSQLLNYTVGNFIPPASVPACLPCPPPCLTCSSLSPMACLSCPPHSSL  683
Frog                  SCIKSCPSGFTSSIQNMHYTLDNNIEPLLVNVCVPCHPSCATCKGTTINDCLTCPAHSHY  668
Chicken               SCLKRCPPGFAPGVQNTHYNLENSMEPIAPQLCLPCHPSCATCTGPGPNQCLTCPAHSHF  671
Rock_dove             SCLKHCPPGFAPGVQSTHYSLENSVEPIAPQLCLPCHPSCATCAGPGPNQCLTCPAHSHF  671
Duck                  SCLKRCPPGFAPGVQSTHYNLENSVEPIAPHLCLPCHPSCATCAGPGPNQCLTCPAHSHF  671
Tiger_snake_X1        TCLKTCPPGFAPALAPPPLENSLDPHLLQPWLCVPCHPSCATCLGPSASQCLSCPAHAHY  728
Human-I2              TALQGSPPKSSIRTIAPR----MTWRPSGPASAPPATPHVPHARG---------------  652
Bat                   SCVQHCPDGFAPQVLSTHYSAENDVEPIRASVCAPCHPSCATCRGPAPTDCLSCPGHFFL  668
Rat                   SCVQRCPPGFTPQVLDTHYSTENDVEIIRASVCTPCHASCATCQGPAPTDCLSCPSHASL  668
Mouse                 SCVQHCPPGFIPQVLDTHYSTENDVEIIRASVCTPCHASCATCQGPAPTDCLSCPSHASL  668
Cow                   NCVQHCPPGFAPQVLDTHYSTENDVEIIRASVCTPCHASCATCQGPAPTDCLSCPSHASL  668
Goat                  NCVQHCPPGFAPQVLDTHYSTENDVEIIRASVCTPCHTSCATCQGPAPTDCLSCPSHASL  668
Sheep                 NCVQHCPPGFAPQVLDTHYSTENDVEIIRASVCTPCHTSCATCQGPAPTDCLSCPSHASL  668
Pig                   TCVQHCPPGFAPQVLDTHYNTENDVEIIRASVCVPCHASCATCQGPAPTDCLTCPSHASL  790
Elephant              TCVQHCPPGFTPQVLNTHYSTENDVEIIRASVCTPCHASCATCQGPVFTDCLSCPSHASL  668
Camel_X1              SCVQHCPPGFAPQVLDTHYSTENDVETIRASVCAPCHASCATCQGPAPTDCLSCPSHASL  700
Human_I1              SCVQHCPPGFAPQVLDTHYSTENDVETIRASVCAPCHASCATCQGPALTDCLSCPSHASL  668
Gorilla               SCVQHCPPGFAPQVLDTHYSTENDVETIRASVCAPCHASCATCQGPAPTDCLSCPSHASL  668
Macaque               SCVQHCPPGFAPQVLDTHYSTENDVETIRASVCAPCHASCATCQGPAPTDCLSCPSHASL  668
Ground_squirrel_X1    SCVQHCPPGFTPQVLDTHYSTENDMEIIRASVCAPCHASCATCQGPAPTDCLSCPSHASL  668
Lemur                 SCVQHCPPGFAPQVLDTHYSTENDVETIRASVCAPCHPSCATCQGPAPTDCLSCPSHASL  668
Beluga_X1             SCVQRCPPGFAPQVLDTHYSTENNVEIIRASVCAPCHASCATCQGPAPTDCLSCPSHASL  668
Dolphin               SCVQRCPPGFTPQVLDTHYSTENNVEIIRASVCAPCHASCATCQGPAPTDCLSCPSHASL  668
Rabbit                SCVQHCPPGFAPQVLDTHYSTENDVETIRASVCIPCHSSCATCRGPAPTDCLSCPSHASL  668
Cat                   SCVQHCPPGFTPQVLDTHYSTENDVEIIRASVCAPCHASCATCQGLAPTDCLSCPSHASL  668
Tiger                 SCVQHCPPGFTPQVLDTHYSTENDVEIIRASVCAPCHASCATCQGLAPTDCLSCPSHASL  612
Horse                 SCVQHCPPGFTPQVLNTHYSTENDVETIRASVCAPCHVSCATCQGPAPTDCLTCPSHASL  668
Grey_seal_X1          SCVQRCPPGFTPQVLDTHYSTDNDVEIIRASVCAPCHSSCATCQGLAPTDCLSCPSHASL  667
Dog                   SCVQHCPSGFTPQVLDTHYSTENDVEIIRASVCAPCHTSCATCQGLAPTDCLSCPSHASL  668
Bear                  SCVQHCPPGFTPQVLDTHYSTENDVEIIRASVCAPCHSSCATCQGLAPTDCLSCPSHASL  668
American_mink         TCVQHCPPGFTPQVLDTHYSTENDVETIRASVCTPCHSSCATCQGLAPTDCLSCPSH---  359
Ferret_X1             TCVQHCPPGFTPQVLDTHYSTENDVETIRASVCTPCHSSCATCQGLAPTDCLSCPSHASL  669
                       .:: .*                          . *.      . .

Darter_(fish)_b       V--LTSCLHQNQVQRKSPLAGEVQGERAQPEGG-IPAAAEDRGEPPGLGVAPSSQLPVVM  732
Trout_X1              V--LTACLHQNQVQRKSPTGQVLQGEGSGPRESPVGSEQGGGGEPPGPNLGLSSPLITLL  740
Darter_(fish)_a       DPNSGTCLHLNQYMRESPGSFTVGQG---N-P-------------G-VQPQLNSRLPITV  725
Frog                  NLVDYSCTHQTQRSRESPTLKDSGHD----------------------YISGTSNLPFIV  706
Chicken               SSLDLSCSHQTQSSRASPALVEGEGQ---S------------------EAPPPANPLVLI  710
Rock_dove             SSLDLSCSHQTQSSRASPALADSEGL---A------------------ETPSAANLPVLI  710
Duck                  SSLDLSCSHQTQSSRASPALAEGEGP---A------------------EAPPAANLPILI  710
Tiger_snake_X1        NSQEHTCSHQMQSSRASPAVGGEG----------------------MAPASPPSNLPLLV  766
Human-I2              ------------------------------------------RP----------------  654
Bat                   DPLEQVCSRQSQSSRESPKQPPLP------PTPPAPEEAEAEAEPRPRAGLLPSHLPEVV  722
Rat                   DPVEQTCSRQSQSSRESRPQ-----------QPPPALRPEVEVEPRLRAGL-ASHLPEVL  716
Mouse                 DPVEQTCSRQSQSSRESRPQ-----------QQPPALRPEVEMEPRLQAGL-ASHLPEVL  716
Cow                   DPVEQTCSRQSQSSRESHQQ-----Q---PPPPPRPPPAEVATEPRLRADLLPSHLPEVV  720
Goat                  DLVERTCSRQSQSSRESHQQ-----Q---PPPP---PPVEVASEPRLRADLLPSHLPEVV  717
Sheep                 DLVERTCSRQSQSSRESHQQ-----Q---PPPP---PPVEVASEPRLRADLLPSHLPEVV  717
Pig                   DPVEQTCSRQSQSSRESPPQQLP-------RPPP-PSPQEEEAEPRVRAGQLPSHLPEVV  842
Elephant              DPVTQTCSRQSQSSRESPLQQQQQQQ---QQPPPQQPVQDVETEPRPRAGLLPSHLPEVV  725
Camel_X1              DPVEQTCSRQSQSSRESPQQ----------Q-PPRPPPLEVETEPRLRAGLLPSHLPELV  749
Human_I1              DPVEQTCSRQSQSSRESPPQ----------QQPP-RLPPEVEAGQRLRAGLLPSHLPEVV  717
Gorilla               DPVEQTCSRQSQSSRESPPQ----------QQPP-RLPPEVEAGQRLRAGLLPSHLPEVV  717
Macaque               DPVEQTCSRQSQSSRESPPQ----------QQPP-RLPPEMEAGPRLRAGLLPSHLPEVV  717
Ground_squirrel_X1    DPVEQTCSRQSQSSRESPEQ----------QQPP-GLSPEVEAEPRLQAGLLPSHLPEVV  717
Lemur                 DPVEQTCSRQSQSSRESPPQ----------QQPPPRPPLEVGTEPRLRAGQLPSHLPEVV  718
Beluga_X1             DPVEQTCSRQSQSSRESPQP----------QQP---PPTEVEAEPRLREGLLPSHLPEVV  715
Dolphin               DPVEQTCSRQSQSSRESPQP----------QQP---PPAEVEAEPRLREGLLPSHLPEVV  715
Rabbit                DPVQQACSRQGQSSRESPPQ----------QLPPP-PPPGAGGEPRLQAGLLPSHLPEVV  717
Cat                   DPVEQTCSRQSQSSRESPEQ----------QPP--RPPPEVEAEPRARAELLPSHLPEVV  716
Tiger                 DPVEQTCSRQSQSSRESPEQ----------QPP--RPPPEVEAEPRARAELLPSHLPEVV  660
Horse                 DPVELTCSRQSQSSRESPQQ----------QRPG--PPPEVQAEPRLRAGLLPSHLPEVV  716
Grey_seal_X1          DPVEQTCSRQSQSSRESPQQ----------RPPP--PPAEVDTEPRPRAGLPPSHLPEVV  715
Dog                   DPVEQTCSRQSQSSRESPQQ----------QPP--RPPAEVEAEPRPRAGLLPSHLPEVV  716
Bear                  DPVEQTCSRQSQSSRESPQQ----------QPP--RPPAEVEAEPRPRAGLLPSHLPEVV  716
American_mink         ------------------------------------------------------------  359
Ferret_X1             DPVEQTCSRQSQSSRESPQQ----------RPPPQRLPTEVEAEPRPRAGLLPSHLPEVV  719
```

```
Darter_(fish)_b      AVLGCAFILAAFVGVFLLLQMRSGGAFWGWRTKLPSVCS---QTRGVRVGFGFGFGQGQE  789
Trout_X1             AVLSCAFILAAFAGVFLLLQLRSGGAPWARRTKLHSVETGRGASGGIRVGIGFGLGLGWE  800
Darter_(fish)_a      AVLSCMAIIATFAGTFLLLQLRSGALLKMPSLE---------------------------  758
Frog                 AILSCLFIIVVFGSIFLFLQLRSGGVLGRKKLY--M----------------------LD  742
Chicken              ASLSCVLIVVIFITVFLVLQVRSGFSLRGVKVY--A----------------------LD  746
Rock_dove            ASLSCVLIVIIFVTVFLVLQARSGFSLRGVKVY--A----------------------LD  746
Duck                 ASLSCVFIVIIFVTVFLVLQARSGFSLRGVKVY--A----------------------LD  746
Tiger_snake_X1       AGLSCAFIVLVFVTIFLVLQLWSGPCRRGYKVY--S----------------------LE  802
Human-I2             ------------------------------------------------------------  654
Bat                  AGLSCAFIVLVFVTVFLVLQLRSGFSFRGVKVY--T----------------------MD  758
Rat                  AGLSCLIIALIFGIVFLFLHRCSGFSFRGVKVY--T----------------------MD  752
Mouse                AGLSCLIIVLIFGIVFLFLHRCSGFSFRGMKVY--T----------------------MD  752
Cow                  AGLSCAFIVLVFVTVFLVLQLRSGFSFRGVKVY--T----------------------MD  756
Goat                 AGLSCAFIVLVFVTVFLVLQLRSGFSFRGVKVY--T----------------------MD  753
Sheep                AGLSCAFIVLVFVTVFLVLQLRSGFSFRGVKVY--T----------------------MD  753
Pig                  AGLICALIVLVFVTVFLVLQLRSGFSFRGVKVY--T----------------------MD  878
Elephant             AGLSCAFIVLVFVTVFLVLQLRSGFRFRGVKVY--T----------------------MD  761
Camel_X1             AGLSCAFIVLVFVTVFLVLQLRSGFSFRGVKVY--T----------------------MD  785
Human_I1             AGLSCAFIVLVFVTVFLVLQLRSGFSFRGVKVY--T----------------------MD  753
Gorilla              AGLSCAFIVLVFVTVFLVLQLRSGFSFRGVKVY--T----------------------MD  753
Macaque              AGLSCAFIVLVFVTVFLVLQLRSGFSFRGVKVY--T----------------------MD  753
Ground_squirrel_X1   AGLSCAFIVLVFVTVFLVLQLRSGFSFRGVKVY--T----------------------MD  753
Lemur                AGLGCAFIVLVFVTVFLVLQLRSGFSFRGVKVY--T----------------------MD  754
Beluga_X1            AGLSCAFIVLVFVTVFLVLQLRSGFSFRGVKVY--T----------------------MD  751
Dolphin              AGLSCAFIVLVFVTVFLVLQLRSGFSFRGVKVY--T----------------------MD  751
Rabbit               AGLSCAFIVLVFATVFLVLQLRSGFSFRGVKVY--T----------------------MD  753
Cat                  AGLSCAFIVLVFVTVFLVLQLRSGFSFRGVKVY--T----------------------MD  752
Tiger                AGLSCAFIVLVFVTVFLVLQLRSGFSFRGVKVY--T----------------------MD  696
Horse                AGLSCAFIVLVFVTVFLVLQLRSGFSFRGVKVY--T----------------------MD  752
Grey_seal_X1         AGLSCAFIVLVFVTVFLVLQLRSGFSFRGVKVY--T----------------------MD  751
Dog                  AGLSCAFIVLVFVTVFLVLQLRSGFSFRGVKVY--T----------------------MD  752
Bear                 AGLSCAFIVLVFVTVFLVLQLRSGFSFRGVKVY--T----------------------MD  752
American_mink        ------------------------------------------------------------  359
Ferret_X1            AGLSCAFIVLVFVTVFLVLQLRSGFSFRGVKVY--T----------------------MD  755
```

```
Darter_(fish)_b        RKARVCYKGIPT-VWGDEDVMGY-QSESDSEEVDGHGERTAFIKTQSSI   836
Trout_X1               RQGRVSYKGIPT-VWRDEDQVTLGGSESDSEELDCHSERTAFIRTQSSL   848
Darter_(fish)_a        -------------------------------------------------   758
Frog                   SG-IISYKGIPSGVWQEE-----GFSESEIEETGAHSERKAFIKQQSTL   785
Chicken                SG-IISYKGLPSDIWQEE-----GPSESDIEECEVHNERTAFIRDQSAL   789
Rock_dove              SG-IISYKGLPSDIWQEE-----GPSESDGEENEAHSERTAFIRDQSAL   789
Duck                   SG-IISYKGLPSDIWQEE-----GPSESDGEEYEAHNERTAFIRDQSAL   789
Tiger_snake_X1         SGNLIAYKGLPSILWQEG-----LEEEEEEEEAAEGERTAFIRDQRL-    845
Human-I2               -------------------------------------------------   654
Bat                    RG-LISYKGLPPEAWQ-E-----CPSDSE--EDEGRGERTTFIRDQSVL   798
Rat                    RG-LISYKGLPPEAWQEE-----CPSDSE--EDEGRGERTAFIKDQSAL   793
Mouse                  RG-LISYKGLPPEAWQEE-----CPSDSE--EDEGRGERTAFIKDQSAL   793
Cow                    RG-LISYKGLPPEAWQEE-----CPSDSE--EDEGRGERTAFIKDQSAL   797
Goat                   RG-LISYKGLPPEAWQEE-----CPSDSE--EDEGRGERTAFIKDQSAL   794
Sheep                  RG-LISYKGLPPEAWQEE-----CPSDSE--EDEGRGERTAFIKDQSAL   794
Pig                    RG-LISYKGLPPEAWQEE-----CPSDSE--EDEGRGERTAFIKDQSAL   919
Elephant               RG-LISYKGLPPEAWQEE-----CPSDSE--EEEGQGERTAFIKDQSAL   802
Camel_X1               RG-LISYKGLPPEAWQEE-----CPSDSE--EDEGRGERTAFIKDQSAL   826
Human_I1               RG-LISYKGLPPEAWQEE-----CPSDSE--EDEGRGERTAFIKDQSAL   794
Gorilla                RG-LISYKGLPPEAWQEE-----CPSDSE--EDEGRGERTAFIKDQSAL   794
Macaque                RG-LISYKGLPPEAWQEE-----CPSDSE--EDEGRGERTAFIKDQSAL   794
Ground_squirrel_X1     RG-LISYKGLPPEAWQEE-----CPSDSE--EDEGRGERTAFIKDQSAL   794
Lemur                  RG-LISYKGLPPEAWQEE-----CPSDSD--EDEGRGERTAFIKDQSAL   795
Beluga_X1              RG-LISYKGLPPEAWQEE-----CPSDSE--EDEGRGERTAFIKDQSAL   792
Dolphin                RG-LISYKGLPPEAWQEE-----CPSDSE--EDEGRGERTAFIKDQSAL   792
Rabbit                 RG-LISYKGLPPEAWQEE-----CPSDSE--EDEGRGERTAFIKDQSVL   794
Cat                    RG-LISYKGLPPEAWQEE-----GPSDSE--EDEGRGERTAFIKDQSAL   793
Tiger                  RG-LISYKGLPPEAWQEE-----GPSDSE--EDEGRGERTAFIKDQSAL   737
Horse                  RG-LISYKGLPPEAWQEE-----CPSDSE--EDEGRGERTAFIKDQSAL   793
Grey_seal_X1           RG-LISYKGLPPEAWQEE-----CPSDSE--EDEGRGERTAFIKDQSAL   792
Dog                    RG-LISYKGLPPEAWQEE-----CPSDSE--EDEGRGERTAFIKDQSAL   793
Bear                   RG-LISYKGLPPEAWQEE-----CPSDSE--EDEGRGERTAFIKDQSAL   793
American_mink          -------------------------------------------------   359
Ferret_X1              RG-LISYKGLPPEAWQEE-----CPSDSE--EDEGRGERTAFIKDQSAL   796
```

Figure S4. Amino acid sequence alignment of a region of the furin polypeptides from a range of animal species. Accession Numbers of the polypeptide sequences used are given in Table 1. Alignments were carried out using Clustal Omega [1]. The last row shown is the consensus analysis given by the algorithm. Human sequences are highlighted in grey to aid comparison to other sequences. Important amino acids used for catalytic site of the enzyme, as discussed [7] are highlighted in yellow, with the S253–P255 region in green. Some of the differences in sequences from the human sequence are shown in blue.

References

1. Sievers, F.; Wilm, A.; Dineen, D.; Gibson, T.J.; Karplus, K.; Li, W.; Lopez, R.; McWilliam, H.; Remmert, M.; Söding, J. et al. Fast, scalable generation of high-quality protein multiple sequence alignments using Clustal Omega. *Mol. Syst. Biol.* **2011**, *7*, 539.
2. Damas, J.; Hughes, G.M.; Keough, K.C.; Painter, C.A.; Persky, N.S.; Corbo, M.; Hiller, M.; Koepfli, K.-P.; Pfenning, A.R.; Zhao, H. et al. Broad host range of SARS-CoV-2 predicted by comparative and structural analysis of ACE2 in vertebrates. *Proceedings of the National Academy of Sciences, U.S.A.* **2020**. *117*, 22311–22322. doi: doi.org/10.1073/pnas.2010146117.
3. Sun, J.; He, W.-T.; Wang, L.; Lai, A.; Ji, X.; Zhai, X.; Li, G.; Suchard, M.A.; Tian, J.; Zhou, J. et al. COVID-19: epidemiology, evolution, and cross-disciplinary perspectives. *Trends Mol. Med*. **2020**, *26*, 483–495. doi:10.1016/j.molmed.2020.02.008.
4. Hou, Y.; Zhao, J.; Martin, W.; Kallianpur, A.; Chung, M.K.; Jehi, L.; Sharifi, N.; Erzurum, S.; Eng, C.; Cheng, F. New insights into genetic susceptibility of COVID-19: an *ACE2* and *TMPRSS2* polymorphism analysis. *BMC Med*. **2020**, *18*, 216. doi:10.1186/s12916-020-01673-z.
5. Senapati, S.; Banerjee, P.; Bhagavatula, S.; Kushwaha, P.P.; Kumar, S. Contributions of human ACE2 and TMPRSS2 in determining host-pathogen interaction in COVID-19. *OSF Preprints* **2020**. doi.org/10.31219/osf.io/evuby.

6. Daly, J.L; Simonetti, B.; Klein, K.; Chen, K.-E.; Williamson, M.K.; Antón-Plágaro, C.; Shoemark, D.K.; Simón-Gracia, L.; Bauer, M.; Hollandi, R. et al. Neuropilin-1 is a host factor for SARS-CoV-2 infection. *Science* **2020**. doi:10.1126/science.abd3072.
7. Dahms, S.O.; Arciniega, M.; Steinmetzer, T.; Huber, R.; Than, M.E. Structure of the unliganded form of the proprotein convertase furin suggests activation by a substrate-induced mechanism. *Proceedings of the National Academy of Science USA* **2016**, *113*, 11196–11201. doi:10.1073/pnas.1613630113
